# A simulation-based comparative effectiveness analysis of policies to improve global maternal health outcomes

**DOI:** 10.1038/s41591-023-02311-w

**Published:** 2023-04-20

**Authors:** Zachary J. Ward, Rifat Atun, Gary King, Brenda Sequeira Dmello, Sue J. Goldie

**Affiliations:** 1 Center for Health Decision Science, Harvard T.H. Chan School of Public Health, Harvard University, Boston, MA USA; 2 Department of Global Health and Population, Harvard T.H. Chan School of Public Health, Harvard University, Boston, MA USA; 3 Department of Health Policy and Management, Harvard T.H. Chan School of Public Health, Harvard University, Boston, MA USA; 4 Department of Global Health and Social Medicine, Harvard Medical School, Harvard University, Boston, MA USA; 5 Institute for Quantitative Social Science, Harvard University, Cambridge, MA USA; 6 Maternal and Newborn Healthcare, Comprehensive Community Based Rehabilitation in Tanzania, Dar es Salaam, Tanzania; 7 Global Health Education and Learning Incubator, Harvard University, Cambridge, MA USA

**Keywords:** Health policy, Reproductive disorders

## Abstract

The Sustainable Development Goals include a target to reduce the global maternal mortality ratio (MMR) to less than 70 maternal deaths per 100,000 live births by 2030, with no individual country exceeding 140. However, on current trends the goals are unlikely to be met. We used the empirically calibrated Global Maternal Health microsimulation model, which simulates individual women in 200 countries and territories to evaluate the impact of different interventions and strategies from 2022 to 2030. Although individual interventions yielded fairly small reductions in maternal mortality, integrated strategies were more effective. A strategy to simultaneously increase facility births, improve the availability of clinical services and quality of care at facilities, and improve linkages to care would yield a projected global MMR of 72 (95% uncertainty interval (UI) = 58–87) in 2030. A comprehensive strategy adding family planning and community-based interventions would have an even larger impact, with a projected MMR of 58 (95% UI = 46–70). Although integrated strategies consisting of multiple interventions will probably be needed to achieve substantial reductions in maternal mortality, the relative priority of different interventions varies by setting. Our regional and country-level estimates can help guide priority setting in specific contexts to accelerate improvements in maternal health.

## Abstract (web summary)

Analyses from the Global Maternal Health microsimulation model revealed that a comprehensive strategy involving family planning and community-based interventions would have the largest impact in reducing maternal mortality, although the priority of different interventions need to vary by setting.

## Main

The Sustainable Development Goals (SDGs) set by the United Nations include a target to reduce the global maternal mortality ratio (MMR) to less than 70 maternal deaths per 100,000 live births by 2030, with no individual country exceeding 140 (ref. ^1^). While progress was made under the previous Millennium Development Goals, this area lags behind and the maternal mortality targets were not met^2^. Previous projections also found that although maternal deaths are projected to decline in the future, on current trends the projected decreases will fail to achieve the SDG maternal mortality target^3,4^. Although effective interventions exist to promote maternal health and address obstetric complications^5^, critical gaps in knowledge remain, especially around how these interventions may take place in various health systems in practice^6^. Monitoring progress and evaluating the effectiveness of strategies is further complicated by the lack of routine and complete information on maternal deaths from vital registration systems in many countries^7^, as well as underreporting and misclassification of maternal deaths, especially in early pregnancy and from complications of induced abortion and indirect obstetric causes^8,9^.

Statistical methods have been used by the United Nations^3,10^ and Institute for Health Metrics and Evaluation^11,12^ to estimate maternal health indicators based on the cross-sectional association between reported levels of maternal mortality and aggregate country-level factors, with per capita gross domestic product (GDP) being the largest driver of trends^13^. However, although these models can provide broad guidance to policymakers and offer insights into trends, the aggregate, cross-sectional nature of these models means that they are not suitable for exploring counterfactual scenarios to estimate the potential impact of realistic interventions. For example, although a cross-sectional association between GDP and maternal mortality may exist across countries, this does not imply that changes in GDP in a country over time would improve maternal health to a similar degree. Moreover, aggregate factors such as GDP do not offer actionable or realistic maternal health strategies for policymakers to consider.

Although economic growth can underpin progress, economic improvements alone are not sufficient, especially because the relationship between economic growth and health outcomes varies across countries, highlighting the need for more specific evidence on why some countries do better than others in preventing maternal deaths^14^. A previous analysis of success factors for reducing maternal and child mortality found that, although there is no standard formula, countries that have made substantial progress moved ahead in various areas, such as making progress across multiple sectors to address crucial health determinants and mobilizing partners across society using robust evidence for decision-making^14^. However, knowledge gaps remain in how to most successfully phase, scale and adapt such strategies for different countries.

Recommendations of specific interventions for countries to consider have been provided by the Disease Control Priorities project, a seven-year international collaborative effort to synthesize evidence and provide recommendations for health priorities in low- and middle-income countries (LMICs) for essential packages of interventions that provide good value for money and are feasible to implement^15–19^. Furthermore, analyses for the Lancet Commission on Investing in Health, which involved modeling scale-up of a range of such interventions in a set of LMICs to the existing rate in the ‘best-performing’ countries, found that countries could reach coverage levels of most interventions of at least 90% by 2035, yielding a large reduction in the average MMR of 74 countries, that is, from around 350 for the ‘low-coverage’ scenario to around 150 for the ‘high-coverage’ scenario^20,21^.

Updated analyses, however, estimated that maintaining the trends observed from 2010 to 2016 would not be sufficient to achieve convergence of coverage indicators for maternal mortality, with more aggressive scale-up of maternal health interventions needed, including structural investments in health systems, improvements in quality of care and use of robust evidence in a timely way for policy decisions and accountability^22^. While these analyses can provide broad guidance to policymakers, the use of a deterministic linear modeling approach^23^ means assumed relationships between coverage indicators and health outcomes may not hold in practice, and lack of accounting for parameter uncertainty, for example, intervention effect sizes, means that the results may not be robust to the large uncertainty surrounding maternal mortality indicators.

Previous efforts using a structural modeling approach and accounting for the ‘natural history’, that is, disease processes and outcomes in the absence of intervention, of maternal mortality have been undertaken, which examined the role of a comprehensive set of interventions, including health system linkages and family planning interventions, focused on specific countries such as India, Afghanistan, Nigeria and Mexico^24–27^. These analyses highlighted the importance of contextualizing the model to local situations and the impact of early intensive efforts to improve family planning and control of fertility choices, accompanied by a stepwise effort to scale up capacity for integrated maternal health services. However, these analyses did not provide global estimates.

To address these gaps, we developed a structural, individual-level model of global maternal health^4^, which can offer predictions of complex systems and, because it is based on a defined system of causal components and their relationships, allows realistic counterfactual scenarios to be evaluated^28^. This type of microsimulation modeling is increasingly recognized in epidemiology as another approach for causal inference because it uses the robust foundations of graphical causal models and can explore the effects of complex interventions that occur over prolonged periods of time^29^. In this study, we simulated various policy interventions aimed at improving maternal health, including individual interventions and integrated strategies consisting of multiple interventions. We provide estimates of their comparative impact, both globally and by region and country, to help guide decision-makers in different contexts seeking to accelerate improvements in maternal health. Table 1 summarizes our findings and policy implications.

**Table 1:** Policy summary

Background	The SDGs set by the United Nations include a target to reduce the global MMR to less than 70 maternal deaths per 100,000 live births by 2030, with no individual country exceeding 140. However, on current trends the goals are unlikely to be met. Evaluating the comparative effectiveness of various policies in different countries can help provide guidance for policymakers seeking to improve maternal health in their own context.
Main findings and limitations	We developed the GMatH simulation model to model the reproductive life courses of individual women in 200 countries and territories. Using the model, we evaluated the impact of various policies of scale-up, informed by the mean level of high-income countries, from 2022 to 2030. We found that individual interventions generally yielded small reductions in maternal mortality, with the largest individual impact coming from increasing the number of women who deliver at facilities (projected global MMR in 2030 of 111, 95% UI = 94–129), followed by improving the quality of care at facilities (projected global MMR of 140, 95% UI = 113–159). Instead, we found that integrated strategies are probably needed to substantially accelerate improvements in maternal mortality. A strategy to simultaneously increase facility births, improve the availability of clinical services and quality of care at facilities, and improve linkages to care would yield a projected global MMR of 72 (95% UI = 58–87) in 2030, with large improvements in LMICs. A comprehensive strategy adding family planning and community-based interventions would have an even larger impact, with a projected MMR of 58 (95% UI = 46–70) in 2030. Limitations to our approach include the lack of data for many variables of interest for some locations, meaning that we had to make certain assumptions when developing the model. Although our estimates can provide insight into the comparative effectiveness of policies to improve maternal health, we recognize the need for decision-makers to also consider the costs of implementing such policies. We also did not model the clinical impact of policies on indirect maternal deaths (for example, malaria and HIV) because these would probably require other targeted (that is, non-obstetric) health system interventions. Therefore, our estimates of total maternal deaths averted by some policies may be conservative.
Policy implications	Although integrated strategies consisting of multiple interventions will probably be needed to achieve substantial reductions in maternal mortality, the relative priority of different interventions varies by setting, highlighting the importance of considering the local context when designing and implementing policies. For example, the family planning strategy has a large impact in areas of Asia and sub-Saharan Africa where fertility is still high, but it has little impact in other areas. Even within regions there are differences in priority setting by country. In addition to insights from our global estimates, our regional and country-level comparative effectiveness estimates can therefore help guide planning and priority setting in various contexts to accelerate improvements in maternal health.

## Results

### Interventions and strategies

We compared the effectiveness of various policies to reduce maternal mortality, ranging from individual interventions to integrated strategies. Following previously used typologies for maternal health interventions^16–19,24^, we categorized policies as family planning interventions, for example, contraception, medical abortion; community-based interventions, for example, antenatal care (ANC), skilled birth attendants (SBAs) for home births; facility-based interventions, for example, facility births, availability of clinical services; and system-relevant interventions, for example, linkages to care and quality of care (Table 2). We also modeled combinations of individual interventions to evaluate the impact of integrated strategies (Table 3).

**Table 2:** Maternal health policy interventions

Intervention	Description	Modeled implementation	Estimated global mean (95% UI), 2022	2030 target (minimum coverage)
**Family planning interventions**				
Contraception	Reduce unmet need through availability and access to contraception for spacing and limiting	Reduce unmet need for contraception. No change in the methods used or desired family size	Met need: 40.3% (30.6–48.4%)	Met need: 80%
Medical abortion	Reduce mortality due to ‘unsafe’ abortion through availability and access to ‘safe’ medical abortion	Increase proportion of abortions that are ‘safe’. No change in probability of abortion	89.2% (83.8–93.5%)	95%
**Community-based interventions**				
ANC	Improve health status (for example, anemia) during pregnancy and knowledge of complication danger signs	Increase probability and number of ANC visits	*P*_any_: 66.8% (47.4–78.4%) No. of visits | any: 5.3 (3.8–6.5)	*P*_any_: 95% No. of visits | any: at least 4
SBAs for home deliveries (SBA-home)	Improve availability and access to trained SBA for home births and use of basic interventions by SBA	Increase proportion of home births attended by an SBA, as well as use of clean birth kits to reduce sepsis, and management of moderate hemorrhage. No change in SBA referral probabilities	SBA: 37.1% (27.7–52.9%) Clean delivery: 58.4% (50.4–65.4%) Hemorrhage management: 8.7% (4.6–13.6%)	SBA: 80% Clean delivery: 90% Hemorrhage management: 50%
**Facility-based interventions**				
Facility births	Increase proportion of women that give birth in medical facilities instead of at home, where they will have access to varying levels of obstetrical care depending on the level of the facility	Increase proportion of facility births. No change in distribution of facility levels	83.0% (80.5–86.0%)	99%
*Availability of clinical services*				
Non-EmOC services	Improve availability of basic interventions at non-EmOC facilities	Improve availability of clean delivery, active management of the third stage of labor (AMTSL), partograph, assisted delivery for moderate obstructed labor, treatment of moderate pregnancy-related hypertension, moderate hemorrhage and sepsis at non-EmOC facilities. No change in quality of care	Clean delivery: 77.6% (67.8–86.2%) AMTSL: 76.6% (66.7–86.4%) Partograph: 50.5% (34.1–72.2%) Assisted delivery: 53.4% (46.5–59.5%) Hypertension management: 32.3% (21.7–43.5%) Hemorrhage management: 54.6% (47.3–62.7%) Sepsis management: 79.0% (72.2–86.3%)	90%
Basic EmOC (BEmOC) services	Improve availability of basic interventions at BEmOC facilities	Improve availability of clean delivery, AMTSL, partograph, assisted delivery for moderate obstructed labor, treatment of ectopic pregnancy, moderate pregnancy-related hypertension, moderate hemorrhage and sepsis at BEmOC facilities. No change in quality of care	Clean delivery: 93.2% (87.5–97.2%) AMTSL: 92.6% (85.3–95.8%) Partograph: 83.2% (75.8–93.0%) Assisted delivery: 66.4% (61.5–72.0%) Ectopic pregnancy management: 92.2% (86.1–96.0%) Hypertension management: 66.2% (59.3–73.1%) Hemorrhage management: 78.9% (74.3–82.2%) Sepsis management: 89.8% (85.6–93.4%)	95%
Comprehensive EmOC (CEmOC) services	Improve the availability of comprehensive set of interventions at CEmOC facilities	Improve availability of clean delivery, AMTSL, partograph, assisted delivery for moderate-to-severe obstructed labor, treatment of ectopic pregnancy, moderate-to-severe pregnancy-related hypertension, moderate-to-severe hemorrhage and sepsis at CEmOC facilities. No change in quality of care	Clean delivery: 97.6% (95.4–98.9%) AMTSL: 96.5% (94.7–98.1%) Partograph: 90.5% (87.1–93.7%) Assisted delivery: 96.4% (91.8–97.7%) Ectopic pregnancy management: 96.8% (92.9–99.0%) Hypertension management: 92.4% (88.8–95.0%) Hemorrhage management: 94.3% (92.4–96.1%) Sepsis management: 97.5% (95.4–98.8%)	99%
**System-relevant interventions**				
Quality of care	Improve the quality of care that women receive at health facilities	Improve complication recognition and quality of care at facilities. No change in availability of clinical services	Non-EmOC: 47.6% (42.9–55.7%) BEmOC: 85.9% (79.1–92.8%) CEmOC: 93.3% (92.3–94.2%)	Non-EmOC: 90% BEmOC: 95% CEmOC: 99%
*Linkages to care*				
Referral	Improve recognition and referral of pregnancy complications to health facilities	Increase referral of complications from SBA-home, non-EmOC and BEmOC	SBA-home: 73.5% (71.1–76.2%) Non-EmoC: 92.8% (90.4–94.5%) BEmOC: 96.6% (95.4–97.8%)	SBA-home: 90% Non-EmOC: 95% BEmOC: 99%
Transportation	Improve availability of timely transportation for women seeking emergency care at a health facility	Increase transportation from home, SBA-home, non-EmOC and BEmOC	Home: 24.0% (20.0–28.2%) SBA-home: 61.1% (49.2–71.2%) Non-EmOC: 94.2% (91.4–95.8%) BEmOC: 97.7% (96.3–99.0%)	Home: 80% SBA-home: 85% Non-EmOC: 97% BEmOC: 99%
Targeted transfers	Improve targeting of referrals to facilities that are a higher level than the current delivery site, as opposed to transferring a woman to another facility of the same level (that is, horizontal transfer)	Reduce horizontal transfers	Non-EmOC: 3.8% (2.7–5.0%) BEmOC: 42.4% (36.4–51.2%)	Non-EmOC: 1% BEmOC: 10%

Estimated global means (and 95% UIs) for 2022 are weighted by population (calculated across countries and demographic subgroups). The 2030 targets are informed by the 2022 estimated means among high-income countries.

**Table 3:** Integrated strategies to reduce maternal mortality

		Family planning		Community-based		Facility-based				System-relevant			
Strategy	Description	Contraception	Medical abortion	ANC	SBA-home	Facility births	Non-EmOC services	BEmOC services	CEmOC services	Referral	Transportation	Targeted transfers	Quality of care
Family planning	Improve the ability of women to achieve their fertility preferences	X	X										
Community + linkages	Improve community-based pregnancy care and referral pathway for emergency care at health facilities			X	X					X	X	X	
Facilities + linkages	Increase facility births, improve availability of clinical interventions at health facilities, and improve referral pathway for emergency care at health facilities					X	X	X	X	X	X	X	
Facilities + linkages + quality	Increase facility births, improve availability of clinical interventions at health facilities, and improve referral pathway and quality of care at health facilities					X	X	X	X	X	X	X	X
Comprehensive	Improve family planning, community-based, facility-based and system-relevant aspects of maternal health	X	X	X	X	X	X	X	X	X	X	X	X

### Global maternal mortality

We found that individual interventions yielded fairly small reductions in maternal mortality, with the largest individual impact coming from increasing the number of women who deliver at facilities (projected global MMR in 2030 of 111, 95% UI = 94–129), followed by improving the quality of care at facilities (projected global MMR of 140, 95% UI = 113–159) (Table 4). However, the potential gains from other individual policy interventions were relatively modest.

**Table 4:** Projected maternal indicators in 2030 according to income group and policy scenario

Policy scenario	Global			Low-income			Lower-middle income			Upper-middle income			High-income		
	Total maternal deaths	MMR	LTR	Total maternal deaths	MMR	LTR	Total maternal deaths	MMR	LTR	Total maternal deaths	MMR	LTR	Total maternal deaths	MMR	LTR
Baseline	327,438 (287,798–360,746)	167 (142–188)	0.43 (0.37–0.48)	163,919 (132,119–192,698)	400 (322–461)	1.70 (1.34–2.02)	140,292 (118,555–162,860)	149 (119–176)	0.41 (0.34–0.49)	19,109 (14,687–28,343)	37 (26–59)	0.07 (0.05–0.12)	4,118 (3,173–5,165)	15 (11–20)	0.03 (0.02–0.04)
**Family planning interventions**															
Contraception	288,490 (249,440–320,663)	152 (129–172)	0.37 (0.32–0.42)	137,752 (110,699–163,904)	356 (293–413)	1.38 (1.09–1.67)	129,906 (107,355–150,919)	144 (114–174)	0.38 (0.31–0.45)	16,883 (12,850–24,022)	32 (23–48)	0.06 (0.05–0.09)	3,949 (3,042–5,138)	15 (11–19)	0.03 (0.02–0.04)
Medical abortion	313,408 (272,408–345,812)	158 (134–177)	0.41 (0.35–0.46)	150,720 (122,210–177,357)	364 (294–423)	1.53 (1.21–1.83)	139,841 (116,957–162,255)	148 (120–175)	0.41 (0.33–0.49)	18,761 (14,074–28,158)	37 (25–58)	0.07 (0.05–0.12)	4,085 (3,187–5,308)	15 (11–20)	0.03 (0.02–0.04)
**Community-based interventions**															
ANC	319,671 (281,016–352,432)	162 (138–183)	0.42 (0.36–0.47)	158,589 (127,725–187,304)	385 (310–448)	1.64 (1.30–1.95)	138,185 (116,785–158,626)	146 (117–173)	0.40 (0.33–0.48)	18,831 (14,109–27,671)	37 (26–56)	0.07 (0.05–0.12)	4,066 (3,132–5,273)	15 (11–20)	0.03 (0.02–0.04)
SBA-home	312,363 (276,042–347,181)	158 (135–180)	0.41 (0.36–0.46)	155,763 (127,132–183,705)	378 (309–438)	1.62 (1.30–1.92)	133,567 (112,494–154,499)	140 (113–165)	0.39 (0.32–0.46)	18,918 (14,371–28,676)	37 (26–59)	0.07 (0.05–0.12)	4,115 (3,133–5,207)	15 (11–20)	0.03 (0.02–0.04)
**Facility-based interventions**															
Facility births	232,820 (206,057–259,701)	111 (94–129)	0.29 (0.25–0.33)	102,921 (84,118–123,009)	236 (195–293)	1.02 (0.80–1.25)	107,817 (89,453–126,003)	107 (82–129)	0.30 (0.24–0.36)	17,976 (13,788–25,476)	34 (24–53)	0.07 (0.05–0.10)	4,106 (3,168–5,202)	15 (11–20)	0.03 (0.02–0.04)
Non-EmOC services	327,054 (287,164–361,654)	166 (142–188)	0.43 (0.37–0.48)	163,522 (131,680–192,590)	398 (323–460)	1.70 (1.33–2.01)	140,280 (116,518–162,840)	148 (120–176)	0.41 (0.33–0.49)	19,135 (14,747–29,405)	37 (26–60)	0.07 (0.05–0.13)	4,118 (3,160–5,196)	15 (11–20)	0.03 (0.02–0.04)
BEmOC services	326,698 (287,057–360,323)	166 (141–188)	0.43 (0.37–0.48)	163,515 (131,591–193,276)	398 (322–462)	1.70 (1.34–2.02)	139,898 (117,759–161,560)	148 (120–175)	0.41 (0.34–0.48)	19,165 (14,665–28,745)	37 (26–59)	0.07 (0.05–0.13)	4,120 (3,159–5,149)	15 (11–20)	0.03 (0.02–0.04)
CEmOC services	321,367 (279,767–356,722)	163 (137–185)	0.42 (0.36–0.48)	160,958 (129,229–191,400)	392 (312–458)	1.67 (1.31–2.02)	137,268 (115,134–160,681)	145 (116–172)	0.40 (0.32–0.48)	19,047 (14,544–29,243)	37 (26–60)	0.07 (0.05–0.13)	4,094 (3,140–5,224)	15 (11–20)	0.03 (0.02–0.04)
**System-relevant interventions**															
Quality of care	280,693 (239,251–314,390)	140 (113–159)	0.37 (0.30–0.41)	131,685 (97,905–162,967)	313 (228–379)	1.35 (0.96–1.69)	126,479 (104,738–149,172)	132 (104–159)	0.37 (0.29–0.44)	18,520 (14,027–28,329)	36 (25–59)	0.07 (0.05–0.12)	4,010 (3,107–5,189)	15 (11–20)	0.03 (0.02–0.04)
Referral	324,183 (285,862–358,425)	165 (140–187)	0.43 (0.37–0.48)	161,942 (130,768–191,786)	394 (317–456)	1.68 (1.33–2.02)	139,030 (116,257–161,928)	147 (117–175)	0.41 (0.33–0.48)	19,094 (14,603–28,420)	37 (26–59)	0.07 (0.05–0.12)	4,116 (3,146–5,157)	15 (11–20)	0.03 (0.02–0.04)
Transportation	303,398 (269,404–336,013)	155 (133–176)	0.40 (0.35–0.45)	149,893 (122,436–176,800)	365 (296–425)	1.56 (1.24–1.86)	131,013 (110,185–151,342)	140 (112–165)	0.39 (0.32–0.46)	18,466 (13,983–28,568)	37 (26–59)	0.07 (0.05–0.12)	4,025 (3,103–5,200)	15 (11–20)	0.03 (0.02–0.04)
Targeted transfers	326,793 (288,463–360,511)	166 (143–189)	0.43 (0.37–0.48)	163,554 (131,713–192,322)	398 (321–461)	1.70 (1.35–2.02)	140,089 (116,843–162,753)	148 (119–176)	0.41 (0.34–0.49)	19,042 (14,330–28,588)	37 (26–59)	0.07 (0.05–0.12)	4,109 (3,182–5,205)	15 (11–20)	0.03 (0.02–0.04)
**Integrated strategies**															
Family planning	284,617 (246,658–316,213)	149 (128–169)	0.37 (0.31–0.41)	134,314 (107,178–159,117)	346 (282–400)	1.34 (1.05–1.62)	129,681 (106,767–151,073)	143 (114–174)	0.38 (0.30–0.45)	16,692 (12,797–23,092)	32 (23–45)	0.06 (0.04–0.09)	3,930 (2,943–5,058)	15 (10–19)	0.03 (0.02–0.04)
Community + linkages	270,519 (240,294–300,366)	136 (117–154)	0.35 (0.31–0.40)	127,056 (106,468–148,237)	304 (255–353)	1.30 (1.06–1.54)	121,623 (101,333–139,823)	129 (101–153)	0.36 (0.29–0.42)	17,841 (13,400–26,389)	35 (24–53)	0.07 (0.05–0.11)	3,999 (3,098–5,102)	15 (11–20)	0.03 (0.02–0.04)
Facilities + linkages	216,808 (191,796–241,777)	104 (87–121)	0.27 (0.23–0.31)	95,808 (78,947–113,958)	220 (180–272)	0.95 (0.75–1.16)	99,677 (81,220–117,294)	100 (74–121)	0.28 (0.21–0.34)	17,337 (12,948–25,041)	33 (23–51)	0.07 (0.05–0.10)	3,986 (2,996–5,202)	15 (11–19)	0.03 (0.02–0.04)
Facilities + linkages + quality	160,441 (138,852–184,190)	72 (58–87)	0.19 (0.15–0.23)	55,469 (41,928–72,194)	114 (81–162)	0.51 (0.34–0.72)	84,412 (67,333–101,698)	82 (56–103)	0.23 (0.17–0.29)	16,665 (12,496–24,155)	32 (22–49)	0.06 (0.04–0.10)	3,895 (2,998–4,935)	15 (11–20)	0.03 (0.02–0.04)
Comprehensive	130,682 (110,629–150,961)	58 (46–70)	0.15 (0.11–0.18)	37,045 (29,339–48,749)	72 (54–104)	0.29 (0.20–0.43)	75,769 (59,376–92,248)	77 (54–97)	0.20 (0.15–0.26)	14,157 (10,696–18,674)	26 (18–37)	0.05 (0.04–0.07)	3,710 (2,791–4,866)	14 (10–19)	0.03 (0.02–0.04)

Data are shown as the mean (95% UI). Total maternal deaths: maternal deaths + late maternal deaths. MMR: number of maternal deaths per 100,000 live births. LTR: the probability that a 15-year old female will eventually die from a maternal cause, assuming fertility and mortality risks do not change in the future. Estimated as the sum of age-specific maternal mortality rates from the ages of 15 to 49.

Instead, integrated strategies that include multiple interventions will probably be needed to substantially accelerate improvements in maternal mortality. Globally, we found that increasing facility births, improving the availability of clinical services at facilities and improving linkages to care (for example, recognition of complications and appropriate referrals and transportation) would yield large improvements, with a projected global MMR of 104 (95% UI = 87–121) in 2030 (Table 4). However, we found that even with this set of interventions, the global SDG target would not be met if improvements in quality of care are neglected. Instead, we found that efforts to improve quality of care in addition to facility-based and linkage interventions will be necessary to potentially achieve the SDG target, yielding a projected global MMR of 72 (95% UI = 58–87) in 2030, with large improvements in LMICs (Table 4).

Family planning interventions also offer the potential for meaningful improvements and can reduce deaths from abortion-related mortality and indirect causes (via reduced pregnancy exposure), which are largely unaffected by the other policy interventions that we modeled (Table 5). Although all strategies reduced the total number of maternal deaths compared to our baseline scenario of current trends, we found small relative increases for particular causes of death in some scenarios (Table 5). This finding reflects the fact that as more women survive pregnancy and labor, the number of deaths from subsequent risks may increase despite reductions in overall mortality, highlighting the need for a comprehensive view of maternal mortality that considers competing risks. For example, increasing the proportion of women who give birth in medical facilities is estimated to reduce total maternal deaths by nearly 30% but may lead to small increases in late and indirect maternal deaths as more women survive direct maternal complications and face these other risks (Table 5).

**Table 5:** Impact of interventions and strategies on projected global maternal deaths in 2030 according to cause

		Total	Abortive	Hypertensive	Hemorrhage	Sepsis	Obstructed labor	Other direct	Late	Indirect
Baseline	Deaths	327,438 (287,798–360,746)	26,606 (15,070–44,675)	34,970 (29,897–41,172)	34,218 (29,509–39,564)	33,636 (27,869–39,450)	29,111 (23,435–35,428)	56,276 (46,071–64,832)	43,414 (37,990–49,042)	69,207 (50,116–88,548)
**Family planning interventions**										
Contraception	Deaths	288,490 (249,440–320,663)	11,939 (7,515–17,956)	31,744 (27,456–37,279)	31,473 (26,469–36,782)	30,501 (24,785–36,654)	26,449 (21,265–32,290)	51,093 (41,830–60,093)	41,260 (35,904–47,362)	64,032 (46,148–82,960)
	Percentage reduction	11.9% (5.2–18.7%)	55.1% (31.5%–67.5%)	9.2% (−5.4%–22.3%)	8.0% (−7.7%–21.3%)	9.3% (−5.0%–22.6%)	9.1% (−7.7%–23.9%)	9.2% (−2.9%–20.8%)	5.0% (−11.7%–19.3%)	7.5% (−4.6%–18.5%)
Medical abortion	Deaths	313,408 (272,408–345,812)	11,955 (7556–18,271)	34,955 (29,810–40,550)	34,375 (29,458–39,510)	33,681 (27,730–39,912)	29,116 (23,551–34,739)	56,437 (46,643–65,377)	43,490 (38,033–49,355)	69,400 (49,297–88,384)
	Percentage reduction	4.3% (−1.0–9.6%)	55.1% (28.9–71.2%)	0.0% (−12.9–13.6%)	−0.5% (−13.6–11.0%)	−0.1% (−12.7–11.8%)	0.0% (−14.7–13.1%)	−0.3% (−11.4–8.5%)	−0.2% (−15.4–11.8%)	−0.3% (−11.7–10.6%)
**Community-based interventions**										
ANC	Deaths	319,671 (281,016–352,432)	24,144 (13,562–38,870)	33,477 (28,280–39,539)	32,915 (28,180–37,645)	31,109 (25,392–36,324)	29,085 (23,732–34,438)	56,292 (46,748–64,485)	43,203 (37,799–49,070)	69,447 (50,261–88,268)
	Percentage reduction	2.4% (−2.2–6.6%)	9.3% (−9.1–23.9%)	4.3% (−10.1–16.4%)	3.8% (−9.3–15.5%)	7.5% (−4.2–18.3%)	0.1% (−13.8–12.9%)	0.0% (−10.4–8.6%)	0.5% (−14.9–14.2%)	−0.3% (−12.1–10.1%)
SBA-home	Deaths	312,363 (276,042–347,181)	26,640 (15,059–45,409)	33,706 (29,063–39,406)	32,403 (27,771–37,600)	28,623 (23,487–33,295)	26,742 (21,351–32,456)	51,534 (42,894–59,564)	43,442 (37,836–49,815)	69,273 (49,493–87,683)
	Percentage reduction	4.6% (−0.4–8.9%)	−0.1% (−17.8–13.6%)	3.6% (−9.8–15.2%)	5.3% (−8.4–16.2%)	14.9% (2.3–26.2%)	8.1% (−5.3–19.7%)	8.4% (−2.2–16.9%)	−0.1% (−13.9–13.1%)	−0.1% (−12.2–10.4%)
**Facility-based interventions**										
Facility births	Deaths	232,820 (206,057–259,701)	26,428 (15,168–45,148)	24,910 (20,950–29,203)	16,216 (13,398–19,348)	11,033 (8,457–14,037)	10,740 (8,228–15,198)	30,590 (26,024–36,130)	43,529 (37,846–49,581)	69,375 (49,339–88,282)
	Percentage reduction	28.9% (21.5–35.1%)	0.7% (−19.0–16.5%)	28.8% (16.0–39.2%)	52.6% (40.5–62.3%)	67.2% (56.2–76.1%)	63.1% (48.4–72.6%)	45.6% (32.6–54.7%)	−0.3% (−15.8–12.4%)	−0.2% (−11.4–9.5%)
Non-EmOC services	Deaths	327,054 (287,164–361,654)	26,657 (14,942–45,286)	34,733 (29,629–41,135)	34,113 (29,425–39,512)	33,471 (27,458–39,076)	28,965 (23,602–34,906)	56,227 (46,216–64,943)	43,560 (37,842–49,694)	69,329 (49,545–88,641)
	Percentage reduction	0.1% (−4.2–4.1%)	−0.2% (−13.4–11.6%)	0.7% (−11.2–12.3%)	0.3% (−12.3–11.0%)	0.5% (−10.6–10.7%)	0.5% (−12.8–12.4%)	0.1% (−9.2–8.6%)	−0.3% (−13.3–11.2%)	−0.2% (−11.9–9.6%)
BEmOC services	Deaths	326,698 (287,057–360,323)	26,600 (14,820–45,613)	34,697 (29,709–40,737)	34,202 (29,652–39,538)	33,462 (27,779–39,191)	28,822 (23,053–34,741)	56,269 (46,707–64,938)	43,523 (37,852–48,951)	69,123 (49,683–87,628)
	Percentage reduction	0.2% (−4.2–4.3%)	0.0% (−16.6–14.2%)	0.8% (−12.1–11.8%)	0.0% (−13.2–11.1%)	0.5% (−12.1–11.8%)	1.0% (−13.6–12.7%)	0.0% (−9.9–8.4%)	−0.3% (−15.0–12.9%)	0.1% (−10.8–10.2%)
CEmOC services	Deaths	321,367 (279,767–356,722)	26,645 (14,872–45,141)	32,510 (27,527–37,832)	32,862 (27,673–38,096)	32,893 (27,017–38,637)	27,197 (21,121–32,841)	56,320 (46,205–65,108)	43,530 (37,712–50,082)	69,411 (49,629–88,417)
	Percentage reduction	1.9% (−2.9–6.1%)	−0.1% (−19.1–15.8%)	7.0% (−7.5–19.5%)	4.0% (−9.8–15.9%)	2.2% (−10.2–14.2%)	6.6% (−6.9–19.1%)	−0.1% (−11.4–9.6%)	−0.3% (−17.6–13.2%)	−0.3% (−11.4–9.5%)
**System-relevant interventions**										
Quality of care	Deaths	280,693 (239,251–314,390)	26,490 (15,063–44,596)	30,001 (24,415–36,189)	28,542 (23,791–33,880)	25,857 (19,335–31,752)	23,268 (17,887–28,322)	34,552 (23,602–42,984)	42,669 (37,485–48,987)	69,315 (50,148–87,613)
	Percentage reduction	14.3% (9.6–19.3%)	0.4% (−17.1–14.8%)	14.2% (2.5–25.4%)	16.6% (4.3–27.9%)	23.1% (10.8–35.4%)	20.1% (8.2–31.4%)	38.6% (28.3–51.7%)	1.7% (−12.0–13.5%)	−0.2% (−11.9–9.5%)
Referral	Deaths	324,183 (285,862–358,425)	26,596 (15,248–44,847)	34,514 (29,300–40,490)	34,030 (29,098–39,195)	33,141 (27,414–38,724)	28,694 (23,180–34,342)	54,578 (44,664–62,957)	43,421 (37,876–48,792)	69,208 (49,431–88,031)
	Percentage reduction	1.0% (−3.4–5.3%)	0.0% (−17.3–15.5%)	1.3% (−12.1–12.7%)	0.5% (−13.0–12.0%)	1.5% (−10.9–12.7%)	1.4% (−12.8–13.9%)	3.0% (−6.9–12.1%)	0.0% (−14.3–12.3%)	0.0% (−12.4–10.7%)
Transportation	Deaths	303,398 (269,404–336,013)	26,133 (14,597–43,921)	32,429 (27,596–38,000)	31,767 (27,501–36,278)	26,519 (21,643–31,111)	25,974 (20,727–31,453)	51,958 (43,539–59,722)	39,453 (34,485–44,815)	69,165 (49,462–87,385)
	Percentage reduction	7.3% (2.6–11.6%)	1.8% (−19.2–18.2%)	7.3% (−6.1–19.1%)	7.2% (−5.5–18.5%)	21.2% (9.6–32.0%)	10.8% (−3.8–22.4%)	7.7% (−2.6–17.1%)	9.1% (−5.6–21.8%)	0.1% (−11.1–9.9%)
Targeted transfers	Deaths	326,793 (288,463–360,511)	26,585 (14,944–43,516)	34,743 (29,655–40,658)	33,990 (29,095–39,158)	33,563 (27,776–39,057)	28,924 (23,312–34,591)	56,284 (46,256–64,735)	43,415 (37,964–49,397)	69,289 (49,422–87,804)
	Percentage reduction	0.2% (−4.2–4.1%)	0.1% (−15.3–13.6%)	0.6% (−10.7–11.4%)	0.7% (−11.3–12.0%)	0.2% (−11.2–9.9%)	0.6% (−12.9–12.8%)	0.0% (−8.7–8.1%)	0.0% (−15.0–13.0%)	−0.1% (−11.6–10.1%)
**Integrated strategies**										
Family planning	Deaths	284,617 (246,658–316,213)	7,852 (4,896–12,550)	31,816 (27,176–37,753)	31,495 (26,839–36,707)	30,495 (24,911–36,777)	26,492 (21,230–32,805)	51,160 (42,399–59,760)	41,443 (36,274–47,835)	63,865 (45,609–81,446)
	Percentage reduction	13.1% (5.8%–19.7%)	70.5% (49.4%–82.9%)	9.0% (−6.0%–22.8%)	8.0% (−5.8%–20.5%)	9.3% (−3.9%–22.4%)	9.0% (−7.6%–23.0%)	9.1% (−3.6%–20.6%)	4.5% (−11.5%–18.5%)	7.7% (−4.2%–18.4%)
Community + linkages	Deaths	270,519 (240,294–300,366)	23,533 (13,496–39,085)	28,880 (24,281–33,811)	28,152 (24,147–32,574)	20,029 (16,466–23,457)	21,851 (17,896–26,259)	39,965 (34,075–46,343)	38,839 (33,647–44,447)	69,271 (49,747–88,205)
	Percentage reduction	17.4% (12.4–22.1%)	11.6% (−7.6–27.3%)	17.4% (4.9–28.6%)	17.7% (5.1–28.4%)	40.5% (30.3–49.5%)	24.9% (11.9–35.8%)	29.0% (18.9–37.3%)	10.5% (−4.9–23.3%)	−0.1% (−12.0–10.2%)
Facilities + linkages	Deaths	216,808 (191,796–241,777)	25,914 (14,532–43,646)	21,182 (17,901–25,173)	13,794 (11,285–16,585)	9,662 (7,370–12,153)	7,604 (5,632–10,699)	29,818 (25,424–35,314)	39,605 (34,358–44,914)	69,228 (49,436–88,327)
	Percentage reduction	33.8% (26.8–39.8%)	2.6% (−16.8–17.7%)	39.4% (27.6–49.6%)	59.7% (50.1–67.9%)	71.3% (61.1–79.1%)	73.9% (63.7–81.0%)	47.0% (34.5–55.5%)	8.8% (−6.5–22.0%)	0.0% (−12.2–10.9%)
Facilities + linkages + quality	Deaths	160,441 (138,852–184,190)	25,721 (14,741–43,588)	14,057 (11,451–17,073)	7,185 (5,524–9,185)	1,560 (874–2,548)	1882 (1111–3211)	2655 (1856–3668)	37911 (32915–43291)	69470 (50379–88553)
	Percentage reduction	51.0% (45.1–56.1%)	3.3% (−15.7–18.7%)	59.8% (50.7–67.3%)	79.0% (72.6–84.1%)	95.4% (92.1–97.4%)	93.5% (89.5–96.3%)	95.3% (93.1–96.8%)	12.7% (−1.9–24.9%)	−0.4% (−11.5–10.4%)
Comprehensive	Deaths	130,682 (110,629–150,961)	6,933 (4,229–10,955)	12,630 (10,139–15,570)	6,502 (4,984–8,332)	1,297 (679–2,160)	1,676 (934–2,859)	2,275 (1,427–3,287)	35,391 (30,486–40,274)	63,979 (45,204–81,218)
	Percentage reduction	60.1% (54.9–65.0%)	73.9% (54.1–84.9%)	63.9% (54.3–72.1%)	81.0% (74.6–85.8%)	96.1% (93.2–98.0%)	94.2% (90.2–96.8%)	96.0% (93.9–97.5%)	18.5% (3.8–30.5%)	7.6% (−4.6–18.5%)

Estimated maternal deaths are reported as the mean and 95% UI. Percentage reduction is shown as the mean percentage reduction. A negative percentage reduction indicates a percentage increase, which could occur for competing mortality causes when other causes of death are reduced, for example, reducing abortive deaths results in more women facing the risk of other obstetric complications. Causes of maternal death: abortive (abortion, ectopic pregnancy, miscarriage), hypertensive disorders, hemorrhage, sepsis and other infections, obstructed labor, other direct, late maternal deaths, and indirect maternal deaths.

**Fig. 1:**
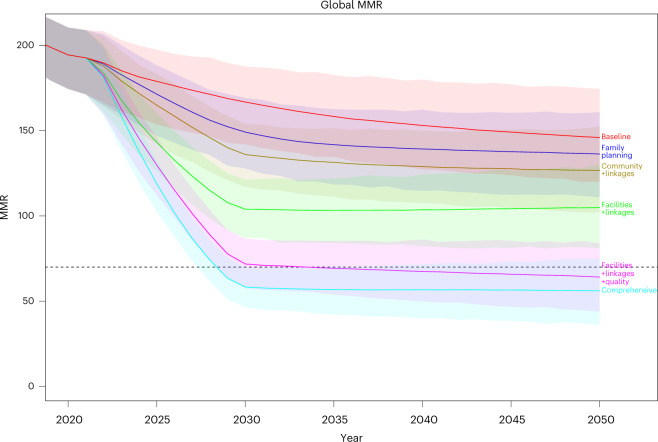
Projected global MMR according to an integrated strategy. The lines indicate the means, the shaded regions indicate the 95% UIs and the dashed line indicates the SDG target 3.1 of a global MMR of 70 by 2030.

Although we found that an integrated strategy including facility-based and system-relevant interventions (Table 3) could potentially achieve the SDG target in reducing maternal mortality, there is uncertainty around whether the goal could be achieved by 2030 (Fig. 1). Instead, due to the multifactorial nature and many causes of maternal death, a comprehensive integrated strategy including community-based and family planning interventions will probably be needed to achieve the global SDG target by 2030, with a projected global MMR of 58 (95% UI = 46–70).

### Region-specific impacts

**Extended Data Fig. 1:**
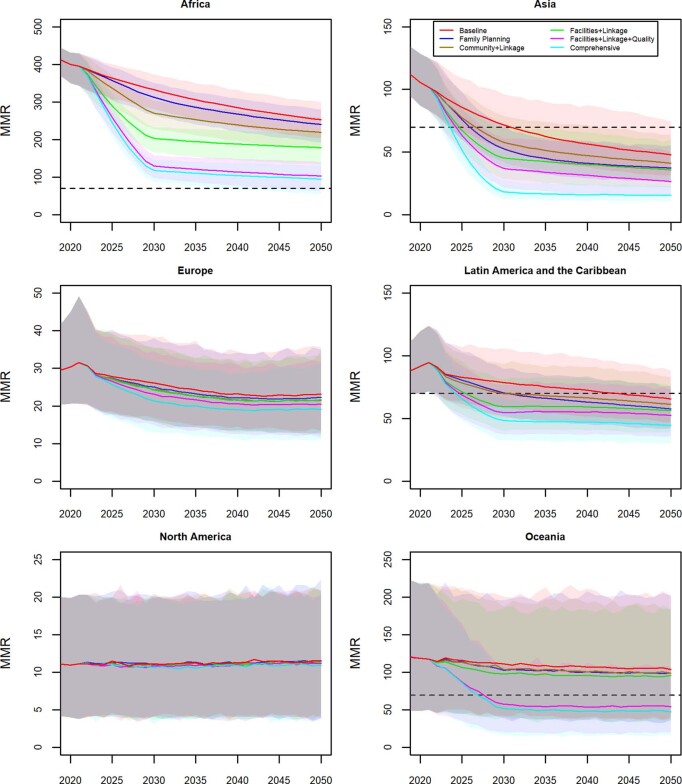
Projected MMR by geographic area and integrated strategy. Lines indicate means. Shaded regions indicate 95% UIs. Dashed line indicates SDG target 3.1 of a global MMR of 70 by 2030.

Overall, the projected impact of various strategies and comprehensive scale-up varied both across and within continents, depending on the baseline situation in each location, with high-income regions such as Europe and North America projected to have smaller reductions in maternal mortality due to better status quo indicators, while other regions would experience large benefits from a comprehensive scale-up, especially in Africa and Asia (Extended Data Fig. 1).

**Extended Data Fig. 2:**
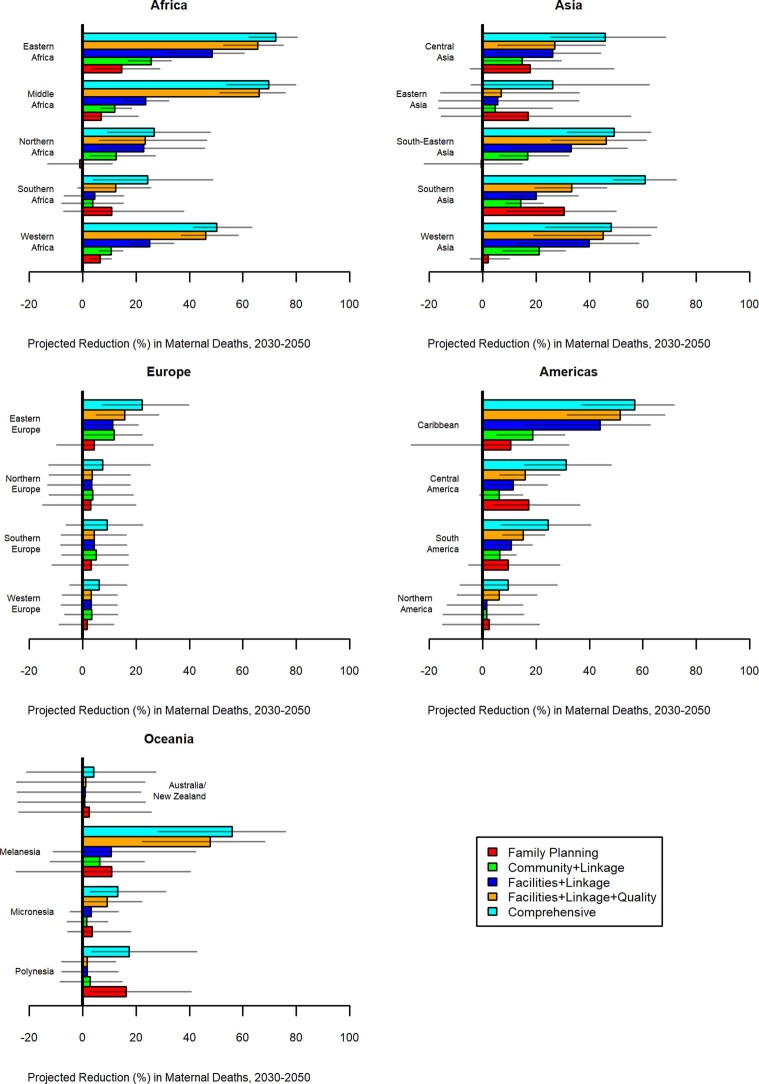
Projected Reduction in Maternal Deaths 2030–2050 by Region and Integrated Strategy. Reduction is mean percent reduction in projected maternal deaths in 2030–2050 (cumulative) compared to Baseline projections. Horizontal lines indicate 95% uncertainty intervals, calculated as the 2.5 and 97.5 percentiles of the simulation results.

Because the profile of causes of maternal death varies according to location^4^, the relative impact of different strategies also varies according to region (Extended Data Fig. 2), highlighting the need to consider the local context when designing and implementing policies. For example, family planning could have a large impact in areas of Asia and sub-Saharan Africa where fertility is still high. Even within regions there are differences in priority setting according to country. For example, in Southern Asia, family planning interventions are projected to have the largest impact among (noncomprehensive) integrated strategies in Afghanistan, where fertility is high. In contrast, in countries such as India, which have already experienced substantial declines in fertility, the incremental benefits of family planning alone are small, with far greater reductions in maternal mortality achieved by integrated strategies that focus on linkages to facilities and improved quality of care. Results for each modeled country and territory are available in the Appendix 1.

## Discussion

We found that an integrated strategy, including facility-based and system-relevant interventions, could substantially improve maternal health but it is unlikely to achieve the SDG global maternal mortality target by 2030 without also including community-based and family planning interventions as part of a comprehensive strategy. Among the interventions in such a strategy, we found that increasing the proportion of births that occur at health facilities is the single intervention with the largest impact on maternal mortality. However, while increasing facility delivery and linkages to care for complications from the community are necessary to substantially improve maternal health, these policies alone may not be sufficient if quality of care is not also improved.

This finding echoes previous evaluations of real-world interventions. For example, Janani Suraksha Yojana, a conditional cash-transfer program to incentivize facility delivery in India, succeeded in substantially increasing facility births but had no significant effect on mortality due to lack of focus on the quality of facilities^30,31^. Similarly, the Better Birth trial succeed in improving the adherence of SBAs to essential birth practices but had no significant impact on health outcomes^32^. These findings highlight the necessity of considering other contextual factors related to health system quality.

The complex interdependencies of service delivery mean that a narrow focus on one aspect of the care continuum is unlikely to substantially improve outcomes. Identifying and estimating the relative contribution of these factors in a structural model can help inform policymakers on the likely impact of such interventions before they are implemented, including the impact of competing risks for other health outcomes.

At the global level, these results can reveal general insights that can be broadly applied, such as that individual policies generally offer modest incremental gains and are less effective than integrated strategies, especially those that include enhanced linkages to care along the referral pathway, for example, recognition and referral, and timely transport to health facilities. These insights echo previous findings and our general estimates are similar to previous model-based studies. For example, a previous study that focused on India estimated that integrated efforts to improve family planning, access to safe abortion and scale up high-quality maternal health services could reduce direct maternal deaths by more than 75%^24^. The Guttmacher Institute Adding It Up report estimated that if all women had access to modern contraceptives and all pregnant women received quality care, maternal deaths would drop by 62% in LMICs^33^, which is very similar to our comprehensive strategy that yields a 60.1% (95% UI = 54.9–65.0%) decrease globally. The report also estimated that meeting all needs for pregnancy-related care, with no change in levels of contraceptive care, would yield a 50% reduction in maternal deaths in LMICs^33^, which is similar to our strategy including facility-based, system-relevant and quality-of-care interventions that yields an estimated 51.0% (95% UI = 45.1%–56.1%) global reduction. These findings are also similar to a previous analysis using the LiST model, which estimated that 51% of maternal deaths could be averted in 75 high-burden countries by achieving high coverage of ANC and care during labor and childbirth^34^.

Our model findings also align with real-world evidence, such as a program in Dar es Salaam, Tanzania that implemented multiple evidence-based health system strengthening interventions addressing the continuum of care and yielded a sixfold improvement in quality of care and a 47% reduction in maternal mortality^35^. Such programs show that implementing integrated strategies and achieving substantial reductions in maternal mortality can be feasible even in resource-constrained settings.

Although our general findings are similar to previous estimates, a high-level global view can also obscure differences and may not provide adequate guidance for policymakers in different contexts. Instead, our regional and country-specific estimates of the impact of various policies are probably more informative for decision-making. These results also highlight the importance of considering a dashboard of multiple indicators when setting targets and assessing progress (in addition to MMR), which reflect differences in baseline indicators and health system priorities that vary according to context. For example, family planning interventions that lower exposure to pregnancy but have smaller effects on obstetrical risk may have larger impacts on indicators such as lifetime risk (LTR) of maternal death and total maternal deaths, while having a smaller impact on MMR. Multiple indicators are therefore needed to monitor progress both globally and especially at the country-level where local contexts may vary substantially. Our country-level comparative effectiveness estimates can help provide guidance on which types of policies local decision-makers should prioritize as interventions are scaled up.

Although we provide estimates of such policy impacts for 200 countries and territories, the lack of data for many variables of interest for some locations, such as the distribution of emergency obstetric care (EmOC) facility types where women give birth, mean that we had to make certain assumptions when developing the model. We used a hierarchical modeling approach to regularize estimates that were available and impute parameter values for countries and territories for which we had no data^4^. We account for uncertainty around all model parameters and report uncertainty intervals for all model outcomes but recognize that as these are conditional on the model structure, there are probably other sources of uncertainty, for example, structural assumptions, which are not reflected in our reported measures of uncertainty. For our projections, we also assumed that current trends will continue (for each model parameter that incorporates secular trends) for our baseline comparator strategy. Although our estimates can provide insight into the comparative effectiveness of policies to improve maternal health, we recognize the need for decision-makers to also consider the costs of implementing such policies. Indeed, in previous work we identified more and less efficient ways of scaling up interventions in India^25^. Although we do not currently include costs in the Global Maternal Health (GMatH) model, an advantage of our approach is that we can assess the construction of such strategies in more realistic, nuanced ways; in future research, we plan to extend our approach to consider costs.

Examining the cost-effectiveness and budget impact of interventions was outside the scope of this study but such analyses can also provide information regarding the feasibility of different policies. We set minimum coverage targets for this analysis informed by the mean levels of high-income countries, instead of modeling convergence with the best-performing countries, to help address concerns regarding feasibility. However, substantial investments will still probably be required to achieve these targets. This point is especially important because we modeled the incremental impact of intervention scale-up in addition to baseline trends, which we assume will continue, but which cannot be taken for granted because progress to date has been due to sustained efforts over long periods of time, which will not only have to be continued but expanded on.

This study has several limitations. Even under comprehensive scale-up, we found that there would still exist large differences in maternal mortality between low-income and high-income countries. This is probably due to the role of indirect maternal deaths, which are heterogeneous across countries and which we assumed were unaffected by our modeled clinical and health system interventions. We also assumed that trends in demographic composition, typically a lower education level and rural location in low-income countries, would continue on projected trends and not be affected by policy interventions. Our assumption of lack of direct impact on late and indirect maternal deaths of the modeled policies means that our estimates may be conservative. Additional interventions to improve women’s education and address indirect causes of maternal death, for example, malaria and HIV, are therefore probably needed in addition to interventions that address direct causes of maternal deaths.

Despite these limitations, we find that our structural modeling approach has high predictive accuracy^4^; because it models the underlying causal relationships between factors that impact various aspects of maternal health, it allows for more realistic counterfactual estimates to be made. In addition to insights from our global estimates, we find that the relative priority of specific interventions varies according to region. Our regional and country-level comparative effectiveness estimates can therefore help guide planning and priority setting in various contexts to accelerate improvements in maternal health.

## Methods

### Simulation model overview

We developed the GMatH microsimulation model (previously described elsewhere)^4^ to simulate the reproductive histories of individual women in 200 countries and territories, accounting for heterogeneity in education and urban or rural location, family planning preferences and history of maternal complications (see http://gmath-model.org/ for online model documentation). The model progresses in monthly cycles and follows an open population, that is, with new female infants entering each cycle, allowing population-level indicators to be estimated by calendar year. The model simulates pregnancy based on age, breastfeeding status and contraceptive use, which is informed by women’s individual fertility preferences and whether their need for contraception is met. Pregnant women may experience ectopic pregnancy or miscarriage, and unintended pregnancies also face a risk of induced abortion, a proportion of which may be ‘unsafe’ (for example, when conducted by untrained personnel) and associated with higher morbidity and mortality. Complications associated with pregnancy and childbirth are modeled based on individual-level risk factors, for example, anemia, with incidence and case fatality rates impacted by various health system factors, such as ANC visits, appropriate referral and transportation to facilities (which we assume are implicitly impacted by factors such as cost of care), availability of specific clinical interventions, level of care available and quality of care.

We included a parameter for ‘quality of care’ to account for health system-level and facility-level factors not explicitly included in the model. Quality of care has been defined as the ‘degree to which health services for individuals and populations increase the likelihood of desired health outcomes and are consistent with current professional knowledge’^36^. Although it is a broad concept, the Institute of Medicine has described quality of care as having six key features: care that is safe; effective; patient-centered; efficient; timely; and equitable. Although all these features are important, there are often particularly substantial deficiencies regarding patient safety, for example, iatrogenic harm; effectiveness, for example, providers may fail to provide the appropriate treatment even if it is available; and patient-centeredness, for example, patients may opt out of or not adhere to treatment if they lack confidence in the health system^31^. We therefore included this parameter in the model to account for residual differences in maternal mortality not explained by differences in the incidence or severity of obstetric complications and availability or efficacy of clinical interventions, such as improved professionalization of health workers. Thus, this parameter is focused on the quality of clinical care received at health facilities after accounting for other factors both within and outside the health system.

In addition to death from pregnancy-related complications, that is, direct maternal deaths, women also face risks of indirect maternal deaths and age-specific competing mortality from other causes.

The model was calibrated to empirical data on a range of maternal health-related indicators, accounting for underreporting of maternal deaths, and had high predictive accuracy for a test set of external data not used to fit the model, with coverage probabilities, that is, the proportion of times the empirical point estimate fell within the model’s 95% UI of 96.0% for maternal mortality indicators and a mean error of 2.6 deaths (s.e. = 8.9) for total maternal deaths^4^.

### Interventions and strategies

Using the model, we compared the effectiveness of a range of different policies to reduce maternal mortality, ranging from individual interventions to integrated strategies. We compared the impact of each policy to a baseline scenario of current trends, that is, no new intervention of scale-up but with current trends due to previous and existing efforts assumed to continue, and modeled the implementation of each policy by scaling up the relevant parameters between 2022 and 2030 to achieve minimum coverage targets informed by the mean level of high-income countries in 2022, assuming a linear scale-up over time. Following previously used typologies for maternal health interventions^16–19,24^, we categorized interventions as family planning, community-based, facility-based and system-relevant interventions (Table 2).

Family planning interventions include reducing unmet need for contraception and reducing the proportion of induced abortions that are unsafe, allowing women more control to achieve their fertility preferences, which we assume are unaffected by these interventions. Community-based interventions include increased ANC visits to improve health status during pregnancy, for example, anemia, and improve recognition of complication danger signs, as well as increased coverage of home births attended by an SBA, who can provide basic interventions and refer complications to facilities for emergency care.

Facility-based interventions include increasing the number of women who give birth in a health facility (regardless of facility type), and improving the availability of clinical services at facilities. Three levels of facilities were modeled based on the level of EmOC they are supposed to be able to provide, based on World Health Organization guidelines: non-EmOC (no EmOC) facility; BEmOC facility; and CEmOC facility^37^. The delivery site for each woman was modeled based on urban or rural location and educational level, thus accounting for differential EmOC access both across and within countries. Although facilities that are designated as CEmOC are theoretically supposed to provide all required obstetric clinical services, for example, surgery, in practice facilities may only offer a subset of services that they would normally be expected to provide due to equipment, drug and personnel shortages, as well as broader infrastructure factors, such as lack of stable power supply^38,39^. Therefore, the GMatH model simulates the availability of specific clinical services at each facility type within each country, assuming that although CEmOC facilities in a given context may not offer all necessary services, the availability of services is higher than at BEmOC or non-EmOC facilities in the same context^4^.

As a conservative assumption, we assumed that increasing EmOC services alone has no clinical impact on late maternal deaths or indirect maternal deaths, for example, malaria or HIV-related deaths exacerbated by pregnancy, as these would probably require other targeted, that is, non-obstetric interventions, or on other direct maternal deaths, which are treated mainly with supportive care that we assumed could be impacted by improving quality of care.

System-relevant policies include improving the quality of care at facilities and improving linkages to care along the referral pathway, such as recognition and referral of complications, timely transportation and transfers to facilities with the appropriate level of care to manage the complication at hand. We also modeled combinations of these individual policies to evaluate the impact of integrated policy strategies (Table 3).

### Statistical analysis

We compared the impact of the policy interventions on projected maternal indicators, including total maternal deaths, MMR and LTR of maternal deaths. We simulated 1,000 iterations of each policy, sampling from the 100 best-fitting parameter sets in each iteration to account for both first-order, that is, individual-level, stochastic, and second-order, that is, parameter uncertainty. For all model outcomes, we report the mean and 95% UIs, calculated as the 2.5 and 97.5 percentiles of the simulation results. The GMatH model was developed in Java v1.8.0.

### Ethics and inclusion statement

All data for this study, including from LMICs, were obtained from publicly available sources. One colleague (B.S.D.) is from an LMIC and the corresponding author (Z.J.W.) is originally from an LMIC and is now based in a high-income country. We fully endorse the *Nature* Portfolio guidance on LMIC authorship and inclusion. Because this work builds on previous modeling work, authorship was based, in part, on previous participation and collaboration. However, we are strongly committed to collaboration with researchers from LMICs in future work, especially for analyses focused on specific contexts or countries.

This research is locally relevant to all countries included as we report findings by country, providing local policymakers with important data on the impact of maternal health interventions.

Because our modeling approach used only publicly available data and published data from the medical literature for each country, ethics review was not required. The data collection and analysis techniques used raised no risks pertaining to stigmatization, incrimination, discrimination, animal welfare, the environment, health, safety, security or other personal risks. No biological materials, cultural artifacts or associated traditional knowledge has been transferred out of any country. In preparing the manuscript, the authors reviewed relevant studies from all countries for which data were available.

### Reporting summary

Further information on research design is available in the Nature Portfolio Reporting Summary linked to this article.

## Online content

Any methods, additional references, Nature Portfolio reporting summaries, source data, extended data, supplementary information, acknowledgements, peer review information; details of author contributions and competing interests; and statements of data and code availability are available at 10.1038/s41591-023-02311-w.

## Supplementary information

Supplementary Information Supplementary Appendix 1 (country results).

Reporting Summary

## Data Availability

The simulation results are available in a public data repository at 10.7910/DVN/4F56ZB. We have also provided documentation for all model parameters, including data sources, assumptions and model implementation details online (http://gmath-model.org/).

## Code availability

No software was used for any primary data collection in this study. The GMatH simulation model was developed in Java v.1.8.0 and statistical analyses were performed in R v.3.6.1. The pseudocode describing the simulation algorithm is available at http://gmath-model.org/1_7_Pseudocode.html. Requests for more code details should be addressed to Z.J.W.
