# Supplementary material for: A simulation-based comparative effectiveness analysis of policies to improve global maternal health outcomes
Source: Nat Med. 2023 Apr 20;29(5):1262–72. doi: 10.1038/s41591-023-02311-w (PMC10202805; doi:10.1038/s41591-023-02311-w)

nature medicine

Article

https://doi.org/10.1038/s41591-023-02311-w

A simulation-based comparative effectiveness analysis of policies to improve global maternal health outcomes

In the format provided by the authors and unedited

Contents

Country Results **5**
Afghanistan . . . 6
Albania . . . 7
Algeria . . . 8
Andorra . . . 9
Angola . . . 10
Antigua and Barbuda . . . 11
Argentina . . . 12
Armenia . . . 13
Australia . . . 14
Austria . . . 15
Azerbaijan . . . 16
Bahamas . . . 17
Bahrain . . . 18
Bangladesh . . . 19
Barbados . . . 20
Belarus . . . 21
Belgium . . . 22
Belize . . . 23
Benin . . . 24
Bermuda . . . 25
Bhutan . . . 26
Bolivia (Plurinational State of) . . . 27
Bosnia and Herzegovina . . . 28
Botswana . . . 29
Brazil . . . 30
Brunei Darussalam . . . 31
Bulgaria . . . 32
Burkina Faso . . . 33
Burundi . . . 34
Cabo Verde . . . 35
Cambodia . . . 36
Cameroon . . . 37
Canada . . . 38
Cayman Islands . . . 39
Central African Republic . . . 40
Chad . . . 41
Chile . . . 42
China . . . 43
Colombia . . . 44
Comoros . . . 45
Congo . . . 46
Costa Rica . . . 47
Côte d'Ivoire . . . 48
Croatia . . . 49
Cuba . . . 50
Cyprus . . . 51
Czechia . . . 52
Dem. People's Republic of Korea . . . 53
Democratic Republic of the Congo . . . 54
Denmark . . . 55
Djibouti . . . 56

Dominica . . . 57
Dominican Republic . . . 58
Ecuador . . . 59
Egypt . . . 60
El Salvador . . . 61
Equatorial Guinea . . . 62
Eritrea . . . 63
Estonia . . . 64
Eswatini . . . 65
Ethiopia . . . 66
Faroe Islands . . . 67
Fiji . . . 68
Finland . . . 69
France . . . 70
Gabon . . . 71
Gambia . . . 72
Georgia . . . 73
Germany . . . 74
Ghana . . . 75
Greece . . . 76
Greenland . . . 77
Grenada . . . 78
Guatemala . . . 79
Guinea . . . 80
Guinea-Bissau . . . 81
Guyana . . . 82
Haiti . . . 83
Honduras . . . 84
Hungary . . . 85
Iceland . . . 86
India . . . 87
Indonesia . . . 88
Iran (Islamic Republic of) . . . 89
Iraq . . . 90
Ireland . . . 91
Israel . . . 92
Italy . . . 93
Jamaica . . . 94
Japan . . . 95
Jordan . . . 96
Kazakhstan . . . 97
Kenya . . . 98
Kiribati . . . 99
Kuwait . . . 100
Kyrgyzstan . . . 101
Lao People's Democratic Republic . . . 102
Latvia . . . 103
Lebanon . . . 104
Lesotho . . . 105
Liberia . . . 106
Libya . . . 107
Liechtenstein . . . 108
Lithuania . . . 109
Luxembourg . . . 110

Madagascar . . . 111
Malawi . . . 112
Malaysia . . . 113
Maldives . . . 114
Mali . . . 115
Malta . . . 116
Marshall Islands . . . 117
Mauritania . . . 118
Mauritius . . . 119
Mexico . . . 120
Micronesia (Fed. States of) . . . 121
Monaco . . . 122
Mongolia . . . 123
Montenegro . . . 124
Morocco . . . 125
Mozambique . . . 126
Myanmar . . . 127
Namibia . . . 128
Nauru . . . 129
Nepal . . . 130
Netherlands . . . 131
New Zealand . . . 132
Nicaragua . . . 133
Niger . . . 134
Nigeria . . . 135
North Macedonia . . . 136
Norway . . . 137
Oman . . . 138
Pakistan . . . 139
Palau . . . 140
Panama . . . 141
Papua New Guinea . . . 142
Paraguay . . . 143
Peru . . . 144
Philippines . . . 145
Poland . . . 146
Portugal . . . 147
Puerto Rico . . . 148
Qatar . . . 149
Republic of Korea . . . 150
Republic of Moldova . . . 151
Romania . . . 152
Russian Federation . . . 153
Rwanda . . . 154
Saint Kitts and Nevis . . . 155
Saint Lucia . . . 156
Saint Vincent and the Grenadines . . . 157
Samoa . . . 158
San Marino . . . 159
Sao Tome and Principe . . . 160
Saudi Arabia . . . 161
Senegal . . . 162
Serbia . . . 163
Seychelles . . . 164

Sierra Leone . . . 165
Singapore . . . 166
Slovakia . . . 167
Slovenia . . . 168
Solomon Islands . . . 169
Somalia . . . 170
South Africa . . . 171
South Sudan . . . 172
Spain . . . 173
Sri Lanka . . . 174
State of Palestine . . . 175
Sudan . . . 176
Suriname . . . 177
Sweden . . . 178
Switzerland . . . 179
Syrian Arab Republic . . . 180
Taiwan . . . 181
Tajikistan . . . 182
Thailand . . . 183
Timor-Leste . . . 184
Togo . . . 185
Tonga . . . 186
Trinidad and Tobago . . . 187
Tunisia . . . 188
Türkiye . . . 189
Turkmenistan . . . 190
Tuvalu . . . 191
Uganda . . . 192
Ukraine . . . 193
United Arab Emirates . . . 194
United Kingdom . . . 195
United Republic of Tanzania . . . 196
United States of America . . . 197
Uruguay . . . 198
Uzbekistan . . . 199
Vanuatu . . . 200
Venezuela (Bolivarian Republic of) . . . 201
Viet Nam . . . 202
Yemen . . . 203
Zambia . . . 204
Zimbabwe . . . 205

Country Results

We report the following maternal health indicators for each country by policy scenario:

- Maternal Deaths: Number of maternal deaths per year, including late maternal deaths
- Maternal Mortality Ratio (MMR): Number of maternal deaths per 100,000 live births
- Lifetime Risk of Maternal Death (LTR): The probability that a 15-year old female will eventually die from a maternal cause, assuming fertility and mortality risks do not change in the future. Estimated as the sum of age-specific maternal mortality rates from ages 15 to 49

Afghanistan

ISO Code	Region	Area	Income Group
AFG	Southern Asia	Asia	Low income

Projected Maternal Indicators in 2030 by Scenario

Scenario	Maternal Deaths	MMR	LTR
**Baseline**	16196 (7207-29716)	1035 (396-2579)	3.99 (1.54-7.7)
**Family Planning Interventions**			
Contraception	5687 (3393-9383)	439 (229-777)	1.23 (0.67-2.13)
Medical abortion	5981 (3479-10111)	329 (191-565)	1.21 (0.62-2.02)
**Community-Based Interventions**			
ANC	14526 (6494-25870)	921 (353-2105)	3.55 (1.33-6.57)
SBA	16140 (7248-29983)	1033 (387-2738)	3.98 (1.52-7.72)
**Facility-Based Interventions**			
Facility births	14538 (6351-28800)	929 (332-2625)	3.62 (1.39-7.42)
nonEmOC services	16172 (7163-29764)	1033 (386-2625)	3.99 (1.48-7.75)
bEmOC services	16256 (7355-30035)	1038 (390-2606)	4 (1.58-7.7)
cEmOC services	16123 (7025-30655)	1033 (373-2590)	3.98 (1.49-7.79)
**System-Relevant Interventions**			
Quality of care	14852 (5756-29263)	949 (287-2556)	3.69 (1.2-7.38)
Referral	16170 (7064-29989)	1033 (373-2592)	3.99 (1.54-7.57)
Transport	15831 (7109-29714)	1013 (371-2616)	3.91 (1.57-7.55)
Targeted transfers	16189 (6973-28878)	1035 (378-2609)	4 (1.52-7.43)
**Integrated Strategies**			
Family Planning	3433 (1940-5421)	239 (131-388)	0.61 (0.3-1.04)
Community + Linkages	13833 (6322-25573)	880 (336-2180)	3.4 (1.33-6.65)
Facilities + Linkages	14349 (6190-28405)	917 (327-2512)	3.58 (1.32-7.12)
Facilities + Linkages + Quality	12607 (4833-27118)	807 (220-2367)	3.2 (1.03-7.04)
Comprehensive	1144 (478-1944)	49 (12-104)	0.13 (0.03-0.27)

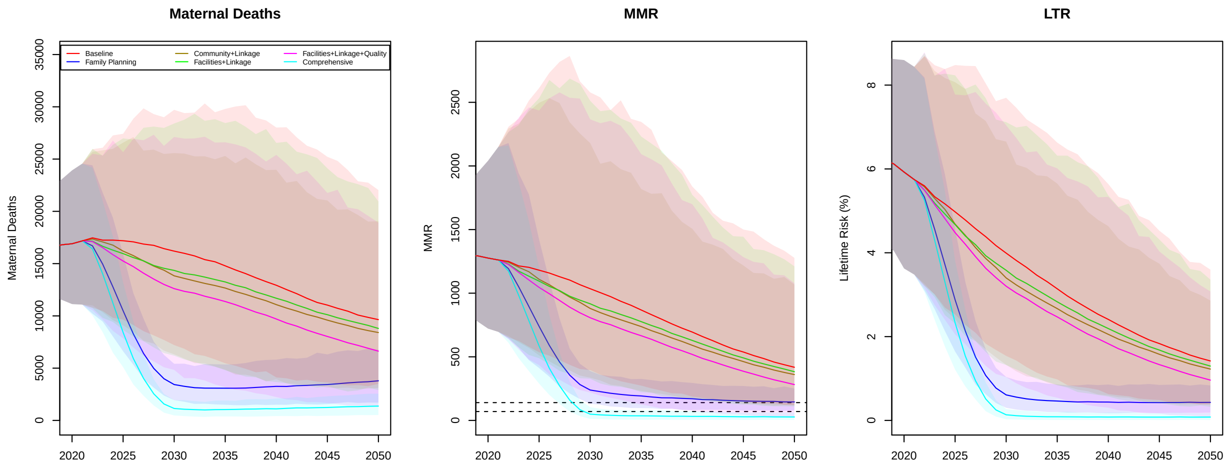

Albania

ISO Code	Region	Area	Income Group
ALB	Southern Europe	Europe	Upper middle income

Projected Maternal Indicators in 2030 by Scenario

Scenario	Maternal Deaths	MMR	LTR
**Baseline**	17 (0-86)	43 (0-320)	0.05 (0-0.39)
**Family Planning Interventions**			
Contraception	15 (0-98)	36 (0-297)	0.04 (0-0.4)
Medical abortion	18 (0-99)	48 (0-337)	0.06 (0-0.45)
**Community-Based Interventions**			
ANC	18 (0-95)	47 (0-330)	0.06 (0-0.43)
SBA	17 (0-89)	42 (0-320)	0.05 (0-0.39)
**Facility-Based Interventions**			
Facility births	17 (0-88)	47 (0-348)	0.06 (0-0.41)
nonEmOC services	17 (0-86)	44 (0-320)	0.05 (0-0.39)
bEmOC services	17 (0-86)	44 (0-320)	0.05 (0-0.39)
cEmOC services	18 (0-92)	43 (0-321)	0.05 (0-0.41)
**System-Relevant Interventions**			
Quality of care	17 (0-86)	44 (0-320)	0.05 (0-0.39)
Referral	17 (0-88)	45 (0-320)	0.05 (0-0.4)
Transport	17 (0-97)	44 (0-319)	0.05 (0-0.42)
Targeted transfers	17 (0-84)	45 (0-318)	0.05 (0-0.41)
**Integrated Strategies**			
Family Planning	15 (0-96)	35 (0-297)	0.04 (0-0.41)
Community + Linkages	19 (0-112)	52 (0-368)	0.06 (0-0.45)
Facilities + Linkages	17 (0-99)	45 (0-320)	0.06 (0-0.43)
Facilities + Linkages + Quality	17 (0-100)	46 (0-326)	0.06 (0-0.43)
Comprehensive	14 (0-100)	32 (0-271)	0.04 (0-0.39)

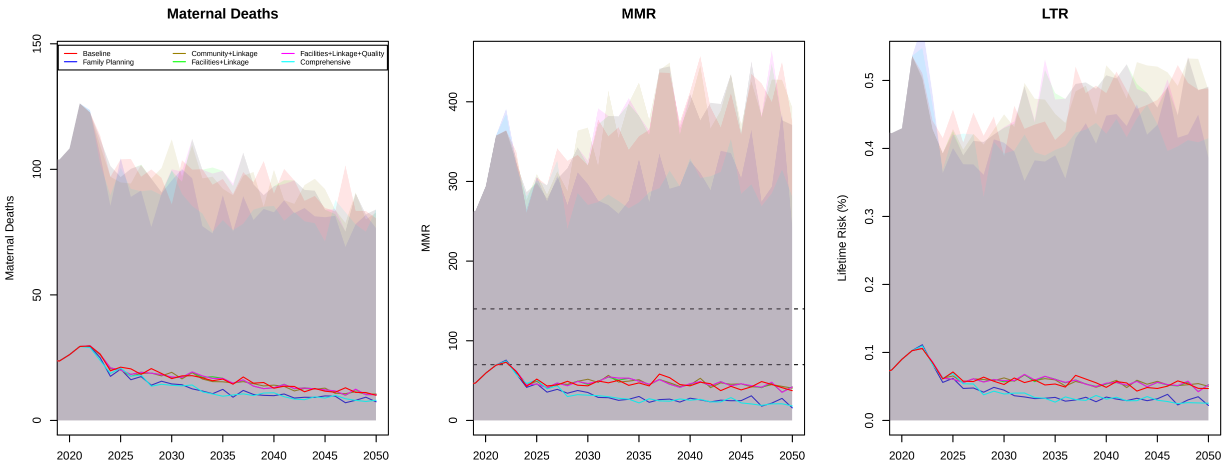

Algeria

ISO Code	Region	Area	Income Group
DZA	Northern Africa	Africa	Upper middle income

Projected Maternal Indicators in 2030 by Scenario

Scenario	Maternal Deaths	MMR	LTR
**Baseline**	449 (0-1199)	21 (0-64)	0.08 (0-0.27)
**Family Planning Interventions**			
Contraception	442 (0-1192)	20 (0-64)	0.08 (0-0.27)
Medical abortion	454 (0-1213)	21 (0-67)	0.08 (0-0.29)
**Community-Based Interventions**			
ANC	451 (0-1289)	21 (0-66)	0.08 (0-0.29)
SBA	444 (0-1234)	21 (0-65)	0.08 (0-0.26)
**Facility-Based Interventions**			
Facility births	441 (0-1195)	20 (0-64)	0.08 (0-0.26)
nonEmOC services	446 (0-1195)	21 (0-64)	0.08 (0-0.27)
bEmOC services	456 (0-1192)	21 (0-62)	0.08 (0-0.25)
cEmOC services	444 (0-1131)	21 (0-61)	0.08 (0-0.25)
**System-Relevant Interventions**			
Quality of care	454 (0-1195)	21 (0-64)	0.08 (0-0.27)
Referral	454 (26-1198)	21 (0-64)	0.08 (0-0.27)
Transport	460 (0-1172)	22 (0-64)	0.08 (0-0.25)
Targeted transfers	447 (0-1174)	21 (0-62)	0.08 (0-0.25)
**Integrated Strategies**			
Family Planning	447 (0-1192)	21 (0-65)	0.08 (0-0.26)
Community + Linkages	428 (0-1166)	20 (0-61)	0.07 (0-0.24)
Facilities + Linkages	440 (0-1206)	20 (0-60)	0.08 (0-0.26)
Facilities + Linkages + Quality	429 (0-1206)	20 (0-61)	0.08 (0-0.26)
Comprehensive	443 (0-1143)	21 (0-64)	0.08 (0-0.28)

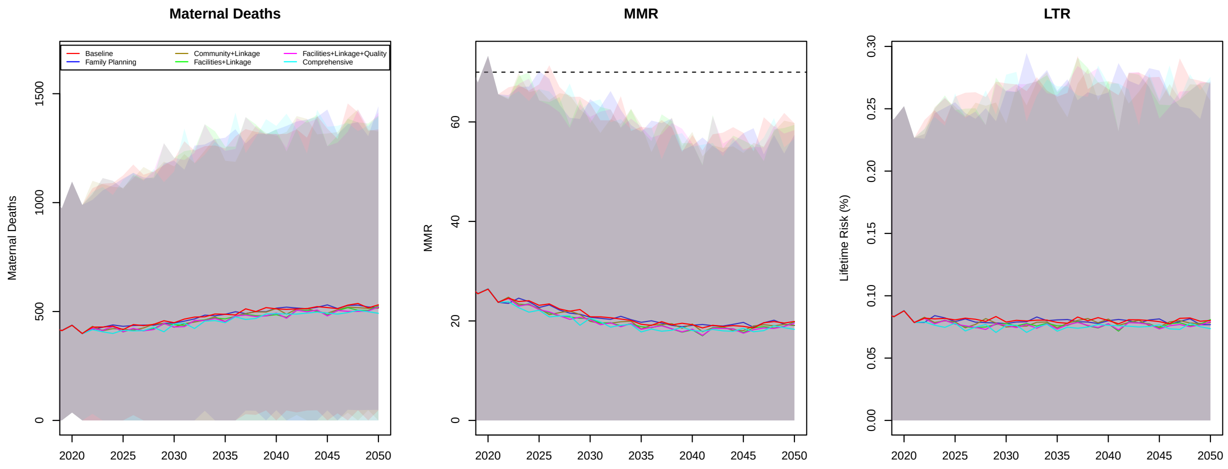

Andorra

ISO Code	Region	Area	Income Group
AND	Southern Europe	Europe	High income

Projected Maternal Indicators in 2030 by Scenario

Scenario	Maternal Deaths	MMR	LTR
**Baseline**	0 (0-2)	10 (0-221)	0.01 (0-0.28)
**Family Planning Interventions**			
Contraception	0 (0-2)	12 (0-239)	0.01 (0-0.28)
Medical abortion	0 (0-2)	10 (0-221)	0.01 (0-0.28)
**Community-Based Interventions**			
ANC	0 (0-2)	10 (0-221)	0.01 (0-0.28)
SBA	0 (0-2)	10 (0-213)	0.01 (0-0.29)
**Facility-Based Interventions**			
Facility births	0 (0-2)	11 (0-229)	0.01 (0-0.28)
nonEmOC services	0 (0-2)	10 (0-221)	0.01 (0-0.28)
bEmOC services	0 (0-2)	10 (0-221)	0.01 (0-0.28)
cEmOC services	0 (0-2)	10 (0-221)	0.01 (0-0.28)
**System-Relevant Interventions**			
Quality of care	0 (0-2)	10 (0-221)	0.01 (0-0.28)
Referral	0 (0-2)	10 (0-221)	0.01 (0-0.28)
Transport	0 (0-2)	9 (0-213)	0.01 (0-0.28)
Targeted transfers	0 (0-2)	10 (0-221)	0.01 (0-0.28)
**Integrated Strategies**			
Family Planning	0 (0-2)	11 (0-235)	0.01 (0-0.28)
Community + Linkages	0 (0-2)	10 (0-213)	0.01 (0-0.31)
Facilities + Linkages	0 (0-2)	13 (0-235)	0.02 (0-0.32)
Facilities + Linkages + Quality	0 (0-2)	13 (0-235)	0.02 (0-0.32)
Comprehensive	0 (0-2)	9 (0-217)	0.01 (0-0.28)

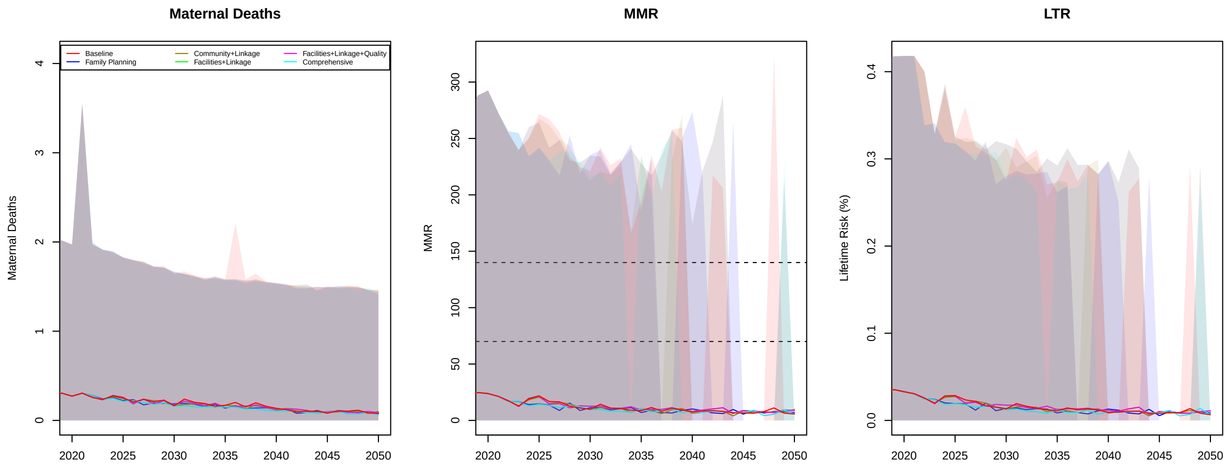

Angola

ISO Code	Region	Area	Income Group
AGO	Middle Africa	Africa	Lower middle income

Projected Maternal Indicators in 2030 by Scenario

Scenario	Maternal Deaths	MMR	LTR
**Baseline**	5000 (3000-7507)	200 (120-305)	1.14 (0.67-1.76)
**Family Planning Interventions**			
Contraception	4455 (2625-6830)	186 (108-294)	0.98 (0.53-1.63)
Medical abortion	4974 (3068-7472)	199 (118-305)	1.14 (0.65-1.77)
**Community-Based Interventions**			
ANC	4841 (3005-7301)	193 (115-308)	1.11 (0.64-1.7)
SBA	4874 (2915-7248)	195 (116-299)	1.12 (0.64-1.7)
**Facility-Based Interventions**			
Facility births	2659 (1427-4274)	96 (41-163)	0.51 (0.2-0.9)
nonEmOC services	4952 (3033-7473)	198 (117-310)	1.14 (0.67-1.81)
bEmOC services	4967 (3064-7319)	198 (123-303)	1.14 (0.67-1.76)
cEmOC services	4970 (2959-7488)	198 (111-307)	1.13 (0.62-1.73)
**System-Relevant Interventions**			
Quality of care	4156 (2484-6585)	162 (93-267)	0.95 (0.53-1.54)
Referral	4834 (2911-7280)	193 (113-309)	1.1 (0.62-1.72)
Transport	4467 (2581-6850)	179 (103-280)	1.01 (0.54-1.61)
Targeted transfers	4985 (2947-7474)	200 (113-308)	1.14 (0.66-1.77)
**Integrated Strategies**			
Family Planning	4434 (2583-6737)	184 (106-285)	0.96 (0.51-1.52)
Community + Linkages	3890 (2265-5874)	152 (86-241)	0.85 (0.46-1.38)
Facilities + Linkages	2492 (1131-4205)	90 (37-157)	0.47 (0.15-0.89)
Facilities + Linkages + Quality	1402 (550-2567)	43 (10-91)	0.22 (0.04-0.54)
Comprehensive	1252 (452-2339)	39 (11-80)	0.18 (0.03-0.47)

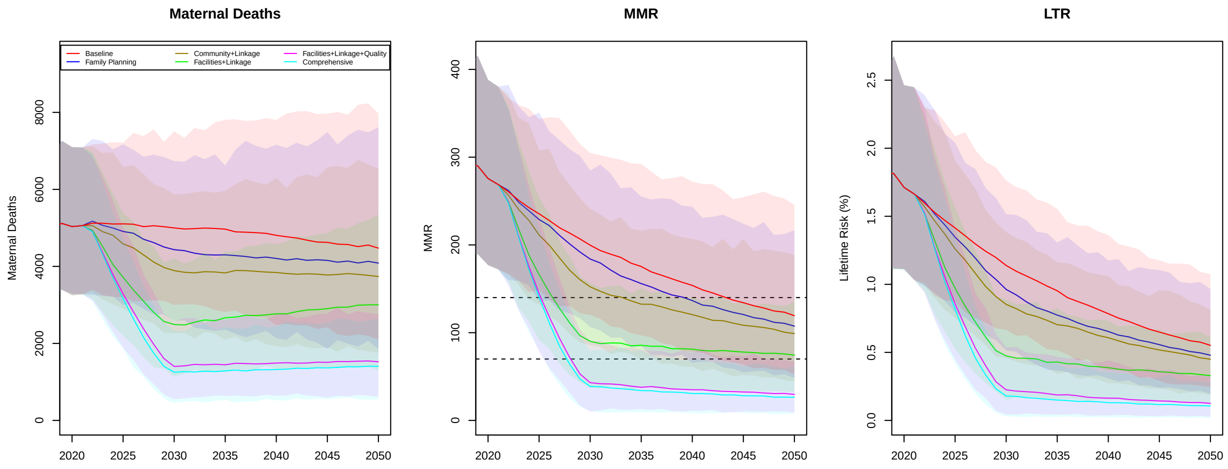

Antigua and Barbuda

ISO Code	Region	Area	Income Group
ATG	Caribbean	Latin America and the Caribbean	High income

Projected Maternal Indicators in 2030 by Scenario

Scenario	Maternal Deaths	MMR	LTR
**Baseline**	2 (0-10)	190 (0-1104)	0.25 (0-1.43)
**Family Planning Interventions**			
Contraception	2 (0-9)	170 (0-1139)	0.22 (0-1.37)
Medical abortion	1 (0-6)	116 (0-665)	0.15 (0-0.89)
**Community-Based Interventions**			
ANC	2 (0-11)	185 (0-1184)	0.25 (0-1.58)
SBA	2 (0-10)	190 (0-1104)	0.25 (0-1.43)
**Facility-Based Interventions**			
Facility births	2 (0-10)	186 (0-1104)	0.25 (0-1.43)
nonEmOC services	2 (0-10)	190 (0-1104)	0.25 (0-1.43)
bEmOC services	2 (0-10)	190 (0-1104)	0.25 (0-1.43)
cEmOC services	2 (0-10)	184 (0-1104)	0.24 (0-1.43)
**System-Relevant Interventions**			
Quality of care	2 (0-10)	180 (0-1119)	0.24 (0-1.43)
Referral	2 (0-10)	190 (0-1104)	0.25 (0-1.43)
Transport	2 (0-9)	179 (0-1003)	0.23 (0-1.3)
Targeted transfers	2 (0-10)	190 (0-1104)	0.25 (0-1.43)
**Integrated Strategies**			
Family Planning	1 (0-5)	97 (0-523)	0.13 (0-0.68)
Community + Linkages	2 (0-9)	175 (0-987)	0.23 (0-1.31)
Facilities + Linkages	2 (0-9)	170 (0-1033)	0.22 (0-1.32)
Facilities + Linkages + Quality	2 (0-9)	169 (0-1027)	0.22 (0-1.32)
Comprehensive	1 (0-4)	88 (0-491)	0.11 (0-0.65)

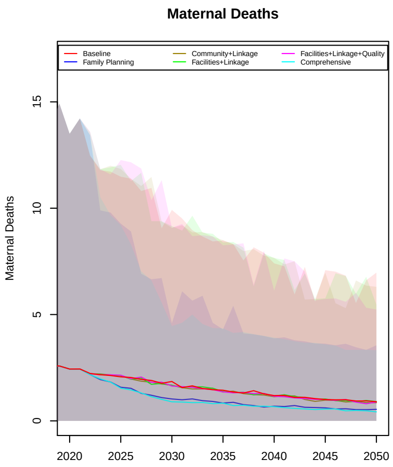

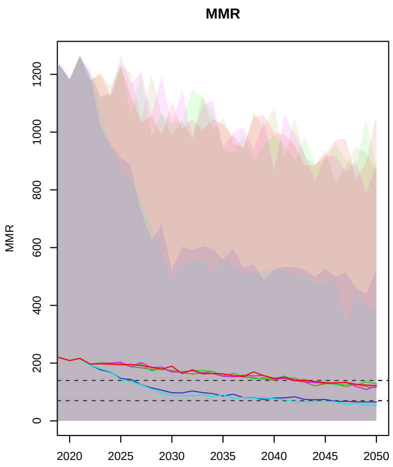

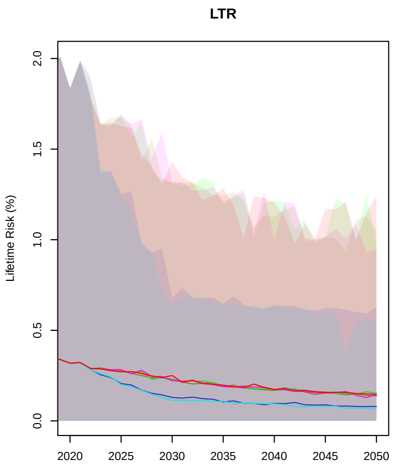

Argentina

ISO Code	Region	Area	Income Group
ARG	South America	Latin America and the Caribbean	High income

Projected Maternal Indicators in 2030 by Scenario

Scenario	Maternal Deaths	MMR	LTR
**Baseline**	357 (114-713)	34 (8-71)	0.08 (0.02-0.18)
**Family Planning Interventions**			
Contraception	335 (99-684)	33 (7-71)	0.08 (0.01-0.17)
Medical abortion	357 (114-713)	34 (8-71)	0.08 (0.02-0.18)
**Community-Based Interventions**			
ANC	360 (101-706)	34 (7-71)	0.08 (0.02-0.17)
SBA	364 (115-695)	34 (7-72)	0.08 (0.02-0.18)
**Facility-Based Interventions**			
Facility births	358 (114-703)	34 (8-72)	0.08 (0.02-0.18)
nonEmOC services	357 (114-713)	34 (8-71)	0.08 (0.02-0.18)
bEmOC services	358 (114-713)	34 (8-72)	0.08 (0.02-0.18)
cEmOC services	364 (124-719)	34 (8-75)	0.08 (0.02-0.18)
**System-Relevant Interventions**			
Quality of care	354 (112-673)	33 (8-69)	0.08 (0.02-0.16)
Referral	357 (111-706)	34 (8-72)	0.08 (0.02-0.17)
Transport	361 (115-695)	34 (7-71)	0.08 (0.02-0.17)
Targeted transfers	357 (114-713)	34 (8-71)	0.08 (0.02-0.18)
**Integrated Strategies**			
Family Planning	335 (99-684)	33 (7-71)	0.08 (0.01-0.17)
Community + Linkages	355 (111-678)	33 (8-70)	0.08 (0.02-0.17)
Facilities + Linkages	358 (114-704)	34 (7-74)	0.08 (0.01-0.17)
Facilities + Linkages + Quality	356 (113-668)	33 (7-68)	0.08 (0.02-0.17)
Comprehensive	326 (83-647)	33 (6-69)	0.07 (0.01-0.17)

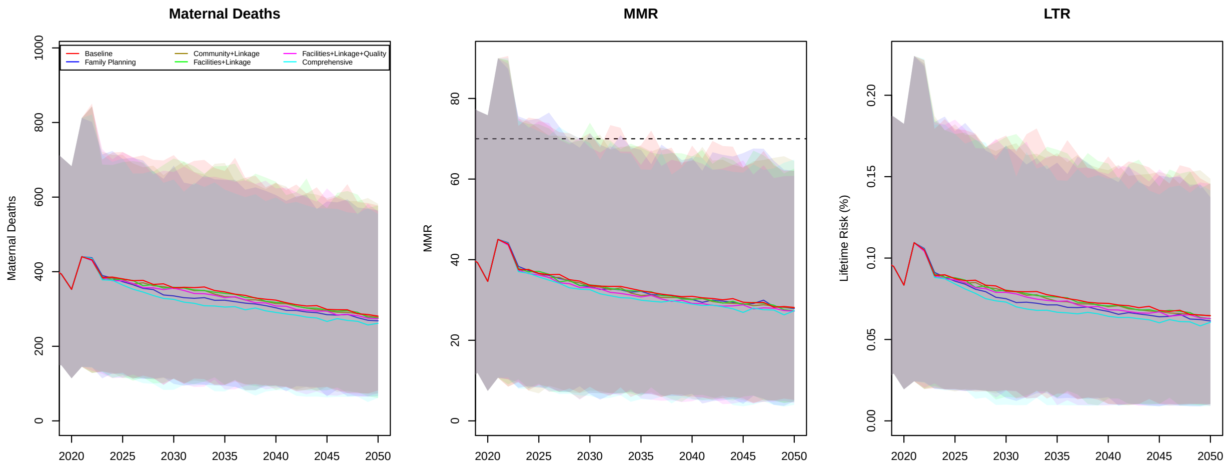

Armenia

ISO Code	Region	Area	Income Group
ARM	Western Asia	Asia	Upper middle income

Projected Maternal Indicators in 2030 by Scenario

Scenario	Maternal Deaths	MMR	LTR
**Baseline**	9 (0-30)	31 (0-132)	0.04 (0-0.14)
**Family Planning Interventions**			
Contraception	10 (0-32)	21 (0-86)	0.03 (0-0.13)
Medical abortion	9 (0-30)	32 (0-136)	0.04 (0-0.15)
**Community-Based Interventions**			
ANC	9 (0-29)	29 (0-127)	0.03 (0-0.14)
SBA	9 (0-30)	31 (0-130)	0.04 (0-0.15)
**Facility-Based Interventions**			
Facility births	9 (0-29)	30 (0-122)	0.03 (0-0.14)
nonEmOC services	9 (0-30)	32 (0-132)	0.04 (0-0.14)
bEmOC services	9 (0-30)	32 (0-139)	0.04 (0-0.14)
cEmOC services	9 (0-30)	32 (0-129)	0.04 (0-0.14)
**System-Relevant Interventions**			
Quality of care	9 (0-28)	29 (0-124)	0.03 (0-0.14)
Referral	9 (0-30)	32 (0-132)	0.04 (0-0.15)
Transport	9 (0-30)	29 (0-132)	0.03 (0-0.15)
Targeted transfers	9 (0-30)	32 (0-132)	0.04 (0-0.15)
**Integrated Strategies**			
Family Planning	10 (0-32)	22 (0-87)	0.04 (0-0.14)
Community + Linkages	8 (0-29)	27 (0-121)	0.03 (0-0.14)
Facilities + Linkages	8 (0-29)	27 (0-118)	0.03 (0-0.13)
Facilities + Linkages + Quality	8 (0-28)	26 (0-107)	0.03 (0-0.13)
Comprehensive	9 (0-29)	19 (0-72)	0.03 (0-0.13)

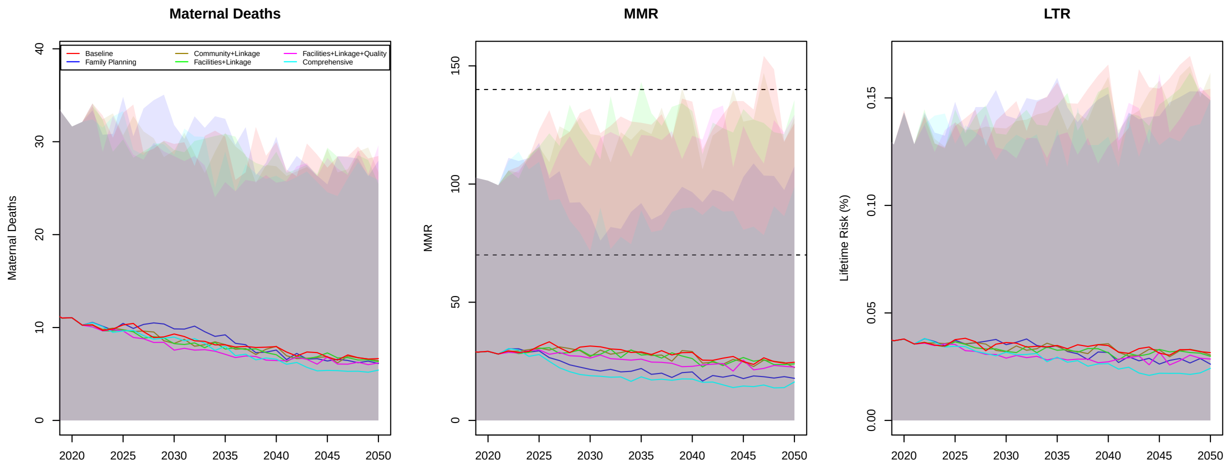

Australia

ISO Code	Region	Area	Income Group
AUS	Australia/New Zealand	Oceania	High income

Projected Maternal Indicators in 2030 by Scenario

Scenario	Maternal Deaths	MMR	LTR
**Baseline**	132 (0-346)	34 (0-88)	0.06 (0-0.17)
**Family Planning Interventions**			
Contraception	128 (0-325)	34 (0-88)	0.06 (0-0.16)
Medical abortion	132 (0-353)	34 (0-91)	0.06 (0-0.18)
**Community-Based Interventions**			
ANC	128 (0-321)	33 (0-84)	0.06 (0-0.16)
SBA	131 (0-338)	34 (0-88)	0.06 (0-0.17)
**Facility-Based Interventions**			
Facility births	130 (0-327)	34 (0-86)	0.06 (0-0.17)
nonEmOC services	131 (0-346)	34 (0-88)	0.06 (0-0.17)
bEmOC services	131 (0-336)	34 (0-88)	0.06 (0-0.17)
cEmOC services	131 (0-325)	34 (0-91)	0.06 (0-0.16)
**System-Relevant Interventions**			
Quality of care	132 (0-346)	34 (0-88)	0.06 (0-0.17)
Referral	131 (0-336)	34 (0-86)	0.06 (0-0.17)
Transport	131 (0-331)	34 (0-90)	0.06 (0-0.17)
Targeted transfers	131 (0-332)	34 (0-85)	0.06 (0-0.17)
**Integrated Strategies**			
Family Planning	128 (0-324)	33 (0-89)	0.06 (0-0.16)
Community + Linkages	129 (0-334)	33 (0-88)	0.06 (0-0.16)
Facilities + Linkages	128 (0-334)	33 (0-89)	0.06 (0-0.17)
Facilities + Linkages + Quality	128 (0-334)	33 (0-89)	0.06 (0-0.17)
Comprehensive	127 (0-320)	33 (0-82)	0.06 (0-0.16)

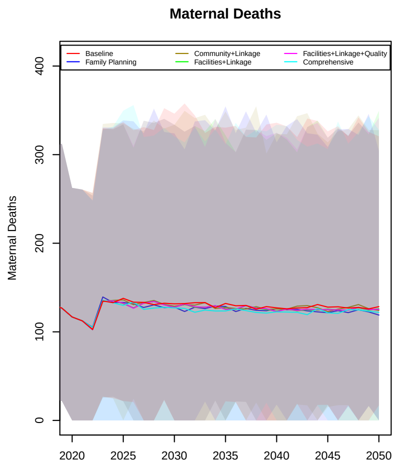

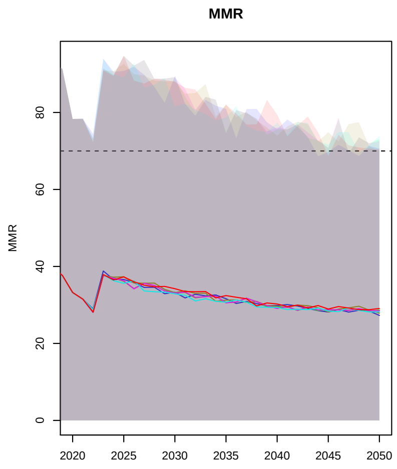

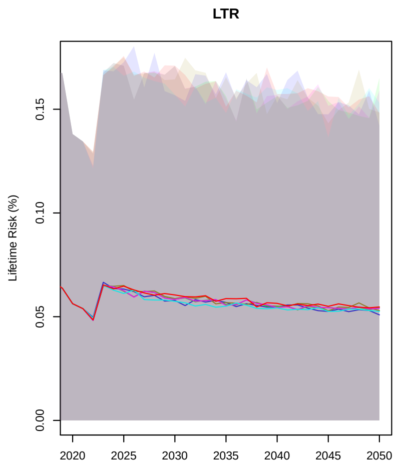

Austria

ISO Code	Region	Area	Income Group
AUT	Western Europe	Europe	High income

Projected Maternal Indicators in 2030 by Scenario

Scenario	Maternal Deaths	MMR	LTR
**Baseline**	24 (0-56)	15 (0-47)	0.02 (0-0.07)
**Family Planning Interventions**			
Contraception	24 (0-54)	15 (0-43)	0.02 (0-0.06)
Medical abortion	24 (0-56)	15 (0-47)	0.02 (0-0.07)
**Community-Based Interventions**			
ANC	25 (0-59)	15 (0-46)	0.02 (0-0.07)
SBA	24 (0-56)	15 (0-47)	0.02 (0-0.07)
**Facility-Based Interventions**			
Facility births	24 (0-56)	15 (0-47)	0.02 (0-0.07)
nonEmOC services	24 (0-56)	15 (0-47)	0.02 (0-0.07)
bEmOC services	24 (0-56)	15 (0-47)	0.02 (0-0.07)
cEmOC services	24 (6-53)	15 (0-44)	0.02 (0-0.07)
**System-Relevant Interventions**			
Quality of care	24 (0-56)	15 (0-47)	0.02 (0-0.07)
Referral	24 (0-56)	15 (0-47)	0.02 (0-0.07)
Transport	24 (0-56)	15 (0-46)	0.02 (0-0.06)
Targeted transfers	24 (0-56)	15 (0-47)	0.02 (0-0.07)
**Integrated Strategies**			
Family Planning	24 (0-54)	15 (0-43)	0.02 (0-0.06)
Community + Linkages	23 (0-52)	14 (0-44)	0.02 (0-0.06)
Facilities + Linkages	23 (0-52)	15 (0-47)	0.02 (0-0.07)
Facilities + Linkages + Quality	23 (0-52)	15 (0-47)	0.02 (0-0.07)
Comprehensive	22 (0-52)	14 (0-44)	0.02 (0-0.06)

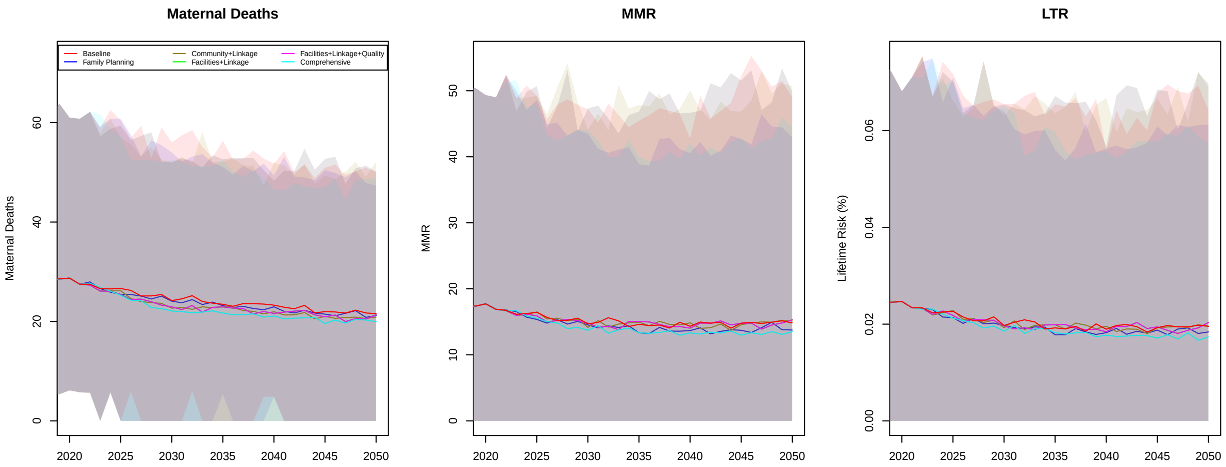

Azerbaijan

ISO Code	Region	Area	Income Group
AZE	Western Asia	Asia	Upper middle income

Projected Maternal Indicators in 2030 by Scenario

Scenario	Maternal Deaths	MMR	LTR
**Baseline**	80 (0-217)	34 (0-101)	0.06 (0-0.21)
**Family Planning Interventions**			
Contraception	93 (0-241)	25 (0-77)	0.07 (0-0.23)
Medical abortion	80 (0-219)	34 (0-109)	0.07 (0-0.22)
**Community-Based Interventions**			
ANC	82 (0-214)	34 (0-108)	0.06 (0-0.21)
SBA	83 (0-228)	34 (0-110)	0.07 (0-0.23)
**Facility-Based Interventions**			
Facility births	73 (0-199)	29 (0-96)	0.06 (0-0.2)
nonEmOC services	81 (0-219)	34 (0-103)	0.06 (0-0.21)
bEmOC services	81 (0-218)	34 (0-102)	0.06 (0-0.21)
cEmOC services	81 (0-228)	33 (0-102)	0.06 (0-0.21)
**System-Relevant Interventions**			
Quality of care	79 (0-216)	34 (0-102)	0.06 (0-0.21)
Referral	81 (0-213)	34 (0-101)	0.06 (0-0.21)
Transport	82 (0-233)	35 (0-115)	0.07 (0-0.22)
Targeted transfers	80 (0-219)	34 (0-101)	0.06 (0-0.21)
**Integrated Strategies**			
Family Planning	93 (0-231)	25 (0-77)	0.07 (0-0.23)
Community + Linkages	77 (0-211)	32 (0-109)	0.06 (0-0.21)
Facilities + Linkages	71 (0-214)	29 (0-109)	0.05 (0-0.2)
Facilities + Linkages + Quality	70 (0-204)	28 (0-101)	0.05 (0-0.2)
Comprehensive	75 (0-207)	19 (0-63)	0.05 (0-0.18)

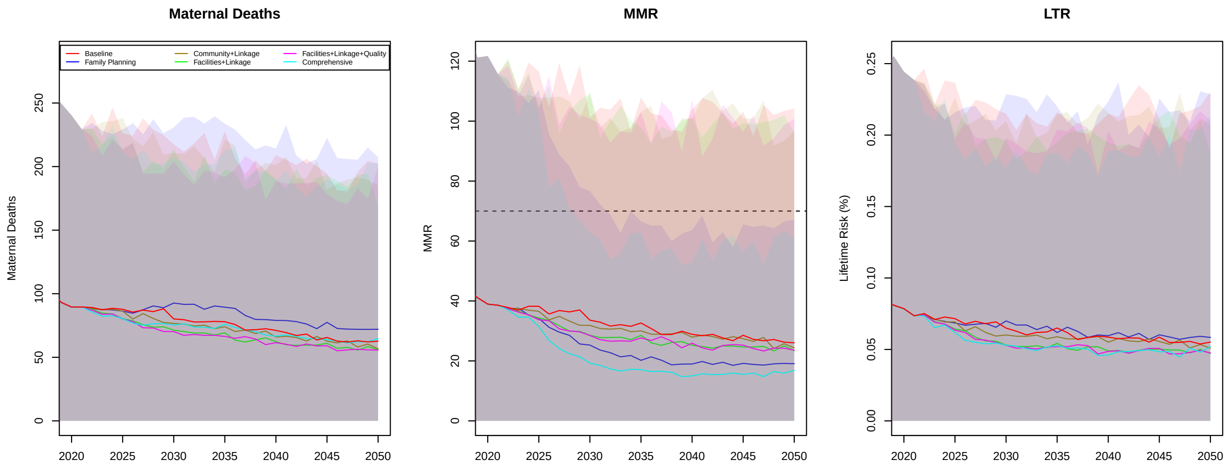

Bahamas

ISO Code	Region	Area	Income Group
BHS	Caribbean	Latin America and the Caribbean	High income

Projected Maternal Indicators in 2030 by Scenario

Scenario	Maternal Deaths	MMR	LTR
**Baseline**	6 (0-25)	110 (0-439)	0.17 (0-0.69)
**Family Planning Interventions**			
Contraception	5 (0-22)	95 (0-396)	0.14 (0-0.59)
Medical abortion	6 (0-24)	101 (0-423)	0.15 (0-0.65)
**Community-Based Interventions**			
ANC	6 (0-23)	97 (0-402)	0.15 (0-0.64)
SBA	6 (0-25)	110 (0-439)	0.17 (0-0.69)
**Facility-Based Interventions**			
Facility births	6 (0-25)	111 (0-450)	0.17 (0-0.7)
nonEmOC services	6 (0-25)	110 (0-439)	0.17 (0-0.69)
bEmOC services	6 (0-25)	110 (0-439)	0.17 (0-0.69)
cEmOC services	6 (0-24)	109 (0-442)	0.17 (0-0.68)
**System-Relevant Interventions**			
Quality of care	6 (0-24)	109 (0-439)	0.17 (0-0.68)
Referral	6 (0-25)	110 (0-439)	0.17 (0-0.69)
Transport	6 (0-25)	107 (0-462)	0.16 (0-0.67)
Targeted transfers	6 (0-25)	110 (0-439)	0.17 (0-0.69)
**Integrated Strategies**			
Family Planning	5 (0-22)	87 (0-391)	0.12 (0-0.64)
Community + Linkages	6 (0-24)	99 (0-436)	0.15 (0-0.67)
Facilities + Linkages	6 (0-24)	104 (0-448)	0.16 (0-0.65)
Facilities + Linkages + Quality	6 (0-24)	104 (0-448)	0.16 (0-0.65)
Comprehensive	5 (0-22)	86 (0-385)	0.12 (0-0.51)

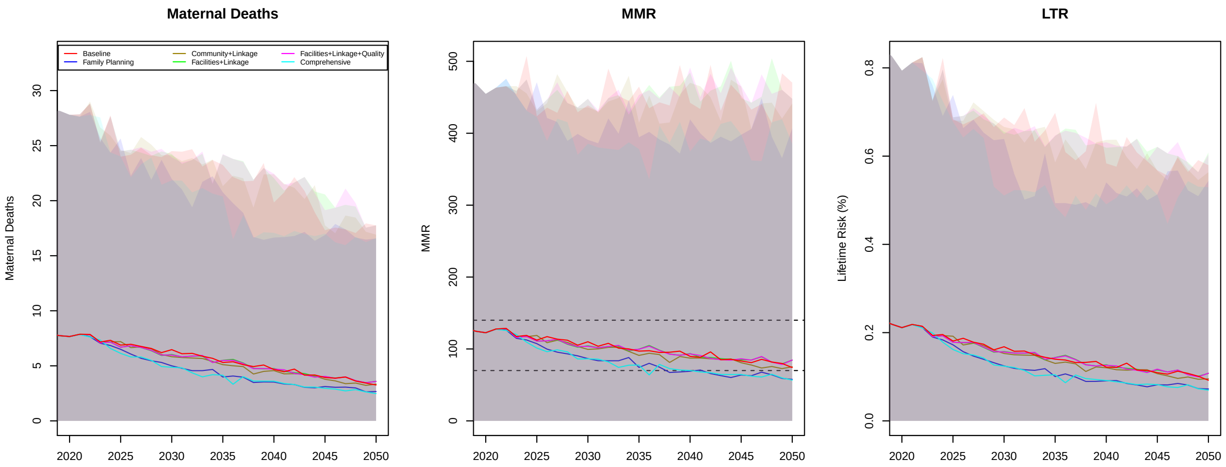

Bahrain

ISO Code	Region	Area	Income Group
BHR	Western Asia	Asia	High income

Projected Maternal Indicators in 2030 by Scenario

Scenario	Maternal Deaths	MMR	LTR
**Baseline**	7 (0-32)	33 (0-194)	0.05 (0-0.28)
**Family Planning Interventions**			
Contraception	6 (0-26)	19 (0-113)	0.04 (0-0.23)
Medical abortion	6 (0-26)	28 (0-173)	0.04 (0-0.25)
**Community-Based Interventions**			
ANC	7 (0-32)	32 (0-188)	0.05 (0-0.3)
SBA	7 (0-32)	34 (0-195)	0.05 (0-0.28)
**Facility-Based Interventions**			
Facility births	7 (0-33)	34 (0-196)	0.05 (0-0.33)
nonEmOC services	7 (0-32)	33 (0-194)	0.05 (0-0.28)
bEmOC services	7 (0-32)	33 (0-194)	0.05 (0-0.28)
cEmOC services	7 (0-32)	33 (0-189)	0.05 (0-0.28)
**System-Relevant Interventions**			
Quality of care	7 (0-32)	33 (0-194)	0.05 (0-0.28)
Referral	7 (0-32)	33 (0-196)	0.05 (0-0.3)
Transport	6 (0-32)	31 (0-179)	0.05 (0-0.25)
Targeted transfers	7 (0-32)	33 (0-194)	0.05 (0-0.28)
**Integrated Strategies**			
Family Planning	5 (0-26)	17 (0-104)	0.03 (0-0.21)
Community + Linkages	7 (0-32)	31 (0-201)	0.05 (0-0.3)
Facilities + Linkages	7 (0-32)	31 (0-175)	0.05 (0-0.25)
Facilities + Linkages + Quality	7 (0-32)	32 (0-175)	0.05 (0-0.25)
Comprehensive	5 (0-26)	16 (0-109)	0.03 (0-0.21)

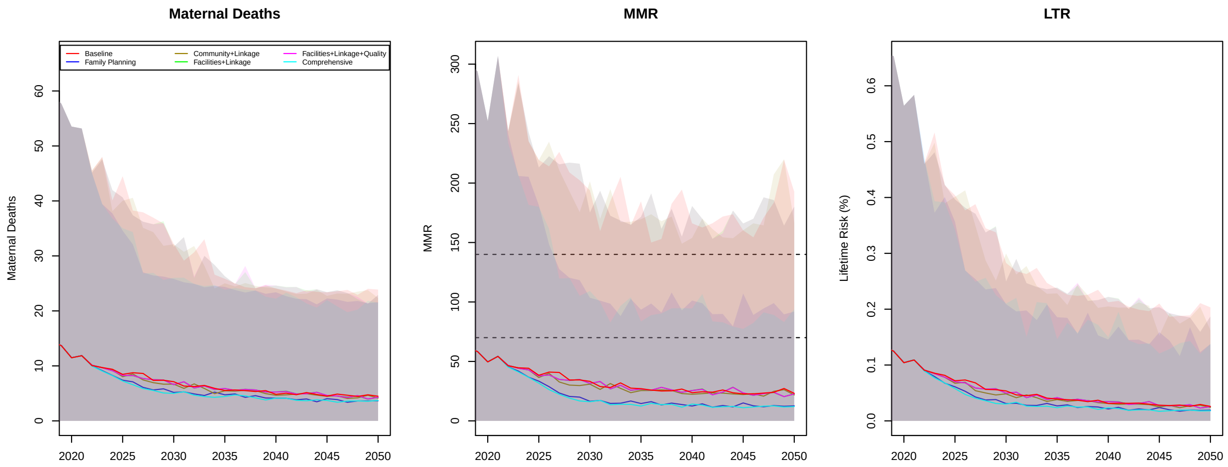

Bangladesh

ISO Code	Region	Area	Income Group
BGD	Southern Asia	Asia	Lower middle income

Projected Maternal Indicators in 2030 by Scenario

Scenario	Maternal Deaths	MMR	LTR
**Baseline**	4713 (2476-6972)	113 (54-182)	0.23 (0.11-0.38)
**Family Planning Interventions**			
Contraception	4560 (2209-6880)	109 (53-178)	0.22 (0.09-0.36)
Medical abortion	4558 (2256-6799)	108 (52-178)	0.22 (0.09-0.37)
**Community-Based Interventions**			
ANC	4635 (2548-6791)	110 (53-174)	0.23 (0.11-0.36)
SBA	4547 (2471-6721)	108 (55-174)	0.22 (0.1-0.35)
**Facility-Based Interventions**			
Facility births	3268 (1726-5137)	66 (31-120)	0.13 (0.05-0.25)
nonEmOC services	4719 (2581-6926)	113 (57-175)	0.23 (0.11-0.37)
bEmOC services	4698 (2620-6952)	112 (55-181)	0.23 (0.11-0.37)
cEmOC services	4635 (2562-6905)	110 (50-178)	0.23 (0.09-0.37)
**System-Relevant Interventions**			
Quality of care	3442 (1666-5341)	74 (20-143)	0.15 (0.05-0.28)
Referral	4661 (2602-6768)	111 (54-178)	0.23 (0.11-0.36)
Transport	4515 (2407-6757)	108 (52-178)	0.22 (0.1-0.36)
Targeted transfers	4701 (2573-7113)	112 (55-182)	0.23 (0.11-0.37)
**Integrated Strategies**			
Family Planning	4486 (2159-6766)	106 (53-171)	0.22 (0.08-0.36)
Community + Linkages	4174 (2162-6191)	97 (49-157)	0.2 (0.1-0.31)
Facilities + Linkages	3101 (1571-5001)	62 (30-110)	0.13 (0.05-0.24)
Facilities + Linkages + Quality	1730 (903-2825)	22 (7-65)	0.04 (0.01-0.12)
Comprehensive	1483 (770-2454)	15 (5-33)	0.03 (0.01-0.07)

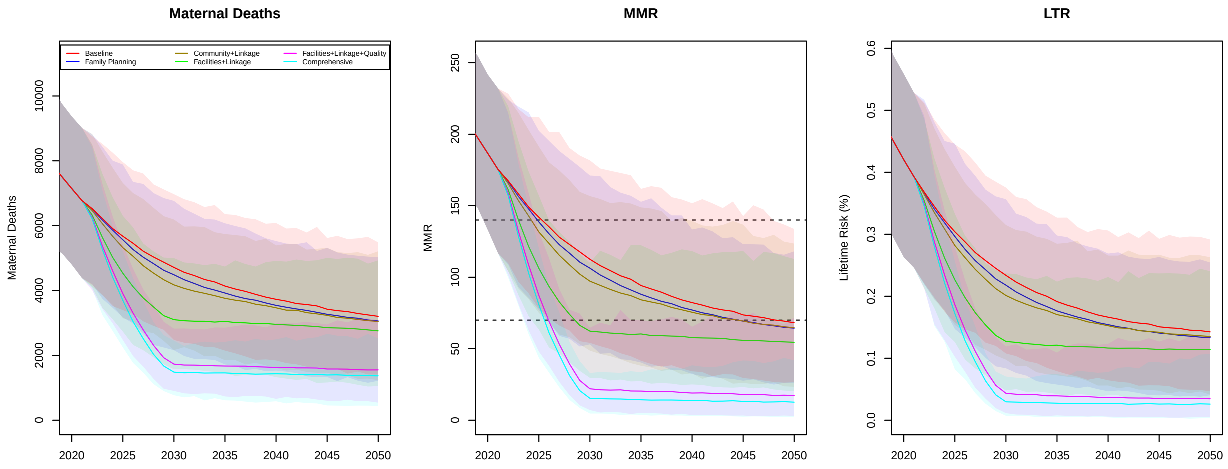

Barbados

ISO Code	Region	Area	Income Group
BRB	Caribbean	Latin America and the Caribbean	High income

Projected Maternal Indicators in 2030 by Scenario

Scenario	Maternal Deaths	MMR	LTR
**Baseline**	5 (0-21)	140 (0-655)	0.26 (0-1.1)
**Family Planning Interventions**			
Contraception	3 (0-16)	103 (0-522)	0.16 (0-0.83)
Medical abortion	4 (0-15)	100 (0-484)	0.18 (0-0.84)
**Community-Based Interventions**			
ANC	5 (0-19)	134 (0-616)	0.24 (0-1.06)
SBA	5 (0-20)	138 (0-630)	0.25 (0-1.09)
**Facility-Based Interventions**			
Facility births	5 (0-21)	140 (0-653)	0.25 (0-1.16)
nonEmOC services	5 (0-21)	141 (0-655)	0.26 (0-1.1)
bEmOC services	5 (0-21)	141 (0-655)	0.26 (0-1.1)
cEmOC services	5 (0-21)	138 (0-655)	0.25 (0-1.1)
**System-Relevant Interventions**			
Quality of care	5 (0-21)	144 (0-630)	0.26 (0-1.19)
Referral	5 (0-21)	140 (0-655)	0.26 (0-1.1)
Transport	5 (0-21)	134 (0-647)	0.24 (0-1.13)
Targeted transfers	5 (0-21)	140 (0-655)	0.26 (0-1.1)
**Integrated Strategies**			
Family Planning	3 (0-11)	78 (0-431)	0.12 (0-0.61)
Community + Linkages	4 (0-20)	128 (0-628)	0.23 (0-1.03)
Facilities + Linkages	5 (0-21)	133 (0-645)	0.24 (0-1.17)
Facilities + Linkages + Quality	5 (0-21)	141 (0-668)	0.25 (0-1.17)
Comprehensive	3 (0-13)	82 (0-426)	0.13 (0-0.6)

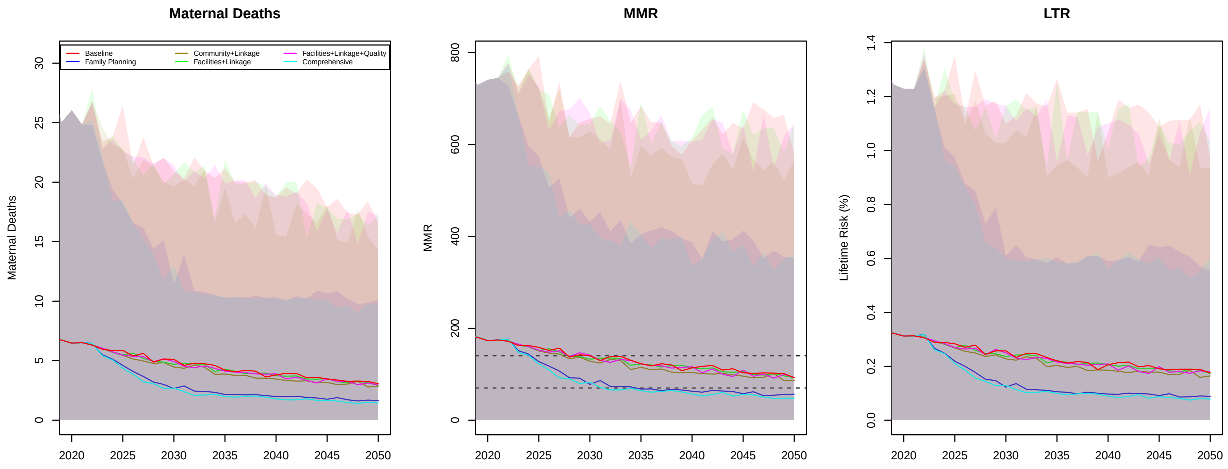

Belarus

ISO Code	Region	Area	Income Group
BLR	Eastern Europe	Europe	Upper middle income

Projected Maternal Indicators in 2030 by Scenario

Scenario	Maternal Deaths	MMR	LTR
**Baseline**	57 (9-137)	40 (0-137)	0.06 (0-0.19)
**Family Planning Interventions**			
Contraception	56 (5-132)	38 (0-120)	0.06 (0-0.17)
Medical abortion	57 (10-133)	39 (0-117)	0.06 (0-0.17)
**Community-Based Interventions**			
ANC	56 (9-130)	40 (0-127)	0.06 (0-0.18)
SBA	57 (9-137)	40 (0-137)	0.06 (0-0.19)
**Facility-Based Interventions**			
Facility births	57 (9-137)	40 (0-137)	0.06 (0-0.19)
nonEmOC services	57 (9-136)	40 (0-136)	0.06 (0-0.19)
bEmOC services	57 (9-134)	40 (0-131)	0.06 (0-0.17)
cEmOC services	58 (10-142)	41 (0-136)	0.06 (0-0.2)
**System-Relevant Interventions**			
Quality of care	57 (9-137)	40 (0-137)	0.06 (0-0.19)
Referral	58 (9-137)	40 (0-139)	0.06 (0-0.19)
Transport	55 (5-136)	40 (0-135)	0.06 (0-0.2)
Targeted transfers	58 (9-143)	41 (0-139)	0.06 (0-0.19)
**Integrated Strategies**			
Family Planning	54 (9-125)	36 (0-110)	0.05 (0-0.16)
Community + Linkages	56 (5-136)	40 (0-128)	0.06 (0-0.19)
Facilities + Linkages	53 (5-132)	37 (0-131)	0.05 (0-0.18)
Facilities + Linkages + Quality	53 (5-132)	37 (0-131)	0.05 (0-0.18)
Comprehensive	49 (5-120)	33 (0-108)	0.05 (0-0.16)

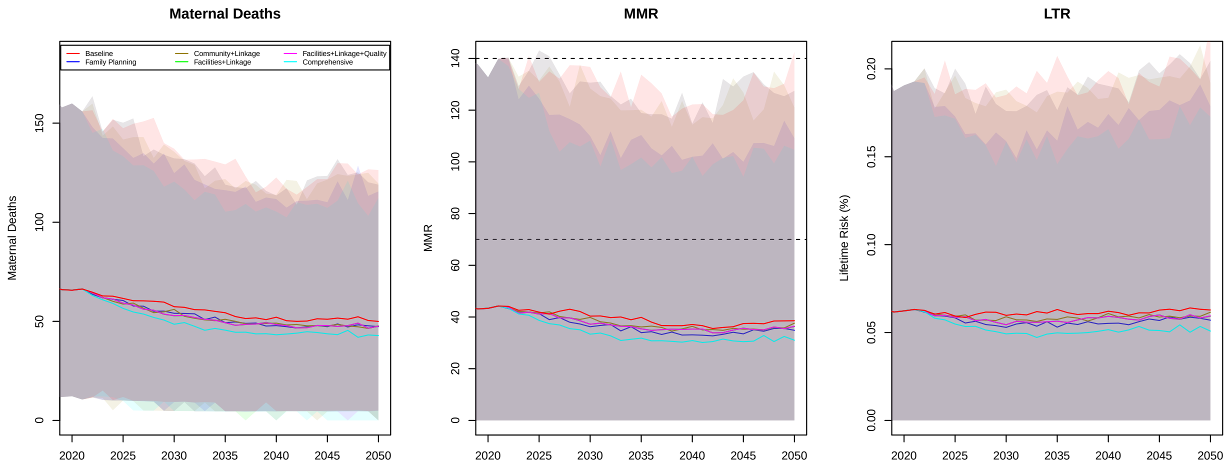

Belgium

ISO Code	Region	Area	Income Group
BEL	Western Europe	Europe	High income

Projected Maternal Indicators in 2030 by Scenario

Scenario	Maternal Deaths	MMR	LTR
**Baseline**	42 (0-95)	20 (0-56)	0.03 (0-0.1)
**Family Planning Interventions**			
Contraception	39 (0-95)	20 (0-57)	0.03 (0-0.09)
Medical abortion	42 (0-99)	20 (0-62)	0.03 (0-0.1)
**Community-Based Interventions**			
ANC	41 (0-91)	19 (0-53)	0.03 (0-0.09)
SBA	41 (0-93)	20 (0-56)	0.03 (0-0.1)
**Facility-Based Interventions**			
Facility births	42 (0-95)	20 (0-56)	0.03 (0-0.1)
nonEmOC services	42 (0-100)	20 (0-57)	0.03 (0-0.1)
bEmOC services	42 (0-93)	20 (0-56)	0.03 (0-0.1)
cEmOC services	41 (0-99)	19 (0-56)	0.03 (0-0.1)
**System-Relevant Interventions**			
Quality of care	42 (0-95)	20 (0-56)	0.03 (0-0.1)
Referral	41 (0-95)	20 (0-56)	0.03 (0-0.1)
Transport	40 (0-91)	20 (0-55)	0.03 (0-0.1)
Targeted transfers	42 (0-95)	20 (0-56)	0.03 (0-0.1)
**Integrated Strategies**			
Family Planning	39 (0-91)	20 (0-55)	0.03 (0-0.09)
Community + Linkages	38 (0-91)	18 (0-54)	0.03 (0-0.09)
Facilities + Linkages	41 (0-97)	19 (0-54)	0.03 (0-0.09)
Facilities + Linkages + Quality	41 (0-97)	19 (0-54)	0.03 (0-0.09)
Comprehensive	38 (0-89)	18 (0-54)	0.03 (0-0.09)

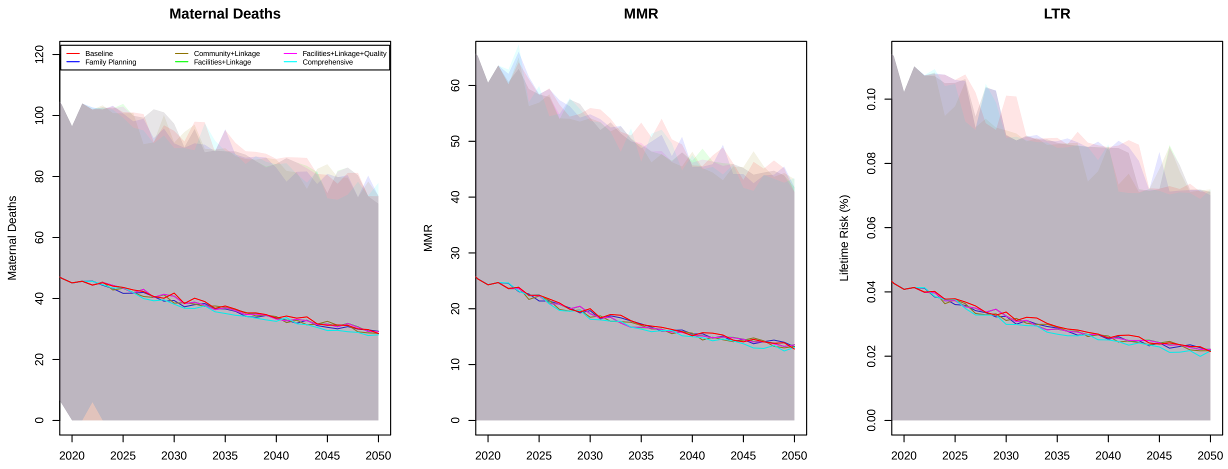

Belize

ISO Code	Region	Area	Income Group
BLZ	Central America	Latin America and the Caribbean	Upper middle income

Projected Maternal Indicators in 2030 by Scenario

Scenario	Maternal Deaths	MMR	LTR
**Baseline**	9 (0-27)	81 (0-271)	0.21 (0-0.71)
**Family Planning Interventions**			
Contraception	7 (0-23)	65 (0-239)	0.15 (0-0.59)
Medical abortion	7 (0-23)	64 (0-225)	0.16 (0-0.56)
**Community-Based Interventions**			
ANC	9 (0-27)	82 (0-267)	0.21 (0-0.7)
SBA	9 (0-28)	82 (0-279)	0.21 (0-0.74)
**Facility-Based Interventions**			
Facility births	9 (0-28)	82 (0-278)	0.21 (0-0.71)
nonEmOC services	9 (0-28)	82 (0-270)	0.21 (0-0.71)
bEmOC services	9 (0-28)	82 (0-278)	0.21 (0-0.72)
cEmOC services	9 (0-28)	82 (0-284)	0.21 (0-0.74)
**System-Relevant Interventions**			
Quality of care	9 (0-28)	79 (0-276)	0.2 (0-0.71)
Referral	9 (0-28)	82 (0-284)	0.21 (0-0.73)
Transport	9 (0-25)	82 (0-258)	0.21 (0-0.66)
Targeted transfers	9 (0-28)	83 (0-279)	0.21 (0-0.72)
**Integrated Strategies**			
Family Planning	6 (0-20)	58 (0-214)	0.13 (0-0.52)
Community + Linkages	9 (0-27)	81 (0-268)	0.21 (0-0.7)
Facilities + Linkages	9 (0-28)	82 (0-266)	0.21 (0-0.71)
Facilities + Linkages + Quality	9 (0-28)	81 (0-265)	0.21 (0-0.7)
Comprehensive	6 (0-19)	53 (0-196)	0.12 (0-0.45)

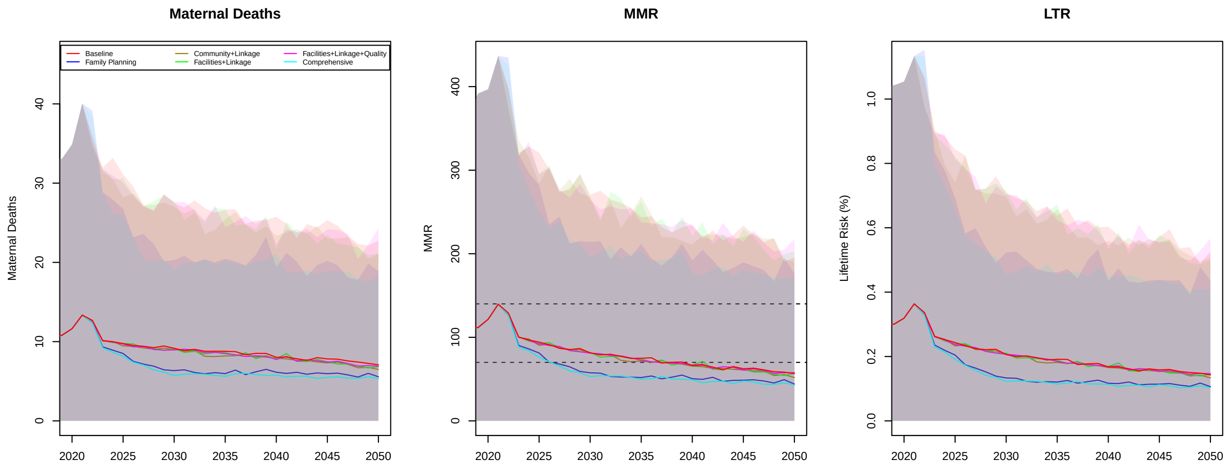

Benin

ISO Code	Region	Area	Income Group
BEN	Western Africa	Africa	Low income

Projected Maternal Indicators in 2030 by Scenario

Scenario	Maternal Deaths	MMR	LTR
**Baseline**	1914 (989-3095)	289 (145-454)	1.29 (0.61-2.18)
**Family Planning Interventions**			
Contraception	1784 (860-2976)	276 (141-450)	1.18 (0.51-2.07)
Medical abortion	1862 (952-2949)	277 (136-434)	1.24 (0.56-2.09)
**Community-Based Interventions**			
ANC	1880 (983-3046)	282 (142-449)	1.26 (0.6-2.2)
SBA	1903 (1040-3052)	287 (153-442)	1.28 (0.65-2.18)
**Facility-Based Interventions**			
Facility births	1768 (890-2840)	263 (134-423)	1.18 (0.53-2.05)
nonEmOC services	1928 (972-3129)	289 (149-461)	1.29 (0.58-2.23)
bEmOC services	1905 (1003-3115)	287 (148-460)	1.29 (0.62-2.23)
cEmOC services	1886 (1013-3027)	285 (142-454)	1.27 (0.64-2.13)
**System-Relevant Interventions**			
Quality of care	979 (346-1934)	128 (34-276)	0.58 (0.13-1.37)
Referral	1857 (951-3043)	278 (146-445)	1.24 (0.59-2.08)
Transport	1867 (930-3038)	281 (139-445)	1.26 (0.57-2.17)
Targeted transfers	1882 (966-3029)	283 (142-452)	1.27 (0.6-2.19)
**Integrated Strategies**			
Family Planning	1741 (876-2733)	270 (138-419)	1.15 (0.51-1.96)
Community + Linkages	1730 (908-2710)	258 (133-424)	1.15 (0.53-1.99)
Facilities + Linkages	1591 (855-2663)	236 (123-399)	1.05 (0.5-1.84)
Facilities + Linkages + Quality	643 (182-1489)	74 (10-204)	0.32 (0.03-0.96)
Comprehensive	518 (132-1199)	57 (7-161)	0.24 (0.02-0.79)

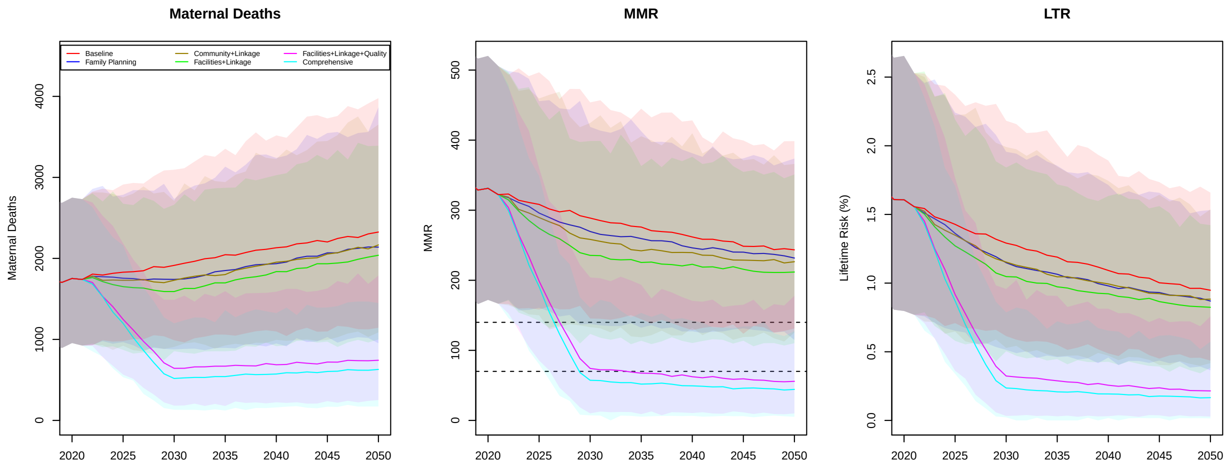

Bermuda

ISO Code	Region	Area	Income Group
BMU	Northern America	Northern America	High income

Projected Maternal Indicators in 2030 by Scenario

Scenario	Maternal Deaths	MMR	LTR
**Baseline**	0 (0-1)	11 (0-120)	0.03 (0-0.44)
**Family Planning Interventions**			
Contraception	0 (0-1)	10 (0-116)	0.03 (0-0.44)
Medical abortion	0 (0-1)	9 (0-119)	0.03 (0-0.43)
**Community-Based Interventions**			
ANC	0 (0-1)	11 (0-120)	0.03 (0-0.44)
SBA	0 (0-1)	11 (0-120)	0.03 (0-0.44)
**Facility-Based Interventions**			
Facility births	0 (0-1)	11 (0-120)	0.03 (0-0.44)
nonEmOC services	0 (0-1)	11 (0-120)	0.03 (0-0.44)
bEmOC services	0 (0-1)	11 (0-120)	0.03 (0-0.44)
cEmOC services	0 (0-1)	11 (0-120)	0.03 (0-0.43)
**System-Relevant Interventions**			
Quality of care	0 (0-1)	11 (0-130)	0.03 (0-0.44)
Referral	0 (0-1)	11 (0-120)	0.03 (0-0.44)
Transport	0 (0-1)	11 (0-120)	0.03 (0-0.44)
Targeted transfers	0 (0-1)	11 (0-120)	0.03 (0-0.44)
**Integrated Strategies**			
Family Planning	0 (0-1)	9 (0-116)	0.03 (0-0.43)
Community + Linkages	0 (0-1)	11 (0-120)	0.03 (0-0.44)
Facilities + Linkages	0 (0-1)	11 (0-120)	0.03 (0-0.43)
Facilities + Linkages + Quality	0 (0-1)	11 (0-130)	0.03 (0-0.44)
Comprehensive	0 (0-1)	9 (0-118)	0.03 (0-0.43)

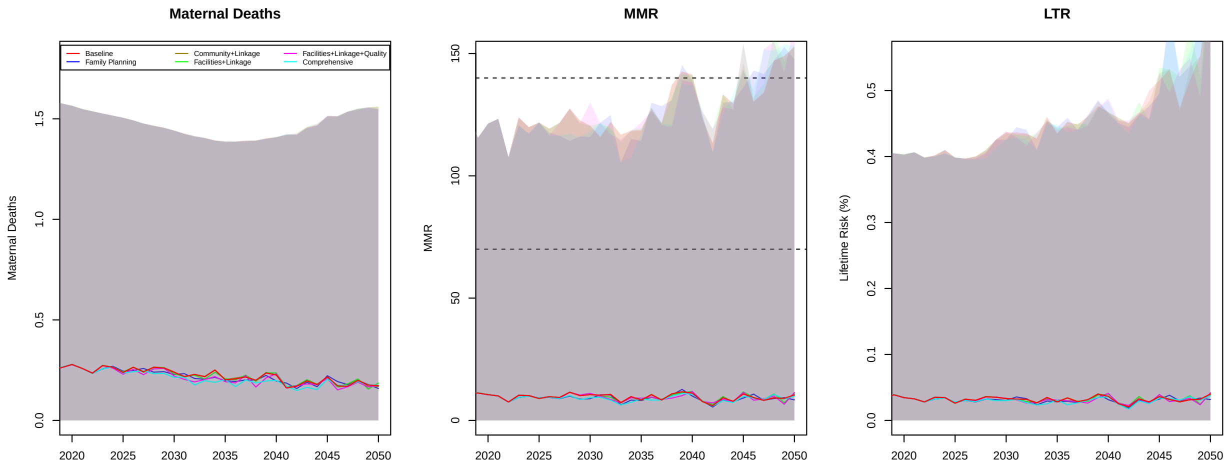

Bhutan

ISO Code	Region	Area	Income Group
BTN	Southern Asia	Asia	Lower middle income

Projected Maternal Indicators in 2030 by Scenario

Scenario	Maternal Deaths	MMR	LTR
**Baseline**	20 (0-69)	81 (0-319)	0.2 (0-0.81)
**Family Planning Interventions**			
Contraception	18 (0-67)	77 (0-337)	0.18 (0-0.79)
Medical abortion	21 (0-77)	87 (0-361)	0.21 (0-0.9)
**Community-Based Interventions**			
ANC	21 (0-70)	84 (0-342)	0.2 (0-0.89)
SBA	19 (0-67)	78 (0-300)	0.19 (0-0.79)
**Facility-Based Interventions**			
Facility births	17 (0-61)	65 (0-283)	0.15 (0-0.67)
nonEmOC services	19 (0-67)	78 (0-312)	0.19 (0-0.79)
bEmOC services	20 (0-70)	82 (0-333)	0.2 (0-0.83)
cEmOC services	19 (0-74)	78 (0-342)	0.19 (0-0.87)
**System-Relevant Interventions**			
Quality of care	16 (0-63)	59 (0-275)	0.14 (0-0.68)
Referral	21 (0-78)	83 (0-369)	0.2 (0-0.85)
Transport	19 (0-71)	81 (0-336)	0.2 (0-0.83)
Targeted transfers	21 (0-75)	86 (0-342)	0.21 (0-0.84)
**Integrated Strategies**			
Family Planning	18 (0-68)	77 (0-309)	0.18 (0-0.79)
Community + Linkages	17 (0-56)	66 (0-284)	0.16 (0-0.7)
Facilities + Linkages	16 (0-62)	60 (0-281)	0.14 (0-0.72)
Facilities + Linkages + Quality	12 (0-53)	43 (0-253)	0.1 (0-0.62)
Comprehensive	10 (0-46)	36 (0-220)	0.08 (0-0.51)

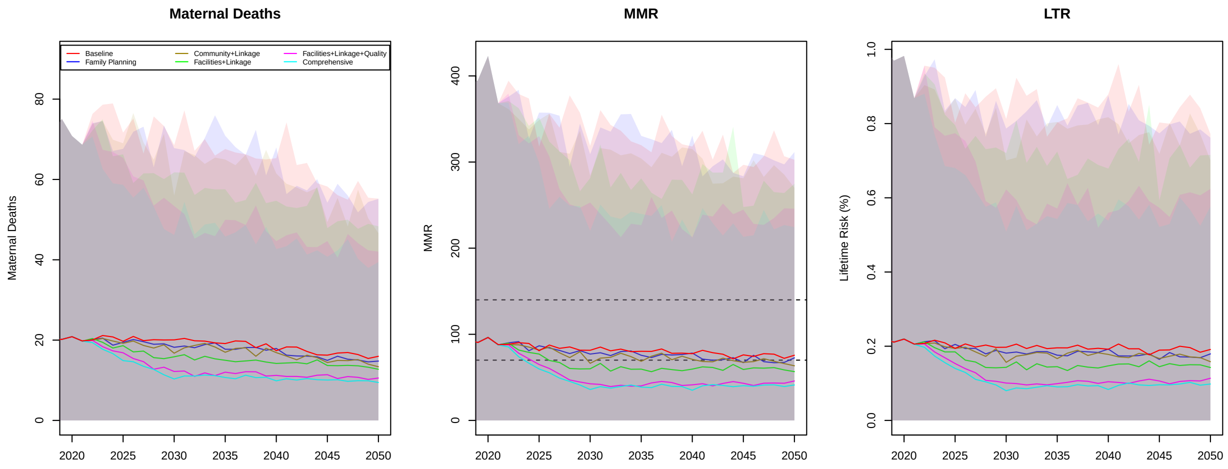

Bolivia (Plurinational State of)

ISO Code	Region	Area	Income Group
BOL	South America	Latin America and the Caribbean	Lower middle income

Projected Maternal Indicators in 2030 by Scenario

Scenario	Maternal Deaths	MMR	LTR
**Baseline**	537 (169-1055)	138 (37-288)	0.39 (0.1-0.83)
**Family Planning Interventions**			
Contraception	480 (149-1003)	136 (30-283)	0.35 (0.07-0.77)
Medical abortion	537 (173-1055)	138 (38-286)	0.39 (0.1-0.83)
**Community-Based Interventions**			
ANC	545 (169-1070)	140 (40-291)	0.4 (0.1-0.86)
SBA	502 (153-964)	125 (37-252)	0.36 (0.09-0.74)
**Facility-Based Interventions**			
Facility births	361 (57-847)	81 (6-196)	0.23 (0-0.61)
nonEmOC services	528 (166-1052)	136 (39-285)	0.39 (0.11-0.81)
bEmOC services	539 (170-1052)	138 (40-291)	0.39 (0.11-0.8)
cEmOC services	521 (163-991)	132 (32-277)	0.37 (0.09-0.77)
**System-Relevant Interventions**			
Quality of care	536 (165-1050)	138 (33-292)	0.39 (0.1-0.82)
Referral	534 (169-1047)	137 (39-299)	0.39 (0.1-0.82)
Transport	472 (136-942)	123 (34-255)	0.35 (0.08-0.77)
Targeted transfers	537 (155-1058)	139 (37-295)	0.39 (0.1-0.83)
**Integrated Strategies**			
Family Planning	479 (148-1003)	135 (30-283)	0.34 (0.07-0.77)
Community + Linkages	429 (117-902)	108 (24-235)	0.31 (0.06-0.73)
Facilities + Linkages	316 (44-734)	75 (0-186)	0.22 (0-0.59)
Facilities + Linkages + Quality	311 (40-710)	71 (0-171)	0.21 (0-0.52)
Comprehensive	261 (18-640)	67 (0-172)	0.17 (0-0.5)

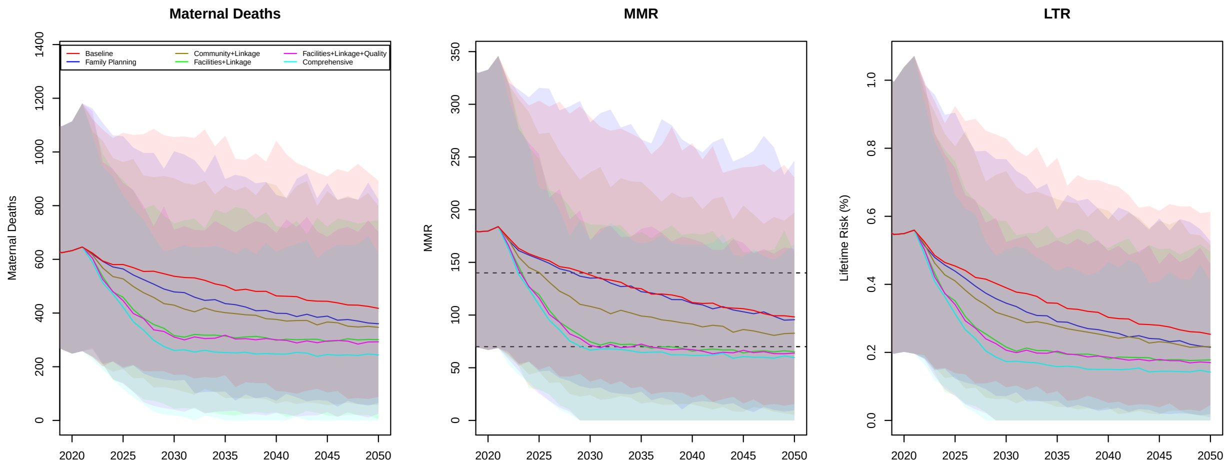

Bosnia and Herzegovina

ISO Code	Region	Area	Income Group
BIH	Southern Europe	Europe	Upper middle income

Projected Maternal Indicators in 2030 by Scenario

Scenario	Maternal Deaths	MMR	LTR
**Baseline**	17 (0-44)	59 (0-182)	0.07 (0-0.22)
**Family Planning Interventions**			
Contraception	14 (0-36)	52 (0-144)	0.06 (0-0.17)
Medical abortion	16 (0-48)	57 (0-186)	0.07 (0-0.23)
**Community-Based Interventions**			
ANC	17 (0-46)	58 (0-196)	0.07 (0-0.23)
SBA	17 (0-44)	58 (0-200)	0.07 (0-0.22)
**Facility-Based Interventions**			
Facility births	16 (0-46)	55 (0-191)	0.06 (0-0.22)
nonEmOC services	17 (0-45)	59 (0-182)	0.07 (0-0.22)
bEmOC services	17 (0-44)	60 (0-184)	0.07 (0-0.22)
cEmOC services	17 (0-42)	58 (0-183)	0.07 (0-0.21)
**System-Relevant Interventions**			
Quality of care	17 (0-46)	59 (0-186)	0.07 (0-0.22)
Referral	17 (0-46)	60 (0-190)	0.07 (0-0.23)
Transport	17 (0-46)	60 (0-196)	0.07 (0-0.23)
Targeted transfers	17 (0-43)	59 (0-182)	0.07 (0-0.22)
**Integrated Strategies**			
Family Planning	14 (0-38)	51 (0-164)	0.05 (0-0.17)
Community + Linkages	16 (0-45)	56 (0-188)	0.07 (0-0.23)
Facilities + Linkages	16 (0-44)	55 (0-196)	0.06 (0-0.22)
Facilities + Linkages + Quality	16 (0-44)	55 (0-196)	0.06 (0-0.23)
Comprehensive	12 (0-30)	44 (0-138)	0.05 (0-0.14)

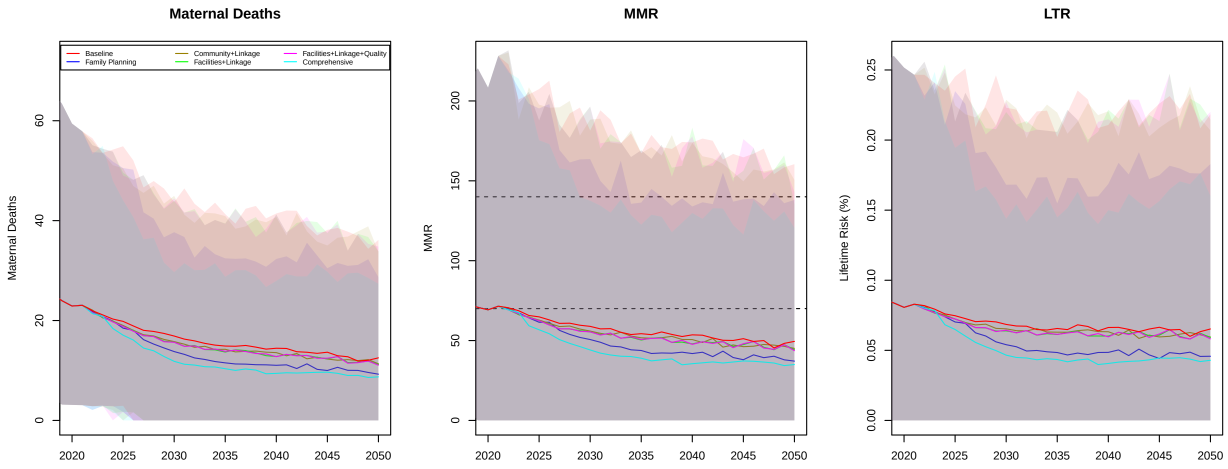

Botswana

ISO Code	Region	Area	Income Group
BWA	Southern Africa	Africa	Upper middle income

Projected Maternal Indicators in 2030 by Scenario

Scenario	Maternal Deaths	MMR	LTR
**Baseline**	132 (32-282)	213 (48-499)	0.54 (0.12-1.19)
**Family Planning Interventions**			
Contraception	107 (24-228)	179 (38-410)	0.43 (0.09-0.95)
Medical abortion	101 (20-222)	159 (28-354)	0.4 (0.07-0.87)
**Community-Based Interventions**			
ANC	128 (33-277)	206 (47-495)	0.53 (0.11-1.2)
SBA	132 (32-282)	213 (48-499)	0.54 (0.12-1.19)
**Facility-Based Interventions**			
Facility births	132 (32-283)	214 (48-493)	0.54 (0.12-1.2)
nonEmOC services	131 (29-283)	212 (46-499)	0.54 (0.11-1.19)
bEmOC services	133 (32-284)	215 (49-519)	0.55 (0.12-1.21)
cEmOC services	132 (34-279)	213 (52-503)	0.54 (0.12-1.21)
**System-Relevant Interventions**			
Quality of care	132 (32-282)	214 (48-499)	0.54 (0.12-1.21)
Referral	132 (32-282)	213 (48-491)	0.54 (0.11-1.19)
Transport	130 (35-273)	210 (54-493)	0.54 (0.13-1.22)
Targeted transfers	133 (32-288)	215 (53-519)	0.55 (0.13-1.21)
**Integrated Strategies**			
Family Planning	93 (16-210)	150 (22-335)	0.36 (0.05-0.85)
Community + Linkages	127 (33-268)	204 (53-458)	0.53 (0.13-1.14)
Facilities + Linkages	132 (32-284)	212 (52-498)	0.54 (0.11-1.19)
Facilities + Linkages + Quality	131 (31-284)	211 (52-498)	0.54 (0.11-1.19)
Comprehensive	90 (12-210)	147 (15-329)	0.36 (0.04-0.84)

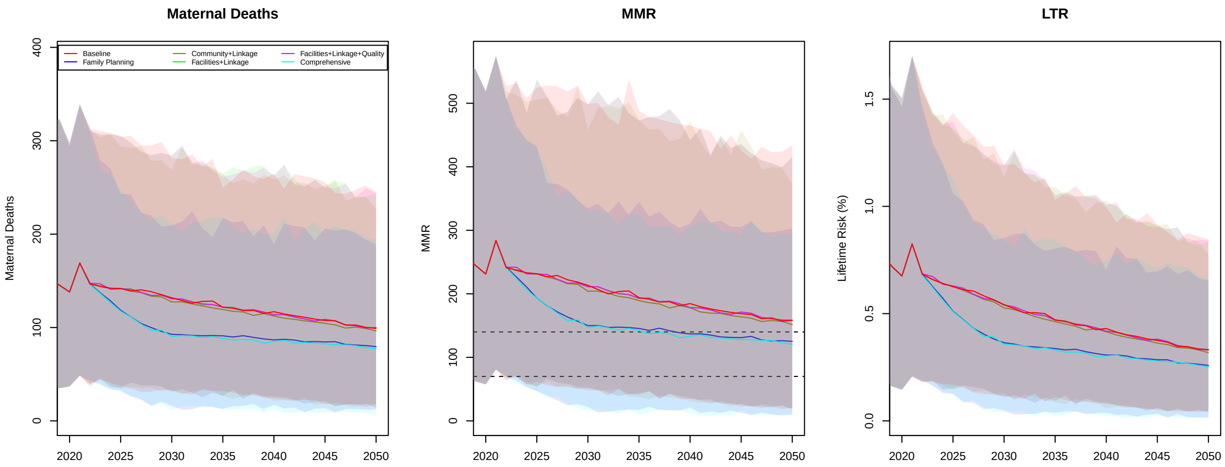

Brazil

ISO Code	Region	Area	Income Group
BRA	South America	Latin America and the Caribbean	Upper middle income

Projected Maternal Indicators in 2030 by Scenario

Scenario	Maternal Deaths	MMR	LTR
**Baseline**	2691 (1427-3978)	65 (29-124)	0.13 (0.06-0.22)
**Family Planning Interventions**			
Contraception	2497 (1293-4092)	56 (26-91)	0.12 (0.05-0.21)
Medical abortion	2690 (1427-3978)	65 (29-124)	0.13 (0.06-0.22)
**Community-Based Interventions**			
ANC	2659 (1392-4083)	64 (28-129)	0.13 (0.06-0.22)
SBA	2686 (1427-4097)	65 (28-127)	0.13 (0.06-0.22)
**Facility-Based Interventions**			
Facility births	2562 (1310-3910)	61 (25-121)	0.13 (0.05-0.22)
nonEmOC services	2682 (1418-4085)	65 (28-126)	0.13 (0.06-0.21)
bEmOC services	2699 (1417-4058)	65 (28-133)	0.13 (0.06-0.22)
cEmOC services	2685 (1422-4142)	65 (29-129)	0.13 (0.06-0.22)
**System-Relevant Interventions**			
Quality of care	2541 (1308-4031)	61 (25-124)	0.13 (0.06-0.21)
Referral	2697 (1450-4223)	65 (28-128)	0.13 (0.07-0.23)
Transport	2650 (1378-4199)	64 (27-128)	0.13 (0.06-0.22)
Targeted transfers	2684 (1438-4108)	65 (28-126)	0.13 (0.06-0.22)
**Integrated Strategies**			
Family Planning	2497 (1293-4089)	56 (26-91)	0.12 (0.05-0.21)
Community + Linkages	2591 (1373-4011)	63 (27-124)	0.13 (0.06-0.21)
Facilities + Linkages	2524 (1157-4034)	61 (23-122)	0.12 (0.05-0.21)
Facilities + Linkages + Quality	2366 (1108-3788)	57 (20-118)	0.12 (0.05-0.2)
Comprehensive	2166 (968-3785)	47 (18-82)	0.1 (0.04-0.19)

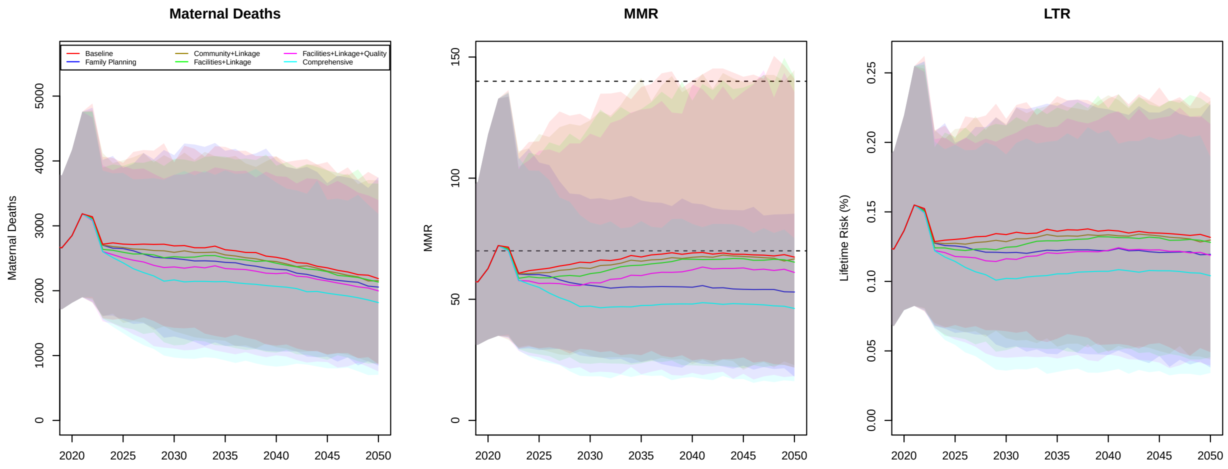

Brunei Darussalam

ISO Code	Region	Area	Income Group
BRN	South-Eastern Asia	Asia	High income

Projected Maternal Indicators in 2030 by Scenario

Scenario	Maternal Deaths	MMR	LTR
**Baseline**	2 (0-11)	25 (0-166)	0.04 (0-0.3)
**Family Planning Interventions**			
Contraception	2 (0-9)	23 (0-152)	0.04 (0-0.26)
Medical abortion	2 (0-11)	25 (0-153)	0.04 (0-0.28)
**Community-Based Interventions**			
ANC	2 (0-11)	25 (0-163)	0.04 (0-0.29)
SBA	2 (0-10)	23 (0-152)	0.04 (0-0.27)
**Facility-Based Interventions**			
Facility births	2 (0-12)	23 (0-167)	0.04 (0-0.31)
nonEmOC services	2 (0-11)	25 (0-162)	0.04 (0-0.29)
bEmOC services	2 (0-11)	25 (0-166)	0.04 (0-0.3)
cEmOC services	2 (0-9)	24 (0-145)	0.04 (0-0.26)
**System-Relevant Interventions**			
Quality of care	2 (0-10)	22 (0-160)	0.04 (0-0.27)
Referral	2 (0-11)	25 (0-159)	0.04 (0-0.27)
Transport	2 (0-11)	25 (0-167)	0.04 (0-0.3)
Targeted transfers	2 (0-11)	26 (0-167)	0.04 (0-0.3)
**Integrated Strategies**			
Family Planning	2 (0-11)	23 (0-153)	0.04 (0-0.26)
Community + Linkages	2 (0-12)	25 (0-169)	0.04 (0-0.3)
Facilities + Linkages	2 (0-9)	25 (0-151)	0.04 (0-0.27)
Facilities + Linkages + Quality	1 (0-9)	19 (0-123)	0.03 (0-0.24)
Comprehensive	2 (0-11)	21 (0-148)	0.04 (0-0.26)

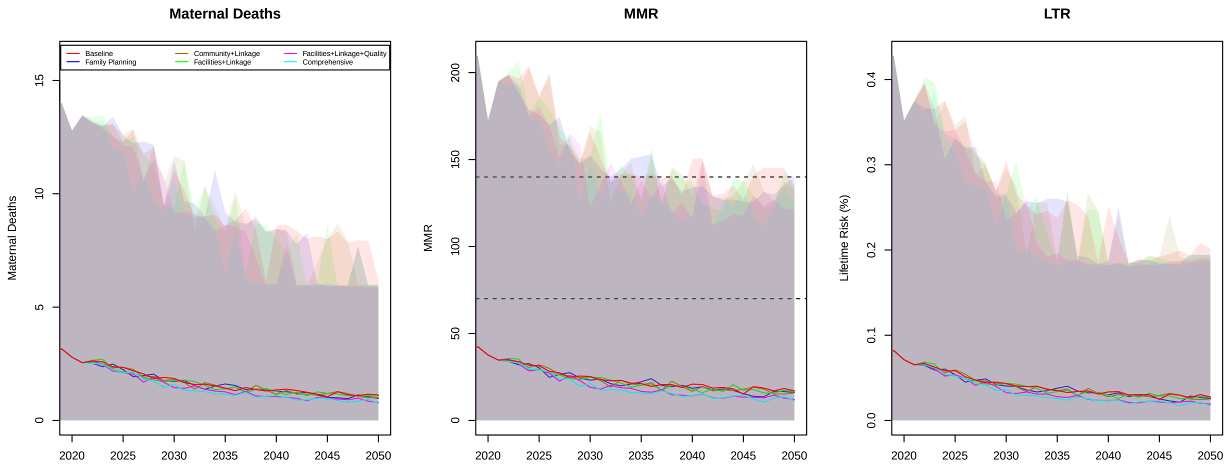

Bulgaria

ISO Code	Region	Area	Income Group
BGR	Eastern Europe	Europe	Upper middle income

Projected Maternal Indicators in 2030 by Scenario

Scenario	Maternal Deaths	MMR	LTR
**Baseline**	34 (5-81)	46 (0-129)	0.06 (0-0.18)
**Family Planning Interventions**			
Contraception	34 (5-83)	45 (0-128)	0.06 (0-0.18)
Medical abortion	34 (5-82)	46 (0-130)	0.06 (0-0.19)
**Community-Based Interventions**			
ANC	34 (5-83)	45 (0-132)	0.06 (0-0.18)
SBA	35 (5-81)	46 (0-129)	0.06 (0-0.18)
**Facility-Based Interventions**			
Facility births	35 (5-81)	46 (0-128)	0.06 (0-0.19)
nonEmOC services	34 (5-81)	46 (0-129)	0.06 (0-0.18)
bEmOC services	34 (5-82)	46 (0-128)	0.06 (0-0.18)
cEmOC services	34 (5-81)	45 (0-128)	0.06 (0-0.18)
**System-Relevant Interventions**			
Quality of care	35 (5-81)	45 (0-128)	0.06 (0-0.18)
Referral	34 (5-82)	46 (0-129)	0.06 (0-0.18)
Transport	34 (5-82)	46 (0-127)	0.06 (0-0.18)
Targeted transfers	34 (5-81)	46 (0-125)	0.06 (0-0.18)
**Integrated Strategies**			
Family Planning	34 (5-83)	45 (0-133)	0.06 (0-0.18)
Community + Linkages	33 (5-84)	45 (0-130)	0.06 (0-0.18)
Facilities + Linkages	33 (5-77)	45 (0-121)	0.06 (0-0.18)
Facilities + Linkages + Quality	34 (5-77)	45 (0-122)	0.06 (0-0.18)
Comprehensive	33 (5-81)	44 (0-134)	0.06 (0-0.19)

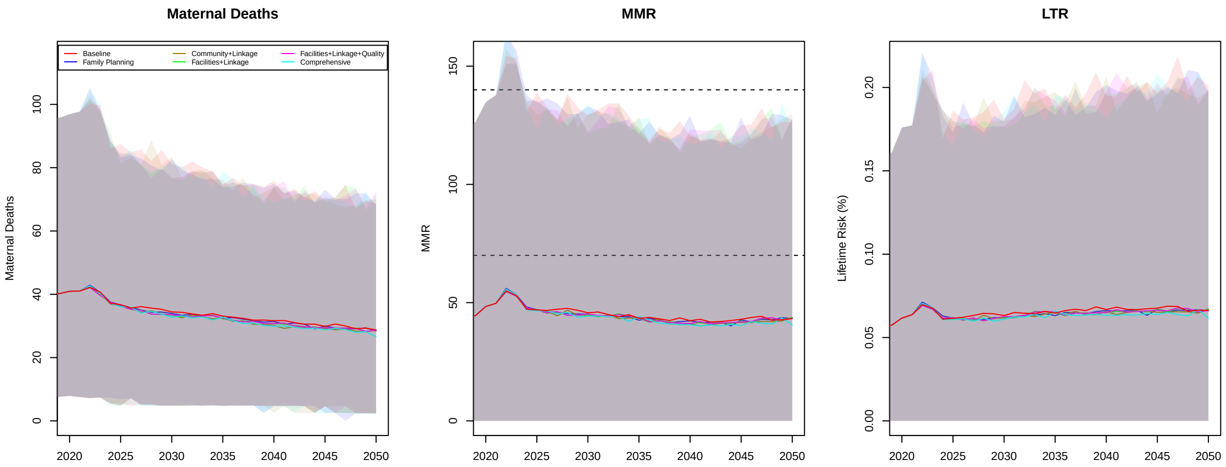

Burkina Faso

ISO Code	Region	Area	Income Group
BFA	Western Africa	Africa	Low income

Projected Maternal Indicators in 2030 by Scenario

Scenario	Maternal Deaths	MMR	LTR
**Baseline**	4062 (2748-5876)	286 (207-386)	1.51 (0.99-2.12)
**Family Planning Interventions**			
Contraception	4072 (2774-5653)	287 (207-385)	1.52 (1.01-2.13)
Medical abortion	4076 (2668-5932)	287 (200-396)	1.51 (0.97-2.17)
**Community-Based Interventions**			
ANC	4014 (2685-5624)	282 (202-384)	1.48 (0.96-2.06)
SBA	4013 (2687-5751)	282 (198-380)	1.48 (0.95-2.08)
**Facility-Based Interventions**			
Facility births	3710 (2405-5247)	258 (182-352)	1.36 (0.83-1.9)
nonEmOC services	4051 (2719-5741)	284 (203-384)	1.5 (0.95-2.1)
bEmOC services	4043 (2695-5801)	285 (204-394)	1.5 (0.96-2.13)
cEmOC services	4016 (2670-5683)	283 (198-385)	1.49 (0.94-2.09)
**System-Relevant Interventions**			
Quality of care	1970 (1110-3162)	121 (60-212)	0.64 (0.3-1.15)
Referral	4007 (2698-5740)	282 (203-380)	1.48 (0.97-2.09)
Transport	3933 (2531-5538)	278 (194-379)	1.46 (0.93-2.06)
Targeted transfers	3981 (2720-5625)	280 (205-390)	1.47 (0.99-2.09)
**Integrated Strategies**			
Family Planning	4079 (2773-5659)	286 (204-391)	1.52 (1-2.15)
Community + Linkages	3638 (2347-5264)	254 (179-350)	1.33 (0.83-1.94)
Facilities + Linkages	3370 (2180-4765)	233 (160-322)	1.23 (0.77-1.75)
Facilities + Linkages + Quality	1125 (548-1999)	57 (20-123)	0.3 (0.09-0.69)
Comprehensive	1100 (526-2002)	54 (21-121)	0.28 (0.09-0.7)

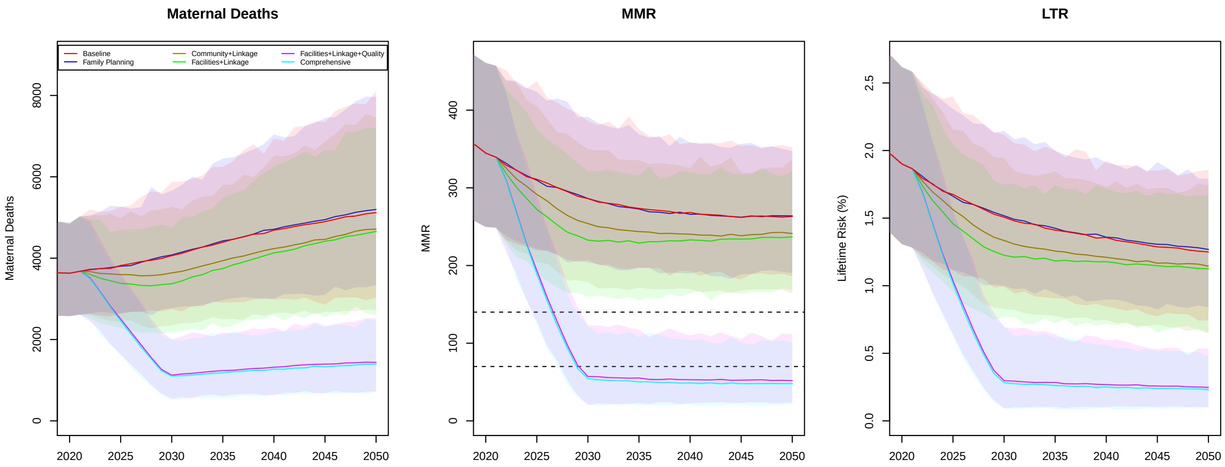

Burundi

ISO Code	Region	Area	Income Group
BDI	Eastern Africa	Africa	Low income

Projected Maternal Indicators in 2030 by Scenario

Scenario	Maternal Deaths	MMR	LTR
**Baseline**	2119 (1077-3363)	315 (143-502)	1.44 (0.67-2.33)
**Family Planning Interventions**			
Contraception	1728 (699-2977)	296 (134-484)	1.11 (0.38-2)
Medical abortion	2081 (1117-3365)	309 (141-497)	1.42 (0.62-2.36)
**Community-Based Interventions**			
ANC	2077 (1068-3280)	308 (137-488)	1.41 (0.63-2.31)
SBA	1939 (1012-3170)	287 (128-474)	1.31 (0.6-2.18)
**Facility-Based Interventions**			
Facility births	1052 (365-1943)	139 (35-291)	0.62 (0.13-1.28)
nonEmOC services	2099 (1035-3339)	313 (149-498)	1.43 (0.66-2.36)
bEmOC services	2114 (1108-3327)	315 (147-503)	1.44 (0.67-2.33)
cEmOC services	2077 (1128-3308)	309 (139-487)	1.42 (0.69-2.31)
**System-Relevant Interventions**			
Quality of care	1852 (916-2912)	270 (112-432)	1.24 (0.55-2.08)
Referral	2005 (997-3265)	295 (140-477)	1.36 (0.63-2.22)
Transport	1763 (867-2892)	258 (119-439)	1.18 (0.51-2.07)
Targeted transfers	2090 (1091-3262)	311 (152-481)	1.43 (0.75-2.26)
**Integrated Strategies**			
Family Planning	1728 (680-3013)	296 (128-488)	1.12 (0.38-2.01)
Community + Linkages	1394 (617-2417)	199 (81-363)	0.9 (0.34-1.62)
Facilities + Linkages	866 (282-1690)	112 (23-258)	0.51 (0.07-1.19)
Facilities + Linkages + Quality	562 (147-1183)	60 (10-144)	0.27 (0-0.69)
Comprehensive	463 (71-1028)	56 (0-147)	0.19 (0-0.6)

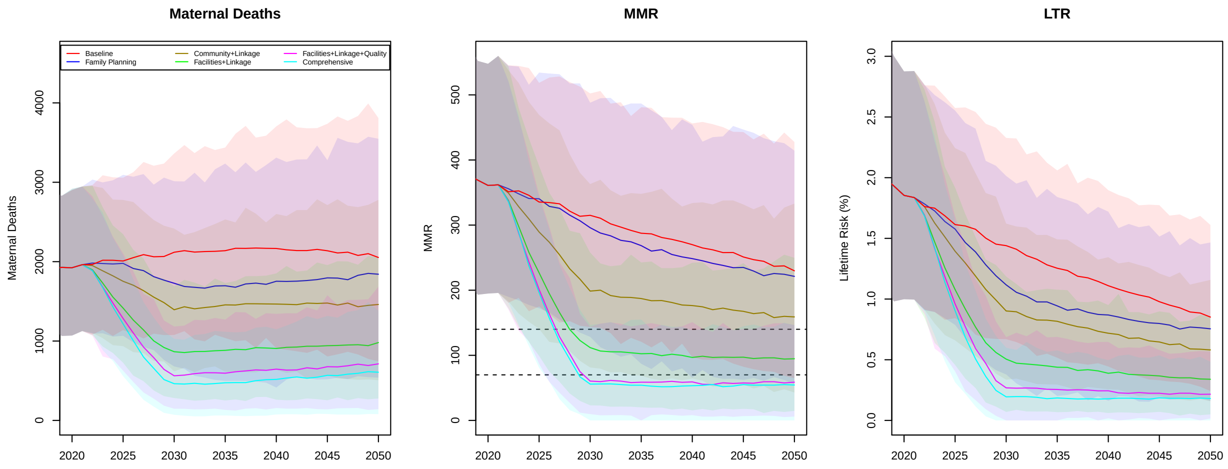

Cabo Verde

ISO Code	Region	Area	Income Group
CPV	Western Africa	Africa	Lower middle income

Projected Maternal Indicators in 2030 by Scenario

Scenario	Maternal Deaths	MMR	LTR
**Baseline**	27 (2-65)	234 (16-562)	0.45 (0-1.12)
**Family Planning Interventions**			
Contraception	28 (2-68)	221 (0-519)	0.46 (0-1.18)
Medical abortion	26 (2-65)	220 (0-559)	0.43 (0-1.1)
**Community-Based Interventions**			
ANC	27 (2-68)	227 (0-580)	0.44 (0-1.13)
SBA	27 (2-65)	230 (0-545)	0.44 (0-1.13)
**Facility-Based Interventions**			
Facility births	26 (2-62)	223 (0-542)	0.43 (0-1.08)
nonEmOC services	27 (2-64)	230 (0-556)	0.44 (0-1.11)
bEmOC services	27 (3-65)	234 (0-593)	0.45 (0-1.12)
cEmOC services	26 (3-65)	219 (20-569)	0.42 (0.03-1.1)
**System-Relevant Interventions**			
Quality of care	13 (0-41)	106 (0-366)	0.2 (0-0.69)
Referral	27 (2-66)	235 (17-588)	0.45 (0-1.14)
Transport	27 (2-64)	229 (0-607)	0.44 (0-1.09)
Targeted transfers	27 (2-64)	227 (0-570)	0.44 (0-1.14)
**Integrated Strategies**			
Family Planning	27 (2-69)	218 (12-538)	0.45 (0-1.19)
Community + Linkages	25 (0-62)	211 (0-545)	0.41 (0-1.05)
Facilities + Linkages	24 (0-61)	206 (0-566)	0.4 (0-1.06)
Facilities + Linkages + Quality	10 (0-36)	75 (0-316)	0.14 (0-0.58)
Comprehensive	8 (0-30)	51 (0-203)	0.11 (0-0.45)

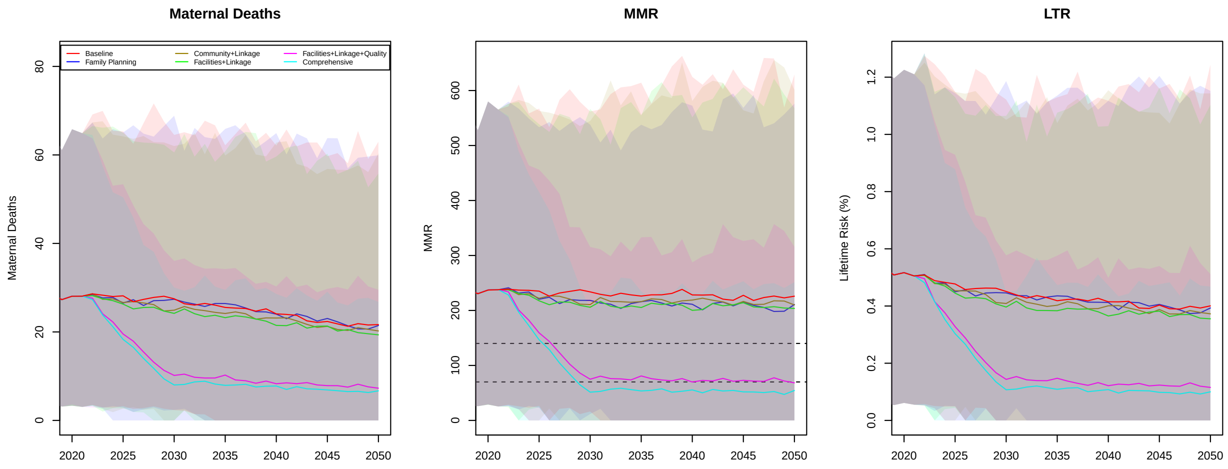

Cambodia

ISO Code	Region	Area	Income Group
KHM	South-Eastern Asia	Asia	Lower middle income

Projected Maternal Indicators in 2030 by Scenario

Scenario	Maternal Deaths	MMR	LTR
**Baseline**	211 (18-528)	52 (0-165)	0.09 (0-0.27)
**Family Planning Interventions**			
Contraception	225 (24-546)	42 (0-124)	0.09 (0-0.28)
Medical abortion	213 (23-519)	53 (0-159)	0.09 (0-0.28)
**Community-Based Interventions**			
ANC	215 (22-543)	51 (0-152)	0.09 (0-0.27)
SBA	213 (0-563)	53 (0-167)	0.09 (0-0.3)
**Facility-Based Interventions**			
Facility births	220 (17-527)	55 (0-175)	0.09 (0-0.28)
nonEmOC services	211 (23-528)	51 (0-160)	0.09 (0-0.26)
bEmOC services	215 (0-574)	50 (0-152)	0.09 (0-0.28)
cEmOC services	209 (17-571)	47 (0-161)	0.08 (0-0.26)
**System-Relevant Interventions**			
Quality of care	203 (21-550)	46 (0-148)	0.08 (0-0.25)
Referral	214 (17-532)	51 (0-156)	0.09 (0-0.28)
Transport	202 (19-501)	50 (0-154)	0.09 (0-0.26)
Targeted transfers	206 (0-539)	49 (0-148)	0.09 (0-0.27)
**Integrated Strategies**			
Family Planning	229 (0-544)	43 (0-128)	0.09 (0-0.28)
Community + Linkages	188 (0-465)	45 (0-138)	0.08 (0-0.24)
Facilities + Linkages	176 (0-457)	38 (0-134)	0.07 (0-0.23)
Facilities + Linkages + Quality	166 (0-475)	35 (0-128)	0.06 (0-0.22)
Comprehensive	164 (0-428)	27 (0-95)	0.06 (0-0.21)

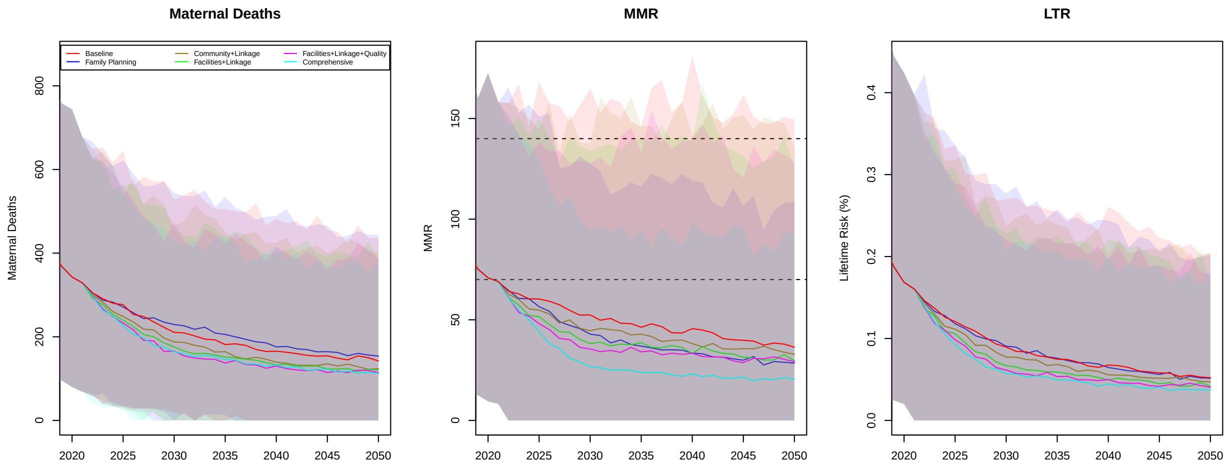

Cameroon

ISO Code	Region	Area	Income Group
CMR	Middle Africa	Africa	Lower middle income

Projected Maternal Indicators in 2030 by Scenario

Scenario	Maternal Deaths	MMR	LTR
**Baseline**	4731 (2513-7397)	312 (149-448)	1.64 (0.84-2.56)
**Family Planning Interventions**			
Contraception	4374 (2245-6815)	294 (136-441)	1.5 (0.71-2.32)
Medical abortion	4616 (2436-7132)	302 (141-449)	1.6 (0.78-2.44)
**Community-Based Interventions**			
ANC	4641 (2479-7203)	305 (147-427)	1.61 (0.81-2.44)
SBA	4498 (2476-6879)	296 (148-423)	1.56 (0.83-2.35)
**Facility-Based Interventions**			
Facility births	4037 (2206-6118)	263 (142-401)	1.4 (0.71-2.13)
nonEmOC services	4714 (2486-7334)	311 (147-446)	1.64 (0.84-2.52)
bEmOC services	4718 (2489-7428)	311 (148-449)	1.64 (0.83-2.55)
cEmOC services	4665 (2406-7249)	307 (143-447)	1.62 (0.81-2.51)
**System-Relevant Interventions**			
Quality of care	4063 (1946-6900)	264 (110-402)	1.42 (0.64-2.38)
Referral	4716 (2486-7364)	311 (149-448)	1.64 (0.83-2.6)
Transport	4567 (2461-6992)	302 (150-427)	1.59 (0.83-2.39)
Targeted transfers	4726 (2438-7515)	311 (148-449)	1.64 (0.83-2.52)
**Integrated Strategies**			
Family Planning	4326 (2249-6766)	291 (134-431)	1.48 (0.69-2.29)
Community + Linkages	4293 (2333-6589)	283 (144-415)	1.49 (0.76-2.32)
Facilities + Linkages	3941 (2142-5875)	258 (134-393)	1.37 (0.69-2.07)
Facilities + Linkages + Quality	3227 (1618-5311)	207 (91-322)	1.14 (0.51-1.87)
Comprehensive	2920 (1382-4761)	191 (79-294)	1.02 (0.4-1.69)

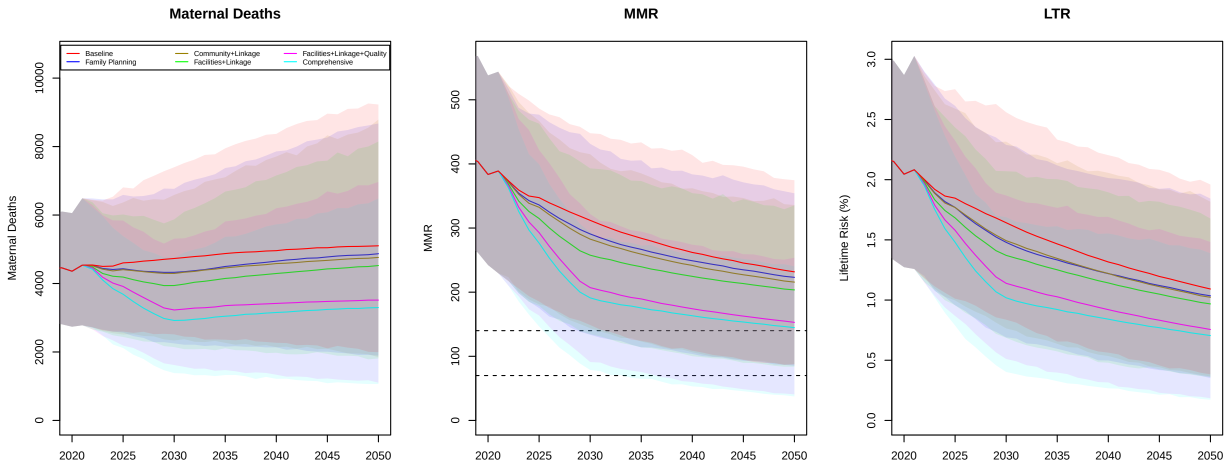

Canada

ISO Code	Region	Area	Income Group
CAN	Northern America	Northern America	High income

Projected Maternal Indicators in 2030 by Scenario

Scenario	Maternal Deaths	MMR	LTR
**Baseline**	105 (27-218)	13 (0-29)	0.02 (0-0.05)
**Family Planning Interventions**			
Contraception	102 (27-206)	13 (0-30)	0.02 (0-0.05)
Medical abortion	102 (31-196)	13 (0-28)	0.02 (0-0.05)
**Community-Based Interventions**			
ANC	101 (27-199)	12 (0-27)	0.02 (0-0.05)
SBA	105 (27-217)	13 (0-29)	0.02 (0-0.05)
**Facility-Based Interventions**			
Facility births	105 (27-218)	13 (0-29)	0.02 (0-0.05)
nonEmOC services	105 (27-217)	13 (0-29)	0.02 (0-0.05)
bEmOC services	105 (27-218)	13 (0-29)	0.02 (0-0.05)
cEmOC services	103 (27-209)	13 (0-29)	0.02 (0-0.05)
**System-Relevant Interventions**			
Quality of care	100 (25-203)	12 (0-29)	0.02 (0-0.05)
Referral	105 (27-218)	13 (0-29)	0.02 (0-0.05)
Transport	103 (28-206)	13 (0-29)	0.02 (0-0.05)
Targeted transfers	105 (27-218)	13 (0-30)	0.02 (0-0.05)
**Integrated Strategies**			
Family Planning	103 (27-214)	13 (0-29)	0.02 (0-0.05)
Community + Linkages	103 (27-203)	13 (0-28)	0.02 (0-0.05)
Facilities + Linkages	103 (29-210)	13 (0-29)	0.02 (0-0.05)
Facilities + Linkages + Quality	96 (26-193)	12 (0-28)	0.02 (0-0.05)
Comprehensive	95 (16-188)	11 (0-27)	0.02 (0-0.04)

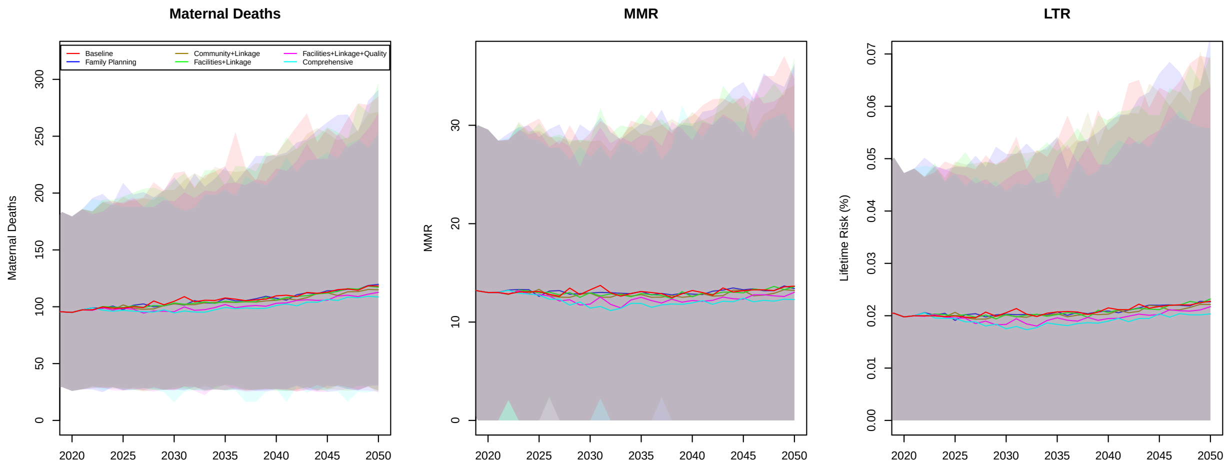

Cayman Islands

ISO Code	Region	Area	Income Group
CYM	Caribbean	Latin America and the Caribbean	High income

Projected Maternal Indicators in 2030 by Scenario

Scenario	Maternal Deaths	MMR	LTR
**Baseline**	0 (0-2)	26 (0-240)	0.05 (0-0.45)
**Family Planning Interventions**			
Contraception	0 (0-2)	26 (0-239)	0.05 (0-0.43)
Medical abortion	0 (0-2)	26 (0-239)	0.05 (0-0.49)
**Community-Based Interventions**			
ANC	0 (0-2)	26 (0-240)	0.05 (0-0.45)
SBA	0 (0-2)	26 (0-240)	0.05 (0-0.45)
**Facility-Based Interventions**			
Facility births	0 (0-2)	25 (0-239)	0.04 (0-0.44)
nonEmOC services	0 (0-2)	26 (0-240)	0.05 (0-0.45)
bEmOC services	0 (0-2)	26 (0-240)	0.05 (0-0.45)
cEmOC services	0 (0-2)	26 (0-241)	0.05 (0-0.46)
**System-Relevant Interventions**			
Quality of care	0 (0-2)	26 (0-241)	0.05 (0-0.47)
Referral	0 (0-2)	26 (0-240)	0.05 (0-0.45)
Transport	0 (0-2)	28 (0-248)	0.05 (0-0.49)
Targeted transfers	0 (0-2)	26 (0-240)	0.05 (0-0.45)
**Integrated Strategies**			
Family Planning	0 (0-2)	25 (0-235)	0.04 (0-0.43)
Community + Linkages	0 (0-2)	28 (0-248)	0.05 (0-0.49)
Facilities + Linkages	0 (0-2)	29 (0-248)	0.05 (0-0.49)
Facilities + Linkages + Quality	0 (0-4)	29 (0-254)	0.05 (0-0.49)
Comprehensive	0 (0-4)	29 (0-257)	0.05 (0-0.51)

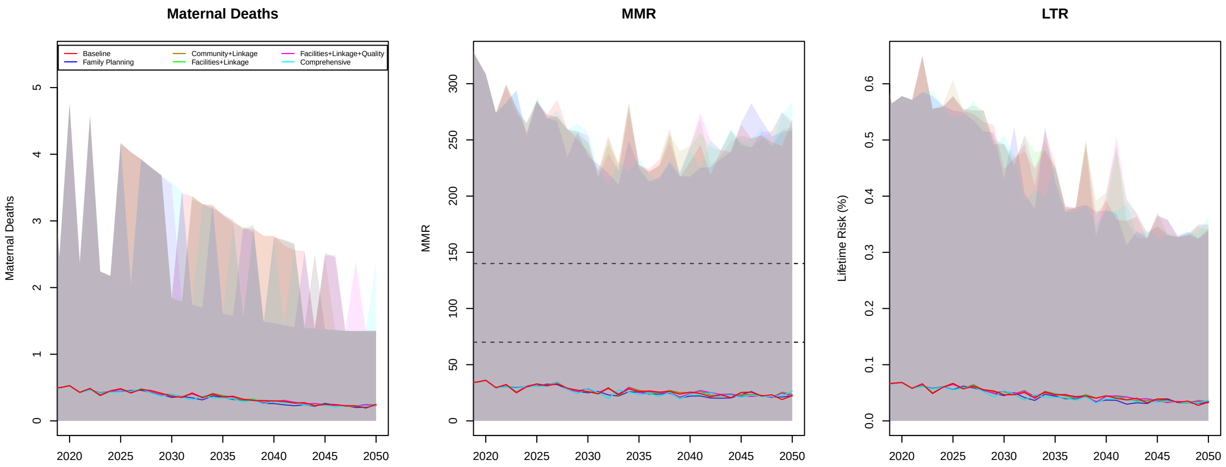

Central African Republic

ISO Code	Region	Area	Income Group
CAF	Middle Africa	Africa	Low income

Projected Maternal Indicators in 2030 by Scenario

Scenario	Maternal Deaths	MMR	LTR
**Baseline**	1363 (524-2438)	471 (214-881)	2.28 (0.82-4.22)
**Family Planning Interventions**			
Contraception	1188 (457-1988)	432 (182-774)	1.96 (0.69-3.43)
Medical abortion	1287 (530-2242)	438 (198-804)	2.13 (0.75-3.88)
**Community-Based Interventions**			
ANC	1295 (518-2281)	444 (189-832)	2.16 (0.79-4.03)
SBA	1310 (513-2352)	451 (200-849)	2.2 (0.82-4.1)
**Facility-Based Interventions**			
Facility births	1007 (342-1954)	334 (123-651)	1.63 (0.48-3.36)
nonEmOC services	1354 (519-2515)	467 (211-893)	2.27 (0.81-4.2)
bEmOC services	1347 (520-2359)	465 (206-866)	2.26 (0.81-4.08)
cEmOC services	1315 (478-2435)	452 (182-863)	2.2 (0.71-4.13)
**System-Relevant Interventions**			
Quality of care	940 (249-2042)	316 (73-713)	1.55 (0.31-3.53)
Referral	1336 (556-2337)	461 (205-853)	2.23 (0.89-4.08)
Transport	1247 (494-2269)	429 (182-788)	2.09 (0.72-3.94)
Targeted transfers	1351 (524-2380)	465 (209-884)	2.26 (0.81-4.16)
**Integrated Strategies**			
Family Planning	1159 (442-1991)	419 (190-807)	1.91 (0.63-3.4)
Community + Linkages	1126 (417-2095)	382 (158-726)	1.86 (0.6-3.63)
Facilities + Linkages	943 (316-1940)	312 (115-652)	1.53 (0.45-3.4)
Facilities + Linkages + Quality	466 (113-1157)	138 (22-375)	0.69 (0.07-2)
Comprehensive	359 (68-892)	108 (8-303)	0.5 (0-1.5)

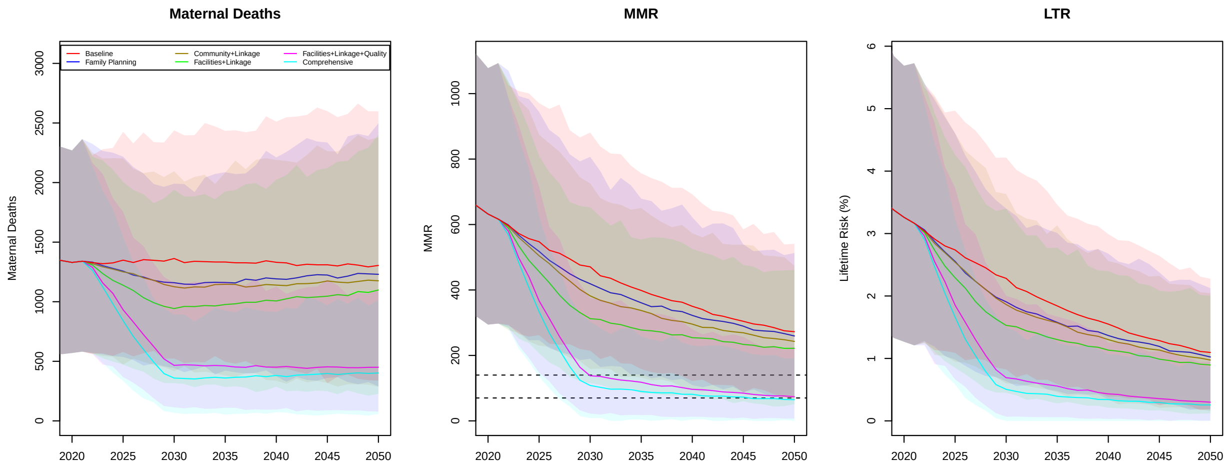

Chad

ISO Code	Region	Area	Income Group
TCD	Middle Africa	Africa	Low income

Projected Maternal Indicators in 2030 by Scenario

Scenario	Maternal Deaths	MMR	LTR
**Baseline**	12194 (8246-16798)	980 (693-1334)	6 (3.92-8.33)
**Family Planning Interventions**			
Contraception	11028 (6781-15410)	939 (668-1251)	5.32 (3.02-7.51)
Medical abortion	11695 (7953-15660)	937 (668-1248)	5.69 (3.57-7.8)
**Community-Based Interventions**			
ANC	11218 (7819-15598)	898 (652-1201)	5.45 (3.74-7.65)
SBA	11925 (8223-15943)	959 (692-1303)	5.89 (3.93-8.05)
**Facility-Based Interventions**			
Facility births	6262 (3697-9665)	482 (280-764)	2.99 (1.54-4.96)
nonEmOC services	12118 (8369-16748)	973 (690-1338)	5.97 (3.96-8.56)
bEmOC services	12091 (8375-16575)	973 (696-1323)	5.97 (4.04-8.33)
cEmOC services	12084 (8205-16847)	971 (696-1323)	5.96 (3.98-8.4)
**System-Relevant Interventions**			
Quality of care	10996 (7238-15627)	880 (606-1258)	5.42 (3.45-7.86)
Referral	11843 (8188-16706)	951 (662-1329)	5.82 (3.84-8.29)
Transport	10406 (7015-14475)	831 (590-1161)	5.08 (3.25-7.21)
Targeted transfers	12159 (8224-16919)	977 (693-1340)	5.98 (3.93-8.35)
**Integrated Strategies**			
Family Planning	10873 (5960-15548)	924 (643-1259)	5.23 (2.64-7.6)
Community + Linkages	8510 (5666-12260)	672 (454-946)	4.08 (2.51-6.21)
Facilities + Linkages	5791 (3127-9273)	446 (241-756)	2.76 (1.34-4.59)
Facilities + Linkages + Quality	2701 (1050-5825)	191 (60-488)	1.17 (0.23-3.08)
Comprehensive	1993 (748-4452)	142 (45-345)	0.73 (0.11-2.23)

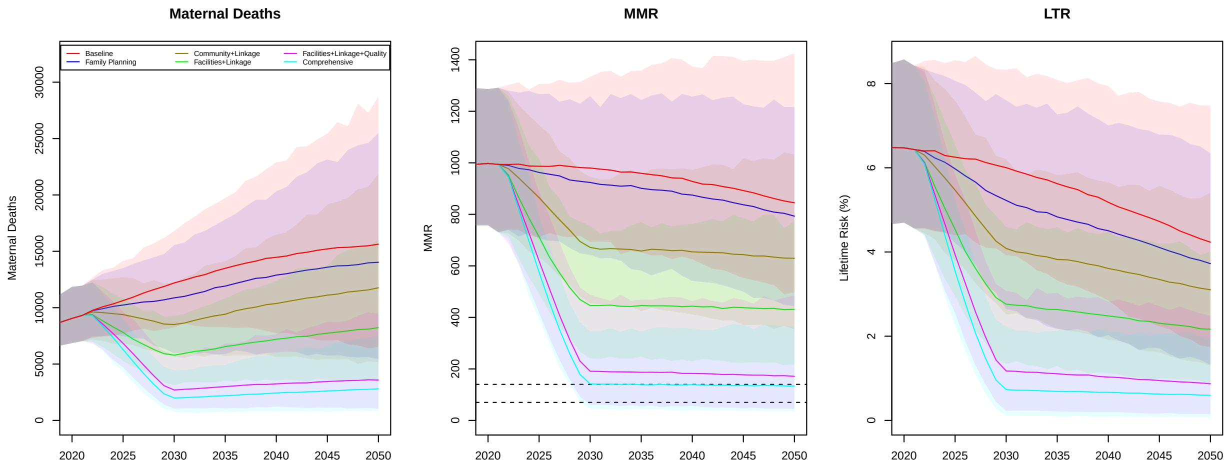

Chile

ISO Code	Region	Area	Income Group
CHL	South America	Latin America and the Caribbean	High income

Projected Maternal Indicators in 2030 by Scenario

Scenario	Maternal Deaths	MMR	LTR
**Baseline**	162 (63-272)	48 (18-91)	0.1 (0.03-0.17)
**Family Planning Interventions**			
Contraception	150 (53-271)	46 (17-85)	0.09 (0.03-0.17)
Medical abortion	162 (63-273)	48 (18-92)	0.1 (0.03-0.17)
**Community-Based Interventions**			
ANC	162 (67-282)	48 (18-90)	0.1 (0.03-0.18)
SBA	159 (58-274)	47 (16-90)	0.1 (0.03-0.17)
**Facility-Based Interventions**			
Facility births	157 (60-267)	46 (16-88)	0.09 (0.03-0.17)
nonEmOC services	161 (66-272)	48 (18-92)	0.1 (0.03-0.17)
bEmOC services	162 (63-272)	48 (18-90)	0.1 (0.03-0.17)
cEmOC services	160 (58-273)	47 (17-89)	0.1 (0.03-0.18)
**System-Relevant Interventions**			
Quality of care	160 (62-273)	47 (18-90)	0.1 (0.03-0.17)
Referral	162 (63-272)	48 (17-92)	0.1 (0.03-0.17)
Transport	159 (60-271)	47 (17-90)	0.1 (0.03-0.18)
Targeted transfers	163 (68-274)	48 (19-91)	0.1 (0.03-0.17)
**Integrated Strategies**			
Family Planning	150 (53-271)	46 (17-85)	0.09 (0.03-0.17)
Community + Linkages	157 (65-277)	47 (18-87)	0.09 (0.03-0.17)
Facilities + Linkages	158 (61-281)	47 (16-84)	0.1 (0.03-0.18)
Facilities + Linkages + Quality	158 (59-279)	47 (16-83)	0.1 (0.03-0.18)
Comprehensive	142 (47-261)	43 (15-78)	0.09 (0.02-0.17)

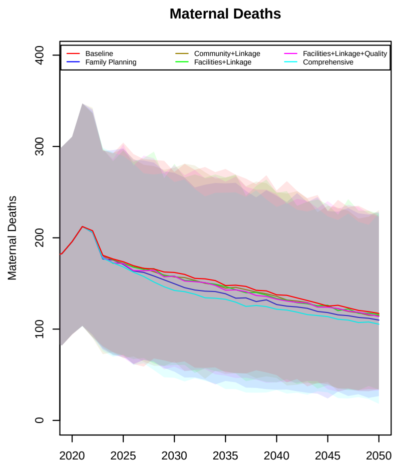

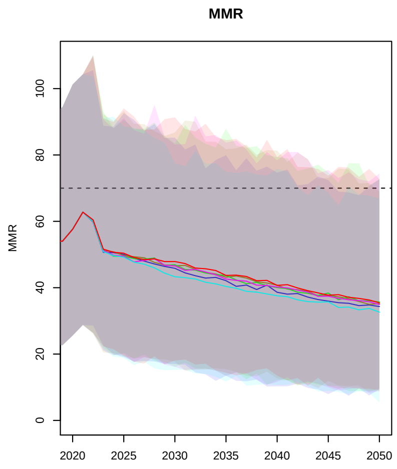

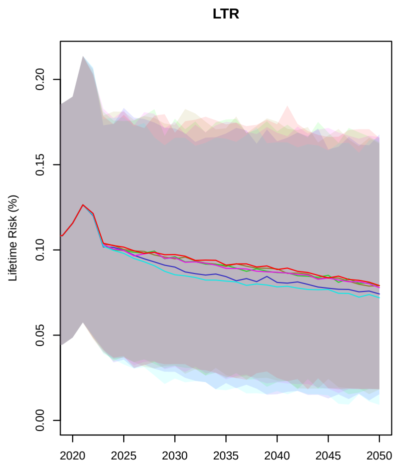

China

ISO Code	Region	Area	Income Group
CHN	Eastern Asia	Asia	Upper middle income

Projected Maternal Indicators in 2030 by Scenario

Scenario	Maternal Deaths	MMR	LTR
**Baseline**	4947 (1266-12883)	20 (2-62)	0.03 (0-0.12)
**Family Planning Interventions**			
Contraception	4090 (999-10683)	14 (1-48)	0.02 (0-0.09)
Medical abortion	4884 (1202-12821)	19 (2-61)	0.03 (0-0.12)
**Community-Based Interventions**			
ANC	4842 (1165-12246)	19 (2-55)	0.03 (0-0.1)
SBA	4926 (1228-13300)	19 (2-62)	0.03 (0-0.11)
**Facility-Based Interventions**			
Facility births	4645 (1113-11288)	18 (1-55)	0.03 (0-0.09)
nonEmOC services	4977 (1286-13486)	19 (2-64)	0.03 (0-0.12)
bEmOC services	4994 (1298-12997)	20 (2-62)	0.03 (0-0.12)
cEmOC services	4954 (1303-12983)	19 (2-60)	0.03 (0-0.12)
**System-Relevant Interventions**			
Quality of care	4951 (1279-13377)	20 (2-62)	0.03 (0-0.12)
Referral	4940 (1292-12809)	19 (2-63)	0.03 (0-0.12)
Transport	4796 (1243-13011)	19 (2-62)	0.03 (0-0.11)
Targeted transfers	4903 (1294-13206)	19 (2-62)	0.03 (0-0.11)
**Integrated Strategies**			
Family Planning	4092 (1101-10215)	14 (1-46)	0.02 (0-0.08)
Community + Linkages	4626 (1161-12228)	18 (2-53)	0.03 (0-0.1)
Facilities + Linkages	4483 (1174-11247)	17 (1-54)	0.03 (0-0.09)
Facilities + Linkages + Quality	4502 (1175-11325)	17 (1-56)	0.03 (0-0.09)
Comprehensive	3446 (950-7051)	11 (1-30)	0.02 (0-0.05)

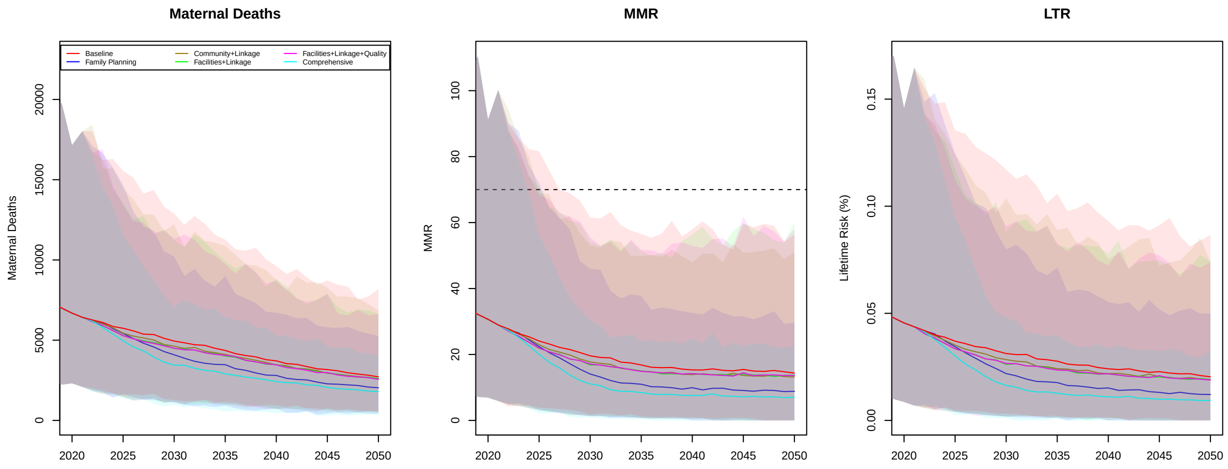

Colombia

ISO Code	Region	Area	Income Group
COL	South America	Latin America and the Caribbean	Upper middle income

Projected Maternal Indicators in 2030 by Scenario

Scenario	Maternal Deaths	MMR	LTR
**Baseline**	418 (111-832)	39 (9-77)	0.07 (0.01-0.16)
**Family Planning Interventions**			
Contraception	369 (95-758)	38 (9-80)	0.06 (0.01-0.15)
Medical abortion	418 (111-832)	39 (9-77)	0.07 (0.01-0.16)
**Community-Based Interventions**			
ANC	412 (123-797)	38 (9-80)	0.07 (0.01-0.16)
SBA	396 (114-806)	36 (7-76)	0.07 (0.01-0.16)
**Facility-Based Interventions**			
Facility births	327 (70-727)	27 (4-64)	0.05 (0.01-0.14)
nonEmOC services	420 (115-829)	39 (10-77)	0.07 (0.02-0.16)
bEmOC services	419 (128-829)	39 (9-79)	0.07 (0.01-0.16)
cEmOC services	413 (118-817)	38 (11-81)	0.07 (0.02-0.16)
**System-Relevant Interventions**			
Quality of care	406 (112-812)	38 (8-75)	0.07 (0.01-0.15)
Referral	421 (118-849)	39 (10-77)	0.07 (0.02-0.16)
Transport	379 (99-748)	35 (7-72)	0.07 (0.01-0.14)
Targeted transfers	426 (111-846)	40 (10-78)	0.07 (0.01-0.16)
**Integrated Strategies**			
Family Planning	368 (95-758)	38 (9-80)	0.06 (0.01-0.15)
Community + Linkages	355 (95-741)	32 (7-69)	0.06 (0.01-0.14)
Facilities + Linkages	310 (70-662)	26 (2-59)	0.05 (0-0.12)
Facilities + Linkages + Quality	297 (64-667)	24 (2-56)	0.05 (0-0.13)
Comprehensive	258 (47-595)	23 (0-58)	0.04 (0-0.11)

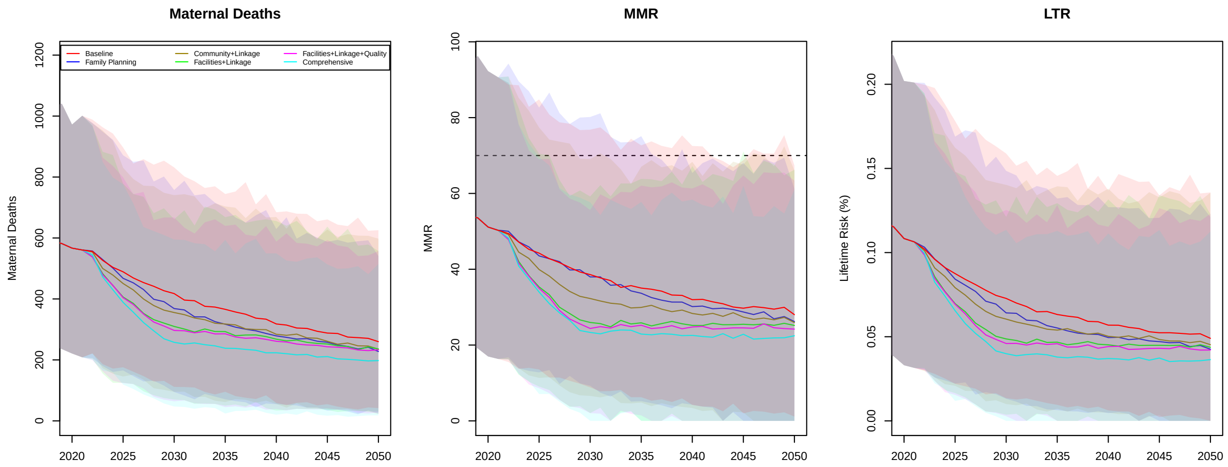

Comoros

ISO Code	Region	Area	Income Group
COM	Eastern Africa	Africa	Low income

Projected Maternal Indicators in 2030 by Scenario

Scenario	Maternal Deaths	MMR	LTR
**Baseline**	36 (7-82)	98 (10-249)	0.35 (0.03-0.97)
**Family Planning Interventions**			
Contraception	34 (9-74)	74 (12-189)	0.3 (0.04-0.8)
Medical abortion	31 (5-68)	76 (7-178)	0.27 (0-0.7)
**Community-Based Interventions**			
ANC	36 (8-83)	96 (9-246)	0.34 (0-0.95)
SBA	36 (7-88)	96 (12-256)	0.34 (0.03-1)
**Facility-Based Interventions**			
Facility births	33 (5-75)	86 (0-230)	0.3 (0-0.85)
nonEmOC services	36 (6-85)	98 (11-255)	0.35 (0.03-0.97)
bEmOC services	36 (7-83)	99 (0-251)	0.35 (0-0.99)
cEmOC services	33 (5-90)	89 (0-256)	0.31 (0-1)
**System-Relevant Interventions**			
Quality of care	34 (7-83)	91 (14-251)	0.32 (0.03-0.9)
Referral	36 (9-89)	98 (10-253)	0.35 (0.03-1)
Transport	33 (6-77)	93 (8-244)	0.33 (0-0.89)
Targeted transfers	36 (7-86)	99 (0-255)	0.35 (0-1)
**Integrated Strategies**			
Family Planning	32 (7-72)	68 (8-168)	0.28 (0-0.72)
Community + Linkages	32 (5-74)	87 (0-248)	0.31 (0-0.92)
Facilities + Linkages	27 (4-75)	72 (0-226)	0.25 (0-0.79)
Facilities + Linkages + Quality	26 (3-67)	66 (0-221)	0.23 (0-0.77)
Comprehensive	21 (4-47)	36 (0-95)	0.14 (0-0.43)

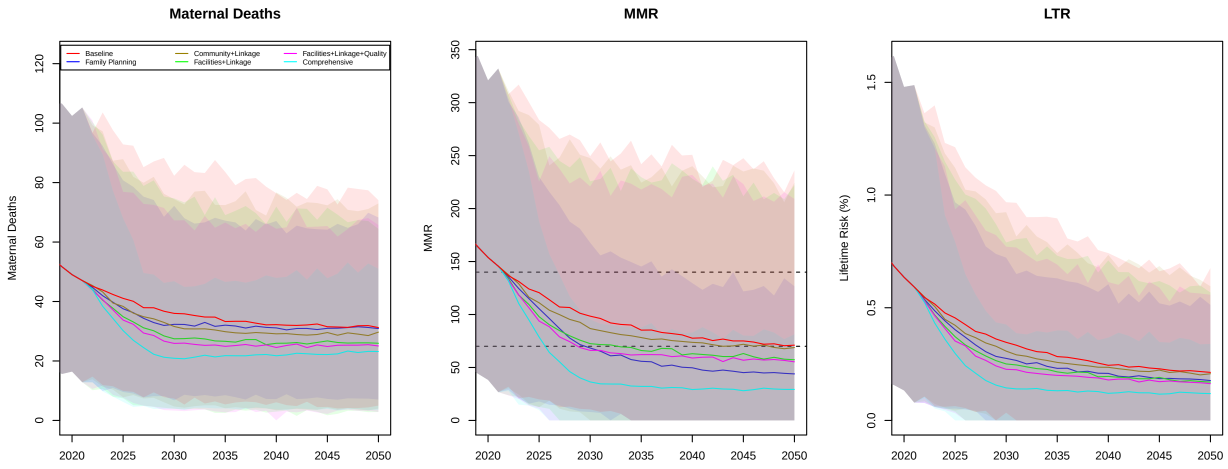

Congo

ISO Code	Region	Area	Income Group
COG	Middle Africa	Africa	Lower middle income

Projected Maternal Indicators in 2030 by Scenario

Scenario	Maternal Deaths	MMR	LTR
**Baseline**	586 (234-1083)	228 (89-399)	1.03 (0.39-1.97)
**Family Planning Interventions**			
Contraception	535 (205-1018)	215 (87-389)	0.94 (0.32-1.85)
Medical abortion	568 (228-1104)	218 (83-392)	1 (0.36-2.01)
**Community-Based Interventions**			
ANC	585 (245-1077)	228 (93-399)	1.03 (0.4-2.03)
SBA	591 (243-1102)	230 (90-393)	1.05 (0.39-2.04)
**Facility-Based Interventions**			
Facility births	582 (242-1113)	226 (88-406)	1.03 (0.38-1.99)
nonEmOC services	590 (232-1068)	229 (89-405)	1.04 (0.38-1.99)
bEmOC services	589 (253-1102)	229 (93-398)	1.04 (0.4-2.05)
cEmOC services	586 (253-1125)	228 (93-403)	1.04 (0.39-2.03)
**System-Relevant Interventions**			
Quality of care	461 (146-952)	174 (47-352)	0.83 (0.22-1.88)
Referral	588 (241-1112)	228 (92-402)	1.03 (0.39-2.03)
Transport	589 (248-1127)	229 (89-413)	1.04 (0.39-2.08)
Targeted transfers	589 (242-1075)	229 (90-388)	1.03 (0.41-1.95)
**Integrated Strategies**			
Family Planning	529 (210-1014)	212 (78-385)	0.91 (0.31-1.84)
Community + Linkages	578 (254-1085)	225 (90-409)	1.02 (0.41-2.01)
Facilities + Linkages	576 (249-1058)	224 (91-396)	1.02 (0.37-1.95)
Facilities + Linkages + Quality	448 (141-922)	171 (43-338)	0.81 (0.22-1.76)
Comprehensive	382 (95-854)	150 (31-309)	0.68 (0.12-1.59)

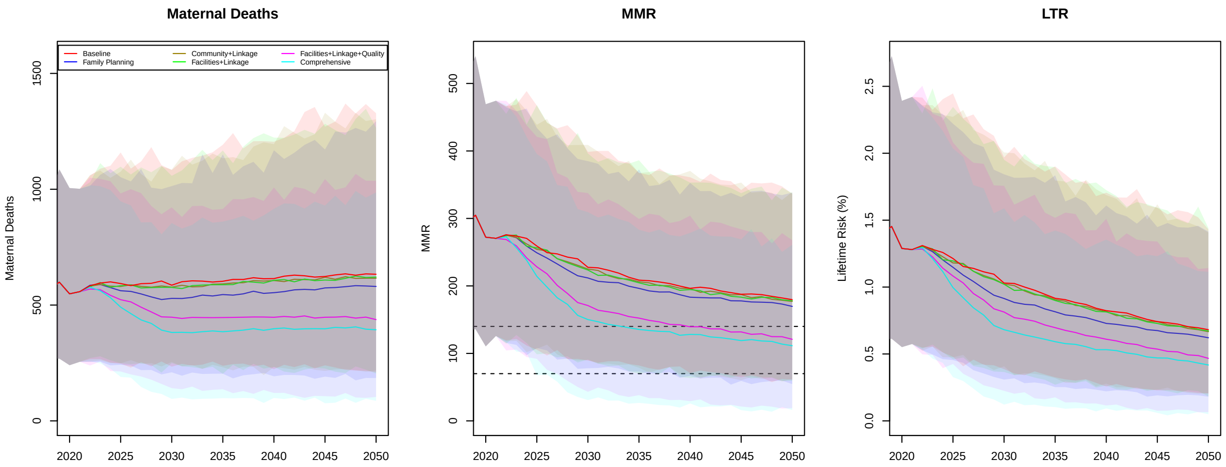

Costa Rica

ISO Code	Region	Area	Income Group
CRI	Central America	Latin America and the Caribbean	Upper middle income

Projected Maternal Indicators in 2030 by Scenario

Scenario	Maternal Deaths	MMR	LTR
**Baseline**	32 (5-70)	31 (1-76)	0.06 (0-0.13)
**Family Planning Interventions**			
Contraception	27 (3-62)	30 (0-78)	0.05 (0-0.12)
Medical abortion	32 (4-73)	32 (0-79)	0.06 (0-0.14)
**Community-Based Interventions**			
ANC	33 (3-73)	32 (1-81)	0.06 (0-0.15)
SBA	32 (4-73)	32 (2-80)	0.06 (0-0.14)
**Facility-Based Interventions**			
Facility births	32 (5-73)	31 (1-82)	0.05 (0-0.14)
nonEmOC services	33 (5-73)	32 (0-79)	0.06 (0-0.14)
bEmOC services	32 (6-69)	31 (2-76)	0.05 (0-0.14)
cEmOC services	32 (4-72)	31 (0-78)	0.05 (0-0.14)
**System-Relevant Interventions**			
Quality of care	32 (6-70)	31 (1-76)	0.06 (0-0.14)
Referral	33 (4-72)	33 (2-80)	0.06 (0-0.14)
Transport	32 (3-73)	33 (1-88)	0.06 (0-0.15)
Targeted transfers	32 (4-72)	32 (1-81)	0.06 (0-0.15)
**Integrated Strategies**			
Family Planning	26 (2-66)	28 (0-76)	0.04 (0-0.13)
Community + Linkages	32 (3-73)	32 (0-79)	0.06 (0-0.14)
Facilities + Linkages	32 (3-73)	31 (0-80)	0.05 (0-0.14)
Facilities + Linkages + Quality	31 (3-71)	31 (0-84)	0.05 (0-0.14)
Comprehensive	24 (1-59)	26 (0-72)	0.04 (0-0.11)

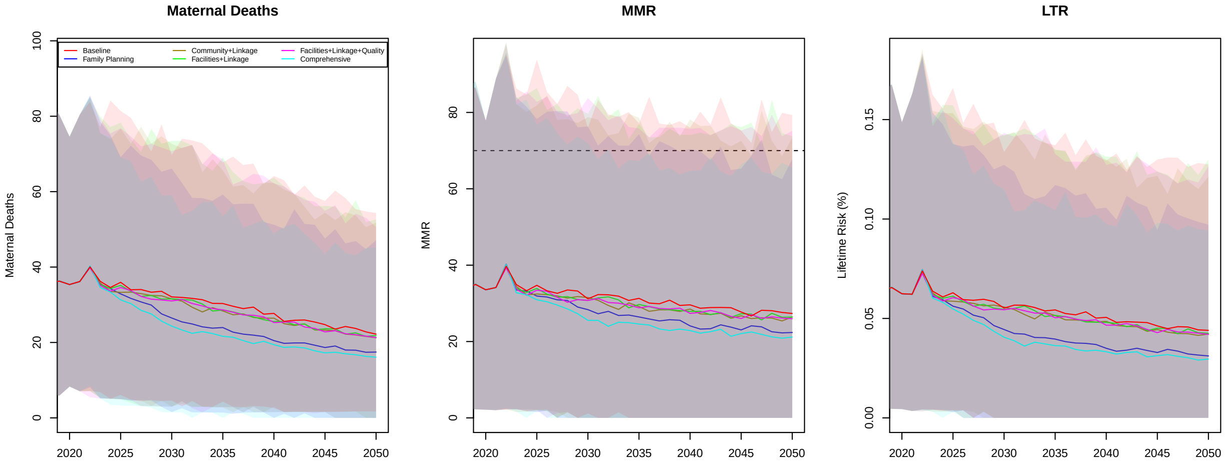

Côte d’Ivoire

ISO Code	Region	Area	Income Group
CIV	Western Africa	Africa	Lower middle income

Projected Maternal Indicators in 2030 by Scenario

Scenario	Maternal Deaths	MMR	LTR
**Baseline**	4480 (1860-8165)	357 (152-575)	1.63 (0.59-3.04)
**Family Planning Interventions**			
Contraception	3748 (1483-6984)	314 (125-523)	1.33 (0.44-2.58)
Medical abortion	4351 (1805-7790)	346 (143-564)	1.58 (0.55-2.92)
**Community-Based Interventions**			
ANC	4397 (1735-8034)	350 (145-571)	1.59 (0.56-2.99)
SBA	4393 (1809-7987)	350 (147-574)	1.6 (0.57-3.02)
**Facility-Based Interventions**			
Facility births	3765 (1416-7141)	295 (103-522)	1.38 (0.42-2.75)
nonEmOC services	4468 (1858-8044)	356 (149-576)	1.62 (0.57-3.03)
bEmOC services	4480 (1863-8232)	357 (147-578)	1.63 (0.6-3.08)
cEmOC services	4467 (1810-8061)	356 (149-577)	1.62 (0.57-3)
**System-Relevant Interventions**			
Quality of care	3963 (1452-7449)	313 (110-533)	1.45 (0.46-2.84)
Referral	4468 (1819-8197)	356 (150-574)	1.62 (0.59-3.05)
Transport	4322 (1747-8034)	345 (134-567)	1.58 (0.56-3.03)
Targeted transfers	4477 (1843-8011)	357 (150-574)	1.62 (0.58-3.01)
**Integrated Strategies**			
Family Planning	3686 (1443-6896)	308 (114-516)	1.31 (0.41-2.55)
Community + Linkages	4071 (1557-7607)	323 (120-539)	1.49 (0.48-2.89)
Facilities + Linkages	3696 (1294-7158)	290 (104-513)	1.36 (0.39-2.76)
Facilities + Linkages + Quality	3094 (871-6480)	240 (52-468)	1.16 (0.22-2.52)
Comprehensive	2513 (475-5348)	203 (29-403)	0.92 (0.1-2.05)

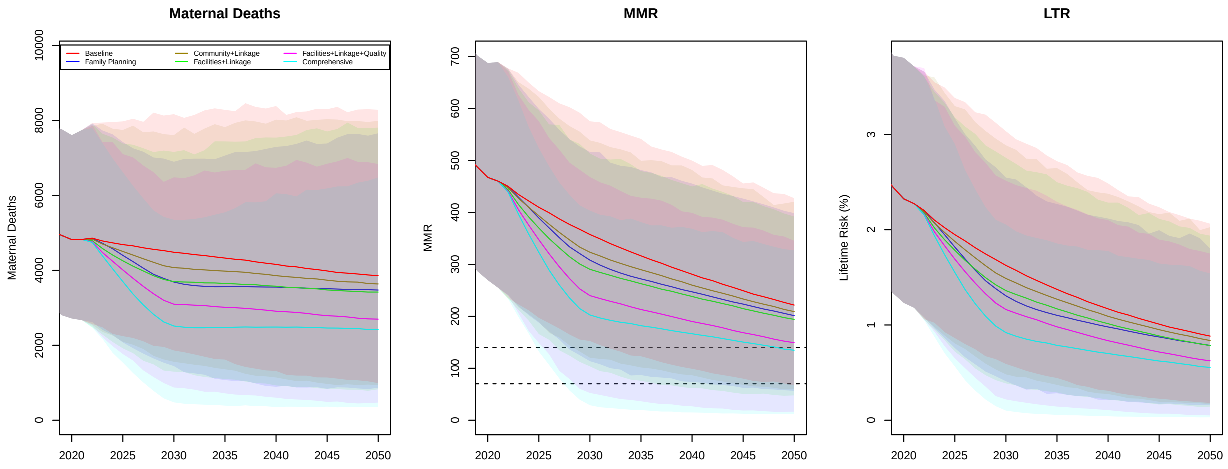

Croatia

ISO Code	Region	Area	Income Group
HRV	Southern Europe	Europe	High income

Projected Maternal Indicators in 2030 by Scenario

Scenario	Maternal Deaths	MMR	LTR
**Baseline**	13 (0-33)	25 (0-81)	0.04 (0-0.12)
**Family Planning Interventions**			
Contraception	13 (0-34)	24 (0-80)	0.04 (0-0.12)
Medical abortion	13 (0-36)	25 (0-81)	0.04 (0-0.12)
**Community-Based Interventions**			
ANC	13 (0-34)	25 (0-82)	0.04 (0-0.13)
SBA	13 (0-32)	25 (0-79)	0.04 (0-0.12)
**Facility-Based Interventions**			
Facility births	13 (0-32)	24 (0-77)	0.04 (0-0.12)
nonEmOC services	13 (0-33)	25 (0-81)	0.04 (0-0.12)
bEmOC services	13 (0-33)	25 (0-81)	0.04 (0-0.12)
cEmOC services	13 (0-33)	25 (0-78)	0.04 (0-0.12)
**System-Relevant Interventions**			
Quality of care	13 (0-33)	25 (0-81)	0.04 (0-0.12)
Referral	13 (0-34)	25 (0-89)	0.04 (0-0.13)
Transport	12 (0-34)	25 (0-84)	0.04 (0-0.12)
Targeted transfers	13 (0-33)	25 (0-84)	0.04 (0-0.12)
**Integrated Strategies**			
Family Planning	13 (0-33)	24 (0-77)	0.04 (0-0.12)
Community + Linkages	12 (0-35)	25 (0-80)	0.04 (0-0.12)
Facilities + Linkages	12 (0-36)	25 (0-83)	0.04 (0-0.13)
Facilities + Linkages + Quality	12 (0-36)	25 (0-83)	0.04 (0-0.13)
Comprehensive	11 (0-30)	21 (0-75)	0.03 (0-0.12)

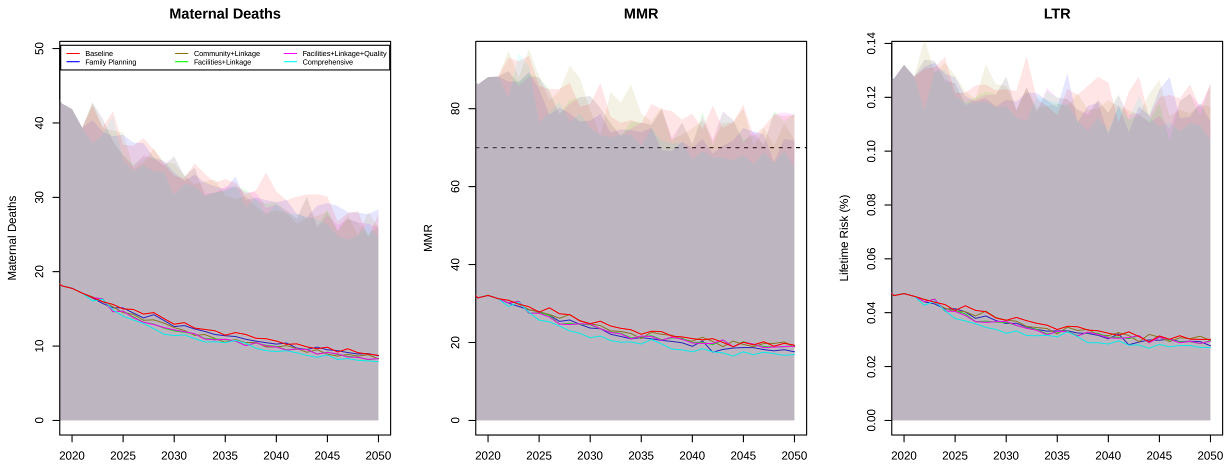

Cuba

ISO Code	Region	Area	Income Group
CUB	Caribbean	Latin America and the Caribbean	Upper middle income

Projected Maternal Indicators in 2030 by Scenario

Scenario	Maternal Deaths	MMR	LTR
**Baseline**	78 (0-206)	78 (0-238)	0.09 (0-0.27)
**Family Planning Interventions**			
Contraception	71 (0-196)	76 (0-231)	0.08 (0-0.26)
Medical abortion	78 (0-212)	79 (0-238)	0.09 (0-0.28)
**Community-Based Interventions**			
ANC	75 (0-199)	74 (0-215)	0.09 (0-0.26)
SBA	75 (0-199)	74 (0-212)	0.09 (0-0.26)
**Facility-Based Interventions**			
Facility births	71 (0-206)	70 (0-219)	0.08 (0-0.27)
nonEmOC services	77 (0-208)	77 (0-237)	0.09 (0-0.28)
bEmOC services	79 (0-205)	79 (0-241)	0.09 (0-0.27)
cEmOC services	78 (0-206)	77 (0-233)	0.09 (0-0.28)
**System-Relevant Interventions**			
Quality of care	75 (0-216)	74 (0-222)	0.09 (0-0.27)
Referral	75 (0-198)	76 (0-233)	0.09 (0-0.26)
Transport	73 (0-198)	74 (0-219)	0.09 (0-0.26)
Targeted transfers	74 (0-203)	73 (0-223)	0.09 (0-0.27)
**Integrated Strategies**			
Family Planning	69 (0-194)	76 (0-228)	0.08 (0-0.25)
Community + Linkages	67 (0-193)	67 (0-209)	0.08 (0-0.26)
Facilities + Linkages	67 (0-195)	67 (0-215)	0.08 (0-0.25)
Facilities + Linkages + Quality	63 (0-188)	61 (0-192)	0.07 (0-0.24)
Comprehensive	57 (0-174)	62 (0-206)	0.07 (0-0.23)

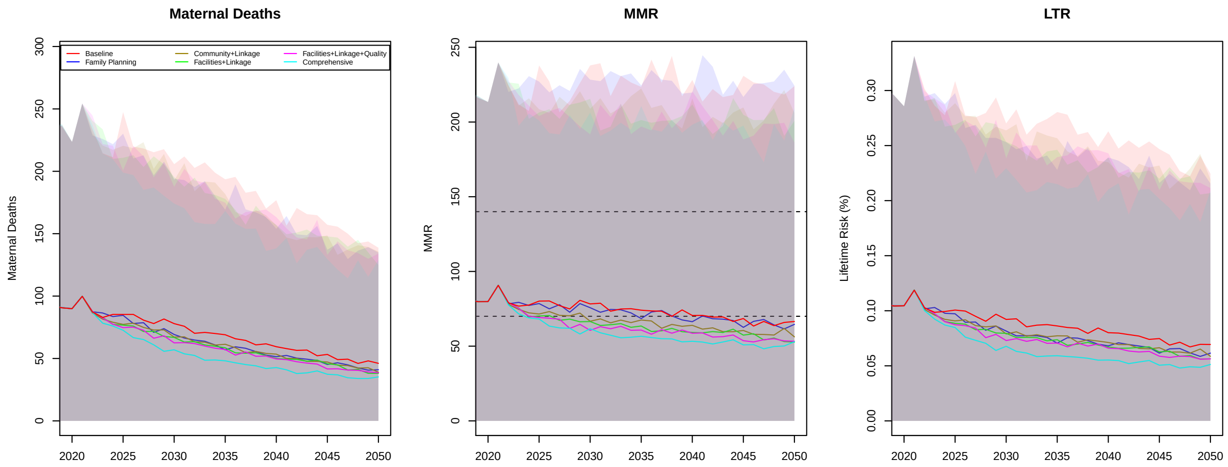

Cyprus

ISO Code	Region	Area	Income Group
CYP	Western Asia	Asia	High income

Projected Maternal Indicators in 2030 by Scenario

Scenario	Maternal Deaths	MMR	LTR
**Baseline**	4 (0-17)	26 (0-123)	0.03 (0-0.16)
**Family Planning Interventions**			
Contraception	4 (0-14)	21 (0-125)	0.03 (0-0.15)
Medical abortion	4 (0-14)	23 (0-120)	0.03 (0-0.16)
**Community-Based Interventions**			
ANC	3 (0-14)	23 (0-120)	0.03 (0-0.15)
SBA	4 (0-18)	26 (0-123)	0.03 (0-0.16)
**Facility-Based Interventions**			
Facility births	4 (0-17)	25 (0-123)	0.03 (0-0.16)
nonEmOC services	4 (0-17)	26 (0-123)	0.03 (0-0.16)
bEmOC services	4 (0-17)	26 (0-123)	0.03 (0-0.16)
cEmOC services	4 (0-19)	25 (0-137)	0.03 (0-0.16)
**System-Relevant Interventions**			
Quality of care	4 (0-18)	26 (0-122)	0.03 (0-0.16)
Referral	4 (0-17)	26 (0-123)	0.03 (0-0.16)
Transport	4 (0-14)	25 (0-136)	0.03 (0-0.16)
Targeted transfers	4 (0-17)	26 (0-123)	0.03 (0-0.16)
**Integrated Strategies**			
Family Planning	3 (0-14)	20 (0-122)	0.03 (0-0.16)
Community + Linkages	3 (0-14)	23 (0-137)	0.03 (0-0.16)
Facilities + Linkages	4 (0-19)	25 (0-139)	0.03 (0-0.16)
Facilities + Linkages + Quality	4 (0-19)	26 (0-139)	0.03 (0-0.16)
Comprehensive	3 (0-14)	18 (0-112)	0.02 (0-0.15)

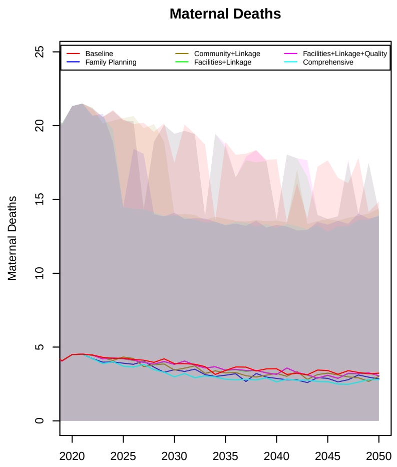

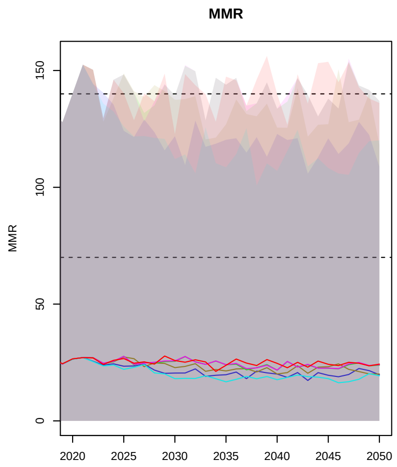

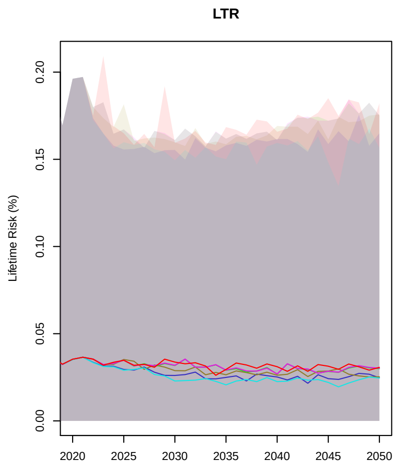

Czechia

ISO Code	Region	Area	Income Group
CZE	Eastern Europe	Europe	High income

Projected Maternal Indicators in 2030 by Scenario

Scenario	Maternal Deaths	MMR	LTR
**Baseline**	31 (0-84)	28 (0-85)	0.04 (0-0.11)
**Family Planning Interventions**			
Contraception	31 (0-84)	26 (0-82)	0.04 (0-0.12)
Medical abortion	31 (0-77)	28 (0-88)	0.04 (0-0.12)
**Community-Based Interventions**			
ANC	29 (0-76)	26 (0-86)	0.03 (0-0.11)
SBA	31 (0-84)	28 (0-85)	0.04 (0-0.11)
**Facility-Based Interventions**			
Facility births	31 (0-83)	28 (0-84)	0.04 (0-0.11)
nonEmOC services	31 (0-84)	28 (0-85)	0.04 (0-0.11)
bEmOC services	31 (0-79)	27 (0-86)	0.04 (0-0.11)
cEmOC services	30 (0-78)	26 (0-88)	0.03 (0-0.11)
**System-Relevant Interventions**			
Quality of care	30 (0-76)	26 (0-86)	0.03 (0-0.11)
Referral	31 (0-83)	28 (0-84)	0.04 (0-0.11)
Transport	28 (0-76)	26 (0-86)	0.03 (0-0.11)
Targeted transfers	30 (0-77)	28 (0-86)	0.04 (0-0.11)
**Integrated Strategies**			
Family Planning	30 (0-82)	26 (0-79)	0.04 (0-0.11)
Community + Linkages	29 (0-84)	26 (0-83)	0.03 (0-0.11)
Facilities + Linkages	28 (0-75)	25 (0-78)	0.03 (0-0.1)
Facilities + Linkages + Quality	28 (0-76)	25 (0-85)	0.03 (0-0.1)
Comprehensive	28 (0-78)	23 (0-82)	0.03 (0-0.12)

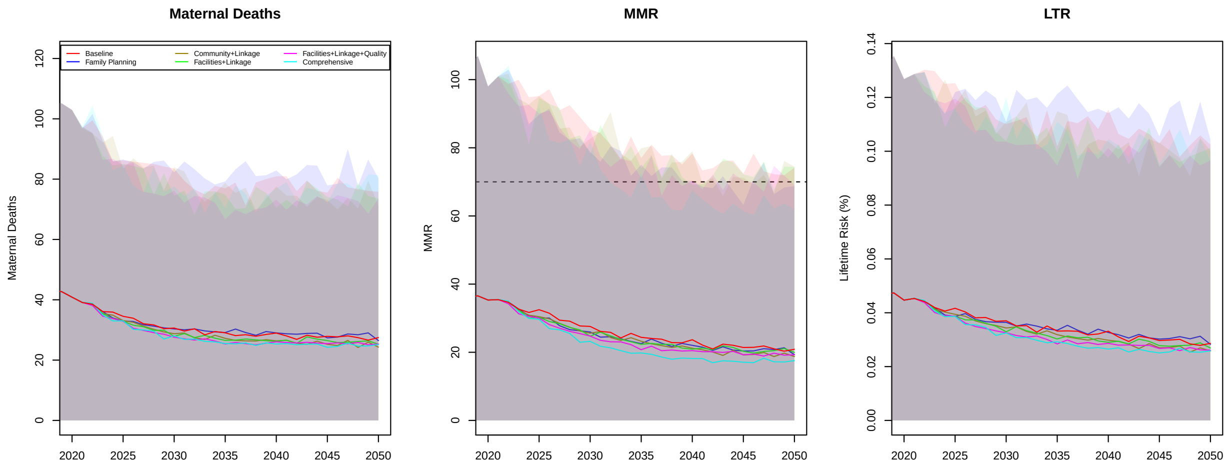

Dem. People's Republic of Korea

ISO Code	Region	Area	Income Group
PRK	Eastern Asia	Asia	Low income

Projected Maternal Indicators in 2030 by Scenario

Scenario	Maternal Deaths	MMR	LTR
**Baseline**	295 (56-647)	42 (0-131)	0.08 (0-0.25)
**Family Planning Interventions**			
Contraception	296 (58-635)	42 (0-121)	0.08 (0-0.22)
Medical abortion	295 (45-633)	42 (0-121)	0.08 (0-0.23)
**Community-Based Interventions**			
ANC	297 (45-690)	41 (0-116)	0.08 (0-0.24)
SBA	293 (55-636)	42 (0-129)	0.08 (0-0.24)
**Facility-Based Interventions**			
Facility births	287 (54-629)	40 (0-122)	0.07 (0-0.22)
nonEmOC services	299 (55-640)	43 (0-123)	0.08 (0-0.23)
bEmOC services	297 (51-654)	44 (0-128)	0.08 (0-0.24)
cEmOC services	282 (54-628)	37 (0-109)	0.07 (0-0.22)
**System-Relevant Interventions**			
Quality of care	255 (42-535)	32 (0-94)	0.06 (0-0.17)
Referral	297 (55-652)	43 (0-124)	0.08 (0-0.23)
Transport	285 (54-649)	43 (0-120)	0.08 (0-0.23)
Targeted transfers	298 (56-656)	43 (0-129)	0.08 (0-0.25)
**Integrated Strategies**			
Family Planning	299 (56-678)	43 (0-125)	0.08 (0-0.23)
Community + Linkages	270 (43-610)	38 (0-112)	0.07 (0-0.21)
Facilities + Linkages	252 (41-560)	32 (0-97)	0.06 (0-0.19)
Facilities + Linkages + Quality	213 (41-452)	22 (0-67)	0.04 (0-0.12)
Comprehensive	202 (0-446)	20 (0-64)	0.04 (0-0.12)

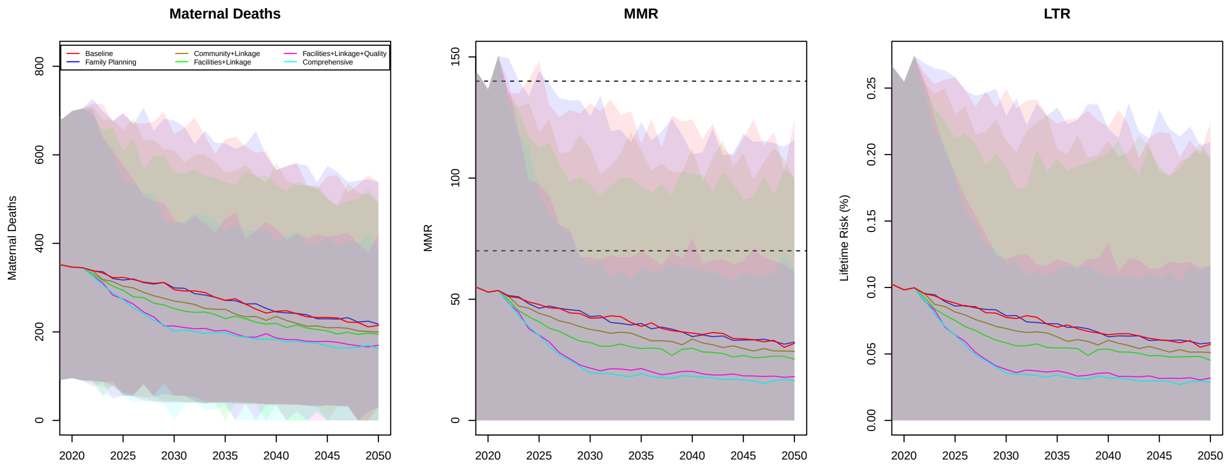

Democratic Republic of the Congo

ISO Code	Region	Area	Income Group
COD	Middle Africa	Africa	Low income

Projected Maternal Indicators in 2030 by Scenario

Scenario	Maternal Deaths	MMR	LTR
**Baseline**	23542 (14794-35483)	334 (216-516)	2.05 (1.21-3.36)
**Family Planning Interventions**			
Contraception	22395 (13648-34695)	330 (212-494)	1.95 (1.13-3.29)
Medical abortion	23480 (14875-35575)	334 (214-518)	2.05 (1.21-3.33)
**Community-Based Interventions**			
ANC	23113 (15093-34946)	328 (218-494)	2.01 (1.23-3.26)
SBA	23477 (14605-35145)	333 (218-508)	2.05 (1.22-3.37)
**Facility-Based Interventions**			
Facility births	21756 (13589-33520)	306 (192-474)	1.85 (1.06-3.11)
nonEmOC services	23460 (14949-35757)	333 (216-510)	2.04 (1.18-3.35)
bEmOC services	23450 (15051-35820)	333 (216-516)	2.04 (1.23-3.38)
cEmOC services	22089 (13742-33615)	311 (196-489)	1.92 (1.11-3.24)
**System-Relevant Interventions**			
Quality of care	11151 (5224-22339)	140 (54-317)	0.9 (0.29-2.19)
Referral	23502 (14874-35535)	333 (217-515)	2.05 (1.23-3.4)
Transport	22924 (14474-34340)	327 (214-502)	2 (1.21-3.23)
Targeted transfers	23540 (14966-35304)	334 (217-503)	2.05 (1.19-3.32)
**Integrated Strategies**			
Family Planning	22440 (13463-35275)	331 (213-500)	1.95 (1.11-3.32)
Community + Linkages	22153 (13773-34465)	316 (204-489)	1.92 (1.11-3.23)
Facilities + Linkages	20069 (12238-31660)	282 (173-452)	1.71 (0.93-2.98)
Facilities + Linkages + Quality	7797 (3189-18830)	91 (26-261)	0.56 (0.11-1.85)
Comprehensive	7320 (2704-18585)	88 (23-265)	0.53 (0.08-1.82)

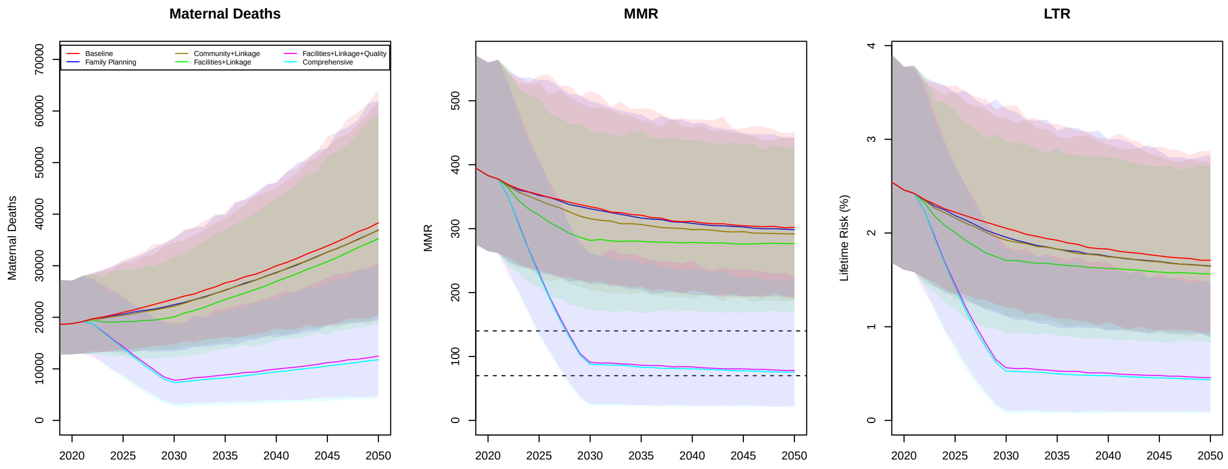

Denmark

ISO Code	Region	Area	Income Group
DNK	Northern Europe	Europe	High income

Projected Maternal Indicators in 2030 by Scenario

Scenario	Maternal Deaths	MMR	LTR
**Baseline**	18 (0-43)	10 (0-36)	0.02 (0-0.07)
**Family Planning Interventions**			
Contraception	17 (0-42)	10 (0-35)	0.02 (0-0.07)
Medical abortion	17 (0-44)	10 (0-36)	0.02 (0-0.08)
**Community-Based Interventions**			
ANC	17 (0-43)	10 (0-36)	0.02 (0-0.07)
SBA	18 (0-45)	10 (0-33)	0.02 (0-0.07)
**Facility-Based Interventions**			
Facility births	18 (0-46)	10 (0-36)	0.02 (0-0.08)
nonEmOC services	18 (0-43)	10 (0-36)	0.02 (0-0.07)
bEmOC services	18 (0-43)	10 (0-36)	0.02 (0-0.07)
cEmOC services	18 (0-43)	10 (0-36)	0.02 (0-0.07)
**System-Relevant Interventions**			
Quality of care	18 (0-43)	10 (0-36)	0.02 (0-0.07)
Referral	17 (0-44)	10 (0-36)	0.02 (0-0.07)
Transport	17 (0-42)	10 (0-35)	0.02 (0-0.07)
Targeted transfers	18 (0-43)	10 (0-36)	0.02 (0-0.07)
**Integrated Strategies**			
Family Planning	17 (0-41)	10 (0-35)	0.02 (0-0.07)
Community + Linkages	17 (0-43)	10 (0-36)	0.02 (0-0.08)
Facilities + Linkages	16 (0-43)	9 (0-32)	0.02 (0-0.07)
Facilities + Linkages + Quality	16 (0-43)	9 (0-32)	0.02 (0-0.07)
Comprehensive	16 (0-40)	9 (0-33)	0.02 (0-0.07)

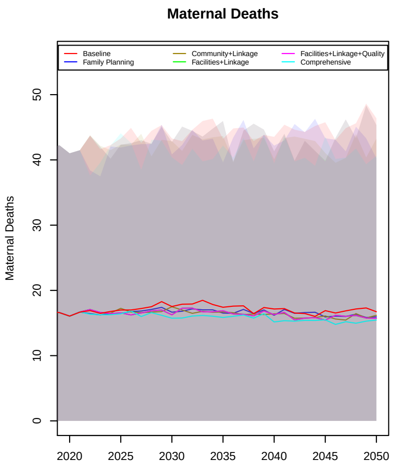

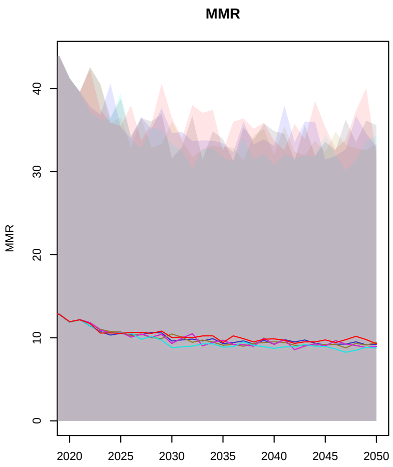

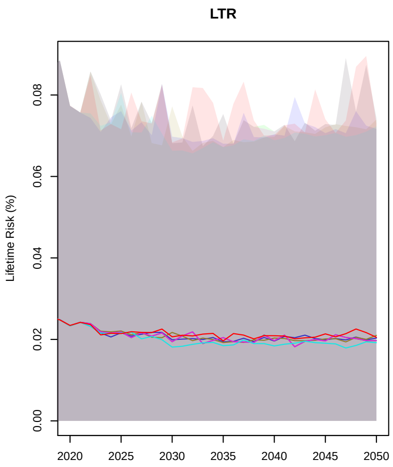

Djibouti

ISO Code	Region	Area	Income Group
DJI	Eastern Africa	Africa	Lower middle income

Projected Maternal Indicators in 2030 by Scenario

Scenario	Maternal Deaths	MMR	LTR
**Baseline**	52 (4-252)	208 (0-1174)	0.45 (0-2.4)
**Family Planning Interventions**			
Contraception	34 (0-112)	127 (0-454)	0.27 (0-1.02)
Medical abortion	31 (0-116)	109 (0-394)	0.24 (0-1.04)
**Community-Based Interventions**			
ANC	52 (3-256)	208 (0-1139)	0.45 (0-2.5)
SBA	50 (0-238)	197 (0-1012)	0.42 (0-2.18)
**Facility-Based Interventions**			
Facility births	48 (0-241)	189 (0-1082)	0.41 (0-2.3)
nonEmOC services	52 (3-256)	208 (0-1174)	0.44 (0-2.4)
bEmOC services	52 (3-239)	209 (0-1069)	0.44 (0-2.26)
cEmOC services	51 (0-238)	199 (0-1102)	0.43 (0-2.27)
**System-Relevant Interventions**			
Quality of care	52 (4-259)	210 (0-1193)	0.45 (0-2.44)
Referral	52 (3-256)	208 (0-1176)	0.44 (0-2.49)
Transport	50 (0-248)	203 (0-1200)	0.43 (0-2.38)
Targeted transfers	52 (4-249)	205 (0-1146)	0.44 (0-2.37)
**Integrated Strategies**			
Family Planning	26 (0-93)	91 (0-359)	0.2 (0-0.9)
Community + Linkages	50 (0-245)	199 (0-1094)	0.43 (0-2.28)
Facilities + Linkages	47 (0-248)	186 (0-1122)	0.4 (0-2.3)
Facilities + Linkages + Quality	47 (0-234)	186 (0-1076)	0.4 (0-2.25)
Comprehensive	21 (0-83)	68 (0-308)	0.15 (0-0.76)

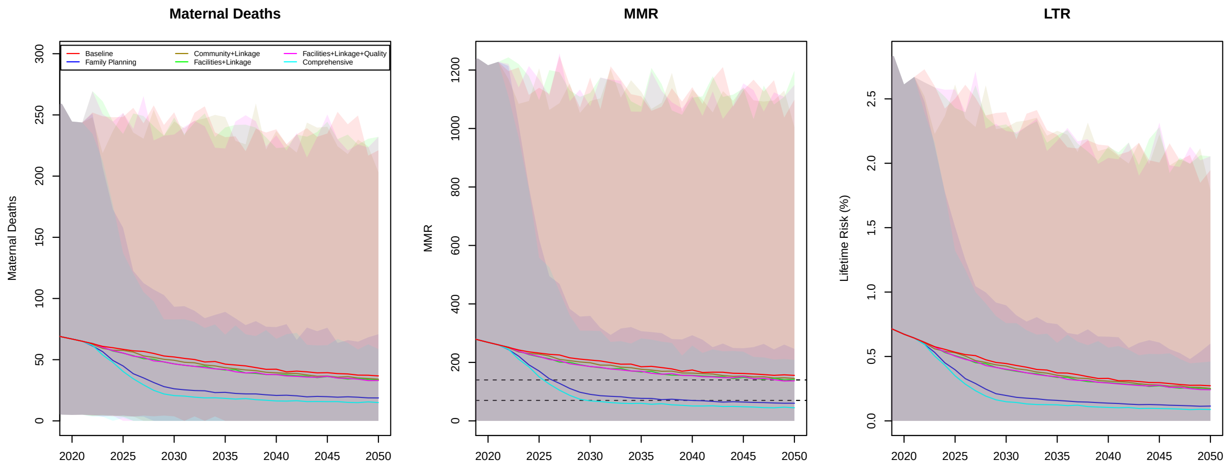

Dominica

ISO Code	Region	Area	Income Group
DMA	Caribbean	Latin America and the Caribbean	Upper middle income

Projected Maternal Indicators in 2030 by Scenario

Scenario	Maternal Deaths	MMR	LTR
**Baseline**	1 (0-5)	64 (0-323)	0.15 (0-0.79)
**Family Planning Interventions**			
Contraception	1 (0-5)	63 (0-357)	0.14 (0-0.8)
Medical abortion	1 (0-5)	63 (0-336)	0.15 (0-0.85)
**Community-Based Interventions**			
ANC	1 (0-5)	63 (0-324)	0.14 (0-0.75)
SBA	1 (0-5)	63 (0-323)	0.15 (0-0.79)
**Facility-Based Interventions**			
Facility births	1 (0-5)	64 (0-323)	0.15 (0-0.79)
nonEmOC services	1 (0-5)	63 (0-324)	0.14 (0-0.78)
bEmOC services	1 (0-5)	64 (0-323)	0.15 (0-0.79)
cEmOC services	1 (0-5)	64 (0-344)	0.15 (0-0.8)
**System-Relevant Interventions**			
Quality of care	1 (0-4)	57 (0-327)	0.13 (0-0.73)
Referral	1 (0-5)	63 (0-320)	0.15 (0-0.75)
Transport	1 (0-5)	64 (0-342)	0.14 (0-0.73)
Targeted transfers	1 (0-5)	64 (0-323)	0.15 (0-0.78)
**Integrated Strategies**			
Family Planning	1 (0-5)	61 (0-345)	0.14 (0-0.75)
Community + Linkages	1 (0-5)	64 (0-320)	0.14 (0-0.78)
Facilities + Linkages	1 (0-5)	68 (0-347)	0.15 (0-0.75)
Facilities + Linkages + Quality	1 (0-4)	50 (0-297)	0.11 (0-0.62)
Comprehensive	1 (0-4)	54 (0-296)	0.12 (0-0.7)

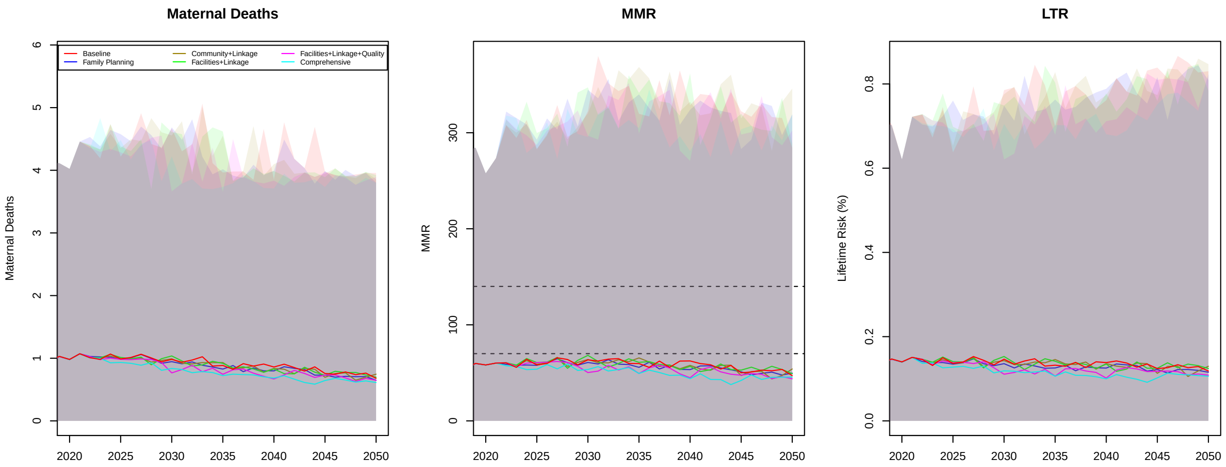

Dominican Republic

ISO Code	Region	Area	Income Group
DOM	Caribbean	Latin America and the Caribbean	Upper middle income

Projected Maternal Indicators in 2030 by Scenario

Scenario	Maternal Deaths	MMR	LTR
**Baseline**	208 (18-482)	66 (2-167)	0.2 (0.01-0.51)
**Family Planning Interventions**			
Contraception	186 (10-428)	63 (0-159)	0.17 (0-0.44)
Medical abortion	207 (22-488)	66 (2-161)	0.2 (0.01-0.5)
**Community-Based Interventions**			
ANC	208 (23-470)	66 (2-166)	0.2 (0.01-0.49)
SBA	206 (17-477)	65 (0-164)	0.19 (0-0.48)
**Facility-Based Interventions**			
Facility births	210 (18-490)	66 (2-167)	0.2 (0.01-0.5)
nonEmOC services	207 (24-481)	66 (2-170)	0.2 (0-0.5)
bEmOC services	207 (20-482)	66 (3-167)	0.2 (0.01-0.5)
cEmOC services	209 (18-491)	65 (2-168)	0.19 (0.01-0.51)
**System-Relevant Interventions**			
Quality of care	199 (27-460)	63 (0-167)	0.19 (0-0.48)
Referral	206 (16-484)	66 (2-166)	0.19 (0.01-0.51)
Transport	202 (24-447)	66 (3-153)	0.19 (0.01-0.47)
Targeted transfers	209 (18-470)	66 (0-167)	0.2 (0-0.49)
**Integrated Strategies**			
Family Planning	183 (14-447)	63 (0-164)	0.17 (0-0.45)
Community + Linkages	204 (13-467)	67 (0-172)	0.2 (0-0.51)
Facilities + Linkages	203 (31-463)	66 (3-169)	0.2 (0.01-0.5)
Facilities + Linkages + Quality	192 (15-463)	62 (0-156)	0.19 (0-0.47)
Comprehensive	171 (5-410)	59 (0-151)	0.16 (0-0.42)

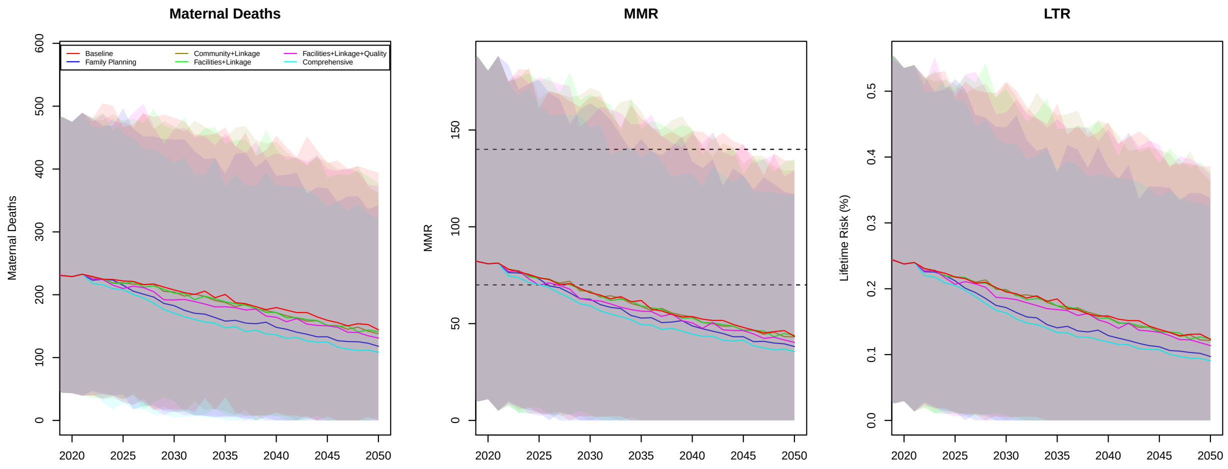

Ecuador

ISO Code	Region	Area	Income Group
ECU	South America	Latin America and the Caribbean	Upper middle income

Projected Maternal Indicators in 2030 by Scenario

Scenario	Maternal Deaths	MMR	LTR
**Baseline**	251 (77-521)	58 (12-126)	0.13 (0.03-0.31)
**Family Planning Interventions**			
Contraception	215 (66-485)	52 (11-130)	0.11 (0.02-0.28)
Medical abortion	252 (77-522)	59 (12-128)	0.13 (0.03-0.31)
**Community-Based Interventions**			
ANC	245 (75-534)	57 (13-133)	0.13 (0.03-0.31)
SBA	240 (67-530)	54 (11-126)	0.12 (0.03-0.3)
**Facility-Based Interventions**			
Facility births	183 (37-456)	38 (1-111)	0.09 (0-0.26)
nonEmOC services	250 (73-526)	58 (12-132)	0.13 (0.03-0.31)
bEmOC services	250 (74-522)	58 (11-126)	0.13 (0.02-0.31)
cEmOC services	252 (84-532)	58 (14-127)	0.13 (0.03-0.3)
**System-Relevant Interventions**			
Quality of care	244 (62-506)	56 (9-127)	0.13 (0.02-0.31)
Referral	248 (73-522)	57 (12-135)	0.13 (0.03-0.3)
Transport	232 (70-486)	54 (11-126)	0.12 (0.02-0.29)
Targeted transfers	250 (71-512)	58 (12-128)	0.13 (0.03-0.3)
**Integrated Strategies**			
Family Planning	215 (66-485)	51 (10-130)	0.11 (0.02-0.28)
Community + Linkages	210 (63-471)	48 (9-119)	0.11 (0.02-0.28)
Facilities + Linkages	171 (29-435)	36 (1-107)	0.08 (0-0.25)
Facilities + Linkages + Quality	157 (27-417)	32 (0-103)	0.07 (0-0.25)
Comprehensive	143 (15-362)	31 (0-95)	0.07 (0-0.21)

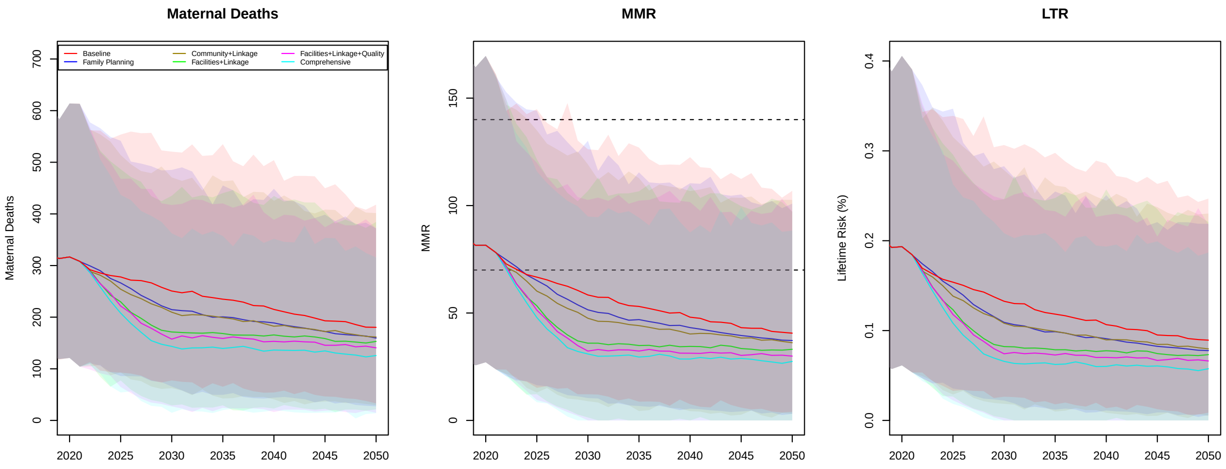

Egypt

ISO Code	Region	Area	Income Group
EGY	Northern Africa	Africa	Lower middle income

Projected Maternal Indicators in 2030 by Scenario

Scenario	Maternal Deaths	MMR	LTR
**Baseline**	1386 (622-2337)	27 (8-55)	0.08 (0.02-0.16)
**Family Planning Interventions**			
Contraception	1353 (606-2209)	26 (7-51)	0.08 (0.02-0.16)
Medical abortion	1390 (641-2332)	27 (8-55)	0.08 (0.02-0.16)
**Community-Based Interventions**			
ANC	1344 (641-2258)	27 (8-53)	0.08 (0.02-0.16)
SBA	1335 (654-2152)	26 (8-50)	0.08 (0.02-0.15)
**Facility-Based Interventions**			
Facility births	1154 (542-1948)	19 (4-42)	0.06 (0.01-0.13)
nonEmOC services	1391 (641-2327)	28 (8-55)	0.08 (0.02-0.16)
bEmOC services	1379 (640-2311)	27 (8-55)	0.08 (0.02-0.16)
cEmOC services	1321 (588-2229)	25 (7-52)	0.08 (0.02-0.16)
**System-Relevant Interventions**			
Quality of care	1357 (668-2245)	27 (7-53)	0.08 (0.02-0.16)
Referral	1385 (673-2327)	27 (8-54)	0.08 (0.02-0.17)
Transport	1205 (578-1981)	25 (7-48)	0.08 (0.02-0.15)
Targeted transfers	1382 (659-2347)	27 (8-54)	0.08 (0.02-0.16)
**Integrated Strategies**			
Family Planning	1361 (613-2267)	26 (7-53)	0.08 (0.02-0.16)
Community + Linkages	1105 (498-1927)	22 (7-44)	0.07 (0.02-0.13)
Facilities + Linkages	937 (412-1699)	15 (2-36)	0.05 (0.01-0.11)
Facilities + Linkages + Quality	906 (354-1610)	14 (2-34)	0.04 (0-0.11)
Comprehensive	858 (288-1503)	14 (1-33)	0.04 (0-0.1)

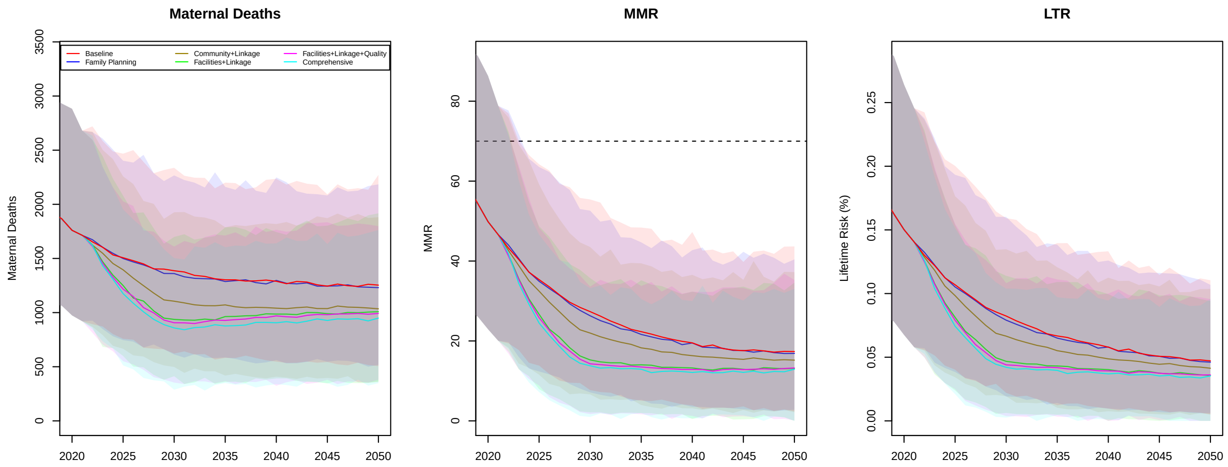

El Salvador

ISO Code	Region	Area	Income Group
SLV	Central America	Latin America and the Caribbean	Lower middle income

Projected Maternal Indicators in 2030 by Scenario

Scenario	Maternal Deaths	MMR	LTR
**Baseline**	149 (31-337)	106 (11-279)	0.21 (0.02-0.58)
**Family Planning Interventions**			
Contraception	140 (25-317)	101 (10-258)	0.2 (0.02-0.5)
Medical abortion	147 (28-347)	105 (10-266)	0.21 (0.02-0.55)
**Community-Based Interventions**			
ANC	147 (24-338)	104 (7-269)	0.21 (0.01-0.54)
SBA	140 (29-318)	98 (8-244)	0.2 (0.01-0.51)
**Facility-Based Interventions**			
Facility births	98 (8-247)	56 (0-181)	0.12 (0-0.38)
nonEmOC services	149 (28-337)	106 (11-284)	0.22 (0.02-0.58)
bEmOC services	150 (32-343)	107 (11-284)	0.22 (0.02-0.57)
cEmOC services	147 (23-341)	106 (10-267)	0.21 (0.02-0.55)
**System-Relevant Interventions**			
Quality of care	136 (25-329)	96 (6-257)	0.19 (0.01-0.53)
Referral	148 (25-338)	105 (8-273)	0.21 (0.01-0.58)
Transport	136 (24-302)	97 (8-239)	0.2 (0.01-0.48)
Targeted transfers	150 (29-342)	108 (10-284)	0.22 (0.02-0.58)
**Integrated Strategies**			
Family Planning	133 (24-308)	96 (9-249)	0.19 (0.01-0.48)
Community + Linkages	119 (17-283)	82 (6-230)	0.17 (0.01-0.47)
Facilities + Linkages	92 (6-251)	55 (0-177)	0.11 (0-0.39)
Facilities + Linkages + Quality	79 (5-234)	43 (0-175)	0.09 (0-0.37)
Comprehensive	66 (0-205)	35 (0-144)	0.07 (0-0.3)

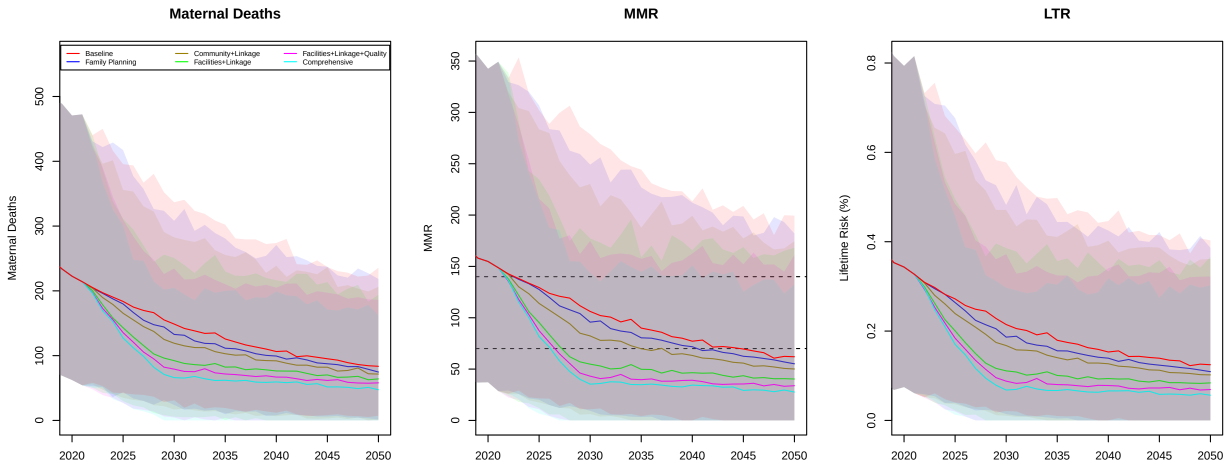

Equatorial Guinea

ISO Code	Region	Area	Income Group
GNQ	Middle Africa	Africa	Upper middle income

Projected Maternal Indicators in 2030 by Scenario

Scenario	Maternal Deaths	MMR	LTR
**Baseline**	111 (29-250)	146 (36-312)	0.74 (0.11-1.74)
**Family Planning Interventions**			
Contraception	100 (24-221)	135 (23-301)	0.64 (0.09-1.51)
Medical abortion	106 (23-238)	138 (23-312)	0.68 (0.08-1.68)
**Community-Based Interventions**			
ANC	113 (26-239)	149 (28-311)	0.75 (0.12-1.68)
SBA	105 (22-242)	138 (25-308)	0.69 (0.1-1.69)
**Facility-Based Interventions**			
Facility births	89 (15-206)	115 (12-270)	0.59 (0.04-1.46)
nonEmOC services	112 (28-249)	146 (36-319)	0.73 (0.11-1.71)
bEmOC services	111 (25-250)	145 (32-312)	0.73 (0.11-1.74)
cEmOC services	111 (29-241)	145 (34-304)	0.73 (0.12-1.69)
**System-Relevant Interventions**			
Quality of care	109 (26-242)	144 (27-324)	0.73 (0.11-1.73)
Referral	110 (26-238)	144 (33-304)	0.73 (0.11-1.66)
Transport	108 (25-240)	143 (29-323)	0.72 (0.12-1.66)
Targeted transfers	111 (28-251)	145 (35-312)	0.73 (0.11-1.71)
**Integrated Strategies**			
Family Planning	100 (20-233)	133 (23-303)	0.62 (0.07-1.59)
Community + Linkages	101 (20-233)	133 (23-298)	0.67 (0.09-1.6)
Facilities + Linkages	87 (15-200)	112 (13-274)	0.58 (0.05-1.46)
Facilities + Linkages + Quality	83 (11-203)	107 (10-254)	0.55 (0.03-1.4)
Comprehensive	73 (4-196)	94 (1-245)	0.46 (0-1.4)

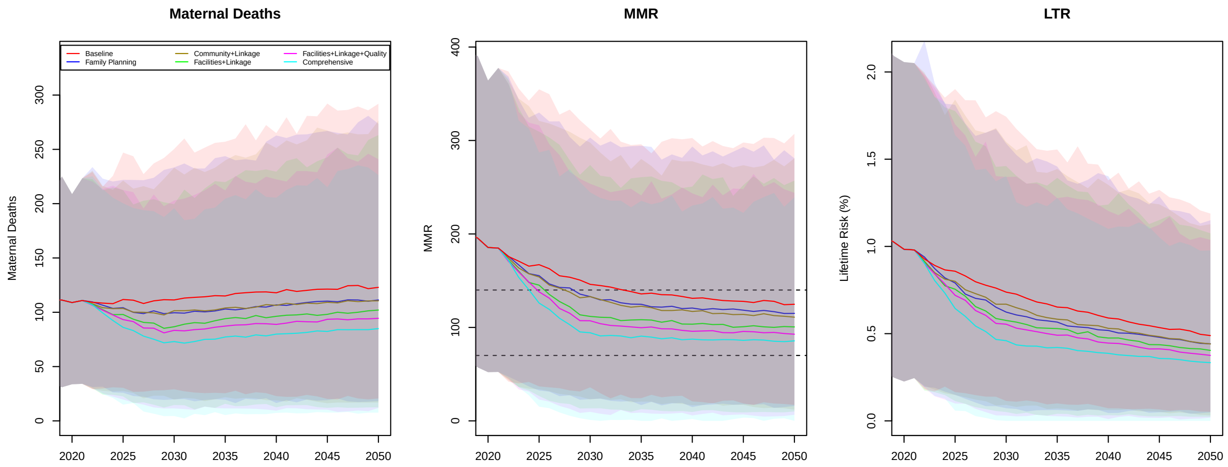

Eritrea

ISO Code	Region	Area	Income Group
ERI	Eastern Africa	Africa	Low income

Projected Maternal Indicators in 2030 by Scenario

Scenario	Maternal Deaths	MMR	LTR
**Baseline**	492 (186-914)	341 (130-598)	1.26 (0.41-2.42)
**Family Planning Interventions**			
Contraception	426 (157-792)	308 (115-568)	1.07 (0.34-2.02)
Medical abortion	484 (187-914)	328 (130-614)	1.24 (0.41-2.42)
**Community-Based Interventions**			
ANC	481 (187-898)	333 (116-612)	1.23 (0.37-2.38)
SBA	479 (160-907)	332 (119-603)	1.23 (0.37-2.41)
**Facility-Based Interventions**			
Facility births	320 (91-665)	208 (49-419)	0.74 (0.17-1.7)
nonEmOC services	492 (188-909)	342 (142-619)	1.26 (0.43-2.39)
bEmOC services	493 (180-894)	342 (125-606)	1.26 (0.42-2.39)
cEmOC services	490 (165-914)	339 (124-607)	1.25 (0.4-2.48)
**System-Relevant Interventions**			
Quality of care	332 (80-696)	221 (43-472)	0.83 (0.14-1.82)
Referral	493 (178-927)	341 (130-640)	1.26 (0.41-2.43)
Transport	467 (164-934)	322 (110-590)	1.18 (0.33-2.45)
Targeted transfers	493 (182-904)	342 (127-595)	1.26 (0.39-2.42)
**Integrated Strategies**			
Family Planning	427 (157-805)	306 (112-557)	1.07 (0.33-2.05)
Community + Linkages	404 (136-782)	276 (102-519)	1 (0.31-2.03)
Facilities + Linkages	308 (87-614)	202 (47-416)	0.71 (0.15-1.53)
Facilities + Linkages + Quality	116 (0-330)	62 (0-178)	0.2 (0-0.71)
Comprehensive	93 (0-258)	43 (0-146)	0.14 (0-0.49)

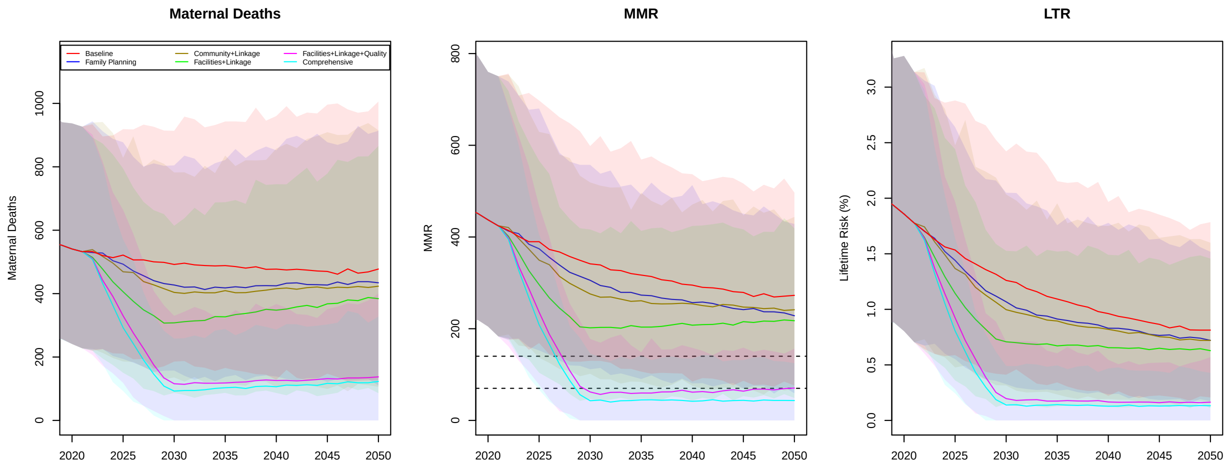

Estonia

ISO Code	Region	Area	Income Group
EST	Northern Europe	Europe	High income

Projected Maternal Indicators in 2030 by Scenario

Scenario	Maternal Deaths	MMR	LTR
**Baseline**	4 (0-14)	21 (0-115)	0.03 (0-0.15)
**Family Planning Interventions**			
Contraception	4 (0-14)	20 (0-101)	0.03 (0-0.15)
Medical abortion	4 (0-14)	21 (0-110)	0.03 (0-0.15)
**Community-Based Interventions**			
ANC	4 (0-14)	20 (0-104)	0.03 (0-0.15)
SBA	4 (0-14)	21 (0-115)	0.03 (0-0.15)
**Facility-Based Interventions**			
Facility births	4 (0-14)	21 (0-115)	0.03 (0-0.15)
nonEmOC services	4 (0-14)	21 (0-115)	0.03 (0-0.15)
bEmOC services	4 (0-14)	21 (0-115)	0.03 (0-0.15)
cEmOC services	4 (0-14)	20 (0-108)	0.03 (0-0.15)
**System-Relevant Interventions**			
Quality of care	4 (0-17)	23 (0-115)	0.03 (0-0.16)
Referral	4 (0-14)	21 (0-115)	0.03 (0-0.15)
Transport	4 (0-14)	22 (0-104)	0.03 (0-0.16)
Targeted transfers	4 (0-14)	21 (0-115)	0.03 (0-0.15)
**Integrated Strategies**			
Family Planning	3 (0-13)	20 (0-100)	0.03 (0-0.15)
Community + Linkages	4 (0-14)	22 (0-104)	0.03 (0-0.15)
Facilities + Linkages	4 (0-14)	21 (0-102)	0.03 (0-0.16)
Facilities + Linkages + Quality	4 (0-14)	21 (0-106)	0.03 (0-0.15)
Comprehensive	3 (0-14)	18 (0-98)	0.03 (0-0.15)

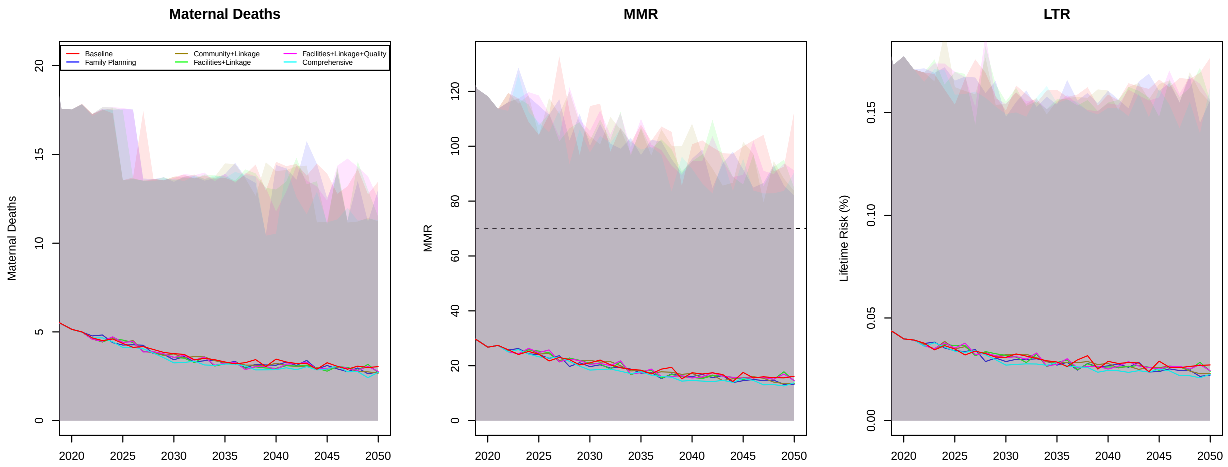

Eswatini

ISO Code	Region	Area	Income Group
SWZ	Southern Africa	Africa	Lower middle income

Projected Maternal Indicators in 2030 by Scenario

Scenario	Maternal Deaths	MMR	LTR
**Baseline**	68 (7-174)	147 (0-416)	0.53 (0-1.5)
**Family Planning Interventions**			
Contraception	56 (0-155)	117 (0-355)	0.41 (0-1.35)
Medical abortion	56 (0-161)	114 (0-353)	0.41 (0-1.37)
**Community-Based Interventions**			
ANC	66 (6-167)	142 (0-413)	0.51 (0-1.44)
SBA	67 (5-171)	144 (0-426)	0.51 (0-1.52)
**Facility-Based Interventions**			
Facility births	59 (0-167)	125 (0-401)	0.45 (0-1.38)
nonEmOC services	68 (8-172)	147 (7-403)	0.53 (0-1.45)
bEmOC services	68 (6-181)	147 (0-423)	0.53 (0-1.55)
cEmOC services	59 (0-162)	127 (0-379)	0.46 (0-1.45)
**System-Relevant Interventions**			
Quality of care	69 (7-187)	148 (0-420)	0.53 (0-1.61)
Referral	67 (8-174)	145 (0-426)	0.52 (0-1.54)
Transport	64 (4-165)	145 (0-412)	0.52 (0-1.45)
Targeted transfers	66 (6-169)	143 (0-399)	0.52 (0-1.46)
**Integrated Strategies**			
Family Planning	52 (0-137)	106 (0-318)	0.37 (0-1.13)
Community + Linkages	58 (0-162)	132 (0-407)	0.47 (0-1.47)
Facilities + Linkages	48 (0-144)	105 (0-373)	0.38 (0-1.34)
Facilities + Linkages + Quality	50 (0-148)	109 (0-372)	0.39 (0-1.33)
Comprehensive	35 (0-121)	70 (0-292)	0.25 (0-1.05)

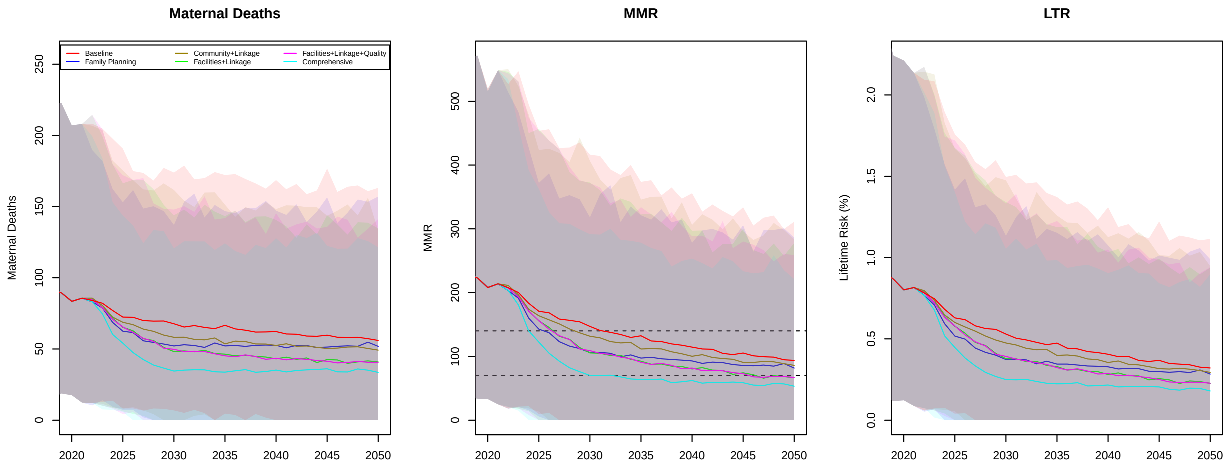

Ethiopia

ISO Code	Region	Area	Income Group
ETH	Eastern Africa	Africa	Low income

Projected Maternal Indicators in 2030 by Scenario

Scenario	Maternal Deaths	MMR	LTR
**Baseline**	31906 (10053-48219)	535 (191-747)	2.18 (0.7-3.42)
**Family Planning Interventions**			
Contraception	24696 (8470-39254)	497 (189-713)	1.62 (0.55-2.68)
Medical abortion	31695 (10172-47799)	531 (187-746)	2.17 (0.7-3.34)
**Community-Based Interventions**			
ANC	31671 (10327-47321)	531 (193-747)	2.16 (0.71-3.36)
SBA	29309 (10185-42660)	489 (191-666)	2 (0.71-2.97)
**Facility-Based Interventions**			
Facility births	11314 (7760-15641)	168 (111-231)	0.69 (0.44-0.99)
nonEmOC services	31834 (10086-48096)	533 (193-744)	2.18 (0.7-3.38)
bEmOC services	31775 (10234-47355)	533 (192-742)	2.17 (0.7-3.34)
cEmOC services	31682 (9784-47660)	531 (183-745)	2.17 (0.68-3.33)
**System-Relevant Interventions**			
Quality of care	29349 (5779-46105)	488 (93-717)	1.99 (0.36-3.24)
Referral	31718 (10206-47783)	532 (192-742)	2.17 (0.69-3.39)
Transport	28133 (10020-40950)	471 (191-643)	1.93 (0.7-2.87)
Targeted transfers	31878 (10167-47082)	534 (194-741)	2.18 (0.72-3.36)
**Integrated Strategies**			
Family Planning	24668 (8228-38552)	496 (185-712)	1.62 (0.52-2.64)
Community + Linkages	21130 (9981-29934)	346 (183-471)	1.42 (0.69-2.05)
Facilities + Linkages	10619 (7112-14471)	159 (104-218)	0.65 (0.39-0.94)
Facilities + Linkages + Quality	5569 (3706-8271)	70 (40-112)	0.29 (0.15-0.52)
Comprehensive	4315 (2453-6333)	64 (38-104)	0.21 (0.09-0.37)

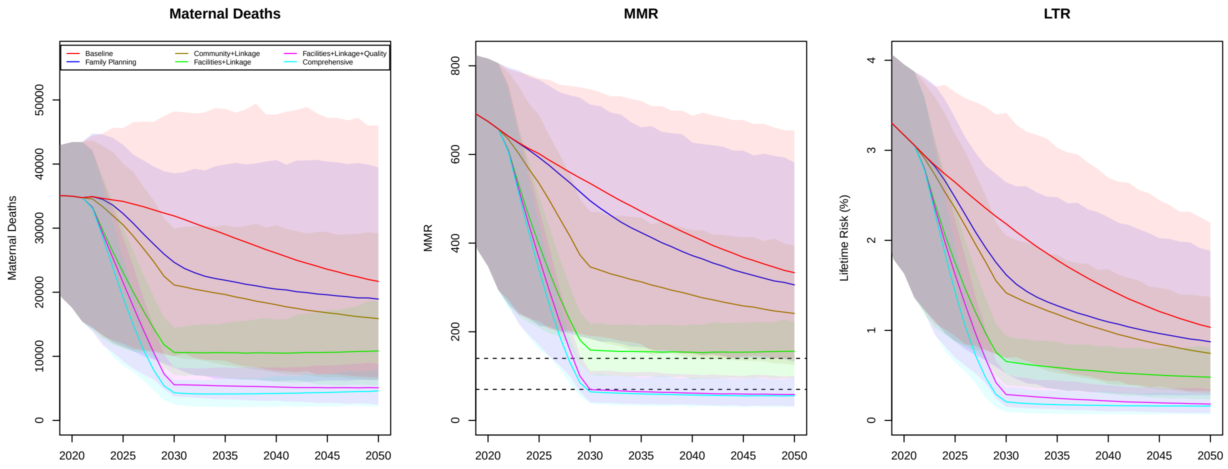

Faroe Islands

ISO Code	Region	Area	Income Group
FRO	Northern Europe	Europe	High income

Projected Maternal Indicators in 2030 by Scenario

Scenario	Maternal Deaths	MMR	LTR
**Baseline**	0 (0-2)	8 (0-84)	0.02 (0-0.28)
**Family Planning Interventions**			
Contraception	0 (0-2)	6 (0-101)	0.02 (0-0.27)
Medical abortion	0 (0-2)	7 (0-83)	0.02 (0-0.27)
**Community-Based Interventions**			
ANC	0 (0-2)	8 (0-84)	0.02 (0-0.27)
SBA	0 (0-2)	7 (0-86)	0.02 (0-0.28)
**Facility-Based Interventions**			
Facility births	0 (0-2)	7 (0-83)	0.02 (0-0.27)
nonEmOC services	0 (0-2)	8 (0-84)	0.02 (0-0.28)
bEmOC services	0 (0-2)	8 (0-84)	0.02 (0-0.28)
cEmOC services	0 (0-2)	8 (0-87)	0.02 (0-0.28)
**System-Relevant Interventions**			
Quality of care	0 (0-2)	8 (0-84)	0.02 (0-0.28)
Referral	0 (0-2)	8 (0-85)	0.02 (0-0.28)
Transport	0 (0-2)	8 (0-95)	0.02 (0-0.28)
Targeted transfers	0 (0-2)	8 (0-84)	0.02 (0-0.28)
**Integrated Strategies**			
Family Planning	0 (0-2)	6 (0-101)	0.02 (0-0.27)
Community + Linkages	0 (0-1)	7 (0-97)	0.02 (0-0.28)
Facilities + Linkages	0 (0-2)	7 (0-84)	0.02 (0-0.28)
Facilities + Linkages + Quality	0 (0-2)	7 (0-84)	0.02 (0-0.28)
Comprehensive	0 (0-1)	7 (0-102)	0.02 (0-0.27)

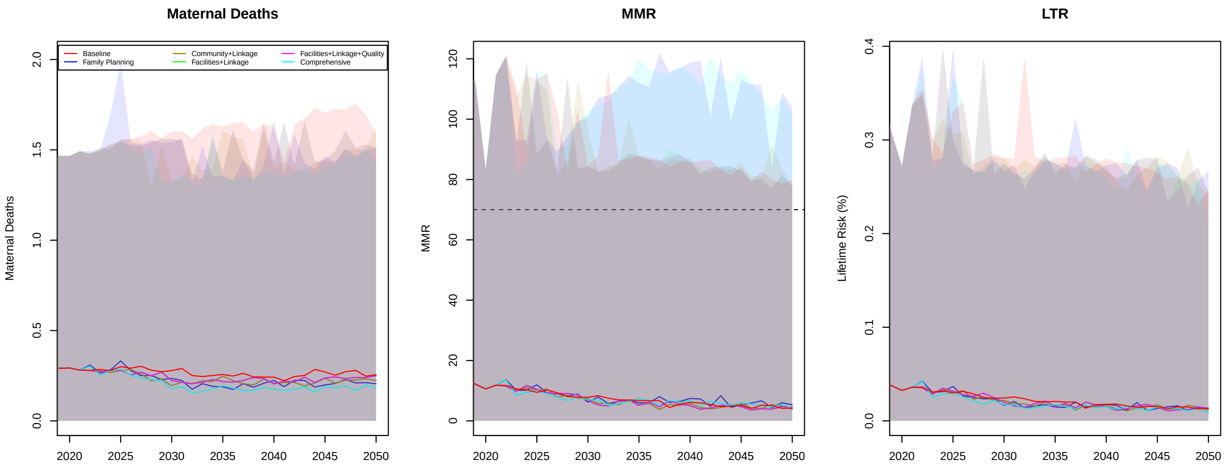

Fiji

ISO Code	Region	Area	Income Group
FJI	Melanesia	Oceania	Upper middle income

Projected Maternal Indicators in 2030 by Scenario

Scenario	Maternal Deaths	MMR	LTR
**Baseline**	25 (3-66)	99 (10-277)	0.29 (0.03-0.79)
**Family Planning Interventions**			
Contraception	22 (3-56)	90 (10-236)	0.25 (0.02-0.65)
Medical abortion	24 (5-55)	94 (10-243)	0.28 (0.03-0.68)
**Community-Based Interventions**			
ANC	24 (5-60)	95 (10-263)	0.28 (0.03-0.77)
SBA	24 (5-58)	94 (11-263)	0.27 (0.03-0.72)
**Facility-Based Interventions**			
Facility births	20 (2-48)	75 (0-189)	0.22 (0-0.57)
nonEmOC services	24 (3-59)	95 (10-248)	0.28 (0.03-0.75)
bEmOC services	25 (5-60)	99 (11-267)	0.29 (0.03-0.75)
cEmOC services	25 (5-67)	100 (10-280)	0.29 (0.03-0.81)
**System-Relevant Interventions**			
Quality of care	24 (4-59)	93 (9-266)	0.27 (0.03-0.75)
Referral	25 (5-61)	98 (11-263)	0.29 (0.03-0.76)
Transport	24 (4-63)	93 (11-268)	0.27 (0.03-0.76)
Targeted transfers	25 (5-64)	98 (10-275)	0.29 (0.03-0.79)
**Integrated Strategies**			
Family Planning	22 (4-52)	88 (10-225)	0.24 (0.03-0.62)
Community + Linkages	22 (5-50)	86 (10-202)	0.25 (0.03-0.59)
Facilities + Linkages	20 (2-48)	76 (0-202)	0.22 (0-0.6)
Facilities + Linkages + Quality	18 (0-43)	69 (0-179)	0.2 (0-0.5)
Comprehensive	15 (0-36)	57 (0-146)	0.16 (0-0.43)

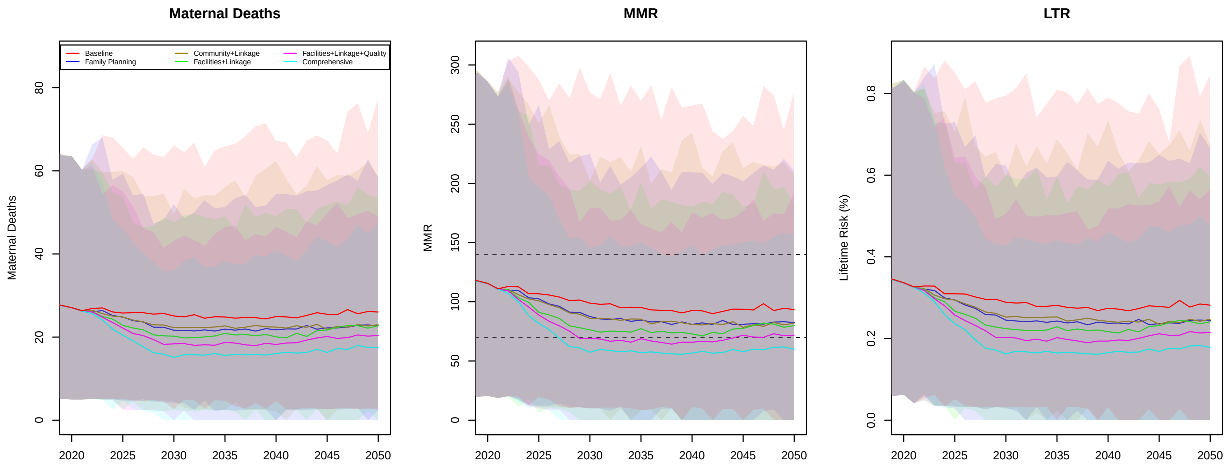

Finland

ISO Code	Region	Area	Income Group
FIN	Northern Europe	Europe	High income

Projected Maternal Indicators in 2030 by Scenario

Scenario	Maternal Deaths	MMR	LTR
**Baseline**	25 (0-63)	23 (0-64)	0.05 (0-0.14)
**Family Planning Interventions**			
Contraception	24 (0-58)	22 (0-64)	0.05 (0-0.14)
Medical abortion	25 (0-63)	23 (0-65)	0.05 (0-0.15)
**Community-Based Interventions**			
ANC	24 (0-60)	22 (0-61)	0.04 (0-0.13)
SBA	25 (0-62)	23 (0-65)	0.05 (0-0.15)
**Facility-Based Interventions**			
Facility births	25 (0-63)	23 (0-65)	0.05 (0-0.15)
nonEmOC services	25 (0-65)	23 (0-65)	0.05 (0-0.15)
bEmOC services	25 (0-62)	23 (0-64)	0.05 (0-0.14)
cEmOC services	24 (4-56)	22 (0-58)	0.04 (0-0.14)
**System-Relevant Interventions**			
Quality of care	25 (0-63)	23 (0-64)	0.05 (0-0.14)
Referral	25 (0-63)	23 (0-64)	0.05 (0-0.14)
Transport	24 (0-59)	22 (0-64)	0.04 (0-0.13)
Targeted transfers	25 (0-63)	23 (0-64)	0.05 (0-0.14)
**Integrated Strategies**			
Family Planning	24 (0-58)	22 (0-61)	0.04 (0-0.14)
Community + Linkages	24 (0-57)	22 (0-60)	0.04 (0-0.13)
Facilities + Linkages	25 (0-60)	24 (0-65)	0.05 (0-0.15)
Facilities + Linkages + Quality	25 (0-60)	24 (0-65)	0.05 (0-0.15)
Comprehensive	23 (0-57)	22 (0-64)	0.04 (0-0.14)

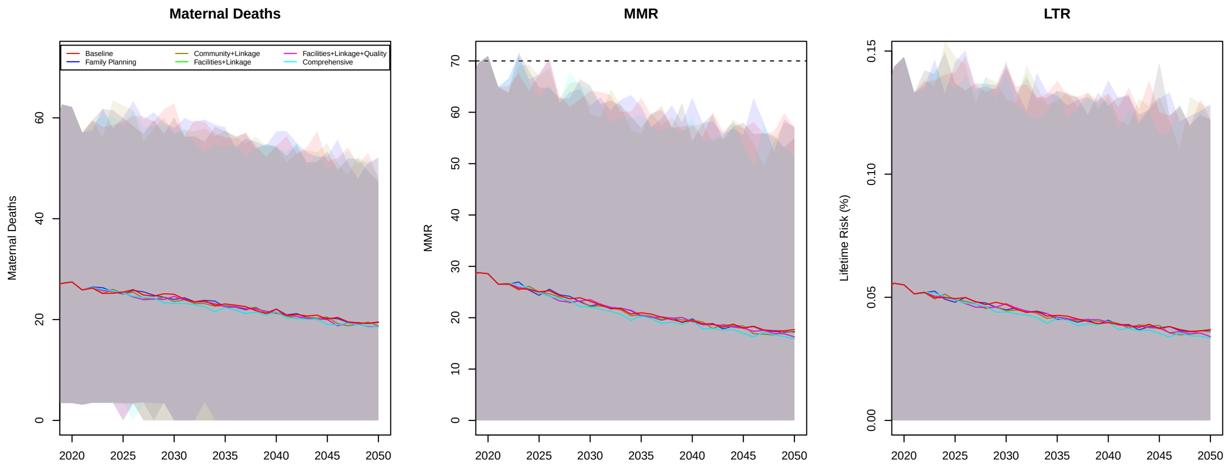

France

ISO Code	Region	Area	Income Group
FRA	Western Europe	Europe	High income

Projected Maternal Indicators in 2030 by Scenario

Scenario	Maternal Deaths	MMR	LTR
**Baseline**	194 (82-327)	9 (1-20)	0.02 (0-0.04)
**Family Planning Interventions**			
Contraception	188 (77-336)	8 (0-21)	0.02 (0-0.05)
Medical abortion	191 (82-326)	8 (1-20)	0.02 (0-0.04)
**Community-Based Interventions**			
ANC	184 (79-311)	8 (1-20)	0.02 (0-0.04)
SBA	194 (81-330)	8 (1-20)	0.02 (0-0.04)
**Facility-Based Interventions**			
Facility births	193 (83-326)	8 (1-20)	0.02 (0-0.04)
nonEmOC services	194 (82-327)	9 (1-20)	0.02 (0-0.04)
bEmOC services	194 (81-329)	8 (1-20)	0.02 (0-0.04)
cEmOC services	190 (78-327)	8 (1-19)	0.02 (0-0.04)
**System-Relevant Interventions**			
Quality of care	194 (81-329)	9 (1-21)	0.02 (0-0.04)
Referral	194 (83-327)	9 (1-20)	0.02 (0-0.04)
Transport	186 (80-318)	8 (1-19)	0.02 (0-0.04)
Targeted transfers	194 (82-327)	9 (1-21)	0.02 (0-0.04)
**Integrated Strategies**			
Family Planning	190 (81-319)	8 (1-22)	0.02 (0-0.05)
Community + Linkages	183 (77-311)	8 (1-20)	0.02 (0-0.04)
Facilities + Linkages	183 (74-318)	8 (0-19)	0.02 (0-0.04)
Facilities + Linkages + Quality	183 (74-318)	8 (1-18)	0.02 (0-0.04)
Comprehensive	177 (73-309)	8 (0-17)	0.02 (0-0.04)

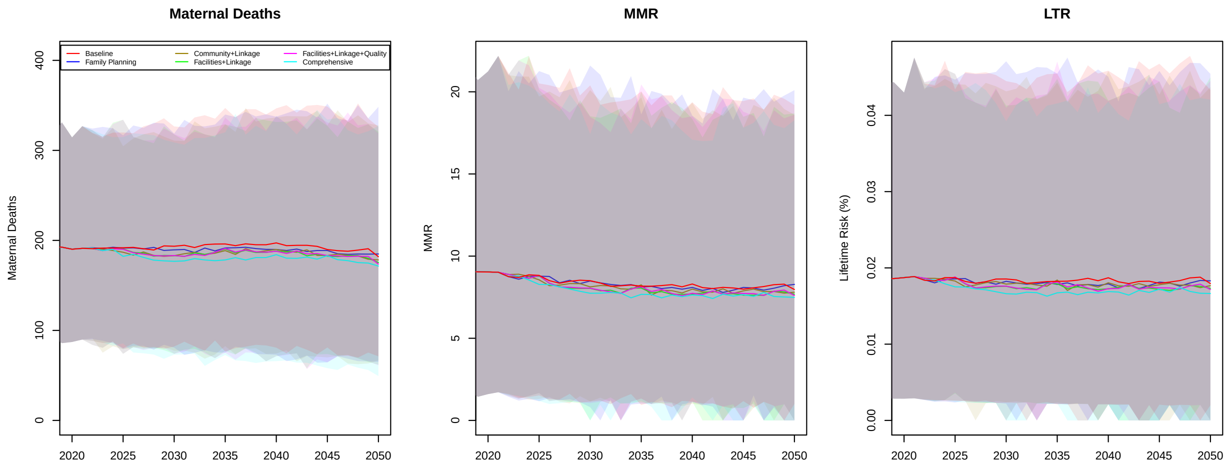

Gabon

ISO Code	Region	Area	Income Group
GAB	Middle Africa	Africa	Upper middle income

Projected Maternal Indicators in 2030 by Scenario

Scenario	Maternal Deaths	MMR	LTR
**Baseline**	158 (38-259)	162 (33-302)	0.7 (0.13-1.23)
**Family Planning Interventions**			
Contraception	136 (34-243)	145 (32-249)	0.6 (0.11-1.14)
Medical abortion	160 (37-269)	163 (37-305)	0.71 (0.14-1.27)
**Community-Based Interventions**			
ANC	158 (44-266)	162 (41-306)	0.71 (0.16-1.24)
SBA	158 (35-258)	162 (32-308)	0.7 (0.12-1.24)
**Facility-Based Interventions**			
Facility births	158 (39-260)	162 (33-306)	0.7 (0.13-1.22)
nonEmOC services	158 (39-262)	161 (32-298)	0.7 (0.13-1.22)
bEmOC services	159 (39-264)	163 (36-315)	0.71 (0.13-1.25)
cEmOC services	158 (39-274)	161 (38-306)	0.7 (0.13-1.26)
**System-Relevant Interventions**			
Quality of care	159 (40-267)	163 (37-302)	0.71 (0.14-1.25)
Referral	158 (40-260)	161 (35-295)	0.7 (0.12-1.23)
Transport	161 (37-272)	165 (37-316)	0.72 (0.13-1.27)
Targeted transfers	158 (43-265)	161 (37-298)	0.7 (0.13-1.22)
**Integrated Strategies**			
Family Planning	134 (31-235)	142 (32-248)	0.59 (0.11-1.1)
Community + Linkages	158 (42-273)	161 (36-296)	0.71 (0.14-1.26)
Facilities + Linkages	156 (39-265)	160 (37-303)	0.7 (0.14-1.23)
Facilities + Linkages + Quality	155 (42-260)	159 (39-300)	0.7 (0.14-1.2)
Comprehensive	133 (36-237)	142 (31-244)	0.59 (0.1-1.11)

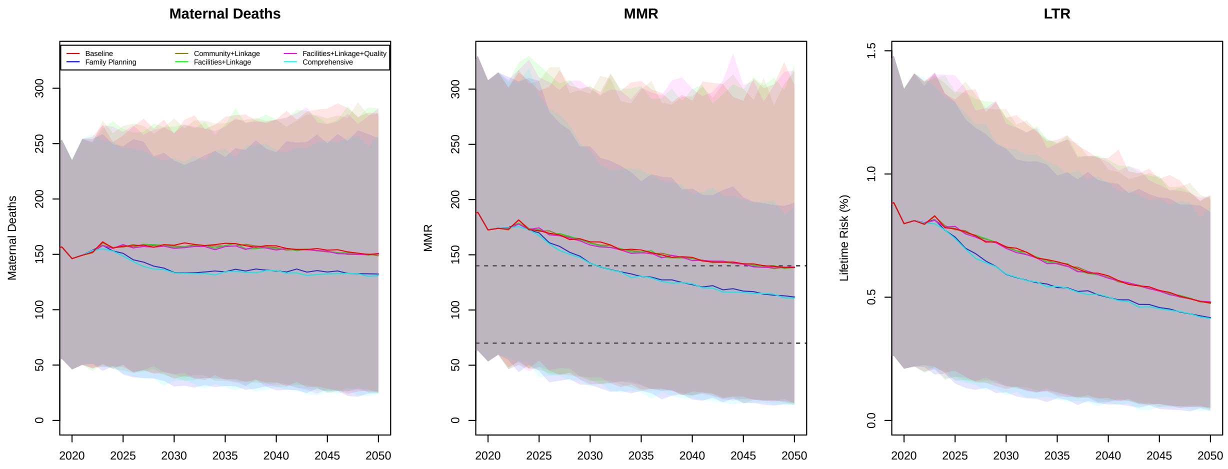

Gambia

ISO Code	Region	Area	Income Group
GMB	Western Africa	Africa	Low income

Projected Maternal Indicators in 2030 by Scenario

Scenario	Maternal Deaths	MMR	LTR
**Baseline**	569 (318-1045)	317 (159-839)	1.64 (0.83-2.99)
**Family Planning Interventions**			
Contraception	515 (283-798)	276 (144-470)	1.47 (0.73-2.31)
Medical abortion	524 (282-794)	284 (147-491)	1.5 (0.79-2.39)
**Community-Based Interventions**			
ANC	559 (308-1075)	308 (157-773)	1.6 (0.79-3.21)
SBA	557 (296-1018)	310 (145-813)	1.61 (0.79-3.11)
**Facility-Based Interventions**			
Facility births	401 (162-877)	215 (70-722)	1.04 (0.3-2.67)
nonEmOC services	564 (311-1030)	314 (154-807)	1.63 (0.84-3.3)
bEmOC services	570 (313-985)	318 (160-778)	1.66 (0.82-3.17)
cEmOC services	566 (299-1012)	315 (149-803)	1.64 (0.81-3.15)
**System-Relevant Interventions**			
Quality of care	416 (214-878)	225 (95-688)	1.16 (0.51-2.71)
Referral	565 (316-1027)	315 (157-824)	1.63 (0.82-3.15)
Transport	514 (266-957)	283 (138-770)	1.45 (0.67-2.87)
Targeted transfers	570 (321-978)	318 (158-793)	1.64 (0.84-3)
**Integrated Strategies**			
Family Planning	497 (279-765)	264 (140-429)	1.42 (0.7-2.25)
Community + Linkages	463 (237-850)	252 (114-681)	1.28 (0.6-2.61)
Facilities + Linkages	381 (148-828)	203 (60-703)	0.98 (0.28-2.42)
Facilities + Linkages + Quality	201 (47-608)	95 (7-502)	0.4 (0-1.82)
Comprehensive	138 (30-279)	49 (0-127)	0.2 (0-0.61)

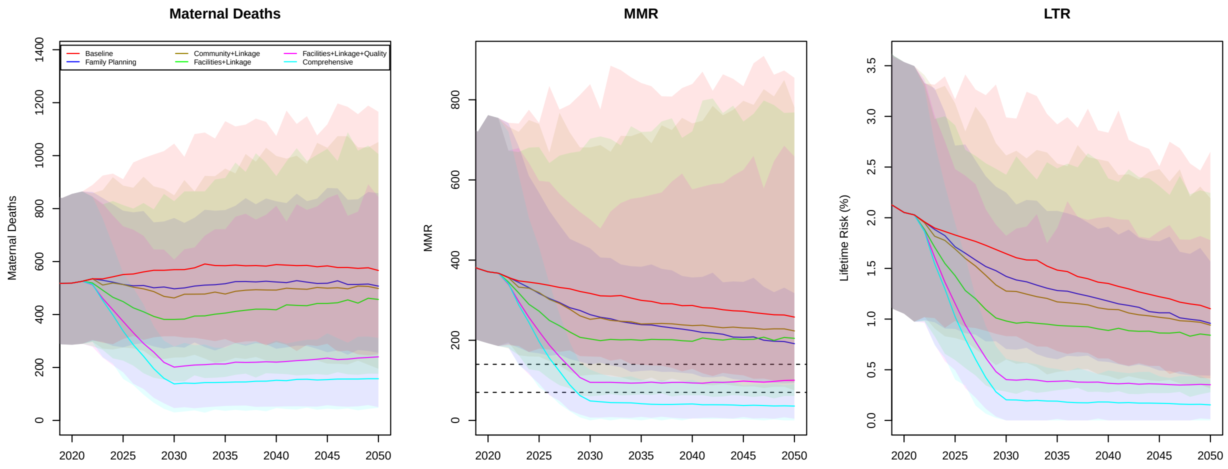

Georgia

ISO Code	Region	Area	Income Group
GEO	Western Asia	Asia	Lower middle income

Projected Maternal Indicators in 2030 by Scenario

Scenario	Maternal Deaths	MMR	LTR
**Baseline**	35 (0-111)	48 (0-210)	0.08 (0-0.36)
**Family Planning Interventions**			
Contraception	36 (0-113)	50 (0-207)	0.09 (0-0.39)
Medical abortion	37 (0-109)	49 (0-209)	0.08 (0-0.35)
**Community-Based Interventions**			
ANC	35 (0-103)	50 (0-212)	0.09 (0-0.36)
SBA	36 (0-114)	52 (0-220)	0.09 (0-0.39)
**Facility-Based Interventions**			
Facility births	35 (0-108)	48 (0-196)	0.08 (0-0.34)
nonEmOC services	35 (0-112)	48 (0-210)	0.08 (0-0.35)
bEmOC services	35 (0-112)	49 (0-209)	0.08 (0-0.36)
cEmOC services	36 (0-112)	50 (0-211)	0.09 (0-0.38)
**System-Relevant Interventions**			
Quality of care	27 (0-89)	27 (0-150)	0.04 (0-0.24)
Referral	35 (0-112)	48 (0-196)	0.08 (0-0.33)
Transport	36 (0-112)	51 (0-194)	0.09 (0-0.34)
Targeted transfers	35 (0-111)	49 (0-210)	0.08 (0-0.36)
**Integrated Strategies**			
Family Planning	35 (0-113)	49 (0-201)	0.08 (0-0.37)
Community + Linkages	33 (0-104)	47 (0-213)	0.08 (0-0.35)
Facilities + Linkages	31 (0-105)	44 (0-204)	0.08 (0-0.36)
Facilities + Linkages + Quality	20 (0-72)	20 (0-110)	0.03 (0-0.2)
Comprehensive	20 (0-76)	21 (0-131)	0.03 (0-0.19)

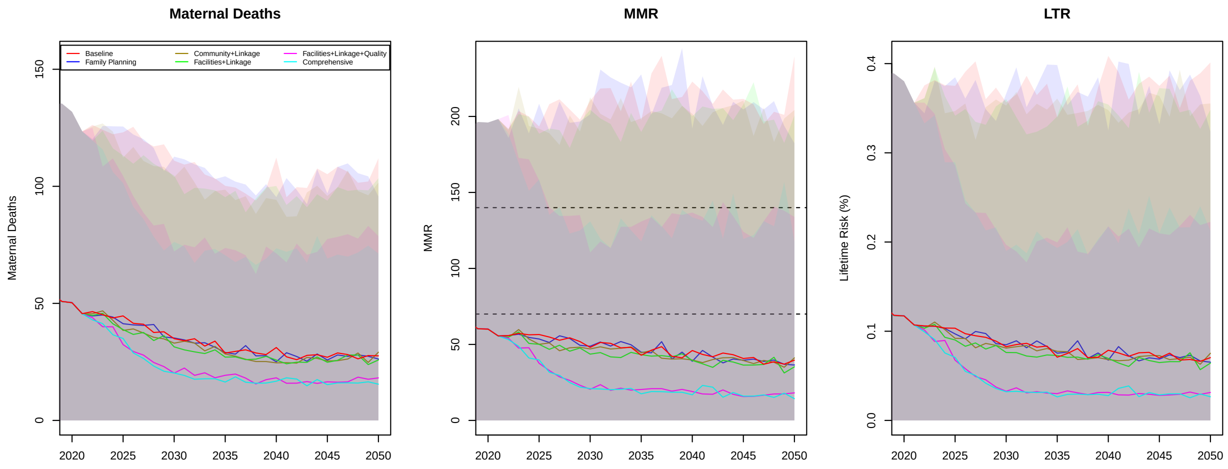

Germany

ISO Code	Region	Area	Income Group
DEU	Western Europe	Europe	High income

Projected Maternal Indicators in 2030 by Scenario

Scenario	Maternal Deaths	MMR	LTR
**Baseline**	152 (43-301)	9 (0-27)	0.01 (0-0.04)
**Family Planning Interventions**			
Contraception	150 (45-301)	9 (0-25)	0.01 (0-0.04)
Medical abortion	151 (43-301)	9 (0-27)	0.01 (0-0.04)
**Community-Based Interventions**			
ANC	148 (27-298)	9 (0-24)	0.01 (0-0.04)
SBA	151 (42-301)	9 (0-27)	0.01 (0-0.04)
**Facility-Based Interventions**			
Facility births	151 (44-301)	9 (0-27)	0.01 (0-0.04)
nonEmOC services	152 (43-301)	9 (0-27)	0.01 (0-0.04)
bEmOC services	151 (43-301)	9 (0-27)	0.01 (0-0.04)
cEmOC services	150 (27-292)	9 (0-24)	0.01 (0-0.04)
**System-Relevant Interventions**			
Quality of care	152 (43-308)	9 (0-27)	0.01 (0-0.04)
Referral	152 (43-301)	9 (0-27)	0.01 (0-0.04)
Transport	145 (38-304)	9 (0-25)	0.01 (0-0.04)
Targeted transfers	152 (44-303)	9 (0-27)	0.01 (0-0.04)
**Integrated Strategies**			
Family Planning	149 (43-301)	9 (0-25)	0.01 (0-0.04)
Community + Linkages	141 (26-295)	9 (0-25)	0.01 (0-0.04)
Facilities + Linkages	143 (39-302)	9 (0-25)	0.01 (0-0.04)
Facilities + Linkages + Quality	143 (39-299)	9 (0-25)	0.01 (0-0.04)
Comprehensive	138 (27-278)	9 (0-23)	0.01 (0-0.04)

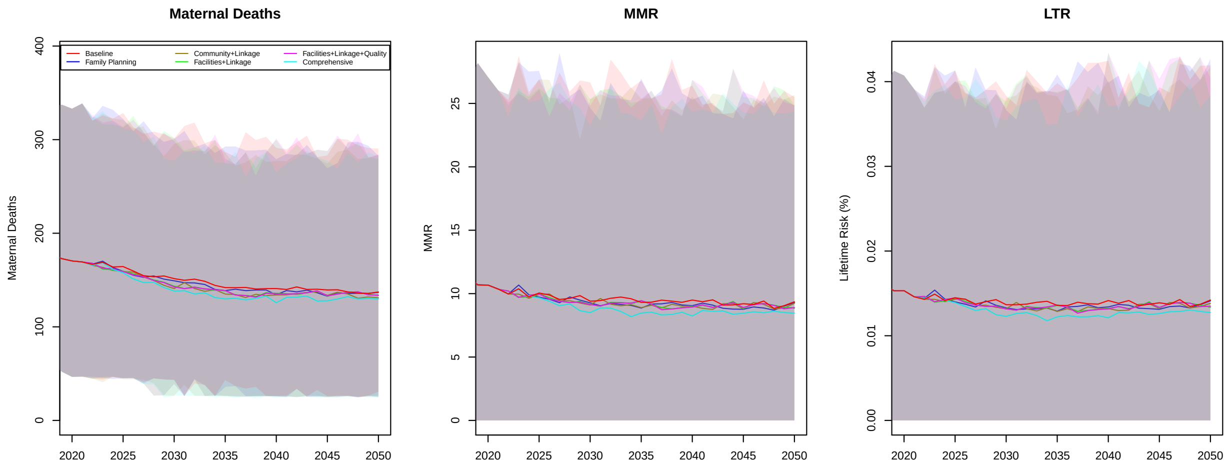

Ghana

ISO Code	Region	Area	Income Group
GHA	Western Africa	Africa	Lower middle income

Projected Maternal Indicators in 2030 by Scenario

Scenario	Maternal Deaths	MMR	LTR
**Baseline**	3152 (1275-5212)	204 (119-309)	0.83 (0.3-1.46)
**Family Planning Interventions**			
Contraception	2872 (1309-5117)	194 (111-285)	0.75 (0.33-1.4)
Medical abortion	3107 (1248-5254)	200 (121-300)	0.82 (0.31-1.45)
**Community-Based Interventions**			
ANC	3083 (1191-5091)	199 (114-298)	0.81 (0.29-1.42)
SBA	3011 (1222-5056)	194 (117-292)	0.79 (0.3-1.4)
**Facility-Based Interventions**			
Facility births	2522 (1076-4487)	159 (101-265)	0.64 (0.26-1.21)
nonEmOC services	3142 (1227-5186)	203 (117-308)	0.83 (0.3-1.46)
bEmOC services	3146 (1250-5203)	203 (118-305)	0.83 (0.31-1.44)
cEmOC services	3040 (1153-5095)	196 (113-307)	0.8 (0.29-1.42)
**System-Relevant Interventions**			
Quality of care	2253 (636-4193)	140 (46-256)	0.6 (0.14-1.17)
Referral	3130 (1238-5167)	202 (117-308)	0.83 (0.3-1.43)
Transport	3009 (1194-5008)	195 (115-295)	0.79 (0.3-1.41)
Targeted transfers	3152 (1216-5149)	204 (117-307)	0.84 (0.29-1.45)
**Integrated Strategies**			
Family Planning	2858 (1337-5056)	193 (110-284)	0.75 (0.33-1.39)
Community + Linkages	2802 (1165-4891)	180 (110-285)	0.73 (0.29-1.35)
Facilities + Linkages	2370 (983-4324)	149 (93-255)	0.6 (0.25-1.19)
Facilities + Linkages + Quality	1397 (474-3359)	80 (25-207)	0.34 (0.09-0.94)
Comprehensive	1176 (385-2987)	67 (21-168)	0.28 (0.06-0.82)

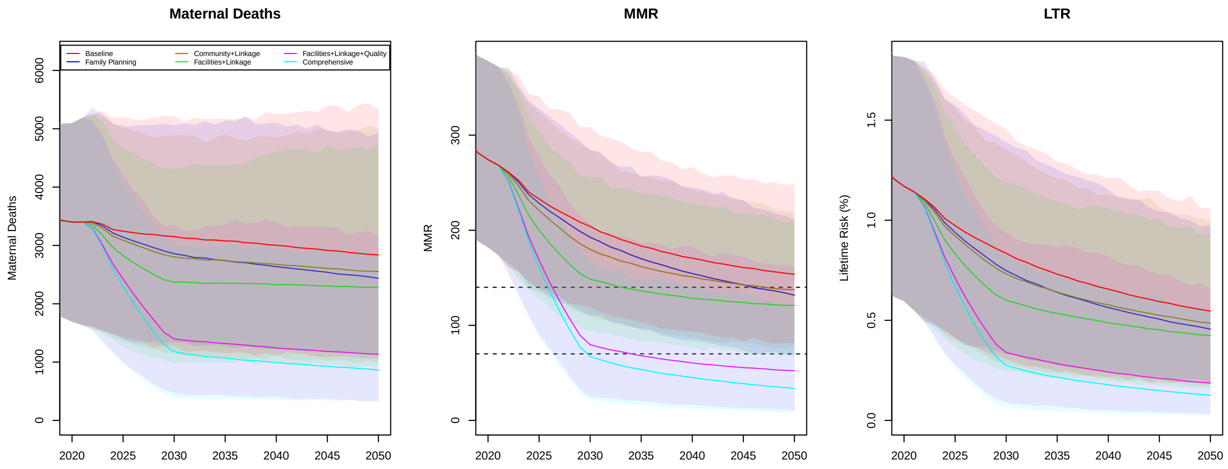

Greece

ISO Code	Region	Area	Income Group
GRC	Southern Europe	Europe	High income

Projected Maternal Indicators in 2030 by Scenario

Scenario	Maternal Deaths	MMR	LTR
**Baseline**	34 (0-80)	25 (0-73)	0.03 (0-0.1)
**Family Planning Interventions**			
Contraception	33 (0-85)	25 (0-73)	0.03 (0-0.1)
Medical abortion	33 (0-76)	25 (0-70)	0.03 (0-0.1)
**Community-Based Interventions**			
ANC	34 (0-78)	24 (0-78)	0.03 (0-0.1)
SBA	34 (0-80)	25 (0-73)	0.03 (0-0.1)
**Facility-Based Interventions**			
Facility births	34 (0-80)	25 (0-73)	0.03 (0-0.1)
nonEmOC services	33 (0-80)	25 (0-73)	0.03 (0-0.1)
bEmOC services	34 (0-80)	25 (0-73)	0.03 (0-0.1)
cEmOC services	34 (0-82)	25 (0-76)	0.03 (0-0.1)
**System-Relevant Interventions**			
Quality of care	34 (0-80)	25 (0-73)	0.03 (0-0.1)
Referral	34 (0-80)	25 (0-73)	0.03 (0-0.1)
Transport	34 (0-79)	25 (0-74)	0.03 (0-0.1)
Targeted transfers	34 (0-80)	25 (0-73)	0.03 (0-0.1)
**Integrated Strategies**			
Family Planning	34 (0-85)	25 (0-72)	0.03 (0-0.1)
Community + Linkages	33 (0-79)	25 (0-77)	0.03 (0-0.11)
Facilities + Linkages	33 (0-81)	25 (0-79)	0.03 (0-0.11)
Facilities + Linkages + Quality	33 (0-81)	25 (0-79)	0.03 (0-0.11)
Comprehensive	32 (0-80)	24 (0-71)	0.03 (0-0.1)

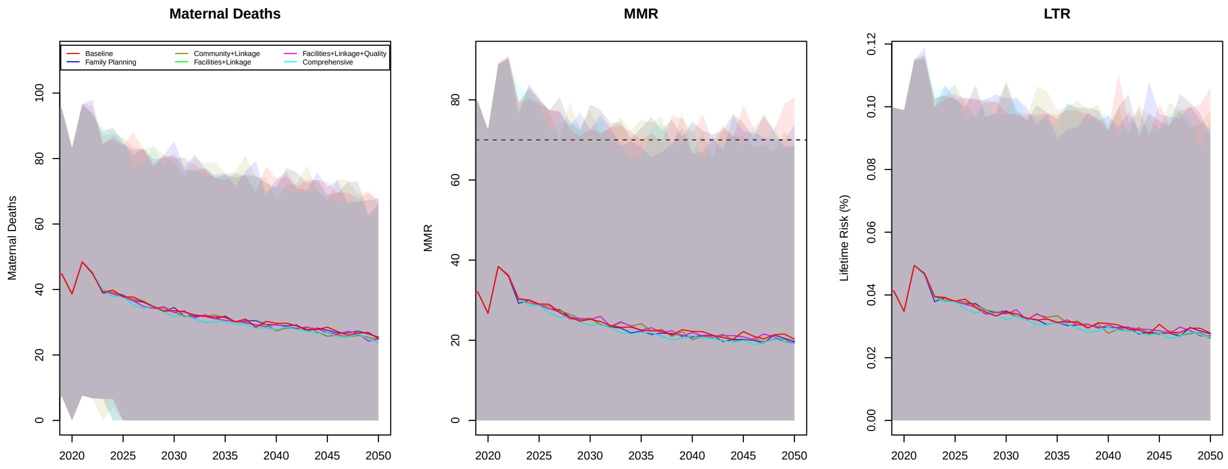

Greenland

ISO Code	Region	Area	Income Group
GRL	Northern America	Northern America	High income

Projected Maternal Indicators in 2030 by Scenario

Scenario	Maternal Deaths	MMR	LTR
**Baseline**	0 (0-1)	10 (0-190)	0.02 (0-0.36)
**Family Planning Interventions**			
Contraception	0 (0-1)	12 (0-199)	0.02 (0-0.34)
Medical abortion	0 (0-1)	13 (0-204)	0.02 (0-0.37)
**Community-Based Interventions**			
ANC	0 (0-1)	10 (0-190)	0.02 (0-0.36)
SBA	0 (0-1)	10 (0-190)	0.02 (0-0.36)
**Facility-Based Interventions**			
Facility births	0 (0-1)	10 (0-190)	0.02 (0-0.36)
nonEmOC services	0 (0-1)	10 (0-190)	0.02 (0-0.36)
bEmOC services	0 (0-1)	10 (0-190)	0.02 (0-0.36)
cEmOC services	0 (0-1)	10 (0-198)	0.02 (0-0.36)
**System-Relevant Interventions**			
Quality of care	0 (0-1)	9 (0-188)	0.02 (0-0.34)
Referral	0 (0-1)	10 (0-190)	0.02 (0-0.36)
Transport	0 (0-1)	10 (0-195)	0.02 (0-0.36)
Targeted transfers	0 (0-1)	10 (0-190)	0.02 (0-0.36)
**Integrated Strategies**			
Family Planning	0 (0-1)	12 (0-205)	0.02 (0-0.35)
Community + Linkages	0 (0-1)	9 (0-193)	0.02 (0-0.35)
Facilities + Linkages	0 (0-1)	10 (0-198)	0.02 (0-0.36)
Facilities + Linkages + Quality	0 (0-1)	9 (0-193)	0.02 (0-0.36)
Comprehensive	0 (0-1)	10 (0-207)	0.02 (0-0.35)

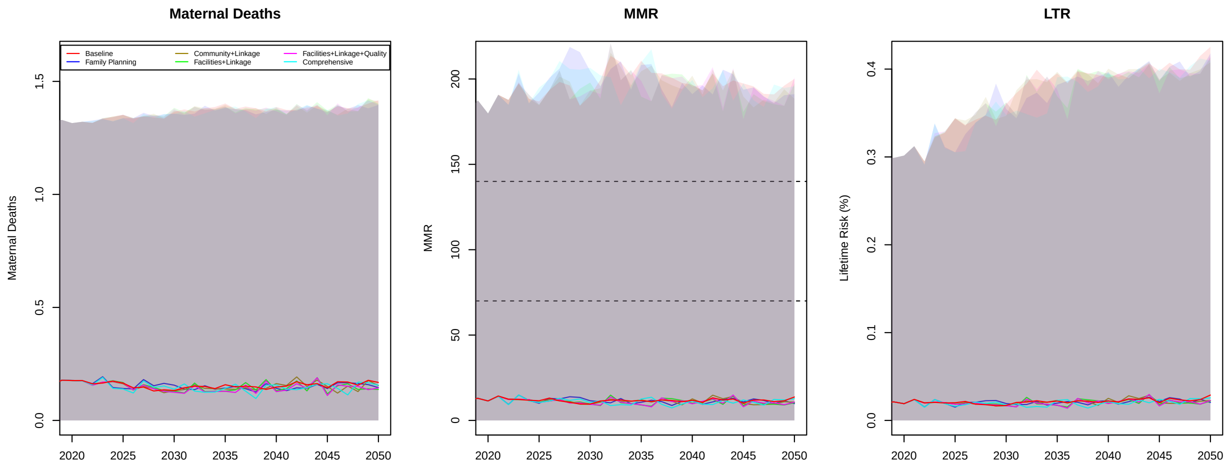

Grenada

ISO Code	Region	Area	Income Group
GRD	Caribbean	Latin America and the Caribbean	Upper middle income

Projected Maternal Indicators in 2030 by Scenario

Scenario	Maternal Deaths	MMR	LTR
**Baseline**	2 (0-10)	108 (0-492)	0.22 (0-0.99)
**Family Planning Interventions**			
Contraception	2 (0-8)	96 (0-399)	0.19 (0-0.77)
Medical abortion	2 (0-7)	80 (0-331)	0.16 (0-0.7)
**Community-Based Interventions**			
ANC	2 (0-9)	100 (0-448)	0.2 (0-0.89)
SBA	2 (0-9)	97 (0-440)	0.2 (0-0.94)
**Facility-Based Interventions**			
Facility births	2 (0-7)	73 (0-365)	0.15 (0-0.75)
nonEmOC services	2 (0-10)	109 (0-483)	0.22 (0-0.97)
bEmOC services	2 (0-10)	108 (0-492)	0.22 (0-0.99)
cEmOC services	2 (0-10)	110 (0-505)	0.22 (0-1.01)
**System-Relevant Interventions**			
Quality of care	2 (0-10)	105 (0-481)	0.21 (0-0.96)
Referral	2 (0-10)	111 (0-505)	0.23 (0-0.99)
Transport	2 (0-8)	99 (0-372)	0.2 (0-0.8)
Targeted transfers	2 (0-10)	109 (0-492)	0.22 (0-0.99)
**Integrated Strategies**			
Family Planning	2 (0-7)	69 (0-354)	0.14 (0-0.68)
Community + Linkages	2 (0-8)	87 (0-394)	0.18 (0-0.78)
Facilities + Linkages	2 (0-8)	74 (0-382)	0.15 (0-0.77)
Facilities + Linkages + Quality	2 (0-7)	66 (0-349)	0.14 (0-0.74)
Comprehensive	1 (0-5)	33 (0-228)	0.07 (0-0.47)

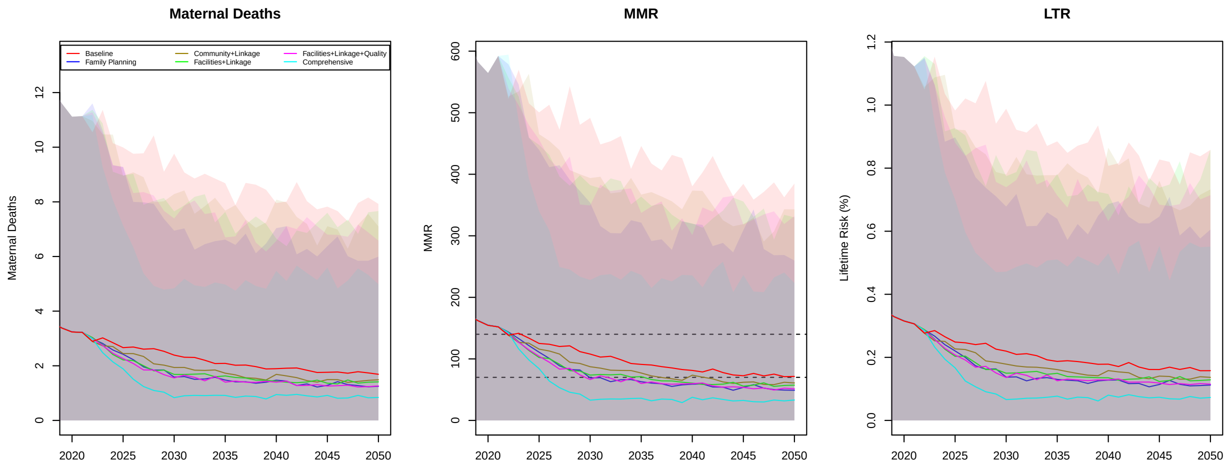

Guatemala

ISO Code	Region	Area	Income Group
GTM	Central America	Latin America and the Caribbean	Upper middle income

Projected Maternal Indicators in 2030 by Scenario

Scenario	Maternal Deaths	MMR	LTR
**Baseline**	641 (262-1125)	117 (42-233)	0.32 (0.11-0.6)
**Family Planning Interventions**			
Contraception	460 (152-870)	95 (31-193)	0.22 (0.06-0.44)
Medical abortion	635 (264-1080)	114 (46-211)	0.32 (0.12-0.57)
**Community-Based Interventions**			
ANC	633 (250-1103)	115 (43-231)	0.32 (0.11-0.59)
SBA	601 (240-1051)	107 (36-213)	0.3 (0.09-0.57)
**Facility-Based Interventions**			
Facility births	409 (114-844)	68 (8-167)	0.19 (0.02-0.46)
nonEmOC services	649 (264-1153)	118 (46-232)	0.33 (0.12-0.61)
bEmOC services	647 (265-1125)	117 (43-228)	0.33 (0.11-0.59)
cEmOC services	644 (282-1121)	117 (44-227)	0.32 (0.11-0.6)
**System-Relevant Interventions**			
Quality of care	643 (268-1134)	117 (48-232)	0.32 (0.12-0.59)
Referral	636 (267-1089)	116 (47-215)	0.32 (0.12-0.58)
Transport	589 (239-1079)	107 (40-218)	0.3 (0.1-0.57)
Targeted transfers	645 (270-1140)	117 (45-237)	0.32 (0.12-0.61)
**Integrated Strategies**			
Family Planning	455 (165-853)	93 (28-180)	0.22 (0.06-0.45)
Community + Linkages	515 (186-965)	91 (28-191)	0.25 (0.07-0.5)
Facilities + Linkages	398 (98-828)	67 (9-172)	0.19 (0.02-0.44)
Facilities + Linkages + Quality	386 (88-827)	65 (7-165)	0.18 (0.02-0.44)
Comprehensive	299 (57-680)	55 (7-138)	0.13 (0.01-0.35)

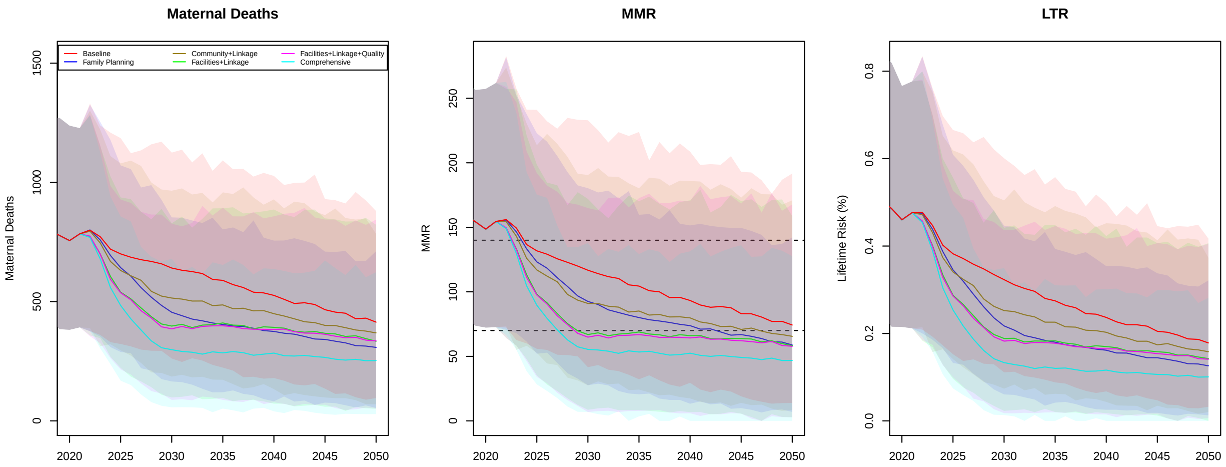

Guinea

ISO Code	Region	Area	Income Group
GIN	Western Africa	Africa	Low income

Projected Maternal Indicators in 2030 by Scenario

Scenario	Maternal Deaths	MMR	LTR
**Baseline**	5094 (2699-7427)	651 (337-958)	3.3 (1.59-4.83)
**Family Planning Interventions**			
Contraception	4932 (2781-7095)	629 (352-919)	3.18 (1.69-4.68)
Medical abortion	4892 (2677-7218)	622 (333-901)	3.14 (1.6-4.82)
**Community-Based Interventions**			
ANC	4972 (2579-7215)	633 (316-942)	3.21 (1.66-4.72)
SBA	4881 (2660-7168)	621 (344-908)	3.16 (1.66-4.68)
**Facility-Based Interventions**			
Facility births	2789 (1100-4636)	337 (116-604)	1.75 (0.56-3.11)
nonEmOC services	5084 (2775-7390)	649 (342-961)	3.29 (1.72-4.84)
bEmOC services	5101 (2652-7482)	651 (337-959)	3.29 (1.59-4.93)
cEmOC services	5111 (2727-7455)	653 (336-958)	3.32 (1.74-4.91)
**System-Relevant Interventions**			
Quality of care	4578 (2070-6811)	581 (243-884)	2.96 (1.18-4.55)
Referral	5014 (2467-7255)	639 (313-937)	3.23 (1.51-4.85)
Transport	4590 (2283-6665)	585 (284-881)	2.97 (1.4-4.4)
Targeted transfers	5057 (2731-7341)	644 (346-927)	3.27 (1.67-4.8)
**Integrated Strategies**			
Family Planning	4795 (2576-7116)	610 (318-899)	3.08 (1.59-4.64)
Community + Linkages	3804 (1845-5734)	477 (232-736)	2.43 (1.11-3.84)
Facilities + Linkages	2661 (933-4705)	320 (102-575)	1.67 (0.51-3.04)
Facilities + Linkages + Quality	1825 (594-3582)	211 (49-459)	1.13 (0.21-2.53)
Comprehensive	1502 (446-2926)	166 (33-348)	0.89 (0.12-2)

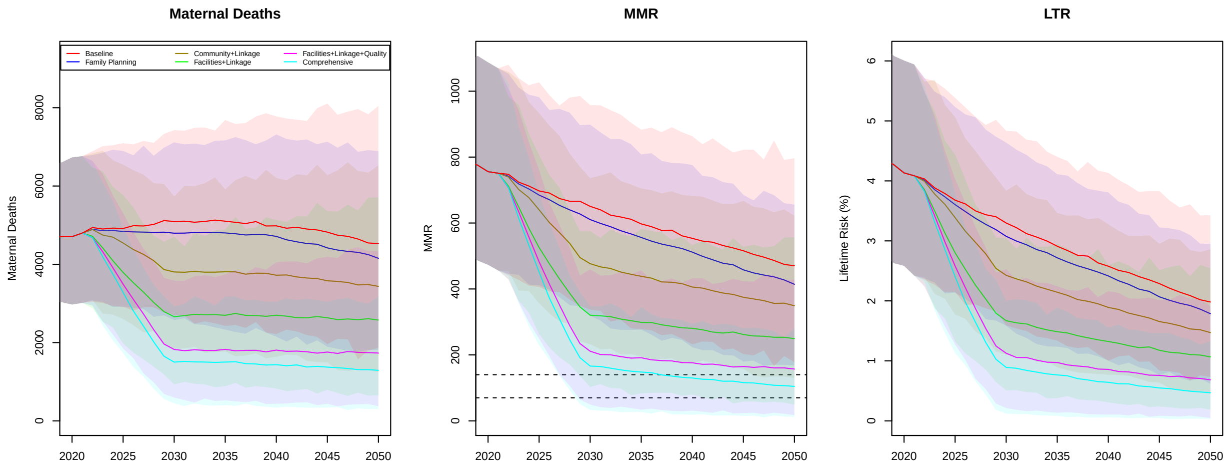

Guinea-Bissau

ISO Code	Region	Area	Income Group
GNB	Western Africa	Africa	Low income

Projected Maternal Indicators in 2030 by Scenario

Scenario	Maternal Deaths	MMR	LTR
**Baseline**	659 (344-1050)	618 (309-1002)	2.8 (1.36-4.65)
**Family Planning Interventions**			
Contraception	409 (217-653)	525 (269-884)	1.68 (0.8-2.72)
Medical abortion	625 (327-964)	582 (292-908)	2.63 (1.31-4.12)
**Community-Based Interventions**			
ANC	632 (320-982)	591 (288-951)	2.68 (1.3-4.48)
SBA	624 (322-967)	583 (291-942)	2.65 (1.23-4.28)
**Facility-Based Interventions**			
Facility births	404 (186-703)	364 (171-650)	1.67 (0.68-3.09)
nonEmOC services	661 (341-1058)	621 (288-1002)	2.81 (1.35-4.62)
bEmOC services	658 (328-1038)	617 (289-970)	2.8 (1.31-4.48)
cEmOC services	651 (334-1021)	609 (298-984)	2.76 (1.35-4.51)
**System-Relevant Interventions**			
Quality of care	509 (189-879)	469 (134-853)	2.12 (0.65-3.92)
Referral	652 (334-1021)	612 (294-993)	2.77 (1.33-4.63)
Transport	607 (324-963)	567 (286-895)	2.58 (1.29-4.45)
Targeted transfers	657 (339-1032)	616 (291-985)	2.79 (1.35-4.68)
**Integrated Strategies**			
Family Planning	399 (198-638)	507 (271-797)	1.62 (0.79-2.64)
Community + Linkages	519 (264-831)	480 (245-788)	2.18 (1.09-3.8)
Facilities + Linkages	379 (174-690)	341 (150-642)	1.56 (0.64-3.04)
Facilities + Linkages + Quality	148 (47-387)	114 (24-343)	0.53 (0.1-1.66)
Comprehensive	72 (23-174)	63 (11-168)	0.2 (0.02-0.68)

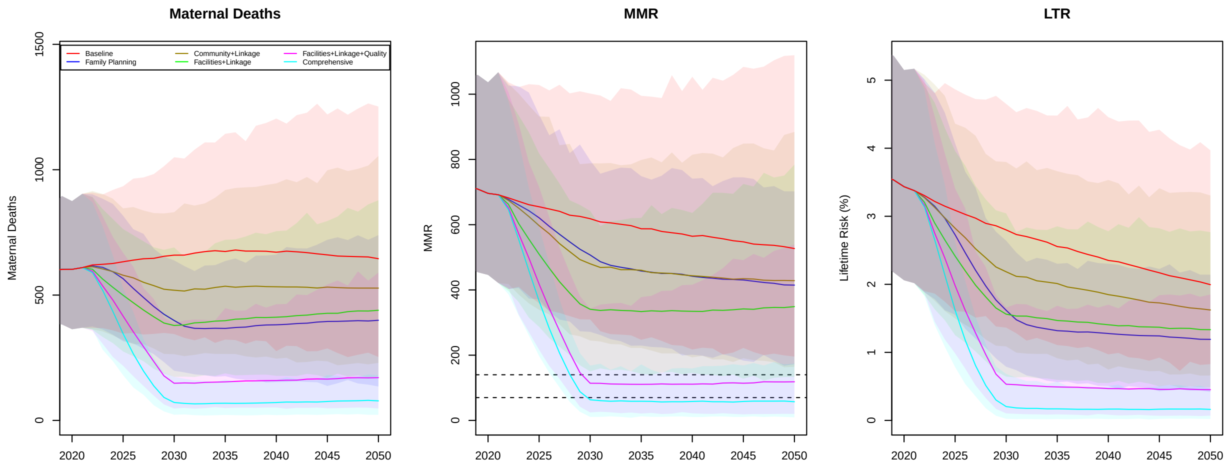

Guyana

ISO Code	Region	Area	Income Group
GUY	South America	Latin America and the Caribbean	Upper middle income

Projected Maternal Indicators in 2030 by Scenario

Scenario	Maternal Deaths	MMR	LTR
**Baseline**	17 (0-51)	98 (0-293)	0.23 (0-0.73)
**Family Planning Interventions**			
Contraception	15 (0-46)	93 (0-305)	0.2 (0-0.66)
Medical abortion	17 (0-51)	98 (0-293)	0.23 (0-0.73)
**Community-Based Interventions**			
ANC	16 (0-49)	92 (0-308)	0.22 (0-0.74)
SBA	16 (0-48)	93 (0-284)	0.22 (0-0.7)
**Facility-Based Interventions**			
Facility births	16 (0-48)	90 (0-294)	0.21 (0-0.69)
nonEmOC services	17 (0-49)	98 (0-290)	0.23 (0-0.72)
bEmOC services	17 (0-52)	98 (0-297)	0.23 (0-0.74)
cEmOC services	17 (0-50)	99 (0-306)	0.23 (0-0.73)
**System-Relevant Interventions**			
Quality of care	17 (0-52)	98 (0-297)	0.23 (0-0.73)
Referral	17 (0-49)	98 (0-289)	0.23 (0-0.7)
Transport	16 (0-47)	93 (0-297)	0.22 (0-0.71)
Targeted transfers	17 (0-50)	95 (0-297)	0.22 (0-0.73)
**Integrated Strategies**			
Family Planning	15 (0-46)	93 (0-305)	0.2 (0-0.66)
Community + Linkages	16 (0-51)	93 (0-313)	0.22 (0-0.76)
Facilities + Linkages	15 (0-48)	85 (0-301)	0.2 (0-0.72)
Facilities + Linkages + Quality	15 (0-48)	86 (0-295)	0.2 (0-0.72)
Comprehensive	13 (0-41)	80 (0-279)	0.17 (0-0.6)

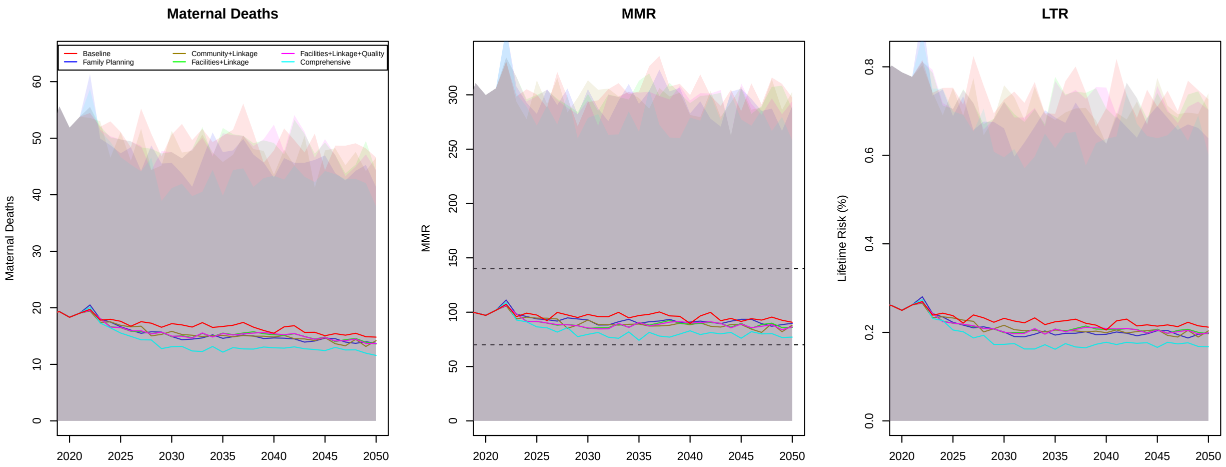

Haiti

ISO Code	Region	Area	Income Group
HTI	Caribbean	Latin America and the Caribbean	Low income

Projected Maternal Indicators in 2030 by Scenario

Scenario	Maternal Deaths	MMR	LTR
**Baseline**	1737 (1025-2745)	489 (254-752)	1.47 (0.84-2.31)
**Family Planning Interventions**			
Contraception	1535 (871-2666)	427 (212-714)	1.26 (0.67-2.2)
Medical abortion	1726 (1004-2665)	487 (249-753)	1.46 (0.85-2.29)
**Community-Based Interventions**			
ANC	1682 (1024-2589)	472 (231-738)	1.42 (0.82-2.26)
SBA	1620 (978-2538)	453 (240-717)	1.36 (0.78-2.17)
**Facility-Based Interventions**			
Facility births	825 (352-1539)	210 (77-447)	0.62 (0.23-1.28)
nonEmOC services	1723 (995-2712)	484 (249-748)	1.46 (0.85-2.3)
bEmOC services	1736 (1030-2682)	488 (256-746)	1.47 (0.84-2.31)
cEmOC services	1676 (995-2578)	472 (245-730)	1.42 (0.83-2.18)
**System-Relevant Interventions**			
Quality of care	1591 (865-2511)	445 (210-713)	1.34 (0.72-2.12)
Referral	1686 (983-2580)	474 (240-747)	1.42 (0.8-2.19)
Transport	1581 (941-2488)	442 (221-696)	1.33 (0.76-2.14)
Targeted transfers	1728 (1017-2758)	486 (247-754)	1.46 (0.83-2.31)
**Integrated Strategies**			
Family Planning	1531 (840-2681)	426 (215-703)	1.26 (0.66-2.15)
Community + Linkages	1296 (768-2067)	356 (182-588)	1.07 (0.61-1.75)
Facilities + Linkages	702 (284-1342)	173 (64-377)	0.51 (0.17-1.08)
Facilities + Linkages + Quality	528 (225-1089)	120 (42-283)	0.36 (0.12-0.89)
Comprehensive	483 (222-1028)	106 (42-252)	0.31 (0.11-0.77)

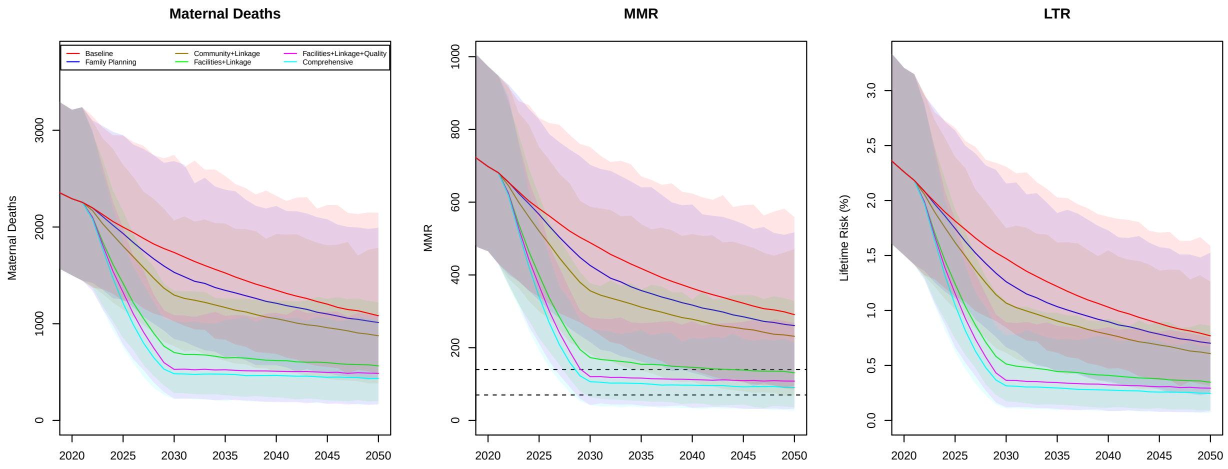

Honduras

ISO Code	Region	Area	Income Group
HND	Central America	Latin America and the Caribbean	Lower middle income

Projected Maternal Indicators in 2030 by Scenario

Scenario	Maternal Deaths	MMR	LTR
**Baseline**	328 (88-645)	90 (17-196)	0.23 (0.03-0.53)
**Family Planning Interventions**			
Contraception	292 (73-596)	86 (14-187)	0.2 (0.03-0.46)
Medical abortion	336 (99-678)	91 (18-188)	0.23 (0.04-0.5)
**Community-Based Interventions**			
ANC	331 (93-643)	90 (18-205)	0.23 (0.04-0.52)
SBA	320 (91-651)	87 (17-200)	0.22 (0.03-0.53)
**Facility-Based Interventions**			
Facility births	285 (62-606)	73 (8-174)	0.19 (0-0.47)
nonEmOC services	332 (96-657)	91 (18-196)	0.23 (0.04-0.54)
bEmOC services	329 (100-658)	90 (18-197)	0.23 (0.04-0.53)
cEmOC services	306 (79-614)	81 (12-182)	0.21 (0.02-0.49)
**System-Relevant Interventions**			
Quality of care	270 (70-576)	69 (8-160)	0.18 (0.02-0.44)
Referral	330 (76-655)	89 (12-204)	0.23 (0.03-0.54)
Transport	323 (85-641)	90 (18-195)	0.23 (0.04-0.51)
Targeted transfers	327 (96-637)	90 (19-192)	0.23 (0.04-0.53)
**Integrated Strategies**			
Family Planning	297 (72-591)	86 (15-195)	0.2 (0.03-0.48)
Community + Linkages	301 (73-621)	82 (12-181)	0.21 (0.03-0.49)
Facilities + Linkages	246 (42-553)	61 (0-157)	0.16 (0-0.42)
Facilities + Linkages + Quality	173 (0-429)	38 (0-120)	0.1 (0-0.33)
Comprehensive	155 (0-376)	36 (0-109)	0.08 (0-0.28)

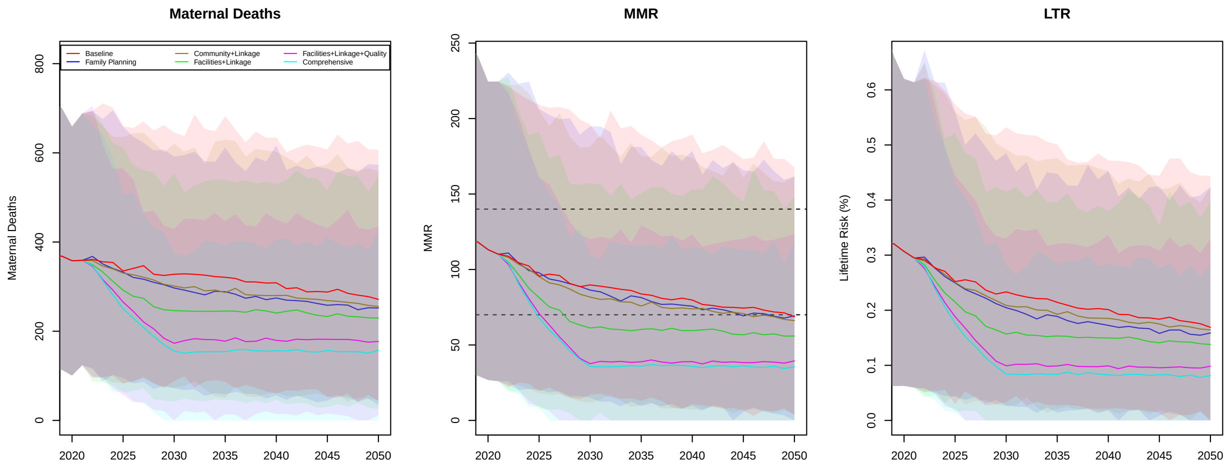

Hungary

ISO Code	Region	Area	Income Group
HUN	Eastern Europe	Europe	High income

Projected Maternal Indicators in 2030 by Scenario

Scenario	Maternal Deaths	MMR	LTR
**Baseline**	31 (4-94)	26 (0-85)	0.03 (0-0.13)
**Family Planning Interventions**			
Contraception	30 (4-74)	24 (0-77)	0.03 (0-0.11)
Medical abortion	32 (4-94)	26 (0-87)	0.04 (0-0.14)
**Community-Based Interventions**			
ANC	31 (4-90)	25 (0-80)	0.03 (0-0.12)
SBA	31 (4-94)	26 (0-84)	0.03 (0-0.13)
**Facility-Based Interventions**			
Facility births	31 (4-95)	26 (0-85)	0.03 (0-0.14)
nonEmOC services	31 (4-94)	26 (0-85)	0.03 (0-0.13)
bEmOC services	31 (4-92)	26 (0-85)	0.03 (0-0.13)
cEmOC services	31 (4-88)	26 (0-85)	0.04 (0-0.13)
**System-Relevant Interventions**			
Quality of care	31 (4-94)	25 (0-85)	0.03 (0-0.13)
Referral	31 (4-94)	26 (0-85)	0.03 (0-0.13)
Transport	30 (2-78)	25 (0-83)	0.03 (0-0.12)
Targeted transfers	31 (4-94)	26 (0-85)	0.03 (0-0.13)
**Integrated Strategies**			
Family Planning	31 (4-78)	25 (0-78)	0.03 (0-0.11)
Community + Linkages	31 (4-82)	25 (0-79)	0.03 (0-0.12)
Facilities + Linkages	30 (4-83)	25 (0-83)	0.03 (0-0.13)
Facilities + Linkages + Quality	30 (4-83)	25 (0-83)	0.03 (0-0.13)
Comprehensive	29 (4-76)	23 (0-72)	0.03 (0-0.1)

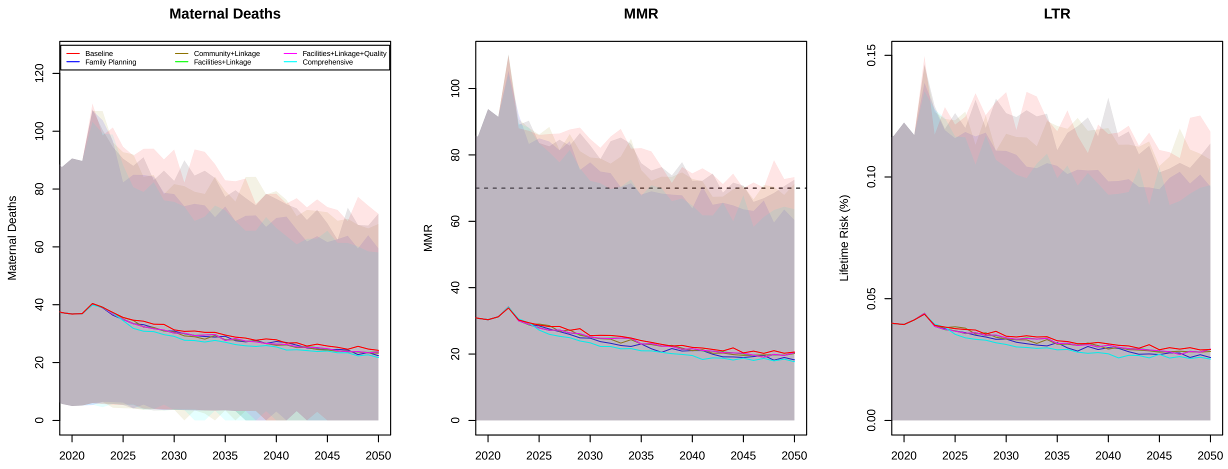

Iceland

ISO Code	Region	Area	Income Group
ISL	Northern Europe	Europe	High income

Projected Maternal Indicators in 2030 by Scenario

Scenario	Maternal Deaths	MMR	LTR
**Baseline**	2 (0-12)	25 (0-179)	0.06 (0-0.46)
**Family Planning Interventions**			
Contraception	2 (0-12)	25 (0-171)	0.06 (0-0.28)
Medical abortion	2 (0-12)	25 (0-179)	0.06 (0-0.46)
**Community-Based Interventions**			
ANC	2 (0-12)	23 (0-170)	0.05 (0-0.46)
SBA	2 (0-12)	25 (0-174)	0.06 (0-0.45)
**Facility-Based Interventions**			
Facility births	2 (0-12)	26 (0-174)	0.06 (0-0.45)
nonEmOC services	2 (0-12)	25 (0-179)	0.06 (0-0.46)
bEmOC services	2 (0-12)	25 (0-179)	0.06 (0-0.46)
cEmOC services	2 (0-12)	24 (0-167)	0.06 (0-0.46)
**System-Relevant Interventions**			
Quality of care	2 (0-12)	25 (0-173)	0.06 (0-0.43)
Referral	2 (0-12)	25 (0-179)	0.06 (0-0.46)
Transport	2 (0-12)	25 (0-176)	0.06 (0-0.39)
Targeted transfers	2 (0-12)	25 (0-179)	0.06 (0-0.46)
**Integrated Strategies**			
Family Planning	2 (0-12)	26 (0-175)	0.06 (0-0.28)
Community + Linkages	2 (0-12)	24 (0-165)	0.06 (0-0.27)
Facilities + Linkages	2 (0-12)	25 (0-176)	0.06 (0-0.4)
Facilities + Linkages + Quality	2 (0-12)	25 (0-179)	0.06 (0-0.4)
Comprehensive	2 (0-12)	23 (0-169)	0.05 (0-0.28)

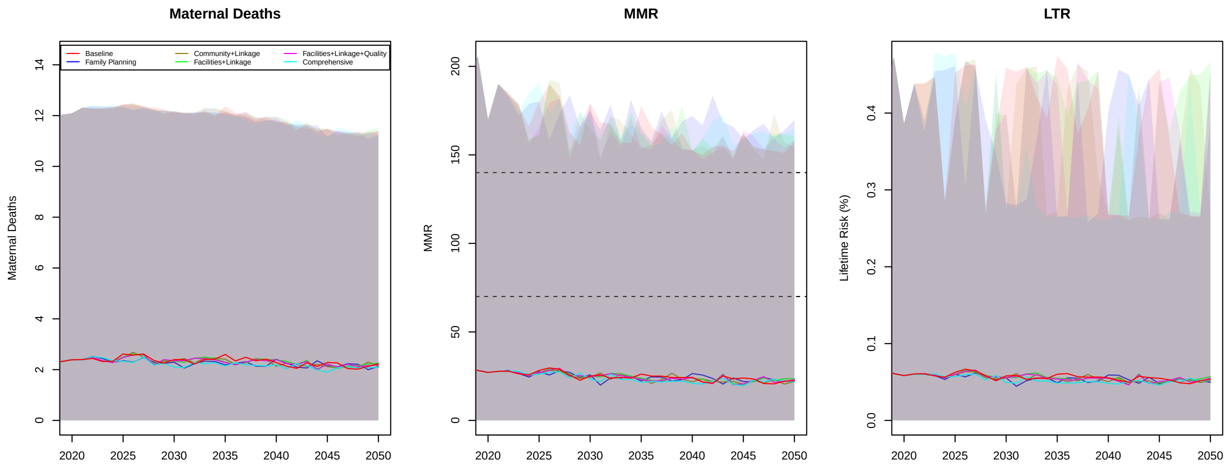

India

ISO Code	Region	Area	Income Group
IND	Southern Asia	Asia	Lower middle income

Projected Maternal Indicators in 2030 by Scenario

Scenario	Maternal Deaths	MMR	LTR
**Baseline**	23186 (12914-40138)	46 (25-94)	0.11 (0.05-0.25)
**Family Planning Interventions**			
Contraception	19909 (11939-35897)	42 (21-90)	0.09 (0.04-0.22)
Medical abortion	23280 (13310-40751)	46 (25-91)	0.11 (0.05-0.25)
**Community-Based Interventions**			
ANC	22432 (12788-38753)	44 (25-86)	0.11 (0.05-0.24)
SBA	21917 (13030-36480)	42 (23-78)	0.1 (0.05-0.2)
**Facility-Based Interventions**			
Facility births	18041 (10171-30336)	28 (14-59)	0.07 (0.03-0.16)
nonEmOC services	23290 (12979-39928)	46 (25-94)	0.11 (0.05-0.26)
bEmOC services	23197 (13190-39988)	46 (26-91)	0.11 (0.05-0.25)
cEmOC services	22179 (12303-39740)	42 (23-91)	0.1 (0.04-0.26)
**System-Relevant Interventions**			
Quality of care	23084 (13061-40322)	46 (25-92)	0.11 (0.05-0.26)
Referral	23076 (12683-40197)	46 (25-93)	0.11 (0.05-0.25)
Transport	20701 (12044-35426)	42 (24-84)	0.1 (0.05-0.22)
Targeted transfers	23030 (13350-40191)	46 (24-93)	0.11 (0.05-0.25)
**Integrated Strategies**			
Family Planning	20128 (12221-35264)	43 (23-90)	0.09 (0.04-0.22)
Community + Linkages	18452 (10712-30737)	35 (20-67)	0.09 (0.04-0.19)
Facilities + Linkages	15320 (8817-25341)	23 (10-50)	0.06 (0.02-0.13)
Facilities + Linkages + Quality	14963 (8618-25346)	23 (11-52)	0.06 (0.02-0.14)
Comprehensive	12123 (6853-19812)	18 (7-39)	0.04 (0.01-0.09)

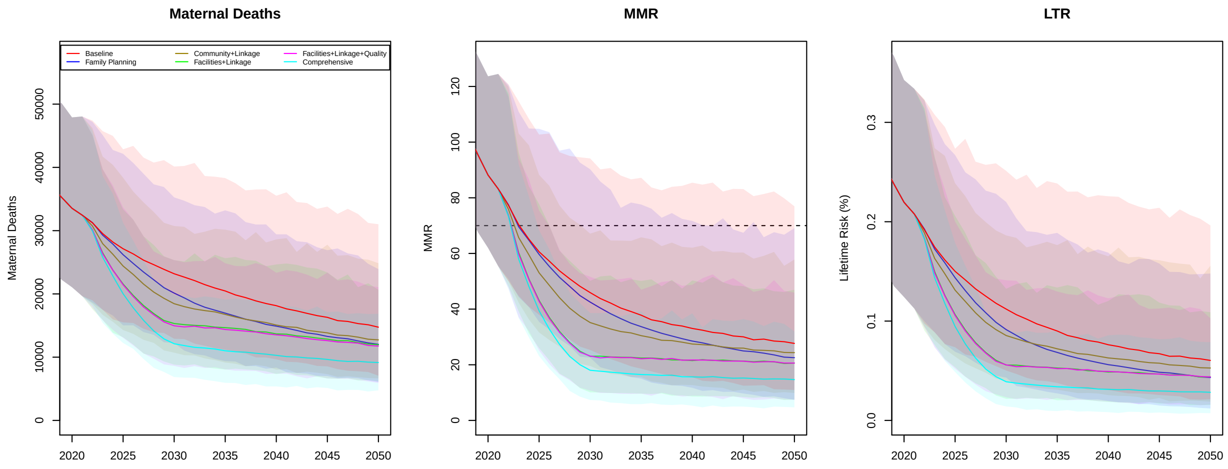

Indonesia

ISO Code	Region	Area	Income Group
IDN	South-Eastern Asia	Asia	Lower middle income

Projected Maternal Indicators in 2030 by Scenario

Scenario	Maternal Deaths	MMR	LTR
**Baseline**	2788 (1304-5686)	45 (15-86)	0.08 (0.03-0.19)
**Family Planning Interventions**			
Contraception	3037 (1271-5651)	46 (13-99)	0.09 (0.02-0.2)
Medical abortion	2815 (1242-5877)	45 (14-87)	0.08 (0.02-0.19)
**Community-Based Interventions**			
ANC	2731 (1221-5578)	44 (13-82)	0.08 (0.02-0.18)
SBA	2637 (1291-5098)	41 (15-78)	0.07 (0.02-0.16)
**Facility-Based Interventions**			
Facility births	2376 (1115-4509)	35 (14-64)	0.06 (0.02-0.14)
nonEmOC services	2797 (1273-5772)	45 (15-86)	0.08 (0.02-0.19)
bEmOC services	2814 (1242-5950)	45 (15-87)	0.08 (0.03-0.19)
cEmOC services	2731 (1196-5659)	44 (13-82)	0.08 (0.02-0.18)
**System-Relevant Interventions**			
Quality of care	2564 (1202-5091)	40 (14-80)	0.07 (0.02-0.17)
Referral	2772 (1276-5610)	45 (15-83)	0.08 (0.02-0.18)
Transport	2539 (1123-5064)	42 (15-76)	0.07 (0.02-0.16)
Targeted transfers	2794 (1304-5724)	45 (15-84)	0.08 (0.03-0.19)
**Integrated Strategies**			
Family Planning	3012 (1251-5756)	46 (14-101)	0.09 (0.02-0.2)
Community + Linkages	2335 (1034-4149)	37 (12-66)	0.06 (0.02-0.14)
Facilities + Linkages	2126 (1051-3989)	31 (12-59)	0.06 (0.02-0.13)
Facilities + Linkages + Quality	1883 (917-3182)	26 (9-51)	0.04 (0.01-0.1)
Comprehensive	1802 (913-3053)	22 (7-43)	0.04 (0.01-0.09)

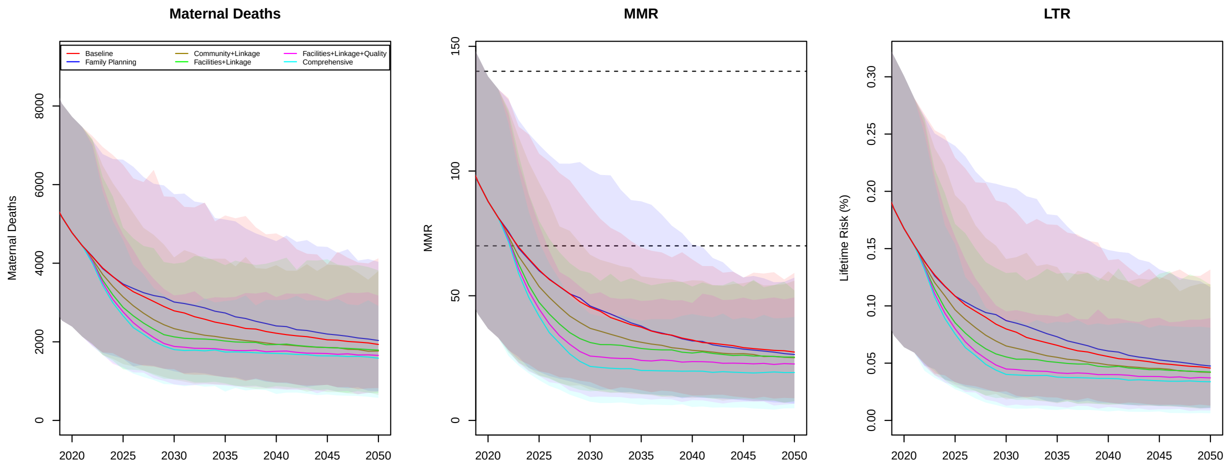

Iran (Islamic Republic of)

ISO Code	Region	Area	Income Group
IRN	Southern Asia	Asia	Upper middle income

Projected Maternal Indicators in 2030 by Scenario

Scenario	Maternal Deaths	MMR	LTR
**Baseline**	427 (65-994)	19 (0-60)	0.03 (0-0.1)
**Family Planning Interventions**			
Contraception	406 (65-921)	15 (0-47)	0.03 (0-0.09)
Medical abortion	421 (66-972)	19 (0-55)	0.03 (0-0.1)
**Community-Based Interventions**			
ANC	407 (34-890)	17 (0-54)	0.03 (0-0.08)
SBA	425 (65-951)	18 (0-61)	0.03 (0-0.1)
**Facility-Based Interventions**			
Facility births	413 (65-939)	18 (0-58)	0.03 (0-0.09)
nonEmOC services	427 (65-994)	19 (0-60)	0.03 (0-0.1)
bEmOC services	428 (66-975)	19 (0-60)	0.03 (0-0.1)
cEmOC services	418 (94-936)	18 (0-57)	0.03 (0-0.09)
**System-Relevant Interventions**			
Quality of care	423 (93-979)	19 (0-60)	0.03 (0-0.1)
Referral	425 (64-994)	19 (0-60)	0.03 (0-0.1)
Transport	401 (65-972)	18 (0-60)	0.03 (0-0.1)
Targeted transfers	416 (64-894)	18 (0-58)	0.03 (0-0.09)
**Integrated Strategies**			
Family Planning	396 (63-915)	14 (0-42)	0.02 (0-0.08)
Community + Linkages	392 (66-929)	18 (0-56)	0.03 (0-0.09)
Facilities + Linkages	399 (64-928)	18 (0-62)	0.03 (0-0.09)
Facilities + Linkages + Quality	394 (64-924)	18 (0-60)	0.03 (0-0.09)
Comprehensive	356 (32-829)	13 (0-42)	0.02 (0-0.08)

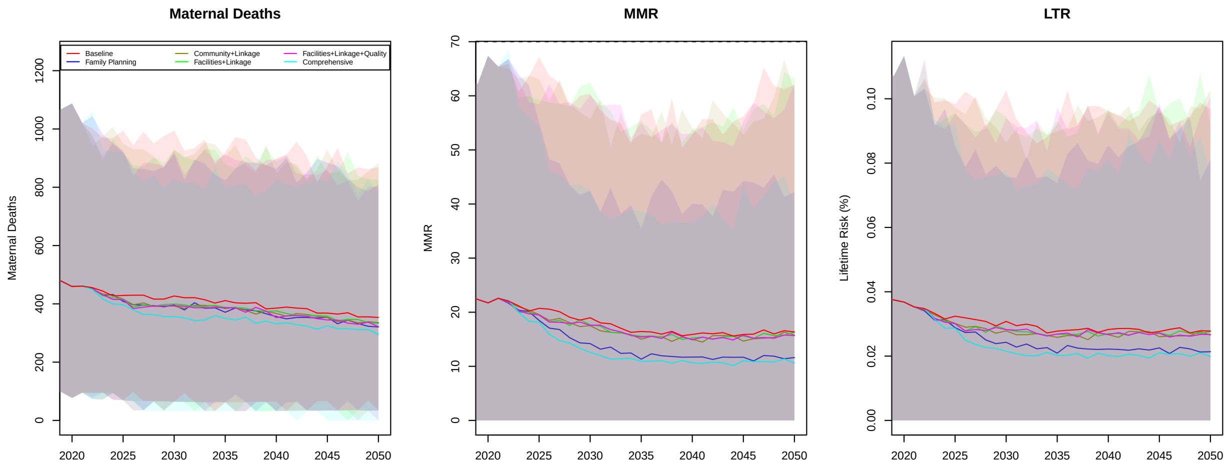

Iraq

ISO Code	Region	Area	Income Group
IRQ	Western Asia	Asia	Upper middle income

Projected Maternal Indicators in 2030 by Scenario

Scenario	Maternal Deaths	MMR	LTR
**Baseline**	801 (307-1452)	23 (5-47)	0.11 (0.02-0.24)
**Family Planning Interventions**			
Contraception	754 (265-1447)	22 (5-48)	0.1 (0.02-0.24)
Medical abortion	789 (309-1444)	23 (6-46)	0.11 (0.02-0.24)
**Community-Based Interventions**			
ANC	787 (302-1434)	23 (5-45)	0.11 (0.02-0.24)
SBA	795 (305-1462)	23 (5-48)	0.11 (0.02-0.24)
**Facility-Based Interventions**			
Facility births	796 (305-1465)	23 (6-47)	0.11 (0.02-0.24)
nonEmOC services	803 (305-1452)	23 (5-48)	0.11 (0.02-0.24)
bEmOC services	800 (292-1464)	23 (5-49)	0.11 (0.02-0.25)
cEmOC services	800 (319-1504)	23 (6-47)	0.11 (0.02-0.24)
**System-Relevant Interventions**			
Quality of care	748 (279-1421)	21 (4-44)	0.1 (0.02-0.23)
Referral	799 (303-1449)	23 (6-47)	0.11 (0.02-0.24)
Transport	772 (293-1421)	23 (5-46)	0.11 (0.02-0.24)
Targeted transfers	787 (288-1429)	23 (6-49)	0.11 (0.02-0.24)
**Integrated Strategies**			
Family Planning	760 (257-1430)	22 (5-48)	0.1 (0.02-0.25)
Community + Linkages	738 (298-1413)	22 (5-48)	0.1 (0.02-0.24)
Facilities + Linkages	736 (247-1377)	22 (4-46)	0.1 (0.01-0.23)
Facilities + Linkages + Quality	676 (239-1320)	19 (4-42)	0.09 (0.01-0.22)
Comprehensive	630 (216-1192)	18 (4-38)	0.08 (0.01-0.19)

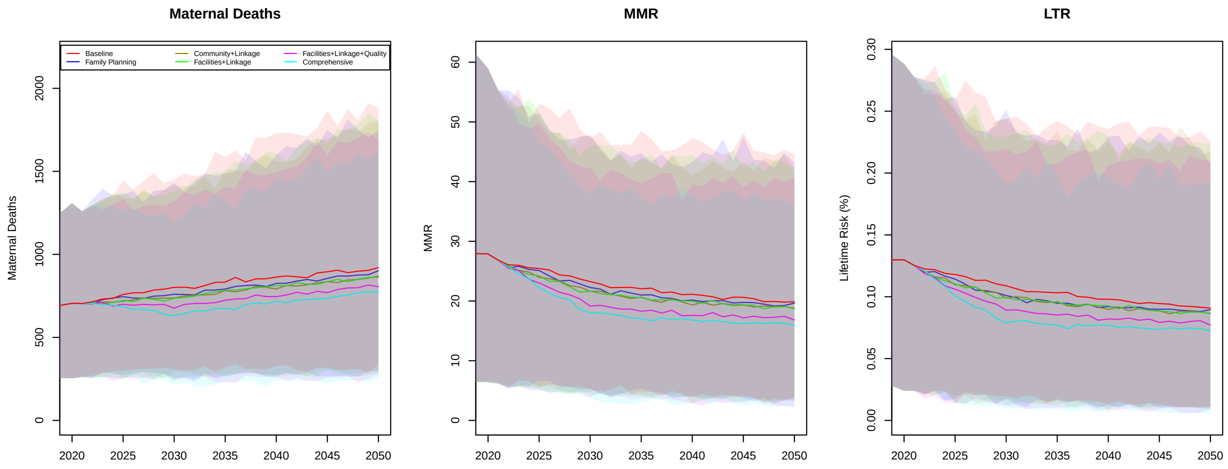

Ireland

ISO Code	Region	Area	Income Group
IRL	Northern Europe	Europe	High income

Projected Maternal Indicators in 2030 by Scenario

Scenario	Maternal Deaths	MMR	LTR
**Baseline**	25 (4-56)	18 (0-54)	0.04 (0-0.12)
**Family Planning Interventions**			
Contraception	24 (4-55)	18 (0-53)	0.04 (0-0.11)
Medical abortion	25 (5-56)	18 (0-55)	0.04 (0-0.12)
**Community-Based Interventions**			
ANC	24 (4-58)	18 (0-55)	0.04 (0-0.12)
SBA	25 (4-56)	18 (0-54)	0.04 (0-0.12)
**Facility-Based Interventions**			
Facility births	25 (4-56)	18 (0-54)	0.04 (0-0.12)
nonEmOC services	25 (4-56)	18 (0-54)	0.04 (0-0.12)
bEmOC services	25 (4-56)	18 (0-54)	0.04 (0-0.12)
cEmOC services	24 (4-56)	18 (0-52)	0.04 (0-0.12)
**System-Relevant Interventions**			
Quality of care	25 (5-56)	18 (0-54)	0.04 (0-0.12)
Referral	25 (4-56)	18 (0-54)	0.04 (0-0.12)
Transport	24 (0-57)	18 (0-55)	0.04 (0-0.12)
Targeted transfers	25 (5-56)	18 (0-54)	0.04 (0-0.12)
**Integrated Strategies**			
Family Planning	24 (4-54)	18 (0-53)	0.04 (0-0.11)
Community + Linkages	23 (5-54)	18 (0-53)	0.04 (0-0.12)
Facilities + Linkages	24 (0-54)	18 (0-51)	0.04 (0-0.12)
Facilities + Linkages + Quality	24 (0-54)	18 (0-51)	0.04 (0-0.12)
Comprehensive	21 (0-49)	16 (0-49)	0.04 (0-0.11)

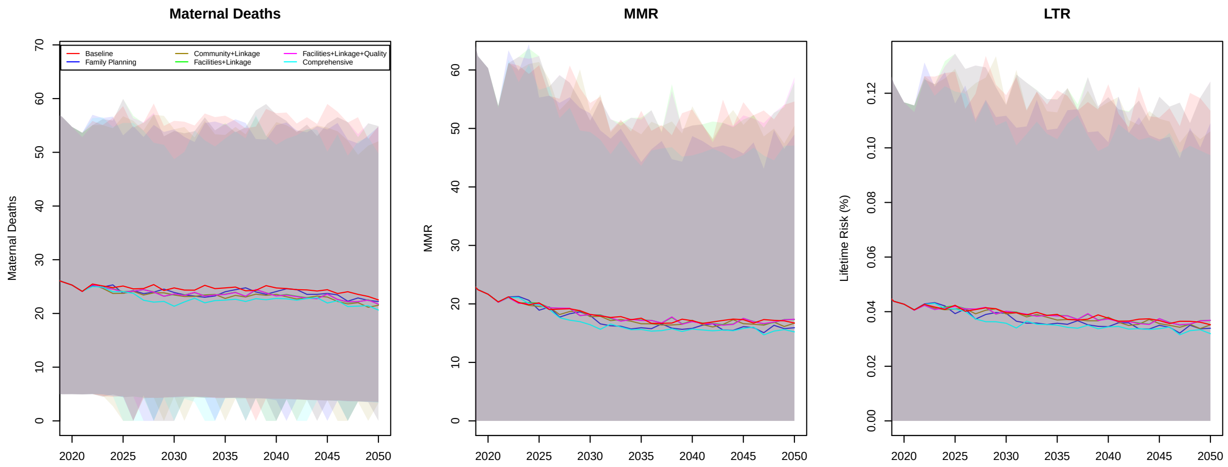

Israel

ISO Code	Region	Area	Income Group
ISR	Western Asia	Asia	High income

Projected Maternal Indicators in 2030 by Scenario

Scenario	Maternal Deaths	MMR	LTR
**Baseline**	45 (14-87)	11 (0-27)	0.04 (0-0.09)
**Family Planning Interventions**			
Contraception	32 (6-71)	11 (0-28)	0.03 (0-0.07)
Medical abortion	45 (15-87)	12 (0-28)	0.04 (0-0.09)
**Community-Based Interventions**			
ANC	45 (14-88)	12 (0-29)	0.04 (0-0.1)
SBA	45 (14-87)	12 (0-27)	0.04 (0-0.09)
**Facility-Based Interventions**			
Facility births	45 (12-85)	11 (0-26)	0.04 (0-0.09)
nonEmOC services	45 (14-87)	11 (0-27)	0.04 (0-0.09)
bEmOC services	45 (14-87)	12 (0-27)	0.04 (0-0.09)
cEmOC services	45 (12-88)	12 (2-28)	0.04 (0-0.1)
**System-Relevant Interventions**			
Quality of care	45 (14-87)	11 (0-27)	0.04 (0-0.09)
Referral	45 (14-88)	12 (0-27)	0.04 (0-0.09)
Transport	44 (12-86)	11 (0-27)	0.04 (0-0.09)
Targeted transfers	45 (14-87)	12 (0-27)	0.04 (0-0.09)
**Integrated Strategies**			
Family Planning	33 (6-69)	11 (0-28)	0.03 (0-0.07)
Community + Linkages	45 (12-91)	12 (0-28)	0.04 (0-0.1)
Facilities + Linkages	43 (12-85)	11 (0-28)	0.04 (0-0.09)
Facilities + Linkages + Quality	43 (12-84)	11 (0-28)	0.04 (0-0.09)
Comprehensive	32 (6-69)	11 (0-28)	0.03 (0-0.07)

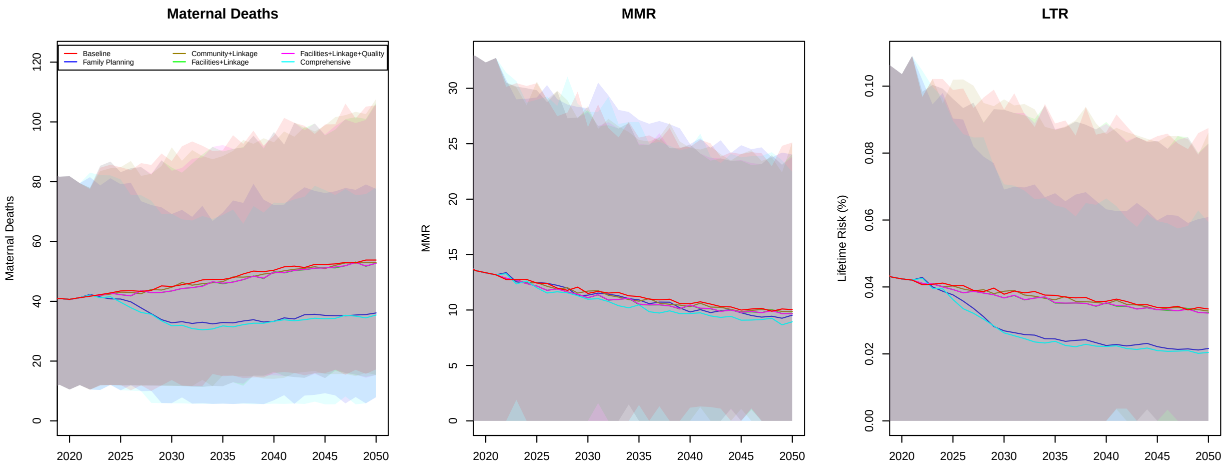

Italy

ISO Code	Region	Area	Income Group
ITA	Southern Europe	Europe	High income

Projected Maternal Indicators in 2030 by Scenario

Scenario	Maternal Deaths	MMR	LTR
**Baseline**	118 (34-228)	11 (0-32)	0.02 (0-0.05)
**Family Planning Interventions**			
Contraception	113 (30-228)	11 (0-31)	0.01 (0-0.05)
Medical abortion	116 (34-229)	11 (0-31)	0.01 (0-0.05)
**Community-Based Interventions**			
ANC	114 (30-223)	10 (0-30)	0.01 (0-0.04)
SBA	118 (30-231)	11 (0-32)	0.01 (0-0.05)
**Facility-Based Interventions**			
Facility births	118 (30-233)	11 (0-32)	0.01 (0-0.05)
nonEmOC services	118 (35-225)	11 (0-32)	0.02 (0-0.04)
bEmOC services	118 (35-225)	11 (0-32)	0.02 (0-0.04)
cEmOC services	117 (30-227)	11 (0-32)	0.01 (0-0.04)
**System-Relevant Interventions**			
Quality of care	118 (34-225)	11 (0-32)	0.02 (0-0.04)
Referral	119 (34-231)	11 (0-33)	0.02 (0-0.05)
Transport	105 (29-219)	11 (0-31)	0.01 (0-0.04)
Targeted transfers	118 (35-231)	11 (0-32)	0.01 (0-0.05)
**Integrated Strategies**			
Family Planning	113 (30-224)	10 (0-30)	0.01 (0-0.04)
Community + Linkages	104 (30-208)	10 (0-30)	0.01 (0-0.04)
Facilities + Linkages	106 (30-224)	10 (0-29)	0.01 (0-0.04)
Facilities + Linkages + Quality	106 (30-224)	10 (0-29)	0.01 (0-0.04)
Comprehensive	99 (24-212)	9 (0-27)	0.01 (0-0.04)

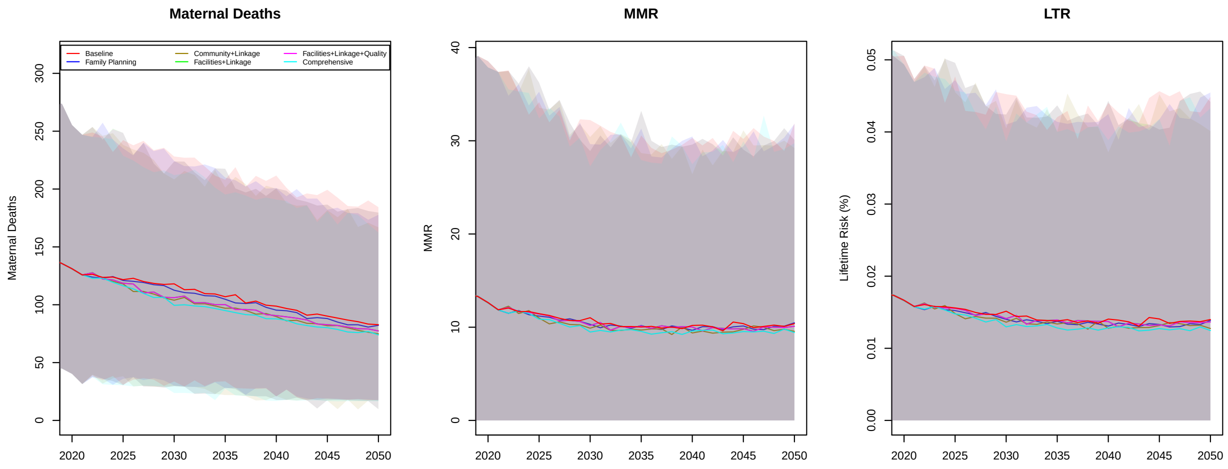

Jamaica

ISO Code	Region	Area	Income Group
JAM	Caribbean	Latin America and the Caribbean	Upper middle income

Projected Maternal Indicators in 2030 by Scenario

Scenario	Maternal Deaths	MMR	LTR
**Baseline**	39 (6-95)	81 (7-215)	0.15 (0.01-0.39)
**Family Planning Interventions**			
Contraception	36 (6-87)	72 (7-201)	0.13 (0.01-0.37)
Medical abortion	39 (7-98)	81 (7-214)	0.14 (0.01-0.39)
**Community-Based Interventions**			
ANC	39 (6-90)	79 (6-206)	0.14 (0.01-0.38)
SBA	38 (6-84)	77 (6-196)	0.14 (0.01-0.34)
**Facility-Based Interventions**			
Facility births	35 (4-78)	69 (0-182)	0.12 (0-0.31)
nonEmOC services	39 (6-93)	81 (7-211)	0.14 (0.01-0.38)
bEmOC services	40 (6-94)	82 (7-215)	0.15 (0.01-0.38)
cEmOC services	40 (5-98)	82 (6-228)	0.15 (0.01-0.4)
**System-Relevant Interventions**			
Quality of care	39 (6-95)	81 (7-215)	0.14 (0.01-0.39)
Referral	39 (5-93)	81 (7-213)	0.14 (0.01-0.38)
Transport	37 (5-92)	78 (5-211)	0.14 (0.01-0.38)
Targeted transfers	39 (7-95)	81 (7-221)	0.15 (0.01-0.4)
**Integrated Strategies**			
Family Planning	36 (6-88)	71 (5-194)	0.13 (0.01-0.37)
Community + Linkages	35 (4-82)	73 (4-185)	0.13 (0.01-0.33)
Facilities + Linkages	34 (3-80)	70 (0-192)	0.12 (0-0.33)
Facilities + Linkages + Quality	33 (3-76)	68 (0-188)	0.12 (0-0.31)
Comprehensive	30 (3-68)	58 (0-144)	0.11 (0-0.27)

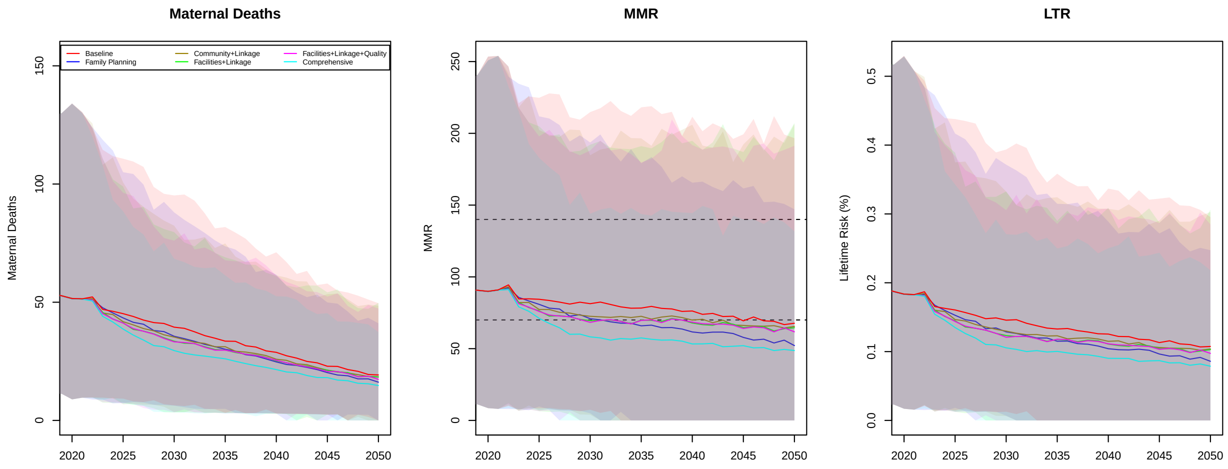

Japan

ISO Code	Region	Area	Income Group
JPN	Eastern Asia	Asia	High income

Projected Maternal Indicators in 2030 by Scenario

Scenario	Maternal Deaths	MMR	LTR
**Baseline**	91 (0-218)	6 (0-15)	0.01 (0-0.03)
**Family Planning Interventions**			
Contraception	88 (0-226)	5 (0-17)	0.01 (0-0.03)
Medical abortion	91 (0-216)	6 (0-15)	0.01 (0-0.03)
**Community-Based Interventions**			
ANC	92 (0-223)	6 (0-17)	0.01 (0-0.03)
SBA	91 (0-218)	6 (0-15)	0.01 (0-0.03)
**Facility-Based Interventions**			
Facility births	92 (0-218)	6 (0-15)	0.01 (0-0.03)
nonEmOC services	91 (0-218)	6 (0-15)	0.01 (0-0.03)
bEmOC services	91 (0-218)	6 (0-15)	0.01 (0-0.03)
cEmOC services	89 (0-216)	5 (0-15)	0.01 (0-0.02)
**System-Relevant Interventions**			
Quality of care	91 (0-218)	6 (0-15)	0.01 (0-0.03)
Referral	91 (0-218)	6 (0-15)	0.01 (0-0.03)
Transport	88 (0-217)	6 (0-15)	0.01 (0-0.02)
Targeted transfers	91 (0-218)	6 (0-15)	0.01 (0-0.03)
**Integrated Strategies**			
Family Planning	88 (0-227)	5 (0-17)	0.01 (0-0.03)
Community + Linkages	87 (0-216)	5 (0-16)	0.01 (0-0.02)
Facilities + Linkages	85 (0-215)	5 (0-16)	0.01 (0-0.03)
Facilities + Linkages + Quality	85 (0-209)	5 (0-16)	0.01 (0-0.03)
Comprehensive	88 (0-220)	6 (0-17)	0.01 (0-0.03)

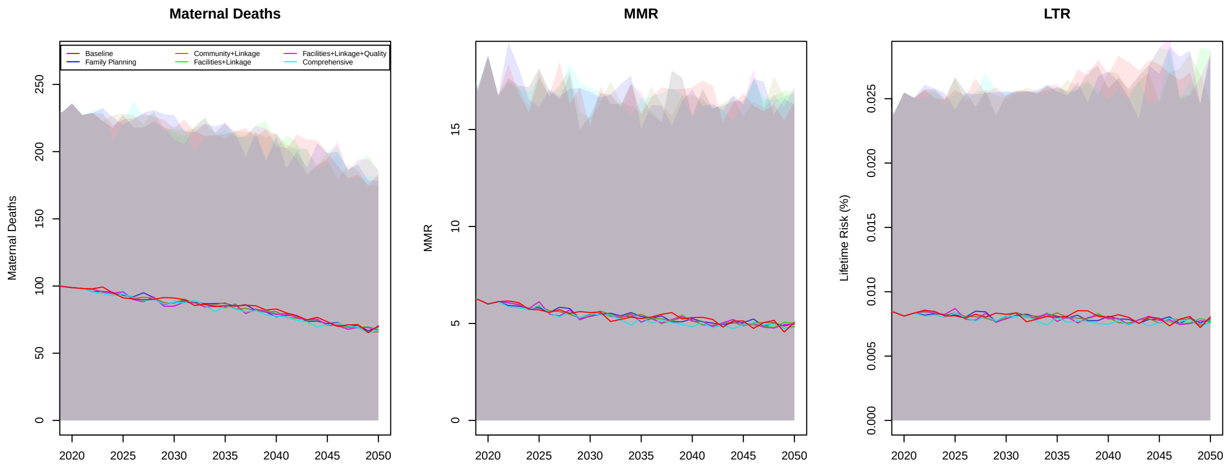

Jordan

ISO Code	Region	Area	Income Group
JOR	Western Asia	Asia	Upper middle income

Projected Maternal Indicators in 2030 by Scenario

Scenario	Maternal Deaths	MMR	LTR
**Baseline**	97 (0-234)	13 (0-43)	0.05 (0-0.18)
**Family Planning Interventions**			
Contraception	94 (0-225)	13 (0-42)	0.05 (0-0.18)
Medical abortion	96 (0-235)	14 (0-44)	0.05 (0-0.18)
**Community-Based Interventions**			
ANC	97 (0-238)	13 (0-42)	0.05 (0-0.18)
SBA	96 (0-238)	13 (0-43)	0.05 (0-0.19)
**Facility-Based Interventions**			
Facility births	98 (0-253)	13 (0-43)	0.05 (0-0.19)
nonEmOC services	97 (0-233)	13 (0-43)	0.05 (0-0.18)
bEmOC services	96 (0-234)	13 (0-44)	0.05 (0-0.18)
cEmOC services	95 (0-231)	13 (0-43)	0.05 (0-0.18)
**System-Relevant Interventions**			
Quality of care	96 (0-244)	13 (0-41)	0.05 (0-0.17)
Referral	98 (0-232)	13 (0-41)	0.05 (0-0.18)
Transport	93 (0-260)	13 (0-46)	0.05 (0-0.18)
Targeted transfers	99 (0-240)	14 (0-44)	0.05 (0-0.18)
**Integrated Strategies**			
Family Planning	92 (0-226)	13 (0-42)	0.05 (0-0.17)
Community + Linkages	93 (0-239)	12 (0-42)	0.05 (0-0.17)
Facilities + Linkages	94 (0-223)	13 (0-40)	0.05 (0-0.18)
Facilities + Linkages + Quality	90 (0-220)	12 (0-39)	0.05 (0-0.17)
Comprehensive	87 (0-220)	11 (0-38)	0.04 (0-0.17)

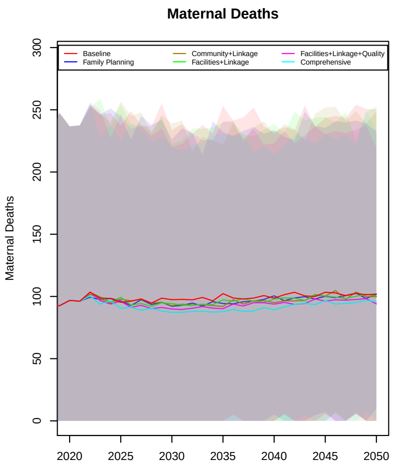

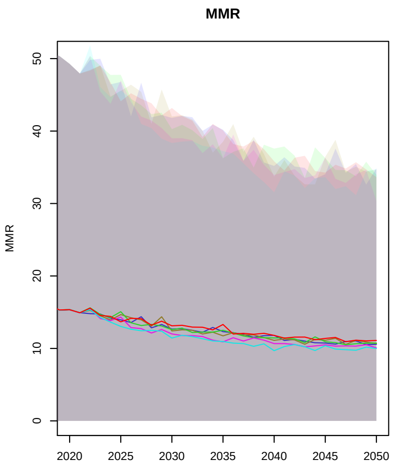

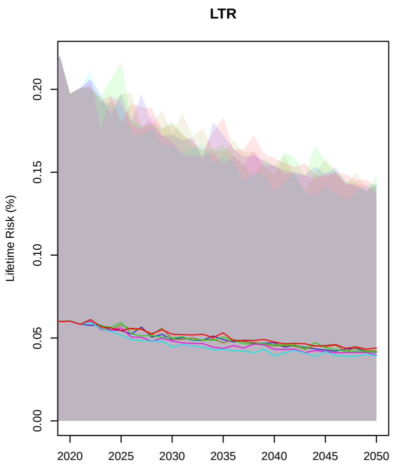

Kazakhstan

ISO Code	Region	Area	Income Group
KAZ	Central Asia	Asia	Upper middle income

Projected Maternal Indicators in 2030 by Scenario

Scenario	Maternal Deaths	MMR	LTR
**Baseline**	284 (34-944)	66 (6-232)	0.17 (0-0.63)
**Family Planning Interventions**			
Contraception	205 (0-626)	48 (0-185)	0.12 (0-0.41)
Medical abortion	194 (28-517)	42 (0-131)	0.11 (0-0.33)
**Community-Based Interventions**			
ANC	273 (34-852)	63 (0-224)	0.17 (0-0.61)
SBA	283 (34-944)	66 (0-232)	0.17 (0-0.63)
**Facility-Based Interventions**			
Facility births	280 (34-812)	65 (0-212)	0.17 (0-0.59)
nonEmOC services	283 (34-944)	66 (6-232)	0.17 (0-0.64)
bEmOC services	283 (34-944)	66 (6-232)	0.17 (0.01-0.63)
cEmOC services	278 (34-773)	65 (0-216)	0.17 (0-0.57)
**System-Relevant Interventions**			
Quality of care	283 (34-1008)	66 (3-269)	0.17 (0-0.66)
Referral	280 (34-815)	65 (5-210)	0.17 (0-0.58)
Transport	279 (34-1015)	66 (0-237)	0.17 (0-0.69)
Targeted transfers	283 (35-930)	66 (6-230)	0.17 (0-0.61)
**Integrated Strategies**			
Family Planning	171 (0-459)	38 (0-123)	0.1 (0-0.29)
Community + Linkages	268 (31-841)	62 (0-224)	0.16 (0-0.57)
Facilities + Linkages	273 (33-822)	64 (0-213)	0.17 (0-0.58)
Facilities + Linkages + Quality	271 (32-822)	64 (0-213)	0.17 (0-0.58)
Comprehensive	157 (0-444)	35 (0-117)	0.09 (0-0.29)

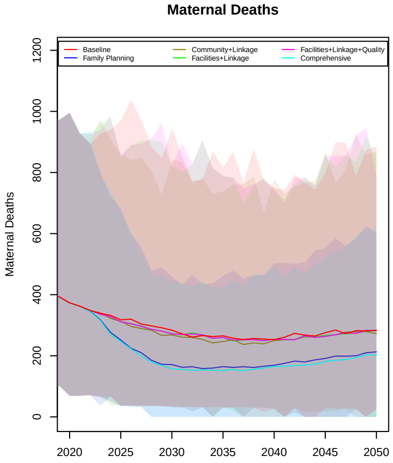

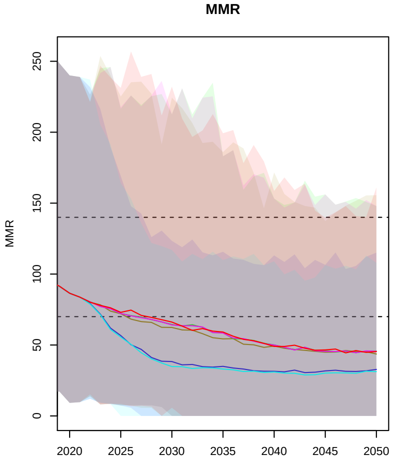

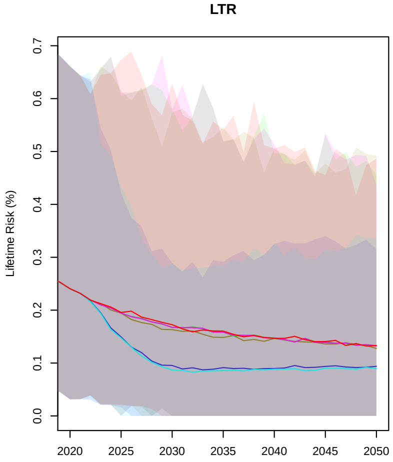

Kenya

ISO Code	Region	Area	Income Group
KEN	Eastern Africa	Africa	Lower middle income

Projected Maternal Indicators in 2030 by Scenario

Scenario	Maternal Deaths	MMR	LTR
**Baseline**	5285 (1800-10771)	222 (84-405)	0.92 (0.26-1.99)
**Family Planning Interventions**			
Contraception	5073 (1923-10160)	219 (89-407)	0.88 (0.27-1.88)
Medical abortion	5288 (1836-10513)	222 (83-406)	0.92 (0.26-1.97)
**Community-Based Interventions**			
ANC	5236 (1812-10346)	220 (85-413)	0.91 (0.27-1.96)
SBA	4577 (1719-9431)	189 (73-368)	0.79 (0.25-1.83)
**Facility-Based Interventions**			
Facility births	3059 (642-7923)	119 (16-312)	0.53 (0.06-1.56)
nonEmOC services	5292 (1800-10531)	222 (84-411)	0.92 (0.27-2)
bEmOC services	5289 (1771-10619)	222 (85-402)	0.92 (0.27-1.95)
cEmOC services	5279 (1855-10542)	221 (85-415)	0.92 (0.28-2)
**System-Relevant Interventions**			
Quality of care	5289 (1860-10556)	222 (84-409)	0.92 (0.28-1.95)
Referral	5235 (1815-10401)	219 (87-405)	0.91 (0.28-1.93)
Transport	4700 (1602-10118)	200 (75-385)	0.83 (0.25-1.87)
Targeted transfers	5302 (1802-10506)	223 (80-417)	0.92 (0.27-1.97)
**Integrated Strategies**			
Family Planning	5016 (1824-10128)	217 (85-395)	0.86 (0.27-1.83)
Community + Linkages	4068 (1363-9093)	172 (57-355)	0.72 (0.19-1.76)
Facilities + Linkages	2873 (574-7890)	115 (15-303)	0.51 (0.05-1.54)
Facilities + Linkages + Quality	2844 (563-7618)	114 (15-290)	0.51 (0.05-1.51)
Comprehensive	2579 (435-6720)	105 (12-278)	0.46 (0.03-1.34)

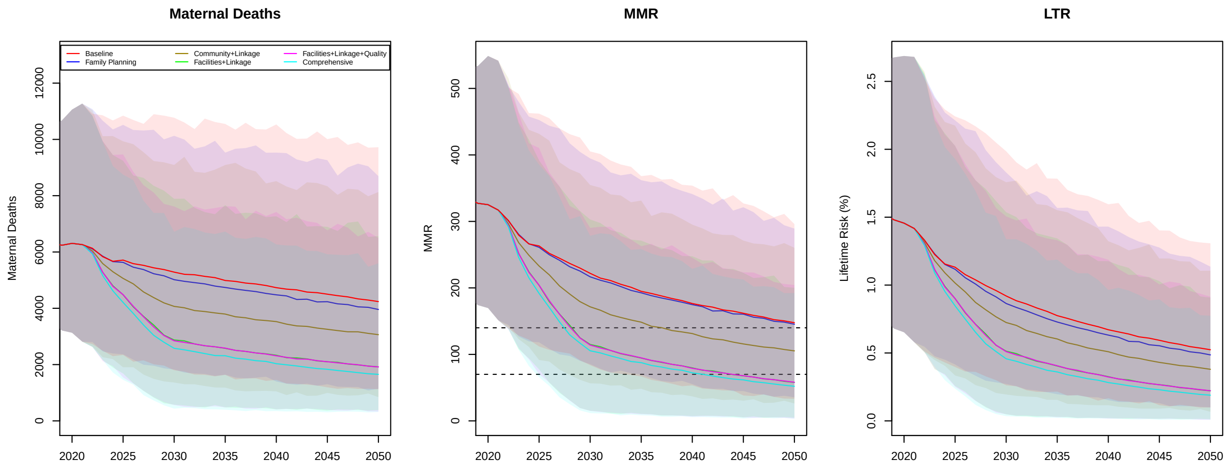

Kiribati

ISO Code	Region	Area	Income Group
KIR	Micronesia	Oceania	Lower middle income

Projected Maternal Indicators in 2030 by Scenario

Scenario	Maternal Deaths	MMR	LTR
**Baseline**	6 (0-19)	146 (0-410)	0.49 (0-1.57)
**Family Planning Interventions**			
Contraception	6 (0-17)	143 (0-395)	0.46 (0-1.53)
Medical abortion	6 (0-19)	147 (0-418)	0.49 (0-1.58)
**Community-Based Interventions**			
ANC	6 (0-19)	146 (0-450)	0.49 (0-1.5)
SBA	6 (0-19)	146 (0-406)	0.49 (0-1.56)
**Facility-Based Interventions**			
Facility births	6 (0-19)	146 (0-406)	0.49 (0-1.59)
nonEmOC services	6 (0-19)	145 (0-402)	0.49 (0-1.57)
bEmOC services	6 (0-20)	144 (0-416)	0.48 (0-1.58)
cEmOC services	6 (0-16)	128 (0-395)	0.42 (0-1.29)
**System-Relevant Interventions**			
Quality of care	6 (0-20)	147 (0-415)	0.49 (0-1.59)
Referral	6 (0-19)	145 (0-416)	0.49 (0-1.61)
Transport	6 (0-18)	150 (0-434)	0.5 (0-1.61)
Targeted transfers	6 (0-19)	146 (0-410)	0.49 (0-1.57)
**Integrated Strategies**			
Family Planning	6 (0-17)	141 (0-400)	0.45 (0-1.51)
Community + Linkages	6 (0-17)	143 (0-416)	0.48 (0-1.48)
Facilities + Linkages	6 (0-16)	131 (0-377)	0.44 (0-1.38)
Facilities + Linkages + Quality	5 (0-16)	128 (0-410)	0.43 (0-1.42)
Comprehensive	5 (0-15)	116 (0-357)	0.37 (0-1.26)

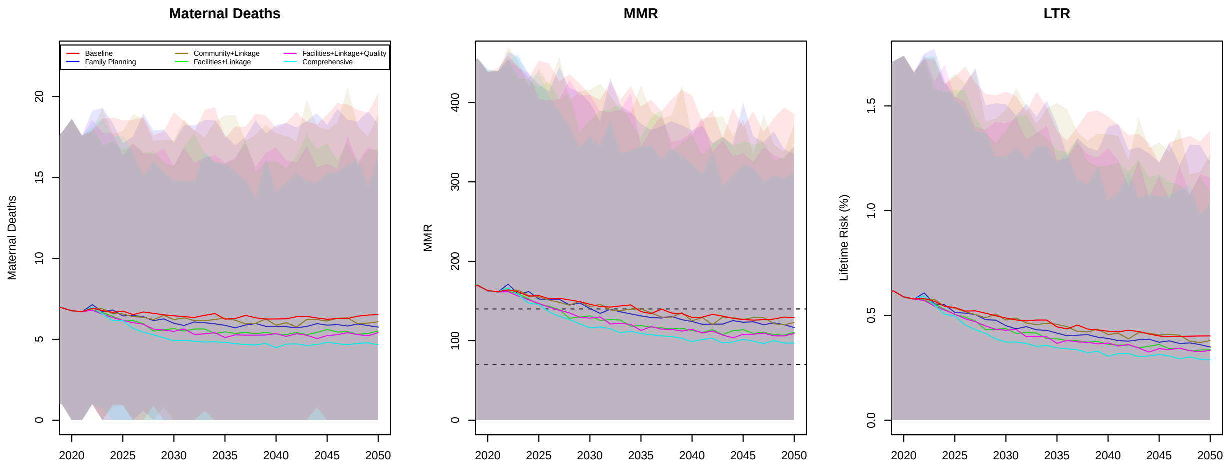

Kuwait

ISO Code	Region	Area	Income Group
KWT	Western Asia	Asia	High income

Projected Maternal Indicators in 2030 by Scenario

Scenario	Maternal Deaths	MMR	LTR
**Baseline**	18 (0-57)	27 (0-95)	0.05 (0-0.19)
**Family Planning Interventions**			
Contraception	16 (0-48)	24 (0-84)	0.04 (0-0.16)
Medical abortion	16 (0-51)	23 (0-88)	0.04 (0-0.17)
**Community-Based Interventions**			
ANC	19 (0-62)	29 (0-101)	0.05 (0-0.21)
SBA	18 (0-57)	27 (0-96)	0.05 (0-0.2)
**Facility-Based Interventions**			
Facility births	18 (0-57)	27 (0-92)	0.05 (0-0.2)
nonEmOC services	18 (0-57)	27 (0-95)	0.05 (0-0.19)
bEmOC services	18 (0-57)	27 (0-94)	0.05 (0-0.19)
cEmOC services	19 (0-56)	28 (0-93)	0.05 (0-0.19)
**System-Relevant Interventions**			
Quality of care	18 (0-57)	27 (0-95)	0.05 (0-0.19)
Referral	19 (0-58)	28 (0-97)	0.05 (0-0.19)
Transport	18 (0-58)	27 (0-98)	0.05 (0-0.2)
Targeted transfers	18 (0-58)	27 (0-99)	0.05 (0-0.2)
**Integrated Strategies**			
Family Planning	16 (0-54)	23 (0-80)	0.04 (0-0.15)
Community + Linkages	18 (0-56)	27 (0-91)	0.05 (0-0.19)
Facilities + Linkages	18 (0-57)	26 (0-98)	0.05 (0-0.2)
Facilities + Linkages + Quality	18 (0-58)	26 (0-98)	0.05 (0-0.2)
Comprehensive	15 (0-46)	22 (0-83)	0.04 (0-0.15)

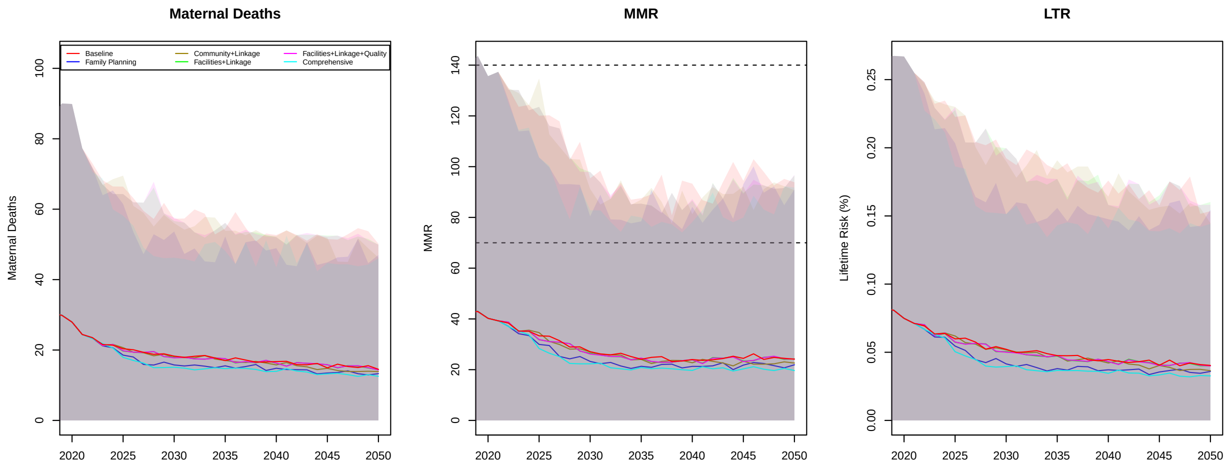

Kyrgyzstan

ISO Code	Region	Area	Income Group
KGZ	Central Asia	Asia	Lower middle income

Projected Maternal Indicators in 2030 by Scenario

Scenario	Maternal Deaths	MMR	LTR
**Baseline**	134 (0-378)	48 (0-166)	0.15 (0-0.56)
**Family Planning Interventions**			
Contraception	108 (0-313)	42 (0-162)	0.12 (0-0.49)
Medical abortion	121 (0-330)	42 (0-154)	0.14 (0-0.5)
**Community-Based Interventions**			
ANC	132 (0-381)	48 (0-176)	0.15 (0-0.58)
SBA	134 (0-374)	48 (0-166)	0.15 (0-0.56)
**Facility-Based Interventions**			
Facility births	134 (0-378)	48 (0-166)	0.15 (0-0.56)
nonEmOC services	134 (0-374)	48 (0-166)	0.15 (0-0.56)
bEmOC services	132 (0-366)	48 (0-164)	0.15 (0-0.56)
cEmOC services	123 (0-358)	42 (0-164)	0.13 (0-0.53)
**System-Relevant Interventions**			
Quality of care	134 (0-362)	48 (0-165)	0.15 (0-0.56)
Referral	134 (0-374)	48 (0-166)	0.15 (0-0.56)
Transport	121 (0-354)	48 (0-166)	0.15 (0-0.52)
Targeted transfers	136 (0-381)	49 (0-171)	0.16 (0-0.57)
**Integrated Strategies**			
Family Planning	106 (0-322)	38 (0-153)	0.11 (0-0.44)
Community + Linkages	117 (0-336)	46 (0-153)	0.15 (0-0.52)
Facilities + Linkages	112 (0-346)	41 (0-165)	0.13 (0-0.53)
Facilities + Linkages + Quality	112 (0-346)	41 (0-165)	0.13 (0-0.53)
Comprehensive	81 (0-259)	30 (0-142)	0.09 (0-0.38)

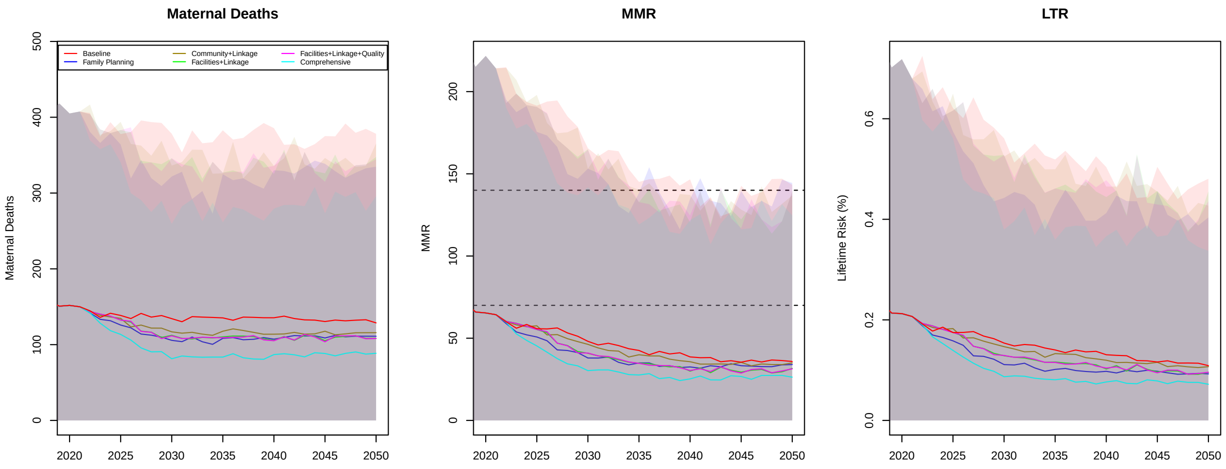

Lao People's Democratic Republic

ISO Code	Region	Area	Income Group
LAO	South-Eastern Asia	Asia	Lower middle income

Projected Maternal Indicators in 2030 by Scenario

Scenario	Maternal Deaths	MMR	LTR
**Baseline**	240 (23-615)	102 (2-305)	0.26 (0-0.74)
**Family Planning Interventions**			
Contraception	234 (23-658)	100 (0-319)	0.25 (0-0.77)
Medical abortion	235 (23-650)	99 (4-293)	0.25 (0-0.76)
**Community-Based Interventions**			
ANC	226 (20-581)	97 (2-305)	0.25 (0-0.7)
SBA	211 (24-581)	86 (4-244)	0.22 (0-0.67)
**Facility-Based Interventions**			
Facility births	135 (0-369)	45 (0-149)	0.12 (0-0.43)
nonEmOC services	241 (22-630)	102 (3-307)	0.26 (0-0.71)
bEmOC services	242 (23-615)	103 (0-316)	0.27 (0-0.76)
cEmOC services	235 (27-610)	100 (5-312)	0.26 (0.01-0.74)
**System-Relevant Interventions**			
Quality of care	222 (19-659)	92 (0-309)	0.24 (0-0.74)
Referral	239 (22-589)	103 (1-309)	0.26 (0-0.7)
Transport	205 (22-525)	87 (0-239)	0.22 (0-0.63)
Targeted transfers	242 (25-589)	103 (0-302)	0.27 (0-0.71)
**Integrated Strategies**			
Family Planning	229 (21-659)	97 (0-306)	0.25 (0-0.75)
Community + Linkages	182 (18-481)	72 (0-205)	0.19 (0-0.55)
Facilities + Linkages	115 (0-327)	37 (0-126)	0.1 (0-0.37)
Facilities + Linkages + Quality	95 (0-282)	27 (0-107)	0.07 (0-0.3)
Comprehensive	89 (0-282)	24 (0-96)	0.06 (0-0.26)

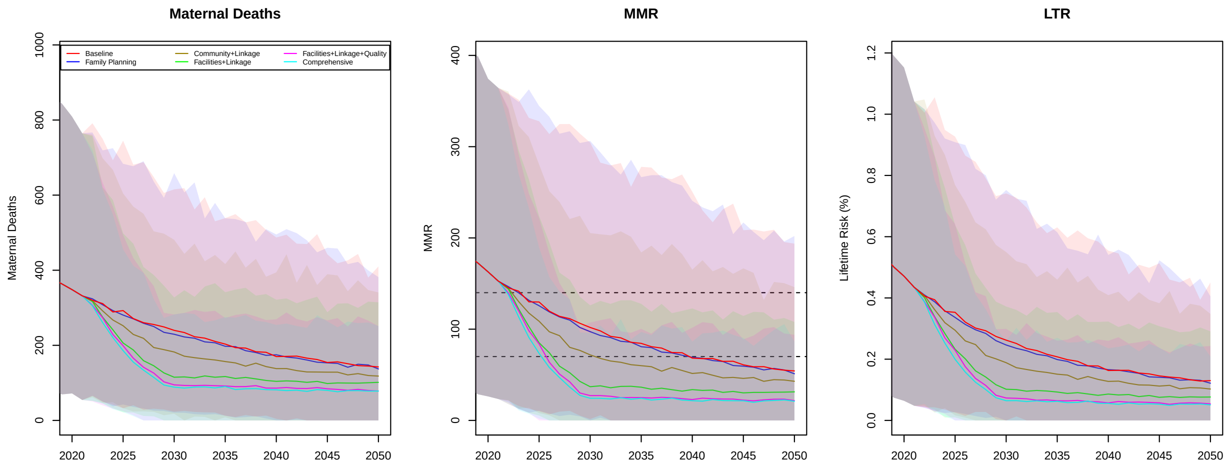

Latvia

ISO Code	Region	Area	Income Group
LVA	Northern Europe	Europe	High income

Projected Maternal Indicators in 2030 by Scenario

Scenario	Maternal Deaths	MMR	LTR
**Baseline**	4 (0-14)	14 (0-63)	0.02 (0-0.1)
**Family Planning Interventions**			
Contraception	4 (0-14)	14 (0-65)	0.02 (0-0.1)
Medical abortion	4 (0-14)	14 (0-62)	0.02 (0-0.09)
**Community-Based Interventions**			
ANC	4 (0-14)	14 (0-64)	0.02 (0-0.1)
SBA	4 (0-14)	14 (0-63)	0.02 (0-0.1)
**Facility-Based Interventions**			
Facility births	4 (0-14)	14 (0-63)	0.02 (0-0.1)
nonEmOC services	4 (0-14)	14 (0-63)	0.02 (0-0.1)
bEmOC services	4 (0-14)	14 (0-63)	0.02 (0-0.1)
cEmOC services	4 (0-14)	13 (0-63)	0.02 (0-0.1)
**System-Relevant Interventions**			
Quality of care	4 (0-11)	12 (0-64)	0.01 (0-0.09)
Referral	4 (0-14)	14 (0-63)	0.02 (0-0.1)
Transport	4 (0-11)	14 (0-64)	0.02 (0-0.1)
Targeted transfers	4 (0-14)	14 (0-63)	0.02 (0-0.1)
**Integrated Strategies**			
Family Planning	4 (0-14)	14 (0-65)	0.02 (0-0.1)
Community + Linkages	4 (0-11)	14 (0-63)	0.02 (0-0.1)
Facilities + Linkages	4 (0-13)	14 (0-64)	0.02 (0-0.1)
Facilities + Linkages + Quality	3 (0-11)	12 (0-65)	0.02 (0-0.09)
Comprehensive	3 (0-11)	12 (0-62)	0.01 (0-0.08)

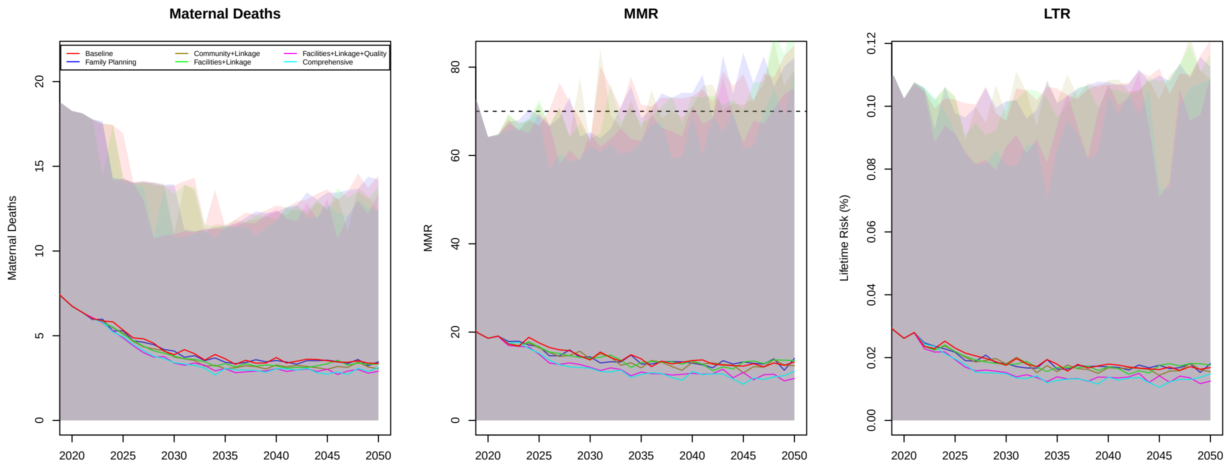

Lebanon

ISO Code	Region	Area	Income Group
LBN	Western Asia	Asia	Upper middle income

Projected Maternal Indicators in 2030 by Scenario

Scenario	Maternal Deaths	MMR	LTR
**Baseline**	18 (0-108)	19 (0-152)	0.03 (0-0.19)
**Family Planning Interventions**			
Contraception	22 (0-110)	19 (0-134)	0.04 (0-0.27)
Medical abortion	16 (0-107)	18 (0-147)	0.03 (0-0.19)
**Community-Based Interventions**			
ANC	17 (0-108)	18 (0-141)	0.03 (0-0.21)
SBA	17 (0-107)	17 (0-141)	0.03 (0-0.19)
**Facility-Based Interventions**			
Facility births	17 (0-108)	19 (0-152)	0.03 (0-0.19)
nonEmOC services	18 (0-108)	19 (0-152)	0.03 (0-0.2)
bEmOC services	18 (0-108)	19 (0-152)	0.03 (0-0.19)
cEmOC services	17 (0-107)	19 (0-152)	0.03 (0-0.19)
**System-Relevant Interventions**			
Quality of care	18 (0-108)	18 (0-149)	0.03 (0-0.19)
Referral	18 (0-108)	19 (0-152)	0.03 (0-0.26)
Transport	18 (0-108)	20 (0-154)	0.03 (0-0.26)
Targeted transfers	18 (0-108)	20 (0-153)	0.03 (0-0.19)
**Integrated Strategies**			
Family Planning	20 (0-109)	17 (0-128)	0.03 (0-0.19)
Community + Linkages	16 (0-109)	18 (0-150)	0.03 (0-0.26)
Facilities + Linkages	16 (0-109)	16 (0-139)	0.03 (0-0.19)
Facilities + Linkages + Quality	16 (0-109)	16 (0-139)	0.03 (0-0.19)
Comprehensive	19 (0-109)	17 (0-130)	0.03 (0-0.2)

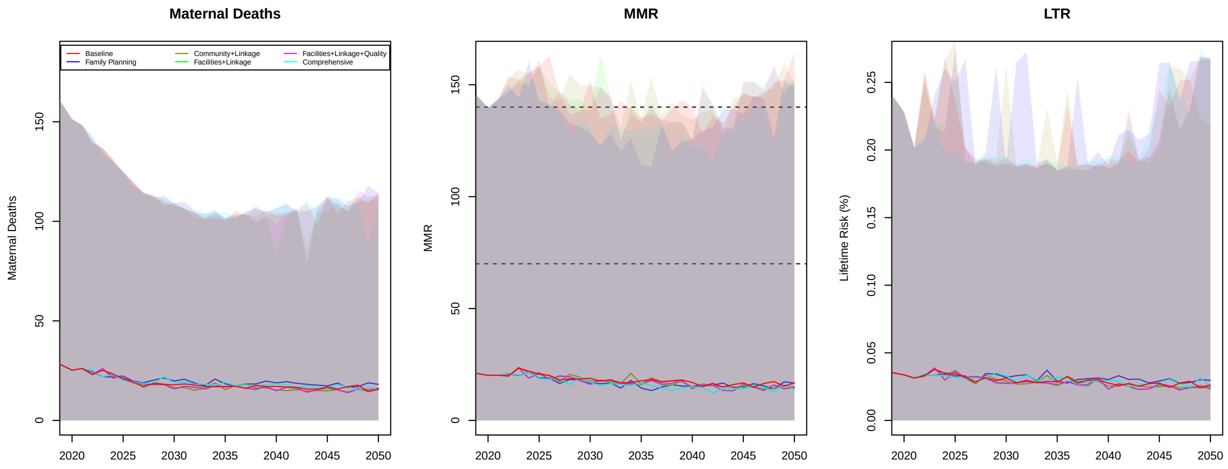

Lesotho

ISO Code	Region	Area	Income Group
LSO	Southern Africa	Africa	Lower middle income

Projected Maternal Indicators in 2030 by Scenario

Scenario	Maternal Deaths	MMR	LTR
**Baseline**	262 (114-467)	426 (187-761)	1.26 (0.53-2.3)
**Family Planning Interventions**			
Contraception	242 (99-440)	393 (154-723)	1.16 (0.41-2.19)
Medical abortion	254 (104-458)	411 (159-764)	1.22 (0.44-2.31)
**Community-Based Interventions**			
ANC	263 (110-481)	427 (175-778)	1.26 (0.5-2.39)
SBA	260 (115-482)	421 (177-770)	1.24 (0.52-2.38)
**Facility-Based Interventions**			
Facility births	247 (103-456)	399 (159-727)	1.19 (0.45-2.22)
nonEmOC services	263 (113-478)	426 (183-768)	1.26 (0.52-2.41)
bEmOC services	261 (114-468)	424 (186-772)	1.26 (0.53-2.35)
cEmOC services	258 (100-474)	418 (168-777)	1.24 (0.47-2.37)
**System-Relevant Interventions**			
Quality of care	230 (90-447)	371 (144-707)	1.1 (0.41-2.24)
Referral	263 (110-486)	428 (180-792)	1.27 (0.51-2.42)
Transport	258 (106-470)	422 (166-784)	1.25 (0.48-2.38)
Targeted transfers	262 (111-468)	427 (186-755)	1.26 (0.52-2.33)
**Integrated Strategies**			
Family Planning	242 (97-452)	392 (145-736)	1.15 (0.43-2.25)
Community + Linkages	253 (103-474)	415 (167-758)	1.23 (0.49-2.35)
Facilities + Linkages	242 (99-441)	395 (145-720)	1.17 (0.43-2.18)
Facilities + Linkages + Quality	212 (71-430)	343 (115-699)	1.03 (0.33-2.13)
Comprehensive	188 (52-382)	304 (84-611)	0.91 (0.24-1.93)

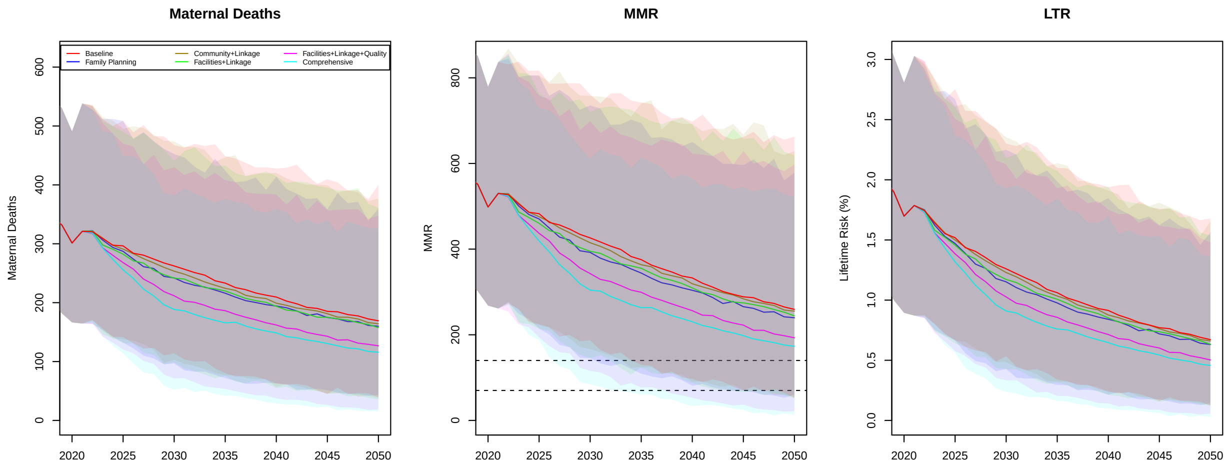

Liberia

ISO Code	Region	Area	Income Group
LBR	Western Africa	Africa	Low income

Projected Maternal Indicators in 2030 by Scenario

Scenario	Maternal Deaths	MMR	LTR
**Baseline**	1470 (700-2331)	654 (310-1041)	2.62 (1.18-4.19)
**Family Planning Interventions**			
Contraception	1359 (671-2136)	638 (320-996)	2.44 (1.13-3.92)
Medical abortion	1435 (741-2314)	638 (319-1021)	2.56 (1.2-4.27)
**Community-Based Interventions**			
ANC	1443 (704-2382)	641 (305-1020)	2.58 (1.12-4.24)
SBA	1436 (754-2281)	642 (320-1029)	2.56 (1.19-4.31)
**Facility-Based Interventions**			
Facility births	1090 (513-1769)	476 (221-790)	1.91 (0.81-3.23)
nonEmOC services	1457 (710-2345)	650 (310-1011)	2.61 (1.13-4.23)
bEmOC services	1478 (729-2382)	659 (329-1060)	2.64 (1.24-4.3)
cEmOC services	1447 (701-2331)	644 (312-1041)	2.59 (1.13-4.35)
**System-Relevant Interventions**			
Quality of care	993 (282-1828)	427 (100-800)	1.76 (0.42-3.27)
Referral	1460 (747-2363)	650 (309-1042)	2.62 (1.23-4.36)
Transport	1376 (694-2270)	612 (307-973)	2.47 (1.14-4.09)
Targeted transfers	1479 (728-2346)	660 (319-1074)	2.66 (1.22-4.31)
**Integrated Strategies**			
Family Planning	1330 (631-2138)	623 (308-988)	2.37 (1.1-3.93)
Community + Linkages	1252 (608-2029)	554 (255-889)	2.23 (0.98-3.68)
Facilities + Linkages	1019 (493-1712)	444 (212-743)	1.78 (0.81-3)
Facilities + Linkages + Quality	334 (59-751)	122 (17-303)	0.51 (0-1.32)
Comprehensive	274 (37-621)	99 (0-252)	0.41 (0-1.18)

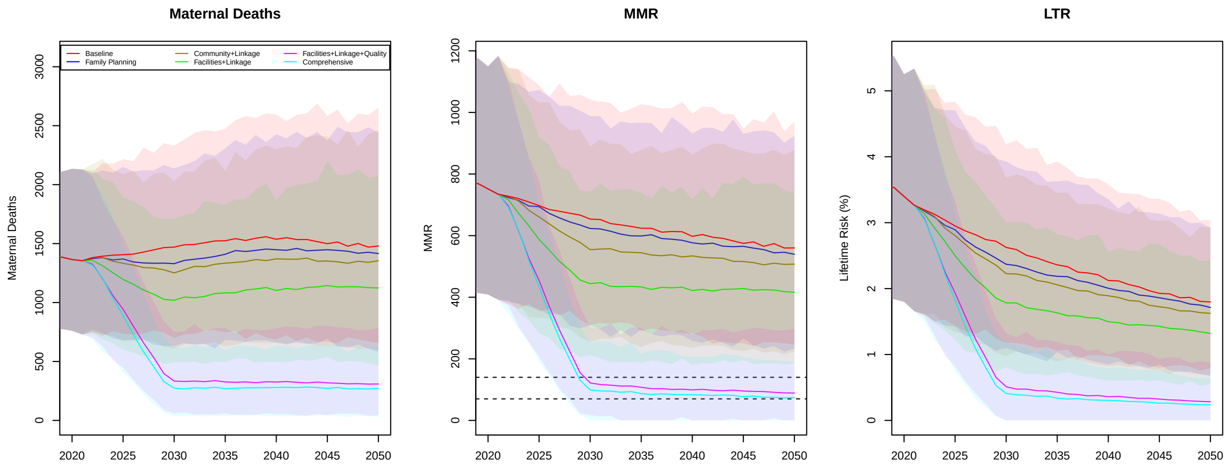

Libya

ISO Code	Region	Area	Income Group
LBY	Northern Africa	Africa	Upper middle income

Projected Maternal Indicators in 2030 by Scenario

Scenario	Maternal Deaths	MMR	LTR
**Baseline**	102 (0-351)	44 (0-164)	0.15 (0-0.59)
**Family Planning Interventions**			
Contraception	89 (0-310)	39 (0-156)	0.12 (0-0.54)
Medical abortion	103 (0-359)	44 (0-177)	0.15 (0-0.61)
**Community-Based Interventions**			
ANC	102 (0-353)	44 (0-166)	0.15 (0-0.6)
SBA	101 (0-345)	43 (0-161)	0.15 (0-0.59)
**Facility-Based Interventions**			
Facility births	101 (0-351)	43 (0-163)	0.15 (0-0.59)
nonEmOC services	100 (0-359)	43 (0-166)	0.15 (0-0.62)
bEmOC services	102 (0-351)	44 (0-164)	0.15 (0-0.59)
cEmOC services	98 (0-339)	41 (0-164)	0.14 (0-0.59)
**System-Relevant Interventions**			
Quality of care	98 (0-330)	41 (0-144)	0.14 (0-0.56)
Referral	100 (0-357)	43 (0-163)	0.15 (0-0.6)
Transport	98 (0-357)	43 (0-166)	0.14 (0-0.59)
Targeted transfers	102 (0-357)	44 (0-172)	0.15 (0-0.6)
**Integrated Strategies**			
Family Planning	89 (0-307)	38 (0-155)	0.12 (0-0.54)
Community + Linkages	98 (0-344)	42 (0-171)	0.14 (0-0.59)
Facilities + Linkages	99 (0-349)	43 (0-173)	0.15 (0-0.59)
Facilities + Linkages + Quality	103 (0-367)	44 (0-181)	0.15 (0-0.62)
Comprehensive	83 (0-317)	36 (0-154)	0.12 (0-0.53)

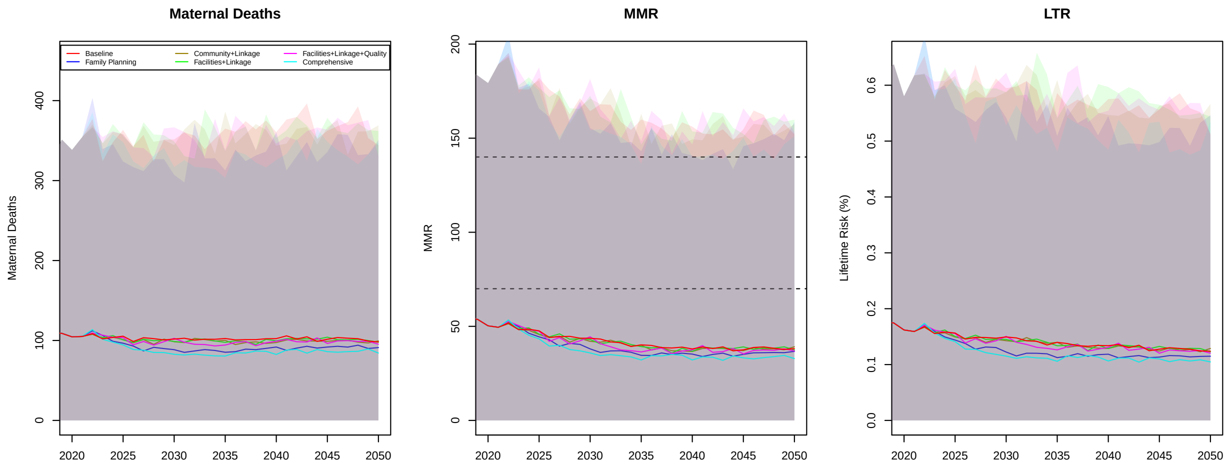

Liechtenstein

ISO Code	Region	Area	Income Group
LIE	Western Europe	Europe	High income

Projected Maternal Indicators in 2030 by Scenario

Scenario	Maternal Deaths	MMR	LTR
**Baseline**	0 (0-1)	16 (0-260)	0.03 (0-0.53)
**Family Planning Interventions**			
Contraception	0 (0-1)	17 (0-280)	0.03 (0-0.57)
Medical abortion	0 (0-1)	16 (0-260)	0.03 (0-0.53)
**Community-Based Interventions**			
ANC	0 (0-1)	17 (0-260)	0.03 (0-0.53)
SBA	0 (0-1)	16 (0-260)	0.03 (0-0.53)
**Facility-Based Interventions**			
Facility births	0 (0-1)	16 (0-260)	0.03 (0-0.53)
nonEmOC services	0 (0-1)	16 (0-260)	0.03 (0-0.53)
bEmOC services	0 (0-1)	16 (0-260)	0.03 (0-0.53)
cEmOC services	0 (0-1)	16 (0-260)	0.03 (0-0.53)
**System-Relevant Interventions**			
Quality of care	0 (0-1)	16 (0-260)	0.03 (0-0.53)
Referral	0 (0-1)	16 (0-260)	0.03 (0-0.53)
Transport	0 (0-1)	17 (0-262)	0.04 (0-0.54)
Targeted transfers	0 (0-1)	16 (0-260)	0.03 (0-0.53)
**Integrated Strategies**			
Family Planning	0 (0-1)	17 (0-280)	0.03 (0-0.57)
Community + Linkages	0 (0-1)	17 (0-262)	0.04 (0-0.54)
Facilities + Linkages	0 (0-1)	18 (0-262)	0.04 (0-0.54)
Facilities + Linkages + Quality	0 (0-1)	17 (0-262)	0.04 (0-0.54)
Comprehensive	0 (0-1)	17 (0-279)	0.03 (0-0.55)

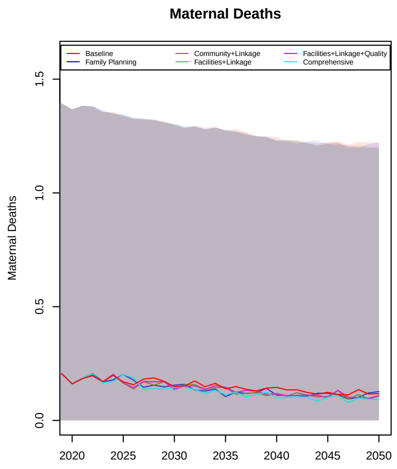

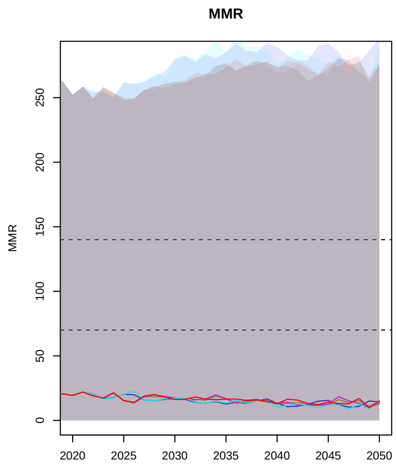

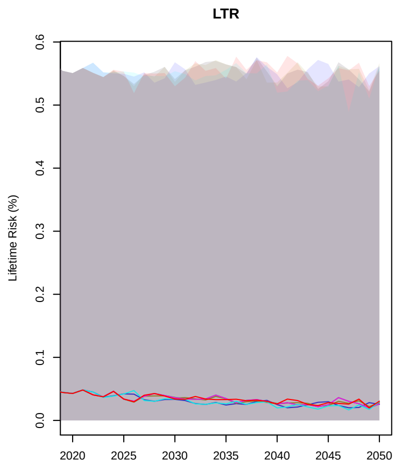

Lithuania

ISO Code	Region	Area	Income Group
LTU	Northern Europe	Europe	High income

Projected Maternal Indicators in 2030 by Scenario

Scenario	Maternal Deaths	MMR	LTR
**Baseline**	8 (0-24)	26 (0-94)	0.03 (0-0.13)
**Family Planning Interventions**			
Contraception	7 (0-21)	25 (0-90)	0.03 (0-0.11)
Medical abortion	8 (0-24)	26 (0-96)	0.03 (0-0.13)
**Community-Based Interventions**			
ANC	7 (0-22)	25 (0-92)	0.03 (0-0.12)
SBA	8 (0-24)	26 (0-93)	0.03 (0-0.13)
**Facility-Based Interventions**			
Facility births	8 (0-23)	26 (0-93)	0.03 (0-0.12)
nonEmOC services	8 (0-24)	26 (0-94)	0.03 (0-0.13)
bEmOC services	8 (0-24)	26 (0-94)	0.03 (0-0.13)
cEmOC services	7 (0-24)	25 (0-92)	0.03 (0-0.12)
**System-Relevant Interventions**			
Quality of care	8 (0-24)	26 (0-93)	0.03 (0-0.13)
Referral	8 (0-23)	27 (0-93)	0.03 (0-0.12)
Transport	7 (0-24)	26 (0-93)	0.03 (0-0.13)
Targeted transfers	8 (0-24)	26 (0-94)	0.03 (0-0.13)
**Integrated Strategies**			
Family Planning	7 (0-20)	25 (0-88)	0.03 (0-0.11)
Community + Linkages	7 (0-22)	26 (0-95)	0.03 (0-0.12)
Facilities + Linkages	7 (0-23)	26 (0-93)	0.03 (0-0.13)
Facilities + Linkages + Quality	7 (0-23)	26 (0-92)	0.03 (0-0.13)
Comprehensive	6 (0-20)	24 (0-90)	0.03 (0-0.11)

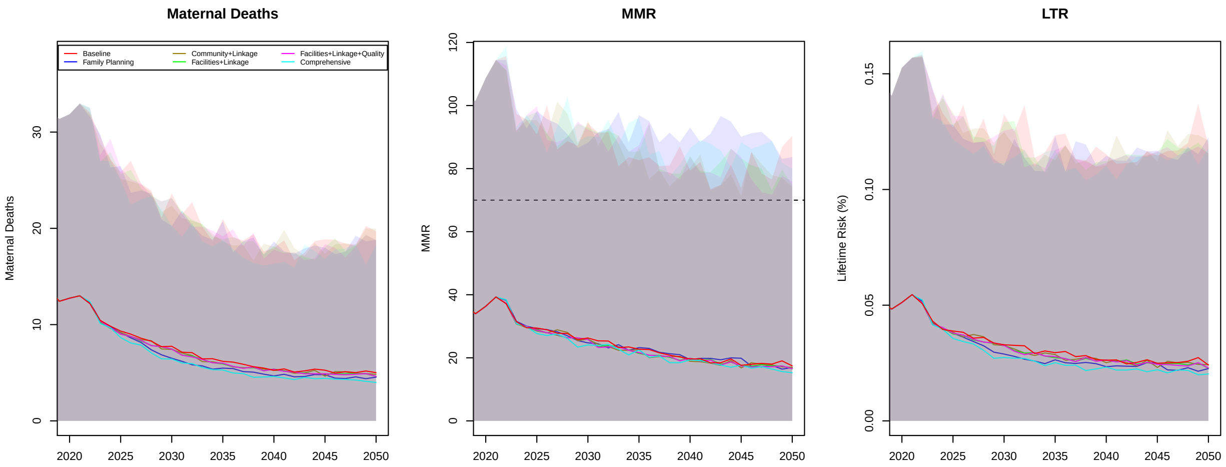

Luxembourg

ISO Code	Region	Area	Income Group
LUX	Western Europe	Europe	High income

Projected Maternal Indicators in 2030 by Scenario

Scenario	Maternal Deaths	MMR	LTR
**Baseline**	2 (0-11)	13 (0-163)	0.02 (0-0.24)
**Family Planning Interventions**			
Contraception	2 (0-11)	10 (0-156)	0.01 (0-0.22)
Medical abortion	2 (0-11)	13 (0-163)	0.02 (0-0.24)
**Community-Based Interventions**			
ANC	2 (0-11)	12 (0-161)	0.02 (0-0.23)
SBA	2 (0-11)	13 (0-163)	0.02 (0-0.24)
**Facility-Based Interventions**			
Facility births	2 (0-11)	13 (0-163)	0.02 (0-0.24)
nonEmOC services	2 (0-11)	13 (0-163)	0.02 (0-0.24)
bEmOC services	2 (0-11)	13 (0-163)	0.02 (0-0.24)
cEmOC services	2 (0-11)	13 (0-160)	0.02 (0-0.23)
**System-Relevant Interventions**			
Quality of care	2 (0-11)	14 (0-164)	0.02 (0-0.23)
Referral	2 (0-11)	13 (0-163)	0.02 (0-0.24)
Transport	2 (0-11)	12 (0-154)	0.02 (0-0.23)
Targeted transfers	2 (0-11)	13 (0-163)	0.02 (0-0.24)
**Integrated Strategies**			
Family Planning	2 (0-11)	10 (0-156)	0.02 (0-0.22)
Community + Linkages	2 (0-10)	11 (0-152)	0.02 (0-0.23)
Facilities + Linkages	2 (0-11)	12 (0-154)	0.02 (0-0.23)
Facilities + Linkages + Quality	2 (0-11)	12 (0-155)	0.02 (0-0.23)
Comprehensive	1 (0-11)	9 (0-153)	0.01 (0-0.22)

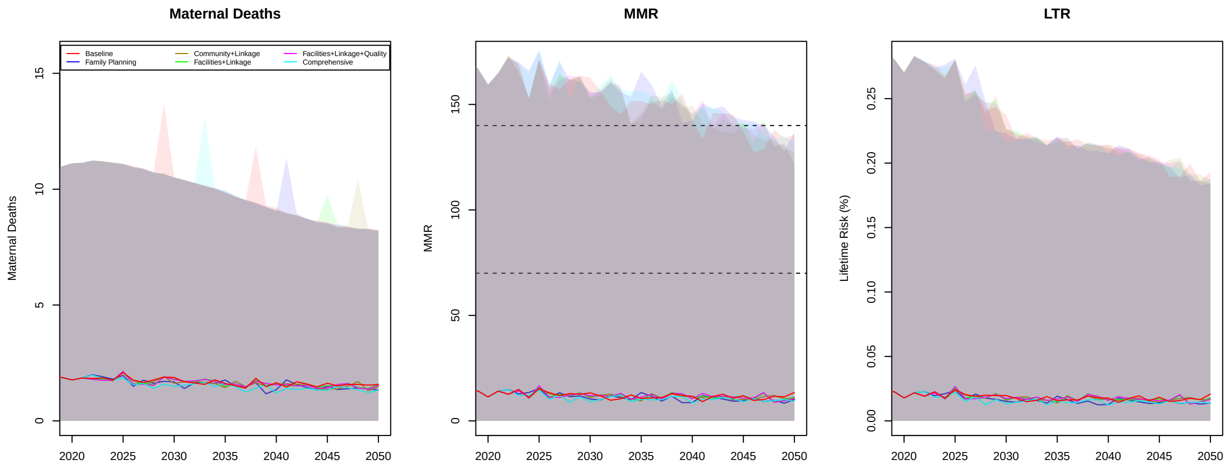

Madagascar

ISO Code	Region	Area	Income Group
MDG	Eastern Africa	Africa	Low income

Projected Maternal Indicators in 2030 by Scenario

Scenario	Maternal Deaths	MMR	LTR
**Baseline**	5023 (2933-7711)	410 (260-597)	1.36 (0.72-2.18)
**Family Planning Interventions**			
Contraception	4620 (2629-7270)	404 (240-594)	1.24 (0.65-2.07)
Medical abortion	5078 (2828-7942)	413 (257-600)	1.38 (0.71-2.25)
**Community-Based Interventions**			
ANC	5060 (2896-7887)	412 (255-614)	1.37 (0.75-2.19)
SBA	4519 (2552-6934)	365 (233-524)	1.22 (0.65-1.93)
**Facility-Based Interventions**			
Facility births	2716 (1386-4413)	203 (109-311)	0.69 (0.31-1.19)
nonEmOC services	5068 (2827-7946)	412 (253-612)	1.37 (0.7-2.19)
bEmOC services	5077 (2827-7996)	414 (250-600)	1.38 (0.71-2.24)
cEmOC services	5024 (2880-7662)	410 (250-606)	1.37 (0.74-2.22)
**System-Relevant Interventions**			
Quality of care	3948 (1878-6769)	313 (149-524)	1.03 (0.41-1.84)
Referral	4964 (2751-7793)	404 (247-596)	1.34 (0.72-2.19)
Transport	4697 (2629-7228)	381 (231-564)	1.27 (0.64-2.11)
Targeted transfers	5075 (2835-7628)	413 (258-614)	1.38 (0.71-2.21)
**Integrated Strategies**			
Family Planning	4591 (2468-7497)	401 (243-601)	1.24 (0.64-2.17)
Community + Linkages	3887 (2057-5934)	310 (180-448)	1.04 (0.52-1.62)
Facilities + Linkages	2490 (1204-3881)	186 (95-285)	0.63 (0.25-1.02)
Facilities + Linkages + Quality	833 (242-1631)	42 (6-91)	0.12 (0-0.33)
Comprehensive	746 (206-1536)	38 (0-88)	0.1 (0-0.3)

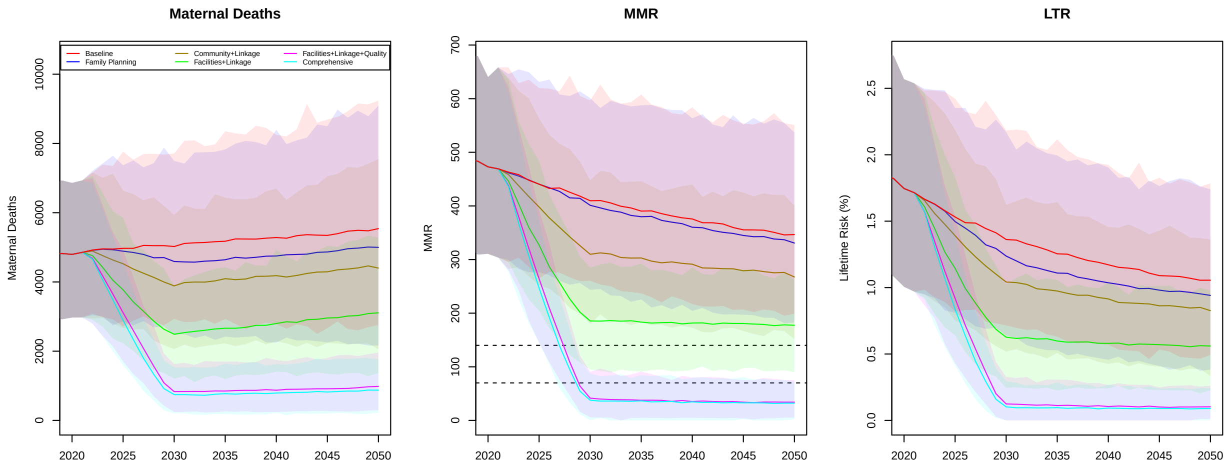

Malawi

ISO Code	Region	Area	Income Group
MWI	Eastern Africa	Africa	Low income

Projected Maternal Indicators in 2030 by Scenario

Scenario	Maternal Deaths	MMR	LTR
**Baseline**	2118 (1013-3897)	261 (117-512)	1 (0.42-1.96)
**Family Planning Interventions**			
Contraception	1803 (891-3101)	224 (117-366)	0.84 (0.36-1.42)
Medical abortion	1838 (897-2930)	221 (110-343)	0.86 (0.38-1.39)
**Community-Based Interventions**			
ANC	2082 (929-3924)	256 (116-499)	0.99 (0.4-1.84)
SBA	2107 (1053-3947)	260 (125-517)	0.99 (0.44-1.88)
**Facility-Based Interventions**			
Facility births	2035 (885-3839)	249 (111-510)	0.96 (0.38-1.9)
nonEmOC services	2084 (973-3811)	257 (117-498)	0.99 (0.42-1.88)
bEmOC services	2109 (994-3871)	259 (121-502)	1 (0.43-1.93)
cEmOC services	2061 (972-3888)	253 (124-506)	0.97 (0.42-1.93)
**System-Relevant Interventions**			
Quality of care	2091 (893-4000)	257 (115-525)	0.99 (0.4-1.93)
Referral	2129 (986-3903)	262 (124-521)	1.01 (0.43-1.91)
Transport	2020 (908-4017)	252 (114-527)	0.97 (0.4-1.96)
Targeted transfers	2106 (954-3897)	259 (122-532)	1 (0.44-1.93)
**Integrated Strategies**			
Family Planning	1670 (759-2758)	207 (94-317)	0.78 (0.3-1.29)
Community + Linkages	1937 (920-3661)	243 (106-486)	0.93 (0.37-1.81)
Facilities + Linkages	1947 (864-3815)	242 (105-488)	0.93 (0.37-1.81)
Facilities + Linkages + Quality	1940 (883-3684)	241 (103-507)	0.93 (0.36-1.87)
Comprehensive	1502 (678-2399)	187 (79-284)	0.71 (0.29-1.17)

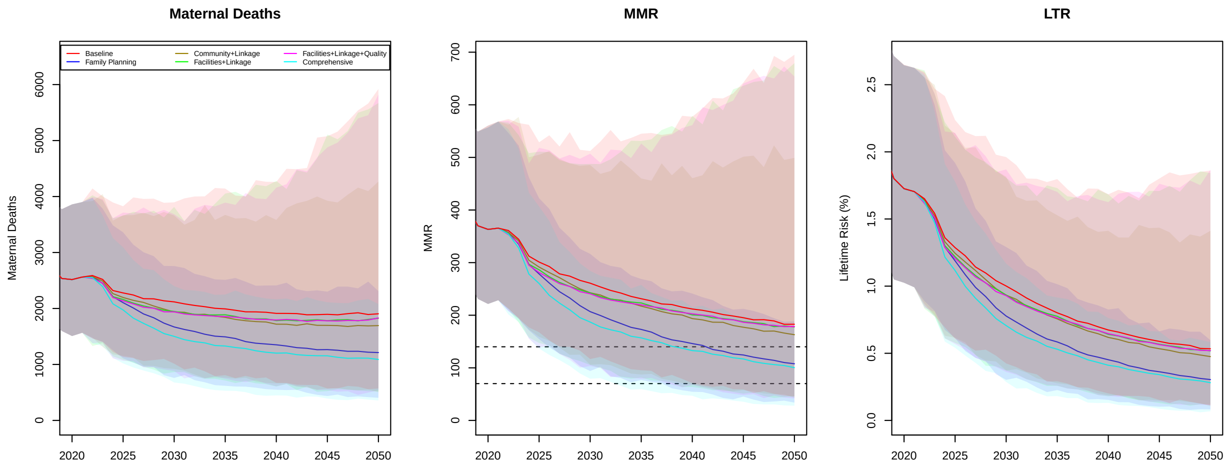

Malaysia

ISO Code	Region	Area	Income Group
MYS	South-Eastern Asia	Asia	Upper middle income

Projected Maternal Indicators in 2030 by Scenario

Scenario	Maternal Deaths	MMR	LTR
**Baseline**	171 (17-429)	18 (0-55)	0.03 (0-0.1)
**Family Planning Interventions**			
Contraception	173 (12-433)	17 (0-54)	0.03 (0-0.11)
Medical abortion	169 (0-423)	17 (0-53)	0.03 (0-0.09)
**Community-Based Interventions**			
ANC	171 (17-436)	17 (0-50)	0.03 (0-0.11)
SBA	176 (12-435)	18 (0-55)	0.03 (0-0.1)
**Facility-Based Interventions**			
Facility births	174 (17-435)	18 (0-55)	0.03 (0-0.1)
nonEmOC services	174 (0-446)	18 (0-55)	0.03 (0-0.1)
bEmOC services	172 (17-427)	17 (0-54)	0.03 (0-0.1)
cEmOC services	171 (12-426)	17 (0-50)	0.03 (0-0.1)
**System-Relevant Interventions**			
Quality of care	172 (11-437)	18 (0-55)	0.03 (0-0.11)
Referral	173 (17-445)	18 (0-55)	0.03 (0-0.1)
Transport	169 (0-421)	17 (0-49)	0.03 (0-0.1)
Targeted transfers	174 (17-437)	18 (0-55)	0.03 (0-0.11)
**Integrated Strategies**			
Family Planning	165 (0-413)	17 (0-50)	0.03 (0-0.1)
Community + Linkages	157 (0-408)	16 (0-50)	0.03 (0-0.1)
Facilities + Linkages	166 (0-412)	16 (0-49)	0.03 (0-0.1)
Facilities + Linkages + Quality	160 (0-405)	16 (0-51)	0.03 (0-0.1)
Comprehensive	153 (0-393)	15 (0-53)	0.03 (0-0.1)

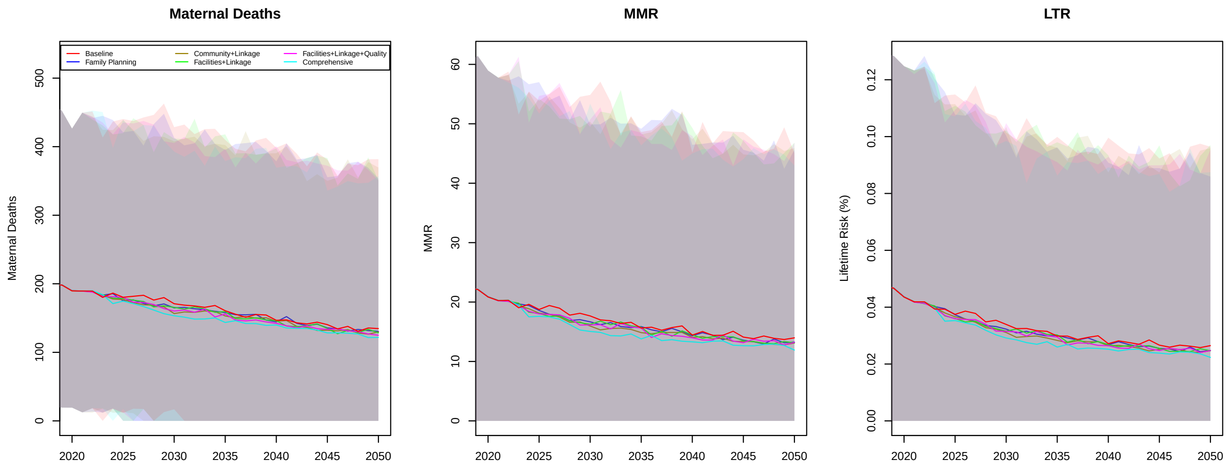

Maldives

ISO Code	Region	Area	Income Group
MDV	Southern Asia	Asia	Upper middle income

Projected Maternal Indicators in 2030 by Scenario

Scenario	Maternal Deaths	MMR	LTR
**Baseline**	13 (0-52)	127 (0-525)	0.31 (0-1.39)
**Family Planning Interventions**			
Contraception	7 (0-25)	61 (0-271)	0.14 (0-0.65)
Medical abortion	8 (0-26)	75 (0-284)	0.18 (0-0.67)
**Community-Based Interventions**			
ANC	12 (0-46)	123 (0-449)	0.3 (0-1.18)
SBA	13 (0-52)	128 (0-521)	0.31 (0-1.37)
**Facility-Based Interventions**			
Facility births	13 (0-48)	126 (0-498)	0.31 (0-1.29)
nonEmOC services	13 (0-51)	129 (0-526)	0.31 (0-1.39)
bEmOC services	13 (0-52)	128 (0-526)	0.31 (0-1.43)
cEmOC services	12 (0-49)	124 (0-507)	0.3 (0-1.29)
**System-Relevant Interventions**			
Quality of care	13 (0-52)	128 (0-525)	0.31 (0-1.37)
Referral	13 (0-51)	128 (0-510)	0.31 (0-1.37)
Transport	13 (0-56)	129 (0-542)	0.32 (0-1.49)
Targeted transfers	13 (0-53)	127 (0-526)	0.31 (0-1.43)
**Integrated Strategies**			
Family Planning	5 (0-19)	36 (0-176)	0.08 (0-0.39)
Community + Linkages	12 (0-44)	117 (0-449)	0.29 (0-1.1)
Facilities + Linkages	12 (0-50)	125 (0-512)	0.31 (0-1.32)
Facilities + Linkages + Quality	12 (0-51)	125 (0-512)	0.31 (0-1.32)
Comprehensive	5 (0-20)	37 (0-197)	0.09 (0-0.45)

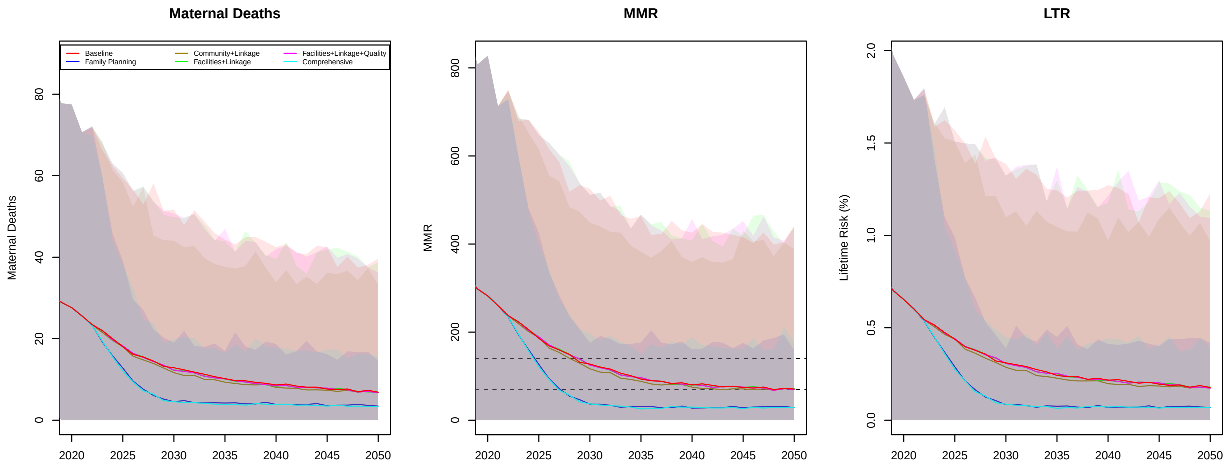

Mali

ISO Code	Region	Area	Income Group
MLI	Western Africa	Africa	Low income

Projected Maternal Indicators in 2030 by Scenario

Scenario	Maternal Deaths	MMR	LTR
**Baseline**	4221 (2596-5756)	319 (188-433)	1.58 (0.9-2.19)
**Family Planning Interventions**			
Contraception	3668 (1980-5317)	300 (178-409)	1.33 (0.67-2.03)
Medical abortion	4219 (2581-5709)	318 (194-421)	1.58 (0.91-2.19)
**Community-Based Interventions**			
ANC	4106 (2500-5681)	310 (179-420)	1.54 (0.86-2.14)
SBA	3988 (2543-5418)	299 (178-395)	1.49 (0.85-2.03)
**Facility-Based Interventions**			
Facility births	2481 (1674-3472)	173 (112-255)	0.85 (0.54-1.27)
nonEmOC services	4216 (2558-5778)	318 (181-430)	1.59 (0.88-2.2)
bEmOC services	4201 (2578-5699)	317 (187-428)	1.58 (0.9-2.16)
cEmOC services	4186 (2571-5761)	316 (186-423)	1.58 (0.87-2.19)
**System-Relevant Interventions**			
Quality of care	3278 (1605-4683)	240 (102-350)	1.19 (0.5-1.75)
Referral	4178 (2594-5710)	315 (189-415)	1.57 (0.91-2.18)
Transport	3861 (2426-5293)	290 (176-387)	1.44 (0.82-2.03)
Targeted transfers	4193 (2450-5695)	316 (183-427)	1.58 (0.87-2.17)
**Integrated Strategies**			
Family Planning	3677 (1952-5357)	300 (178-413)	1.33 (0.64-2.01)
Community + Linkages	3111 (2122-4219)	228 (147-309)	1.12 (0.69-1.58)
Facilities + Linkages	2220 (1444-3192)	152 (93-221)	0.75 (0.44-1.15)
Facilities + Linkages + Quality	962 (572-1504)	48 (24-86)	0.22 (0.08-0.44)
Comprehensive	818 (446-1269)	43 (18-72)	0.16 (0.05-0.32)

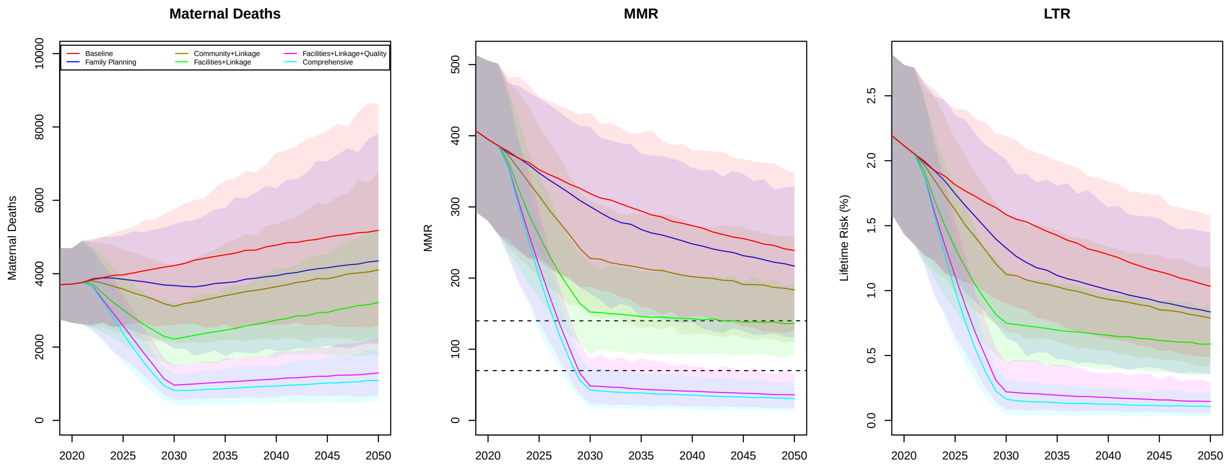

Malta

ISO Code	Region	Area	Income Group
MLT	Southern Europe	Europe	High income

Projected Maternal Indicators in 2030 by Scenario

Scenario	Maternal Deaths	MMR	LTR
**Baseline**	2 (0-8)	18 (0-188)	0.02 (0-0.24)
**Family Planning Interventions**			
Contraception	2 (0-11)	18 (0-198)	0.02 (0-0.25)
Medical abortion	2 (0-8)	17 (0-188)	0.02 (0-0.23)
**Community-Based Interventions**			
ANC	2 (0-8)	17 (0-183)	0.02 (0-0.24)
SBA	2 (0-11)	18 (0-188)	0.02 (0-0.25)
**Facility-Based Interventions**			
Facility births	1 (0-8)	16 (0-183)	0.02 (0-0.23)
nonEmOC services	2 (0-8)	18 (0-188)	0.02 (0-0.24)
bEmOC services	2 (0-8)	18 (0-188)	0.02 (0-0.24)
cEmOC services	2 (0-8)	20 (0-199)	0.03 (0-0.26)
**System-Relevant Interventions**			
Quality of care	2 (0-8)	18 (0-188)	0.02 (0-0.24)
Referral	2 (0-8)	18 (0-188)	0.02 (0-0.24)
Transport	1 (0-8)	15 (0-188)	0.02 (0-0.23)
Targeted transfers	2 (0-8)	18 (0-188)	0.02 (0-0.24)
**Integrated Strategies**			
Family Planning	2 (0-8)	17 (0-196)	0.02 (0-0.25)
Community + Linkages	1 (0-8)	16 (0-188)	0.02 (0-0.24)
Facilities + Linkages	1 (0-8)	14 (0-181)	0.02 (0-0.23)
Facilities + Linkages + Quality	1 (0-8)	14 (0-181)	0.02 (0-0.23)
Comprehensive	1 (0-8)	18 (0-196)	0.02 (0-0.24)

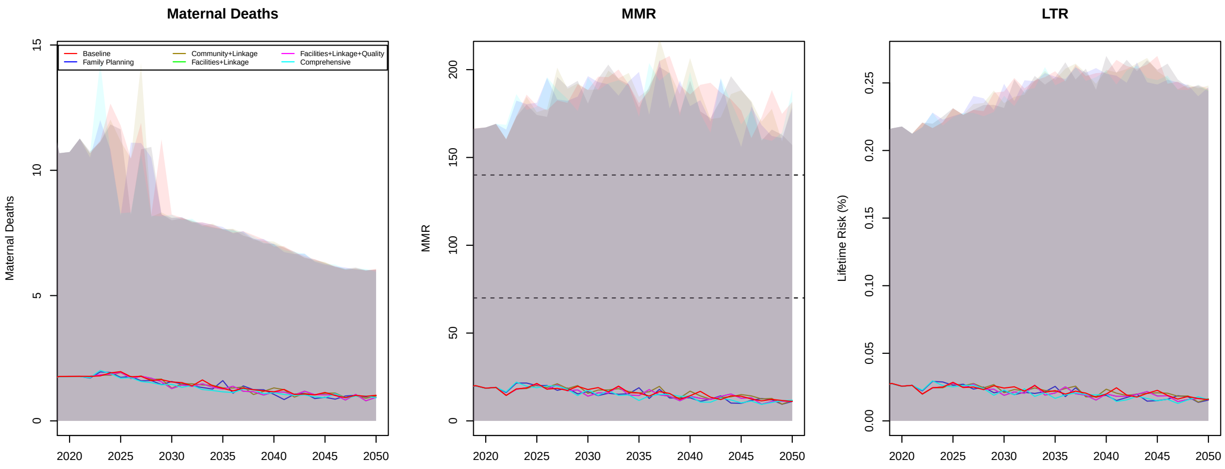

Marshall Islands

ISO Code	Region	Area	Income Group
MHL	Micronesia	Oceania	Upper middle income

Projected Maternal Indicators in 2030 by Scenario

Scenario	Maternal Deaths	MMR	LTR
**Baseline**	1 (0-4)	289 (0-1525)	0.38 (0-1.19)
**Family Planning Interventions**			
Contraception	1 (0-3)	197 (0-658)	0.32 (0-0.99)
Medical abortion	1 (0-3)	210 (0-737)	0.32 (0-1.01)
**Community-Based Interventions**			
ANC	1 (0-4)	279 (0-1426)	0.37 (0-1.23)
SBA	1 (0-4)	289 (0-1525)	0.38 (0-1.19)
**Facility-Based Interventions**			
Facility births	1 (0-4)	288 (0-1525)	0.38 (0-1.19)
nonEmOC services	1 (0-4)	290 (0-1525)	0.38 (0-1.21)
bEmOC services	1 (0-4)	289 (0-1525)	0.38 (0-1.19)
cEmOC services	1 (0-4)	289 (0-1520)	0.38 (0-1.21)
**System-Relevant Interventions**			
Quality of care	1 (0-4)	291 (0-1520)	0.38 (0-1.18)
Referral	1 (0-4)	288 (0-1525)	0.38 (0-1.19)
Transport	1 (0-5)	292 (0-1525)	0.39 (0-1.3)
Targeted transfers	1 (0-4)	287 (0-1525)	0.38 (0-1.19)
**Integrated Strategies**			
Family Planning	1 (0-3)	178 (0-621)	0.29 (0-0.91)
Community + Linkages	1 (0-4)	277 (0-1426)	0.37 (0-1.23)
Facilities + Linkages	1 (0-4)	289 (0-1506)	0.39 (0-1.29)
Facilities + Linkages + Quality	1 (0-4)	280 (0-1505)	0.38 (0-1.3)
Comprehensive	1 (0-3)	178 (0-606)	0.28 (0-0.91)

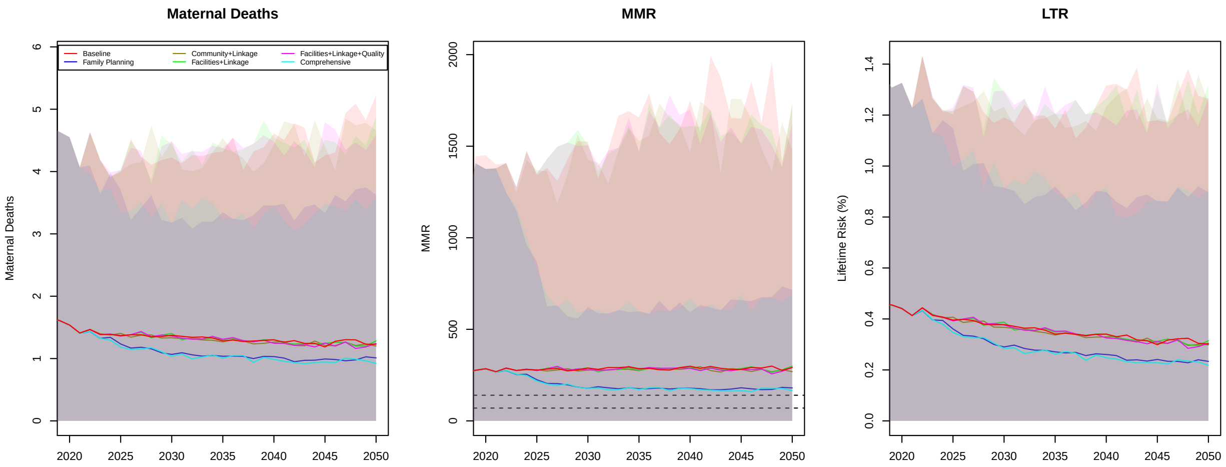

Mauritania

ISO Code	Region	Area	Income Group
MRT	Western Africa	Africa	Lower middle income

Projected Maternal Indicators in 2030 by Scenario

Scenario	Maternal Deaths	MMR	LTR
**Baseline**	528 (253-884)	207 (101-382)	0.96 (0.41-1.7)
**Family Planning Interventions**			
Contraception	446 (214-760)	185 (90-295)	0.79 (0.33-1.38)
Medical abortion	470 (235-765)	181 (91-284)	0.84 (0.35-1.43)
**Community-Based Interventions**			
ANC	515 (242-864)	202 (94-366)	0.93 (0.37-1.61)
SBA	520 (246-893)	203 (97-385)	0.95 (0.39-1.66)
**Facility-Based Interventions**			
Facility births	452 (204-747)	174 (67-337)	0.82 (0.29-1.46)
nonEmOC services	529 (257-884)	207 (97-388)	0.97 (0.4-1.73)
bEmOC services	529 (249-892)	208 (96-392)	0.97 (0.4-1.73)
cEmOC services	518 (244-895)	203 (97-397)	0.95 (0.38-1.75)
**System-Relevant Interventions**			
Quality of care	414 (158-774)	159 (54-338)	0.76 (0.2-1.51)
Referral	528 (255-872)	207 (96-390)	0.97 (0.4-1.68)
Transport	508 (247-841)	200 (92-365)	0.93 (0.38-1.61)
Targeted transfers	530 (259-861)	207 (103-379)	0.97 (0.41-1.66)
**Integrated Strategies**			
Family Planning	418 (190-702)	171 (80-269)	0.73 (0.28-1.28)
Community + Linkages	479 (227-776)	188 (85-336)	0.88 (0.36-1.52)
Facilities + Linkages	436 (192-738)	170 (65-325)	0.79 (0.29-1.42)
Facilities + Linkages + Quality	315 (84-635)	118 (20-278)	0.58 (0.08-1.25)
Comprehensive	223 (63-437)	85 (16-164)	0.38 (0.05-0.82)

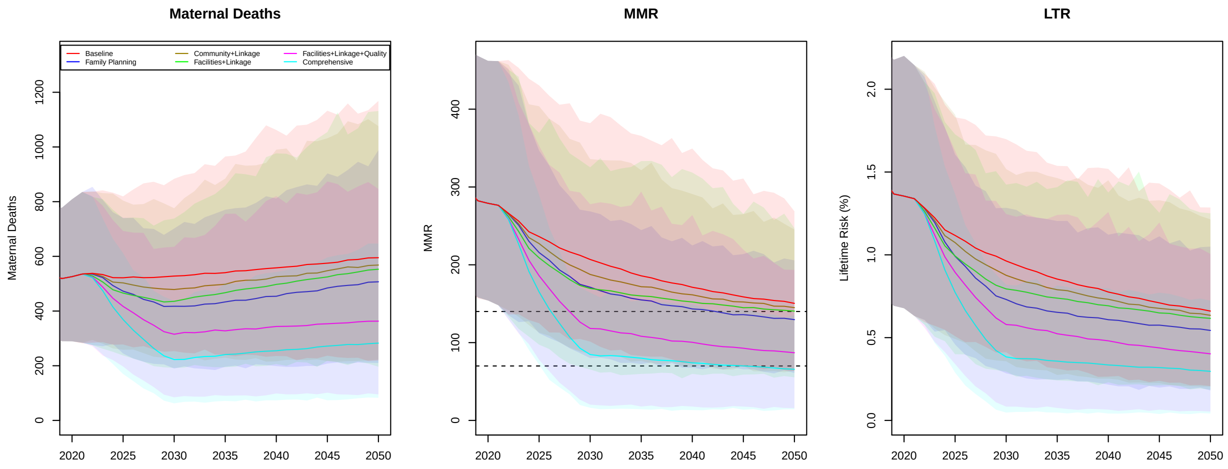

Mauritius

ISO Code	Region	Area	Income Group
MUS	Eastern Africa	Africa	Upper middle income

Projected Maternal Indicators in 2030 by Scenario

Scenario	Maternal Deaths	MMR	LTR
**Baseline**	15 (0-54)	100 (0-389)	0.15 (0-0.55)
**Family Planning Interventions**			
Contraception	13 (0-44)	86 (0-358)	0.12 (0-0.44)
Medical abortion	10 (0-31)	60 (0-219)	0.09 (0-0.32)
**Community-Based Interventions**			
ANC	14 (0-48)	94 (0-364)	0.14 (0-0.51)
SBA	15 (0-53)	97 (0-389)	0.14 (0-0.56)
**Facility-Based Interventions**			
Facility births	14 (0-54)	97 (0-398)	0.14 (0-0.55)
nonEmOC services	15 (0-53)	101 (0-389)	0.15 (0-0.53)
bEmOC services	15 (0-53)	99 (0-375)	0.14 (0-0.55)
cEmOC services	15 (0-54)	98 (0-386)	0.15 (0-0.57)
**System-Relevant Interventions**			
Quality of care	15 (0-54)	100 (0-389)	0.15 (0-0.55)
Referral	15 (0-54)	98 (0-377)	0.14 (0-0.55)
Transport	15 (0-54)	97 (0-377)	0.14 (0-0.57)
Targeted transfers	15 (0-51)	97 (0-375)	0.14 (0-0.53)
**Integrated Strategies**			
Family Planning	9 (0-32)	57 (0-198)	0.09 (0-0.31)
Community + Linkages	14 (0-49)	91 (0-354)	0.14 (0-0.5)
Facilities + Linkages	14 (0-49)	93 (0-362)	0.14 (0-0.52)
Facilities + Linkages + Quality	14 (0-49)	93 (0-362)	0.14 (0-0.52)
Comprehensive	8 (0-27)	48 (0-164)	0.07 (0-0.26)

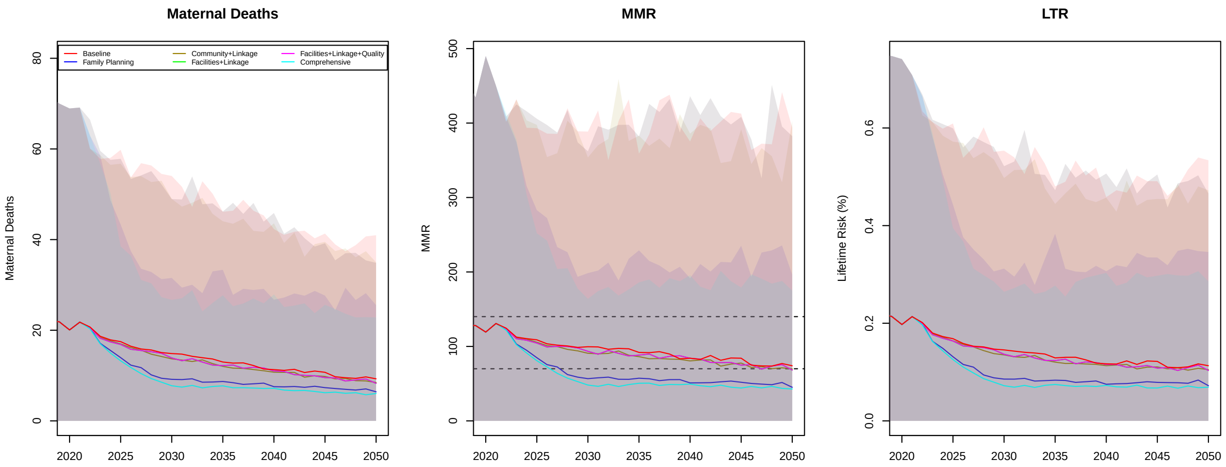

Mexico

ISO Code	Region	Area	Income Group
MEX	Central America	Latin America and the Caribbean	Upper middle income

Projected Maternal Indicators in 2030 by Scenario

Scenario	Maternal Deaths	MMR	LTR
**Baseline**	2365 (1260-3726)	83 (32-141)	0.19 (0.08-0.31)
**Family Planning Interventions**			
Contraception	2029 (1080-3229)	73 (31-123)	0.16 (0.07-0.27)
Medical abortion	2381 (1251-3713)	83 (33-138)	0.19 (0.08-0.31)
**Community-Based Interventions**			
ANC	2355 (1253-3645)	83 (32-139)	0.19 (0.09-0.3)
SBA	2339 (1260-3599)	82 (32-142)	0.19 (0.08-0.31)
**Facility-Based Interventions**			
Facility births	2311 (1189-3601)	81 (32-141)	0.19 (0.08-0.31)
nonEmOC services	2351 (1243-3646)	83 (32-144)	0.19 (0.08-0.31)
bEmOC services	2365 (1259-3770)	83 (31-143)	0.19 (0.08-0.31)
cEmOC services	2352 (1267-3680)	83 (31-142)	0.19 (0.08-0.32)
**System-Relevant Interventions**			
Quality of care	2272 (1165-3554)	80 (30-138)	0.18 (0.08-0.3)
Referral	2350 (1269-3659)	83 (31-142)	0.19 (0.08-0.31)
Transport	2328 (1221-3669)	82 (30-141)	0.19 (0.08-0.31)
Targeted transfers	2368 (1220-3682)	83 (32-143)	0.19 (0.08-0.31)
**Integrated Strategies**			
Family Planning	2025 (1026-3146)	72 (28-125)	0.16 (0.06-0.26)
Community + Linkages	2302 (1211-3573)	81 (30-138)	0.19 (0.08-0.3)
Facilities + Linkages	2275 (1179-3623)	80 (30-138)	0.18 (0.08-0.31)
Facilities + Linkages + Quality	2187 (1057-3517)	76 (28-134)	0.18 (0.07-0.29)
Comprehensive	1839 (875-2979)	65 (25-114)	0.14 (0.05-0.25)

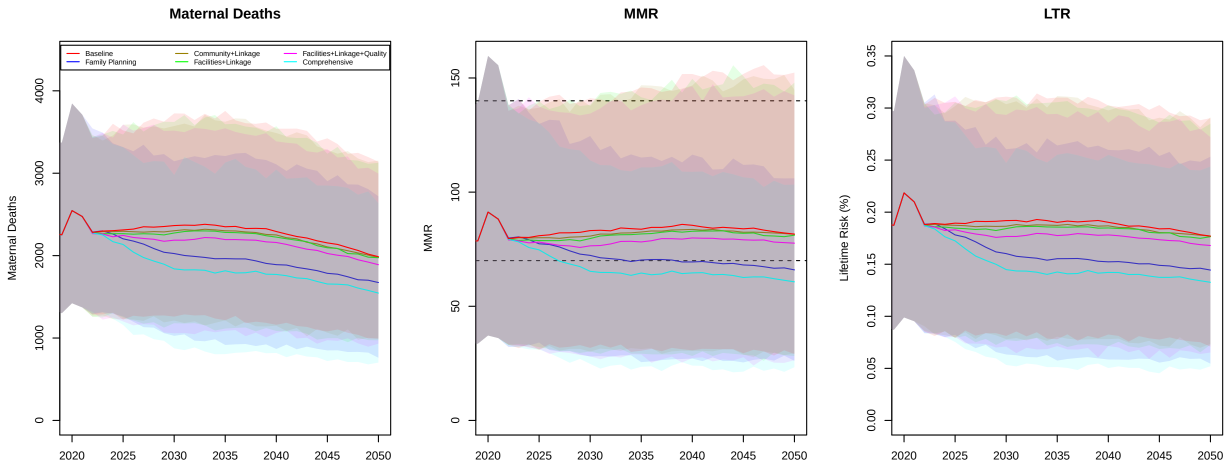

Micronesia (Fed. States of)

ISO Code	Region	Area	Income Group
FSM	Micronesia	Oceania	Lower middle income

Projected Maternal Indicators in 2030 by Scenario

Scenario	Maternal Deaths	MMR	LTR
**Baseline**	6 (0-14)	191 (0-491)	0.52 (0-1.42)
**Family Planning Interventions**			
Contraception	5 (0-13)	178 (0-479)	0.46 (0-1.25)
Medical abortion	6 (0-14)	188 (0-482)	0.51 (0-1.4)
**Community-Based Interventions**			
ANC	5 (0-13)	180 (0-470)	0.49 (0-1.32)
SBA	6 (0-14)	192 (0-501)	0.52 (0-1.43)
**Facility-Based Interventions**			
Facility births	6 (0-14)	190 (0-491)	0.51 (0-1.42)
nonEmOC services	6 (0-14)	193 (0-491)	0.52 (0-1.4)
bEmOC services	6 (0-14)	193 (0-501)	0.52 (0-1.41)
cEmOC services	5 (0-13)	175 (0-448)	0.47 (0-1.24)
**System-Relevant Interventions**			
Quality of care	3 (0-10)	104 (0-309)	0.29 (0-0.91)
Referral	6 (0-14)	191 (0-491)	0.51 (0-1.43)
Transport	5 (0-14)	184 (0-449)	0.49 (0-1.32)
Targeted transfers	6 (0-14)	189 (0-482)	0.51 (0-1.37)
**Integrated Strategies**			
Family Planning	5 (0-13)	176 (0-445)	0.46 (0-1.19)
Community + Linkages	5 (0-13)	175 (0-450)	0.47 (0-1.27)
Facilities + Linkages	5 (0-14)	173 (0-449)	0.47 (0-1.26)
Facilities + Linkages + Quality	3 (0-9)	87 (0-279)	0.24 (0-0.8)
Comprehensive	2 (0-7)	75 (0-252)	0.19 (0-0.68)

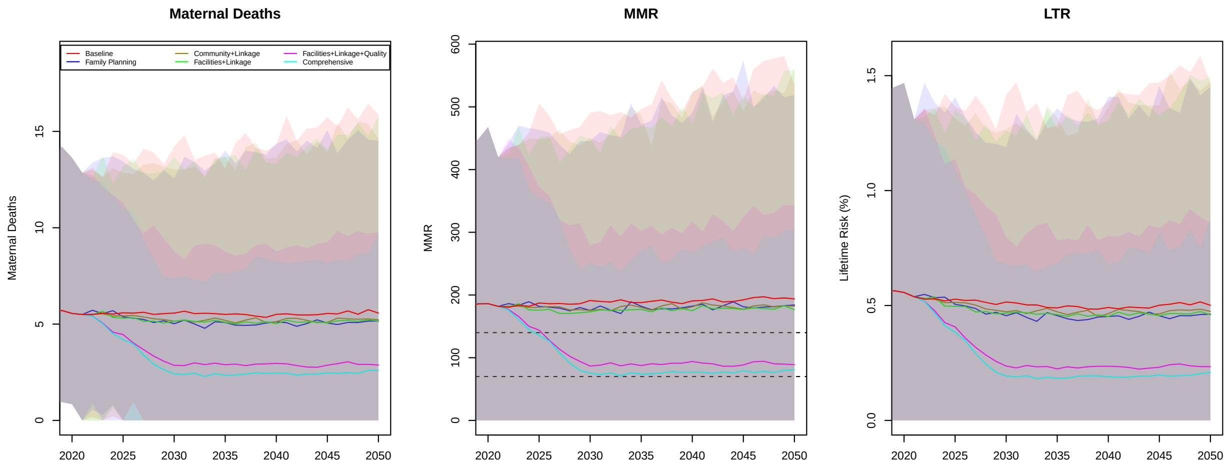

Monaco

ISO Code	Region	Area	Income Group
MCO	Western Europe	Europe	High income

Projected Maternal Indicators in 2030 by Scenario

Scenario	Maternal Deaths	MMR	LTR
**Baseline**	0 (0-1)	7 (0-0)	0.01 (0-0)
**Family Planning Interventions**			
Contraception	0 (0-1)	7 (0-0)	0.01 (0-0)
Medical abortion	0 (0-1)	7 (0-0)	0.01 (0-0)
**Community-Based Interventions**			
ANC	0 (0-1)	7 (0-0)	0.01 (0-0)
SBA	0 (0-1)	7 (0-0)	0.01 (0-0)
**Facility-Based Interventions**			
Facility births	0 (0-1)	7 (0-0)	0.01 (0-0)
nonEmOC services	0 (0-1)	7 (0-0)	0.01 (0-0)
bEmOC services	0 (0-1)	7 (0-0)	0.01 (0-0)
cEmOC services	0 (0-1)	7 (0-0)	0.01 (0-0)
**System-Relevant Interventions**			
Quality of care	0 (0-1)	7 (0-0)	0.01 (0-0)
Referral	0 (0-1)	7 (0-0)	0.01 (0-0)
Transport	0 (0-1)	7 (0-0)	0.01 (0-0)
Targeted transfers	0 (0-1)	7 (0-0)	0.01 (0-0)
**Integrated Strategies**			
Family Planning	0 (0-1)	8 (0-0)	0.01 (0-0)
Community + Linkages	0 (0-1)	7 (0-0)	0.01 (0-0)
Facilities + Linkages	0 (0-1)	7 (0-0)	0.01 (0-0)
Facilities + Linkages + Quality	0 (0-1)	7 (0-0)	0.01 (0-0)
Comprehensive	0 (0-1)	8 (0-0)	0.01 (0-0)

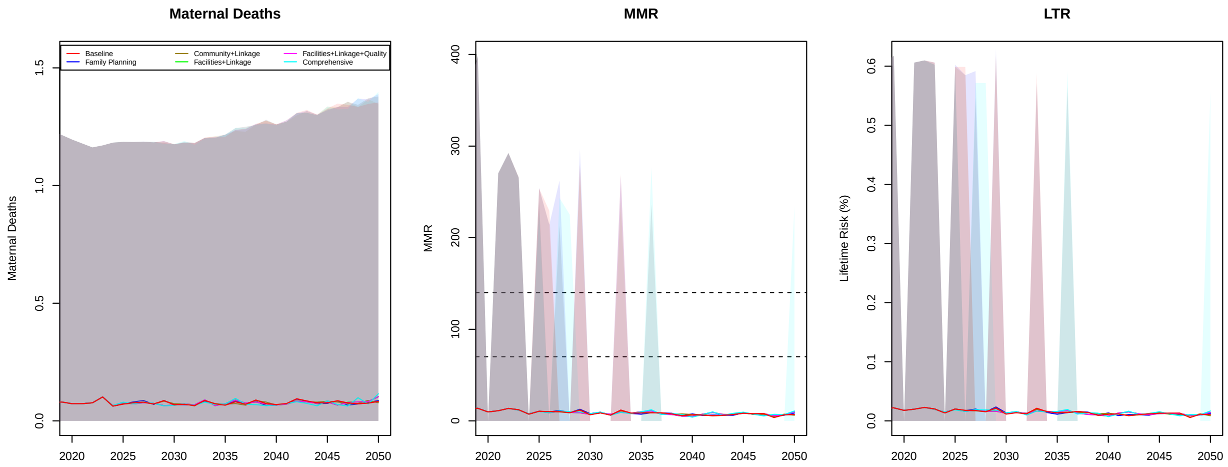

Mongolia

ISO Code	Region	Area	Income Group
MNG	Eastern Asia	Asia	Lower middle income

Projected Maternal Indicators in 2030 by Scenario

Scenario	Maternal Deaths	MMR	LTR
**Baseline**	39 (0-151)	23 (0-131)	0.06 (0-0.35)
**Family Planning Interventions**			
Contraception	42 (0-154)	23 (0-132)	0.06 (0-0.41)
Medical abortion	40 (0-151)	23 (0-134)	0.06 (0-0.35)
**Community-Based Interventions**			
ANC	38 (0-132)	23 (0-129)	0.06 (0-0.34)
SBA	41 (0-153)	25 (0-141)	0.07 (0-0.37)
**Facility-Based Interventions**			
Facility births	38 (0-150)	23 (0-136)	0.06 (0-0.37)
nonEmOC services	40 (0-151)	23 (0-130)	0.06 (0-0.35)
bEmOC services	40 (0-151)	24 (0-141)	0.06 (0-0.35)
cEmOC services	40 (0-151)	25 (0-131)	0.06 (0-0.36)
**System-Relevant Interventions**			
Quality of care	36 (0-149)	23 (0-130)	0.06 (0-0.34)
Referral	40 (0-151)	24 (0-134)	0.06 (0-0.37)
Transport	39 (0-150)	25 (0-134)	0.07 (0-0.39)
Targeted transfers	39 (0-151)	23 (0-130)	0.06 (0-0.35)
**Integrated Strategies**			
Family Planning	42 (0-155)	23 (0-131)	0.07 (0-0.4)
Community + Linkages	38 (0-132)	23 (0-134)	0.06 (0-0.36)
Facilities + Linkages	37 (0-145)	22 (0-133)	0.06 (0-0.39)
Facilities + Linkages + Quality	33 (0-149)	21 (0-126)	0.05 (0-0.33)
Comprehensive	32 (0-130)	17 (0-110)	0.05 (0-0.29)

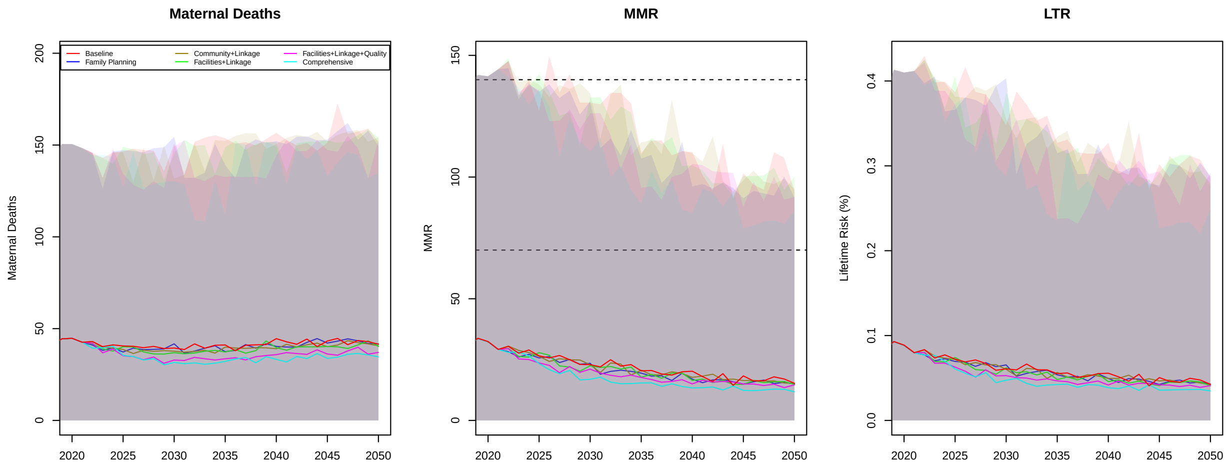

Montenegro

ISO Code	Region	Area	Income Group
MNE	Southern Europe	Europe	Upper middle income

Projected Maternal Indicators in 2030 by Scenario

Scenario	Maternal Deaths	MMR	LTR
**Baseline**	3 (0-13)	20 (0-117)	0.04 (0-0.22)
**Family Planning Interventions**			
Contraception	3 (0-14)	21 (0-127)	0.04 (0-0.23)
Medical abortion	3 (0-12)	20 (0-119)	0.04 (0-0.23)
**Community-Based Interventions**			
ANC	3 (0-14)	20 (0-114)	0.04 (0-0.22)
SBA	3 (0-13)	19 (0-117)	0.03 (0-0.22)
**Facility-Based Interventions**			
Facility births	3 (0-13)	19 (0-114)	0.03 (0-0.18)
nonEmOC services	3 (0-13)	19 (0-117)	0.04 (0-0.22)
bEmOC services	3 (0-13)	20 (0-118)	0.04 (0-0.22)
cEmOC services	3 (0-13)	20 (0-120)	0.04 (0-0.21)
**System-Relevant Interventions**			
Quality of care	3 (0-13)	20 (0-117)	0.04 (0-0.22)
Referral	3 (0-13)	20 (0-117)	0.04 (0-0.22)
Transport	3 (0-12)	20 (0-133)	0.04 (0-0.23)
Targeted transfers	3 (0-13)	21 (0-121)	0.04 (0-0.23)
**Integrated Strategies**			
Family Planning	3 (0-13)	21 (0-131)	0.04 (0-0.23)
Community + Linkages	3 (0-15)	20 (0-138)	0.04 (0-0.24)
Facilities + Linkages	3 (0-12)	19 (0-127)	0.04 (0-0.23)
Facilities + Linkages + Quality	3 (0-13)	19 (0-131)	0.04 (0-0.23)
Comprehensive	3 (0-13)	17 (0-106)	0.03 (0-0.19)

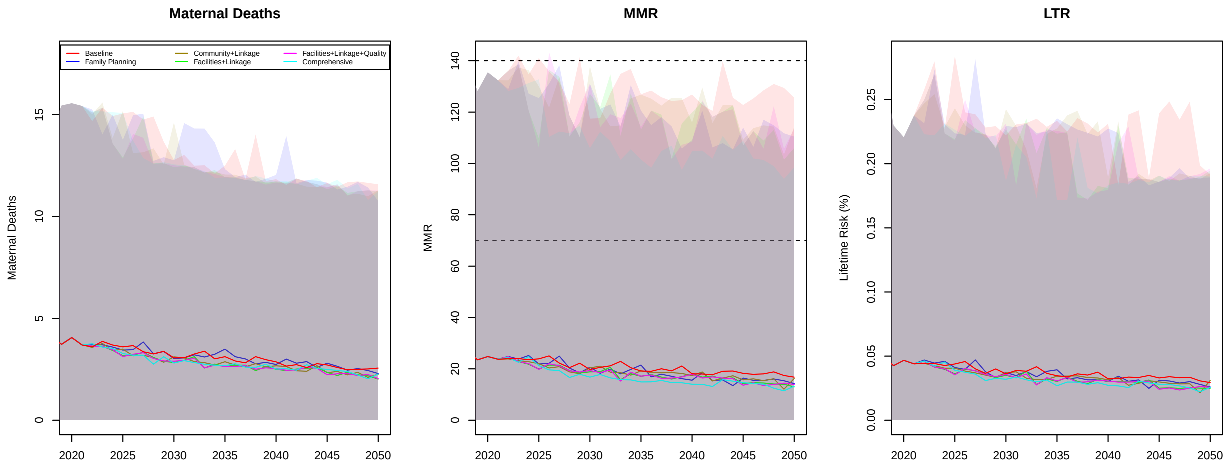

Morocco

ISO Code	Region	Area	Income Group
MAR	Northern Africa	Africa	Lower middle income

Projected Maternal Indicators in 2030 by Scenario

Scenario	Maternal Deaths	MMR	LTR
**Baseline**	614 (112-1660)	73 (2-229)	0.15 (0-0.5)
**Family Planning Interventions**			
Contraception	828 (138-2117)	86 (4-221)	0.22 (0.01-0.63)
Medical abortion	615 (111-1768)	73 (2-230)	0.15 (0-0.5)
**Community-Based Interventions**			
ANC	591 (108-1608)	70 (2-218)	0.14 (0-0.45)
SBA	499 (107-1197)	55 (2-152)	0.11 (0-0.34)
**Facility-Based Interventions**			
Facility births	302 (55-647)	24 (0-63)	0.05 (0-0.14)
nonEmOC services	609 (115-1831)	73 (1-239)	0.15 (0-0.52)
bEmOC services	611 (112-1654)	73 (1-230)	0.15 (0-0.49)
cEmOC services	591 (108-1676)	69 (2-220)	0.14 (0-0.47)
**System-Relevant Interventions**			
Quality of care	597 (110-1626)	70 (0-231)	0.15 (0-0.46)
Referral	607 (110-1710)	72 (2-231)	0.15 (0-0.48)
Transport	529 (112-1388)	61 (2-191)	0.13 (0-0.39)
Targeted transfers	616 (119-1692)	73 (2-232)	0.15 (0-0.49)
**Integrated Strategies**			
Family Planning	834 (137-2104)	86 (4-220)	0.22 (0.01-0.61)
Community + Linkages	420 (89-893)	45 (0-116)	0.09 (0-0.24)
Facilities + Linkages	254 (30-558)	19 (0-55)	0.04 (0-0.13)
Facilities + Linkages + Quality	256 (26-587)	19 (0-50)	0.04 (0-0.12)
Comprehensive	280 (46-585)	17 (0-47)	0.04 (0-0.12)

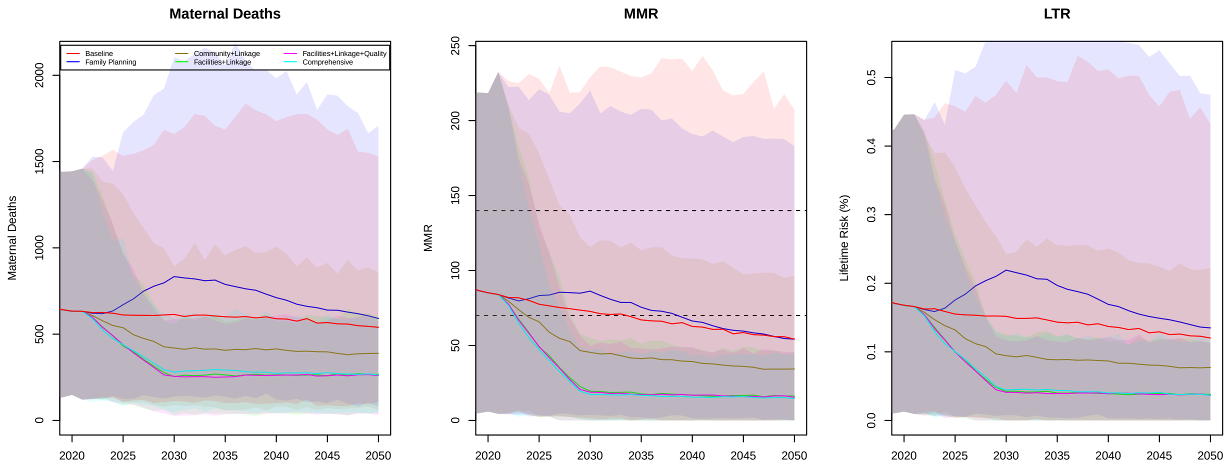

Mozambique

ISO Code	Region	Area	Income Group
MOZ	Eastern Africa	Africa	Low income

Projected Maternal Indicators in 2030 by Scenario

Scenario	Maternal Deaths	MMR	LTR
**Baseline**	5489 (3436-8453)	315 (195-483)	1.49 (0.85-2.39)
**Family Planning Interventions**			
Contraception	5145 (3039-7998)	300 (172-426)	1.38 (0.78-2.28)
Medical abortion	5182 (3188-7961)	295 (172-421)	1.38 (0.82-2.23)
**Community-Based Interventions**			
ANC	5261 (3259-7877)	300 (183-445)	1.42 (0.81-2.24)
SBA	5137 (3290-7771)	292 (185-443)	1.39 (0.79-2.23)
**Facility-Based Interventions**			
Facility births	3112 (1916-4491)	164 (101-273)	0.78 (0.43-1.24)
nonEmOC services	5473 (3487-8377)	314 (198-470)	1.49 (0.85-2.35)
bEmOC services	5484 (3451-8273)	315 (196-470)	1.49 (0.86-2.35)
cEmOC services	5341 (3318-8068)	305 (189-465)	1.45 (0.82-2.29)
**System-Relevant Interventions**			
Quality of care	4372 (2374-7082)	244 (118-383)	1.17 (0.53-1.93)
Referral	5421 (3394-8262)	311 (187-462)	1.47 (0.84-2.3)
Transport	5054 (3260-7515)	289 (181-422)	1.37 (0.79-2.13)
Targeted transfers	5468 (3405-8306)	314 (191-468)	1.49 (0.85-2.36)
**Integrated Strategies**			
Family Planning	5050 (2978-8018)	294 (164-433)	1.34 (0.74-2.24)
Community + Linkages	4038 (2652-5985)	224 (148-344)	1.06 (0.62-1.69)
Facilities + Linkages	2798 (1713-4166)	146 (88-251)	0.7 (0.37-1.15)
Facilities + Linkages + Quality	1431 (812-2636)	59 (26-157)	0.29 (0.11-0.74)
Comprehensive	1040 (694-1511)	36 (22-59)	0.16 (0.08-0.29)

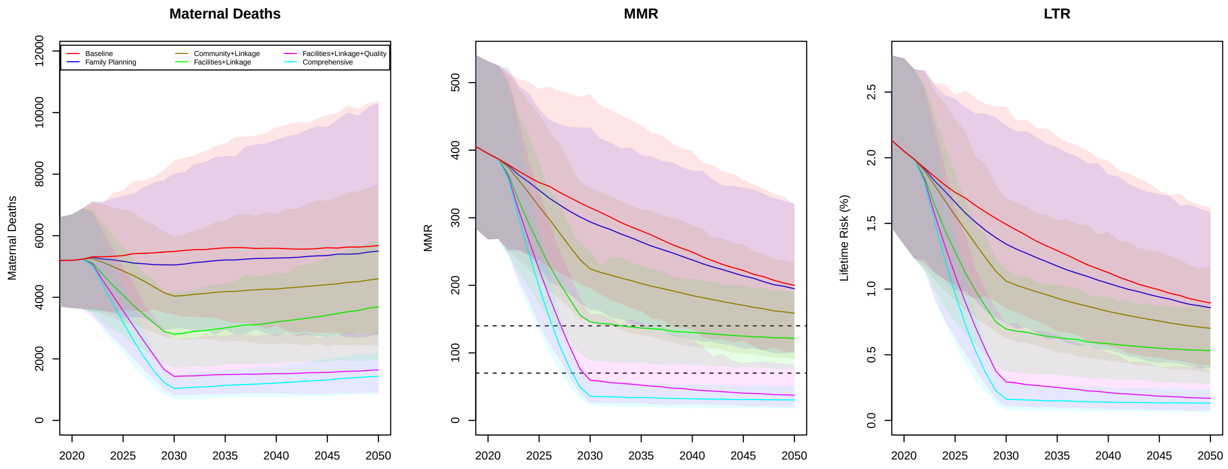

Myanmar

ISO Code	Region	Area	Income Group
MMR	South-Eastern Asia	Asia	Lower middle income

Projected Maternal Indicators in 2030 by Scenario

Scenario	Maternal Deaths	MMR	LTR
**Baseline**	910 (396-1707)	123 (46-201)	0.17 (0.06-0.32)
**Family Planning Interventions**			
Contraception	1049 (430-2401)	100 (34-227)	0.19 (0.06-0.48)
Medical abortion	907 (395-1746)	123 (45-202)	0.17 (0.06-0.33)
**Community-Based Interventions**			
ANC	852 (354-1650)	115 (40-188)	0.16 (0.06-0.32)
SBA	784 (357-1512)	104 (44-170)	0.14 (0.05-0.28)
**Facility-Based Interventions**			
Facility births	442 (195-785)	52 (19-96)	0.07 (0.02-0.13)
nonEmOC services	905 (398-1710)	123 (47-205)	0.17 (0.06-0.33)
bEmOC services	907 (378-1681)	123 (46-197)	0.17 (0.06-0.32)
cEmOC services	897 (374-1668)	122 (46-203)	0.17 (0.06-0.32)
**System-Relevant Interventions**			
Quality of care	899 (369-1702)	122 (43-199)	0.17 (0.06-0.32)
Referral	897 (391-1640)	121 (46-199)	0.17 (0.06-0.32)
Transport	775 (353-1449)	106 (44-171)	0.14 (0.06-0.29)
Targeted transfers	906 (381-1721)	123 (45-203)	0.17 (0.06-0.32)
**Integrated Strategies**			
Family Planning	1040 (420-2343)	99 (31-223)	0.19 (0.05-0.47)
Community + Linkages	624 (287-1205)	83 (37-136)	0.11 (0.04-0.22)
Facilities + Linkages	380 (165-718)	46 (15-89)	0.06 (0.02-0.12)
Facilities + Linkages + Quality	370 (155-656)	44 (16-81)	0.06 (0.02-0.11)
Comprehensive	357 (173-598)	27 (10-48)	0.05 (0.01-0.09)

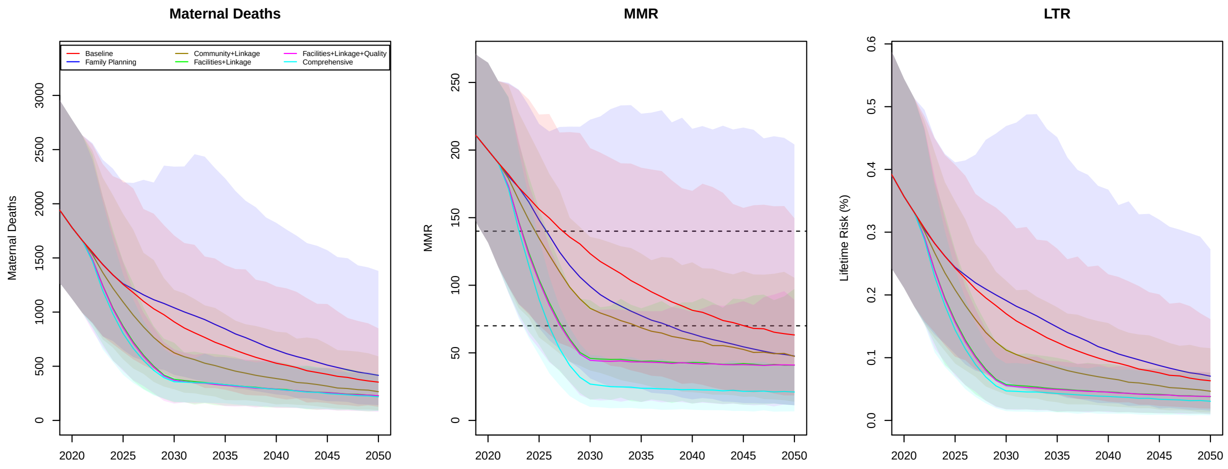

Namibia

ISO Code	Region	Area	Income Group
NAM	Southern Africa	Africa	Upper middle income

Projected Maternal Indicators in 2030 by Scenario

Scenario	Maternal Deaths	MMR	LTR
**Baseline**	55 (0-161)	74 (0-219)	0.21 (0-0.67)
**Family Planning Interventions**			
Contraception	40 (0-121)	55 (0-184)	0.14 (0-0.48)
Medical abortion	42 (0-119)	53 (0-171)	0.14 (0-0.48)
**Community-Based Interventions**			
ANC	55 (2-146)	74 (0-211)	0.21 (0-0.62)
SBA	55 (2-144)	73 (0-213)	0.21 (0-0.63)
**Facility-Based Interventions**			
Facility births	50 (0-139)	64 (0-195)	0.18 (0-0.59)
nonEmOC services	57 (0-157)	77 (0-235)	0.22 (0-0.66)
bEmOC services	55 (0-156)	76 (0-220)	0.21 (0-0.67)
cEmOC services	56 (0-163)	75 (0-215)	0.21 (0-0.68)
**System-Relevant Interventions**			
Quality of care	54 (0-153)	72 (0-216)	0.21 (0-0.64)
Referral	54 (0-153)	71 (0-204)	0.2 (0-0.62)
Transport	53 (0-153)	72 (0-203)	0.2 (0-0.62)
Targeted transfers	55 (0-160)	74 (0-225)	0.21 (0-0.69)
**Integrated Strategies**			
Family Planning	34 (0-115)	46 (0-163)	0.11 (0-0.42)
Community + Linkages	51 (0-147)	69 (0-206)	0.19 (0-0.6)
Facilities + Linkages	49 (0-150)	66 (0-211)	0.19 (0-0.61)
Facilities + Linkages + Quality	47 (0-139)	62 (0-199)	0.18 (0-0.57)
Comprehensive	28 (0-106)	37 (0-146)	0.09 (0-0.38)

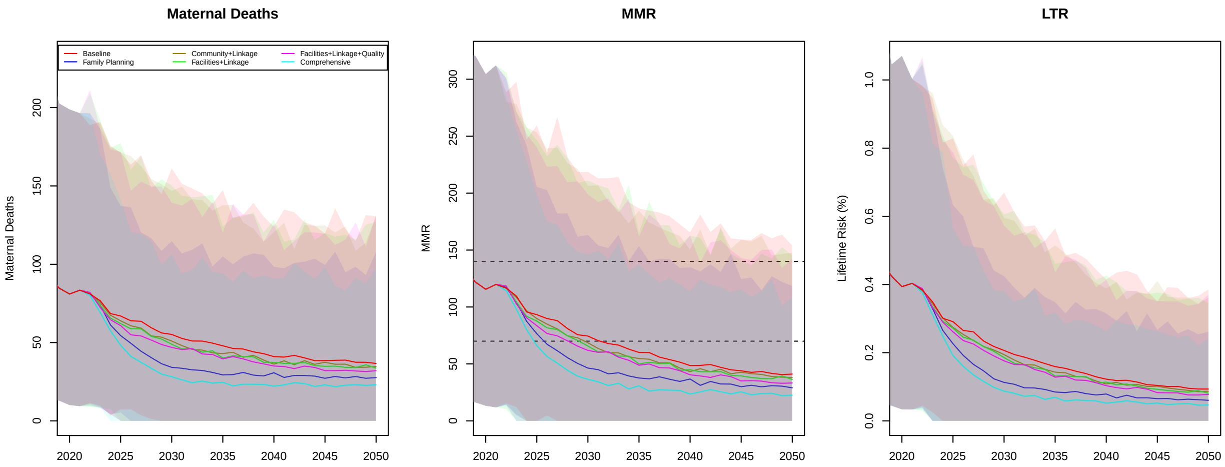

Nauru

ISO Code	Region	Area	Income Group
NRU	Micronesia	Oceania	Upper middle income

Projected Maternal Indicators in 2030 by Scenario

Scenario	Maternal Deaths	MMR	LTR
**Baseline**	1 (0-2)	189 (0-847)	0.51 (0-2.34)
**Family Planning Interventions**			
Contraception	0 (0-2)	184 (0-852)	0.45 (0-2.23)
Medical abortion	0 (0-2)	182 (0-840)	0.49 (0-2.35)
**Community-Based Interventions**			
ANC	1 (0-2)	193 (0-852)	0.52 (0-2.36)
SBA	1 (0-2)	189 (0-847)	0.51 (0-2.34)
**Facility-Based Interventions**			
Facility births	1 (0-2)	190 (0-847)	0.51 (0-2.32)
nonEmOC services	1 (0-2)	190 (0-847)	0.51 (0-2.34)
bEmOC services	0 (0-2)	188 (0-813)	0.5 (0-2.32)
cEmOC services	1 (0-2)	189 (0-860)	0.5 (0-2.3)
**System-Relevant Interventions**			
Quality of care	1 (0-2)	194 (0-866)	0.52 (0-2.36)
Referral	1 (0-2)	190 (0-852)	0.5 (0-2.34)
Transport	0 (0-2)	187 (0-813)	0.49 (0-2.28)
Targeted transfers	1 (0-2)	190 (0-847)	0.51 (0-2.34)
**Integrated Strategies**			
Family Planning	0 (0-2)	192 (0-855)	0.47 (0-2.25)
Community + Linkages	1 (0-2)	193 (0-840)	0.51 (0-2.29)
Facilities + Linkages	1 (0-2)	193 (0-860)	0.51 (0-2.27)
Facilities + Linkages + Quality	1 (0-2)	197 (0-860)	0.52 (0-2.29)
Comprehensive	0 (0-2)	167 (0-785)	0.42 (0-2.14)

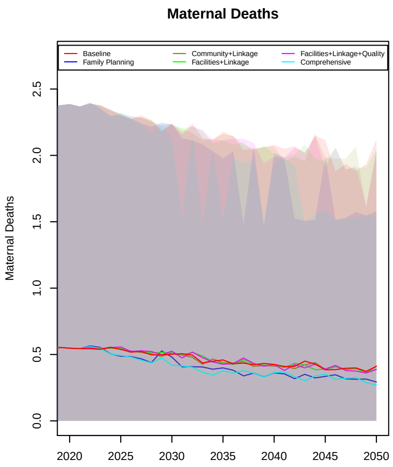

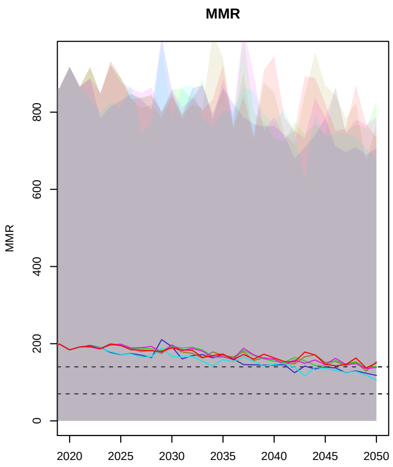

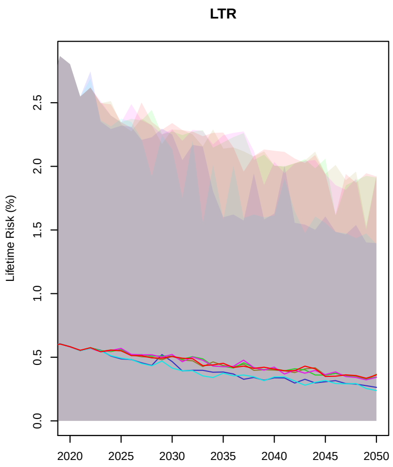

Nepal

ISO Code	Region	Area	Income Group
NPL	Southern Asia	Asia	Low income

Projected Maternal Indicators in 2030 by Scenario

Scenario	Maternal Deaths	MMR	LTR
**Baseline**	1298 (424-2565)	179 (55-366)	0.33 (0.09-0.73)
**Family Planning Interventions**			
Contraception	1204 (373-2622)	169 (54-346)	0.31 (0.08-0.71)
Medical abortion	1296 (440-2592)	177 (56-346)	0.33 (0.09-0.72)
**Community-Based Interventions**			
ANC	1260 (416-2475)	172 (50-342)	0.32 (0.09-0.71)
SBA	1243 (435-2394)	169 (54-333)	0.31 (0.09-0.66)
**Facility-Based Interventions**			
Facility births	805 (192-1720)	90 (9-210)	0.17 (0.01-0.44)
nonEmOC services	1302 (419-2565)	179 (55-362)	0.34 (0.08-0.72)
bEmOC services	1298 (406-2709)	178 (50-361)	0.33 (0.08-0.73)
cEmOC services	1256 (413-2508)	171 (47-337)	0.32 (0.08-0.71)
**System-Relevant Interventions**			
Quality of care	1072 (311-2338)	138 (35-307)	0.26 (0.05-0.67)
Referral	1284 (416-2528)	176 (58-352)	0.33 (0.08-0.72)
Transport	1184 (388-2409)	162 (47-332)	0.3 (0.07-0.67)
Targeted transfers	1304 (433-2589)	180 (58-366)	0.34 (0.09-0.72)
**Integrated Strategies**			
Family Planning	1204 (399-2560)	166 (49-351)	0.3 (0.07-0.72)
Community + Linkages	1042 (318-2108)	137 (39-286)	0.25 (0.06-0.56)
Facilities + Linkages	720 (193-1529)	79 (10-190)	0.14 (0.02-0.37)
Facilities + Linkages + Quality	445 (90-971)	31 (0-87)	0.06 (0-0.17)
Comprehensive	421 (52-963)	27 (0-80)	0.05 (0-0.16)

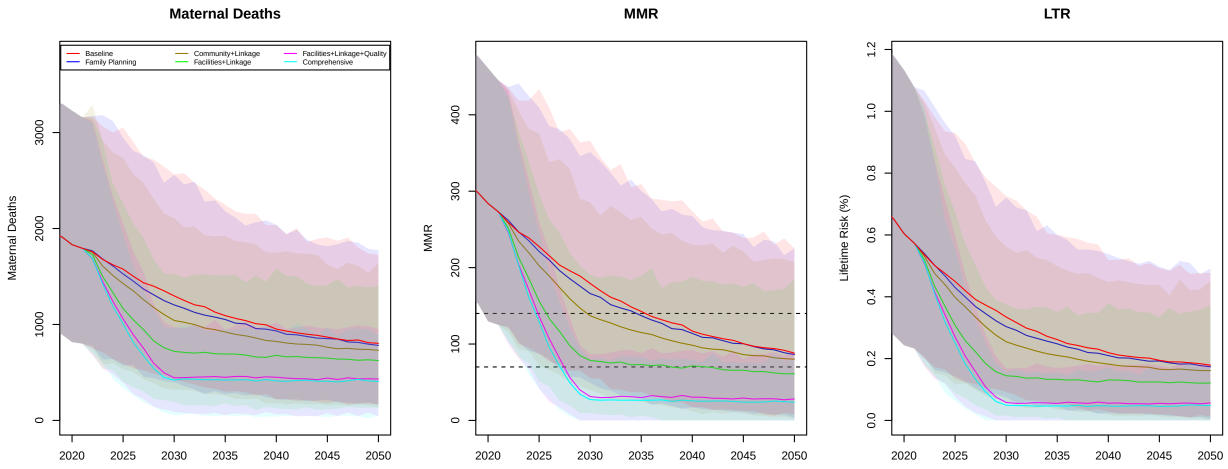

Netherlands

ISO Code	Region	Area	Income Group
NLD	Western Europe	Europe	High income

Projected Maternal Indicators in 2030 by Scenario

Scenario	Maternal Deaths	MMR	LTR
**Baseline**	82 (26-164)	23 (4-53)	0.05 (0.01-0.11)
**Family Planning Interventions**			
Contraception	81 (28-161)	23 (4-53)	0.04 (0.01-0.11)
Medical abortion	82 (26-160)	23 (4-51)	0.05 (0.01-0.11)
**Community-Based Interventions**			
ANC	82 (26-166)	23 (4-53)	0.05 (0.01-0.11)
SBA	82 (26-164)	23 (4-53)	0.05 (0.01-0.11)
**Facility-Based Interventions**			
Facility births	82 (26-164)	23 (4-53)	0.05 (0.01-0.11)
nonEmOC services	82 (26-165)	23 (4-54)	0.05 (0.01-0.11)
bEmOC services	82 (26-164)	23 (4-54)	0.05 (0.01-0.11)
cEmOC services	82 (29-174)	23 (4-55)	0.05 (0.01-0.12)
**System-Relevant Interventions**			
Quality of care	82 (26-165)	23 (4-54)	0.05 (0.01-0.11)
Referral	82 (26-165)	23 (4-53)	0.05 (0.01-0.11)
Transport	81 (26-166)	22 (4-53)	0.04 (0.01-0.11)
Targeted transfers	82 (26-164)	23 (4-53)	0.05 (0.01-0.11)
**Integrated Strategies**			
Family Planning	81 (29-156)	23 (4-53)	0.05 (0.01-0.11)
Community + Linkages	82 (26-170)	23 (4-56)	0.05 (0.01-0.12)
Facilities + Linkages	82 (26-163)	23 (4-52)	0.05 (0.01-0.12)
Facilities + Linkages + Quality	82 (25-163)	23 (4-53)	0.05 (0.01-0.12)
Comprehensive	79 (25-159)	22 (3-54)	0.04 (0.01-0.12)

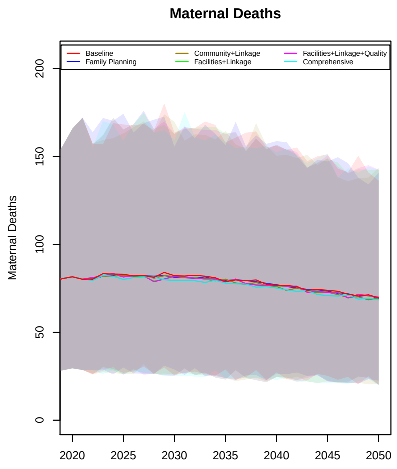

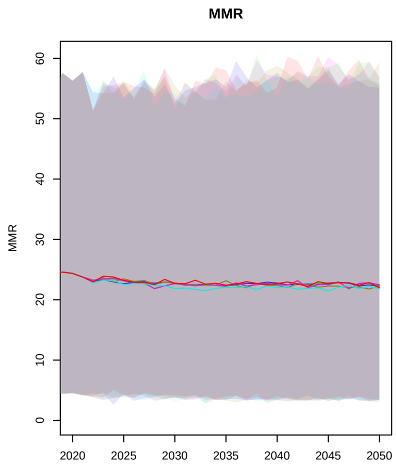

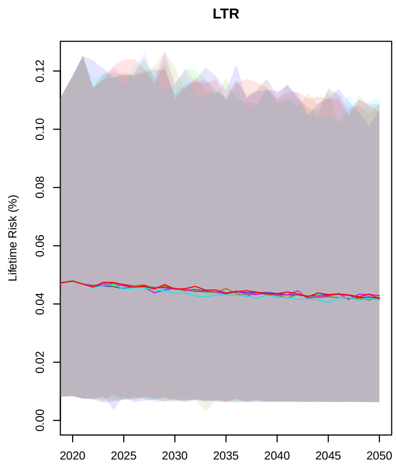

New Zealand

ISO Code	Region	Area	Income Group
NZL	Australia/New Zealand	Oceania	High income

Projected Maternal Indicators in 2030 by Scenario

Scenario	Maternal Deaths	MMR	LTR
**Baseline**	26 (0-98)	40 (0-164)	0.07 (0-0.27)
**Family Planning Interventions**			
Contraception	26 (0-102)	41 (0-164)	0.07 (0-0.28)
Medical abortion	26 (0-100)	40 (0-168)	0.07 (0-0.27)
**Community-Based Interventions**			
ANC	26 (0-97)	39 (0-163)	0.07 (0-0.25)
SBA	26 (0-98)	40 (0-164)	0.07 (0-0.27)
**Facility-Based Interventions**			
Facility births	26 (0-98)	40 (0-164)	0.07 (0-0.27)
nonEmOC services	26 (0-98)	40 (0-164)	0.07 (0-0.27)
bEmOC services	27 (0-98)	41 (0-166)	0.07 (0-0.27)
cEmOC services	27 (0-101)	41 (0-166)	0.07 (0-0.27)
**System-Relevant Interventions**			
Quality of care	26 (0-97)	41 (0-162)	0.07 (0-0.24)
Referral	26 (0-98)	40 (0-164)	0.07 (0-0.27)
Transport	28 (0-100)	42 (0-168)	0.07 (0-0.28)
Targeted transfers	26 (0-98)	41 (0-164)	0.07 (0-0.27)
**Integrated Strategies**			
Family Planning	25 (0-97)	39 (0-152)	0.06 (0-0.23)
Community + Linkages	26 (0-96)	40 (0-159)	0.07 (0-0.23)
Facilities + Linkages	27 (0-105)	41 (0-168)	0.07 (0-0.29)
Facilities + Linkages + Quality	26 (0-93)	41 (0-152)	0.07 (0-0.26)
Comprehensive	24 (0-82)	38 (0-153)	0.06 (0-0.23)

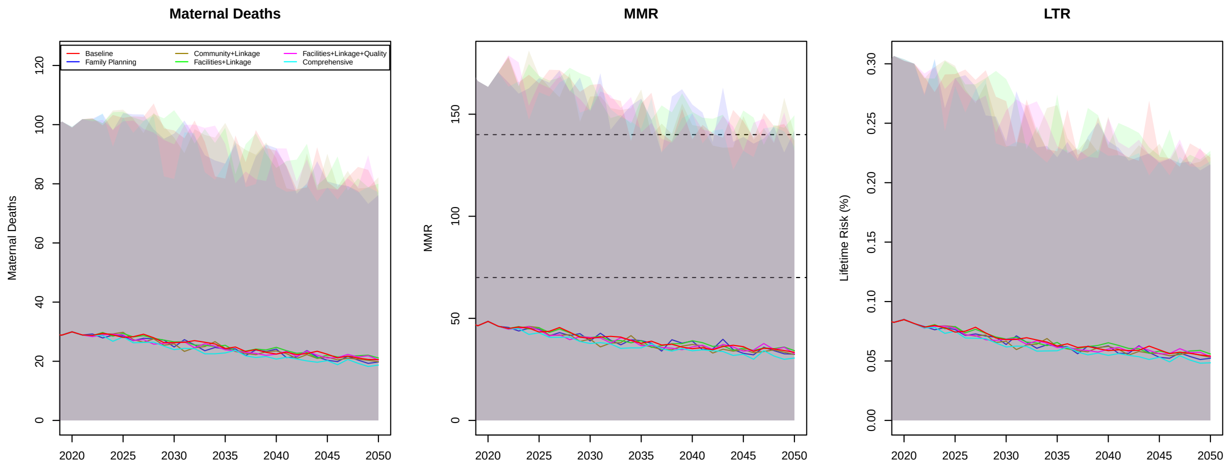

Nicaragua

ISO Code	Region	Area	Income Group
NIC	Central America	Latin America and the Caribbean	Lower middle income

Projected Maternal Indicators in 2030 by Scenario

Scenario	Maternal Deaths	MMR	LTR
**Baseline**	237 (74-535)	125 (25-283)	0.29 (0.06-0.72)
**Family Planning Interventions**			
Contraception	212 (60-445)	116 (24-274)	0.25 (0.05-0.61)
Medical abortion	235 (74-540)	124 (24-300)	0.29 (0.05-0.73)
**Community-Based Interventions**			
ANC	235 (65-532)	124 (27-281)	0.29 (0.05-0.75)
SBA	208 (62-437)	105 (26-240)	0.24 (0.05-0.59)
**Facility-Based Interventions**			
Facility births	136 (31-288)	57 (3-142)	0.13 (0-0.34)
nonEmOC services	233 (75-505)	122 (24-274)	0.28 (0.06-0.7)
bEmOC services	236 (71-535)	124 (27-285)	0.29 (0.06-0.73)
cEmOC services	228 (64-513)	120 (24-286)	0.28 (0.05-0.72)
**System-Relevant Interventions**			
Quality of care	230 (71-526)	124 (29-283)	0.29 (0.06-0.67)
Referral	236 (69-531)	125 (25-289)	0.29 (0.05-0.72)
Transport	208 (60-439)	109 (25-235)	0.25 (0.05-0.6)
Targeted transfers	235 (75-498)	125 (25-276)	0.29 (0.06-0.68)
**Integrated Strategies**			
Family Planning	208 (61-475)	114 (22-278)	0.25 (0.04-0.63)
Community + Linkages	184 (49-391)	92 (18-214)	0.21 (0.03-0.53)
Facilities + Linkages	123 (28-263)	52 (0-137)	0.12 (0-0.32)
Facilities + Linkages + Quality	120 (27-255)	51 (0-130)	0.12 (0-0.3)
Comprehensive	106 (20-230)	45 (0-118)	0.1 (0-0.26)

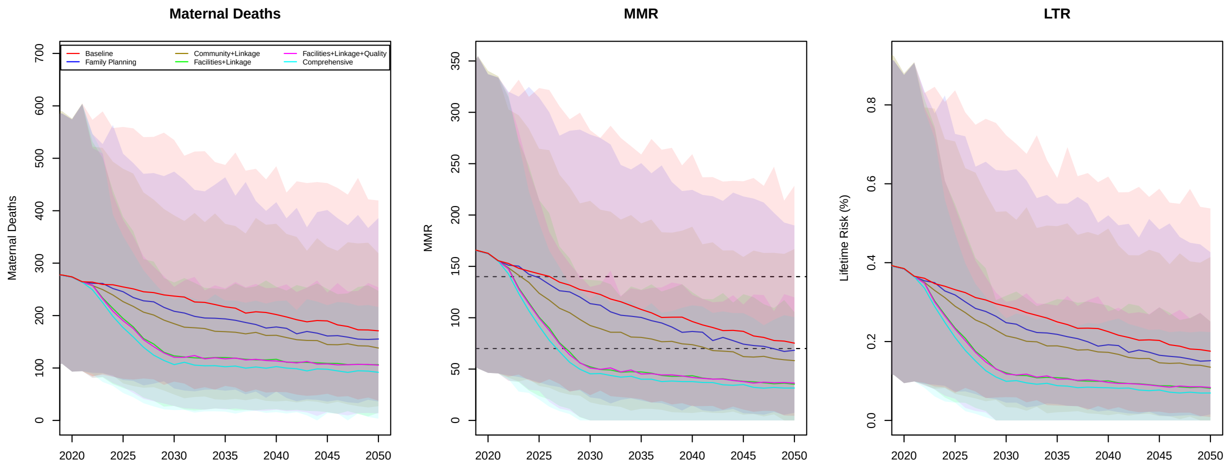

Niger

ISO Code	Region	Area	Income Group
NER	Western Africa	Africa	Low income

Projected Maternal Indicators in 2030 by Scenario

Scenario	Maternal Deaths	MMR	LTR
**Baseline**	6032 (2620-11653)	331 (132-665)	1.87 (0.7-3.79)
**Family Planning Interventions**			
Contraception	5883 (2667-11387)	329 (133-652)	1.83 (0.74-3.69)
Medical abortion	6032 (2581-11699)	331 (129-664)	1.87 (0.71-3.79)
**Community-Based Interventions**			
ANC	5930 (2573-11575)	325 (130-655)	1.83 (0.72-3.75)
SBA	5866 (2600-11198)	321 (127-620)	1.82 (0.73-3.6)
**Facility-Based Interventions**			
Facility births	3278 (1961-5167)	164 (87-273)	0.93 (0.46-1.63)
nonEmOC services	6002 (2663-11746)	329 (135-675)	1.86 (0.73-3.79)
bEmOC services	6064 (2697-11955)	333 (130-667)	1.88 (0.73-3.84)
cEmOC services	6047 (2540-11658)	331 (127-657)	1.87 (0.7-3.75)
**System-Relevant Interventions**			
Quality of care	4611 (1361-11231)	243 (50-640)	1.37 (0.26-3.6)
Referral	5858 (2616-11594)	321 (130-669)	1.82 (0.73-3.73)
Transport	5389 (2498-10083)	294 (127-581)	1.66 (0.66-3.27)
Targeted transfers	6049 (2580-11708)	333 (129-663)	1.88 (0.7-3.82)
**Integrated Strategies**			
Family Planning	5924 (2591-11657)	332 (133-666)	1.84 (0.7-3.72)
Community + Linkages	4422 (2277-7449)	235 (110-409)	1.33 (0.62-2.37)
Facilities + Linkages	3063 (1808-4779)	153 (82-249)	0.86 (0.43-1.49)
Facilities + Linkages + Quality	1254 (621-2229)	43 (14-103)	0.24 (0.05-0.66)
Comprehensive	1191 (564-2120)	39 (11-91)	0.21 (0.03-0.51)

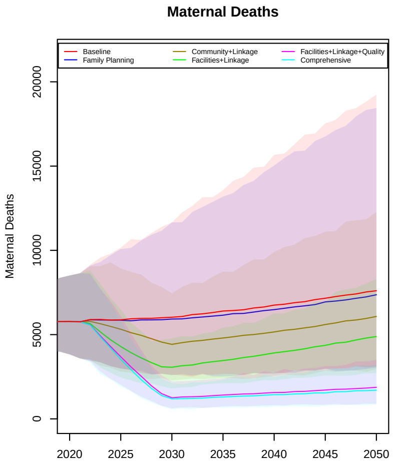

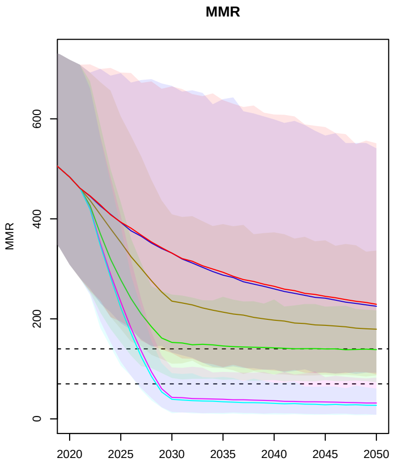

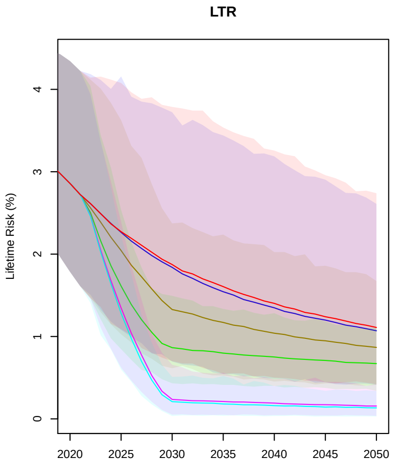

Nigeria

ISO Code	Region	Area	Income Group
NGA	Western Africa	Africa	Lower middle income

Projected Maternal Indicators in 2030 by Scenario

Scenario	Maternal Deaths	MMR	LTR
**Baseline**	60553 (39857-77277)	459 (305-569)	2.8 (1.77-3.61)
**Family Planning Interventions**			
Contraception	56721 (36972-72759)	447 (296-553)	2.6 (1.61-3.35)
Medical abortion	60716 (40792-77177)	460 (308-564)	2.81 (1.79-3.59)
**Community-Based Interventions**			
ANC	60367 (41382-75748)	458 (306-563)	2.79 (1.82-3.55)
SBA	58208 (38322-73346)	440 (289-551)	2.7 (1.68-3.43)
**Facility-Based Interventions**			
Facility births	48450 (29733-63038)	362 (221-467)	2.26 (1.32-3)
nonEmOC services	60583 (39401-77133)	459 (299-567)	2.8 (1.73-3.59)
bEmOC services	60248 (40833-77326)	456 (306-565)	2.79 (1.8-3.58)
cEmOC services	59912 (39366-76324)	453 (300-567)	2.77 (1.72-3.53)
**System-Relevant Interventions**			
Quality of care	55113 (35138-70610)	416 (265-522)	2.57 (1.57-3.33)
Referral	59889 (40351-75610)	454 (300-559)	2.77 (1.74-3.54)
Transport	58251 (38927-73276)	442 (288-543)	2.71 (1.68-3.44)
Targeted transfers	60599 (40244-77588)	460 (300-567)	2.81 (1.76-3.59)
**Integrated Strategies**			
Family Planning	56676 (36832-72611)	446 (292-554)	2.6 (1.56-3.34)
Community + Linkages	55580 (36170-70467)	422 (274-524)	2.59 (1.59-3.27)
Facilities + Linkages	46454 (28023-60781)	348 (199-455)	2.17 (1.22-2.89)
Facilities + Linkages + Quality	40568 (21890-54971)	303 (156-407)	1.93 (0.91-2.62)
Comprehensive	37971 (20412-51376)	295 (147-393)	1.78 (0.84-2.48)

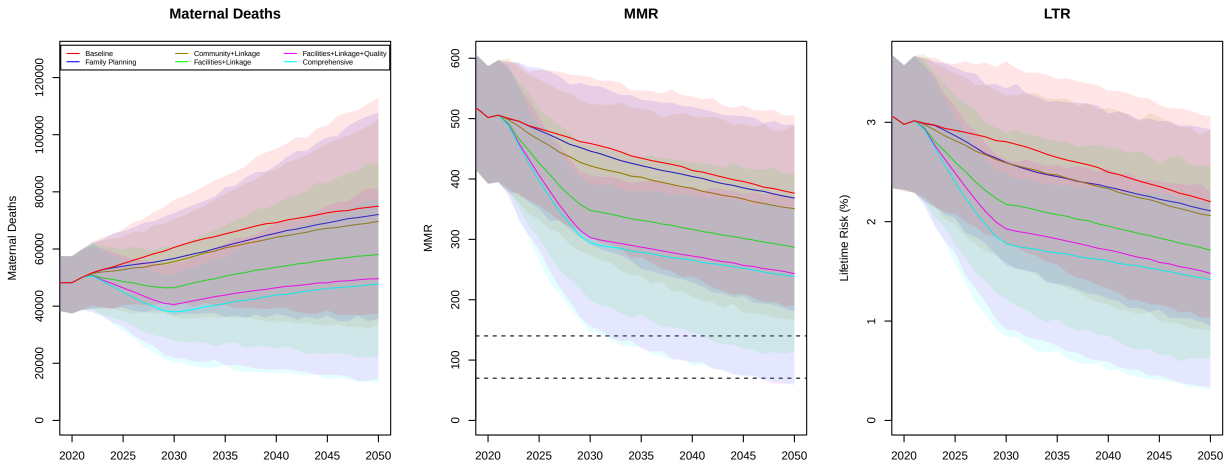

North Macedonia

ISO Code	Region	Area	Income Group
MKD	Southern Europe	Europe	Upper middle income

Projected Maternal Indicators in 2030 by Scenario

Scenario	Maternal Deaths	MMR	LTR
**Baseline**	10 (0-66)	28 (0-273)	0.04 (0-0.34)
**Family Planning Interventions**			
Contraception	9 (0-67)	24 (0-214)	0.03 (0-0.32)
Medical abortion	9 (0-66)	25 (0-238)	0.04 (0-0.33)
**Community-Based Interventions**			
ANC	9 (0-57)	27 (0-278)	0.04 (0-0.34)
SBA	9 (0-66)	27 (0-268)	0.04 (0-0.34)
**Facility-Based Interventions**			
Facility births	9 (0-56)	25 (0-259)	0.04 (0-0.34)
nonEmOC services	10 (0-66)	28 (0-273)	0.04 (0-0.34)
bEmOC services	10 (0-66)	28 (0-273)	0.04 (0-0.34)
cEmOC services	10 (0-68)	29 (0-268)	0.04 (0-0.34)
**System-Relevant Interventions**			
Quality of care	10 (0-66)	28 (0-273)	0.04 (0-0.34)
Referral	10 (0-67)	28 (0-274)	0.04 (0-0.34)
Transport	9 (0-65)	31 (0-275)	0.04 (0-0.35)
Targeted transfers	10 (0-66)	28 (0-273)	0.04 (0-0.34)
**Integrated Strategies**			
Family Planning	9 (0-62)	22 (0-222)	0.03 (0-0.32)
Community + Linkages	8 (0-65)	30 (0-275)	0.04 (0-0.35)
Facilities + Linkages	8 (0-43)	27 (0-275)	0.04 (0-0.34)
Facilities + Linkages + Quality	8 (0-43)	27 (0-275)	0.04 (0-0.34)
Comprehensive	7 (0-43)	16 (0-200)	0.02 (0-0.29)

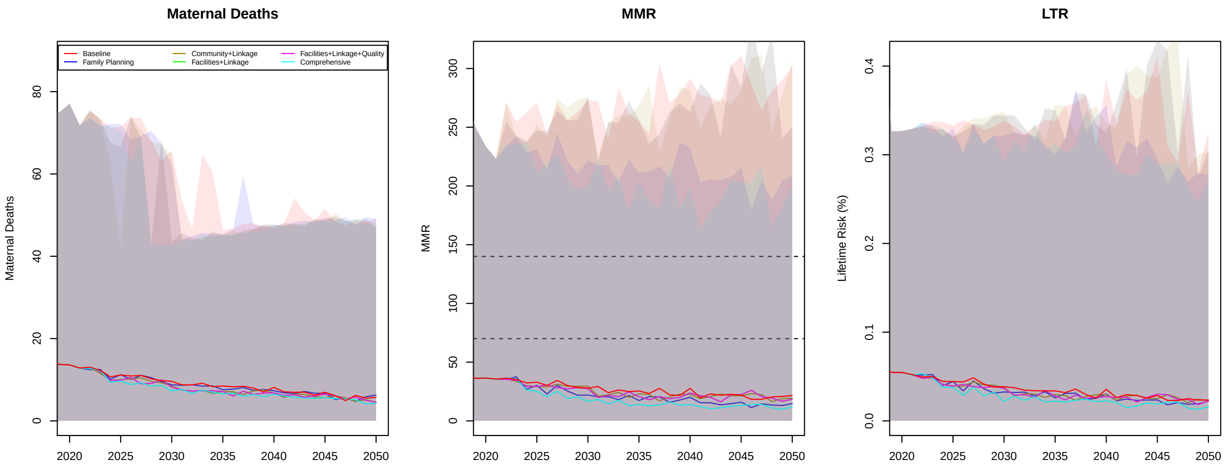

Norway

ISO Code	Region	Area	Income Group
NOR	Northern Europe	Europe	High income

Projected Maternal Indicators in 2030 by Scenario

Scenario	Maternal Deaths	MMR	LTR
**Baseline**	21 (0-54)	19 (0-61)	0.03 (0-0.1)
**Family Planning Interventions**			
Contraception	21 (0-53)	18 (0-63)	0.03 (0-0.09)
Medical abortion	21 (0-54)	19 (0-61)	0.03 (0-0.1)
**Community-Based Interventions**			
ANC	21 (0-52)	18 (0-59)	0.03 (0-0.09)
SBA	21 (0-53)	18 (0-60)	0.03 (0-0.09)
**Facility-Based Interventions**			
Facility births	20 (0-53)	18 (0-60)	0.03 (0-0.09)
nonEmOC services	21 (0-55)	19 (0-61)	0.03 (0-0.1)
bEmOC services	21 (0-53)	19 (0-61)	0.03 (0-0.1)
cEmOC services	21 (0-53)	18 (0-63)	0.03 (0-0.1)
**System-Relevant Interventions**			
Quality of care	21 (0-54)	18 (0-61)	0.03 (0-0.1)
Referral	21 (0-53)	18 (0-60)	0.03 (0-0.09)
Transport	20 (0-53)	18 (0-61)	0.03 (0-0.09)
Targeted transfers	21 (0-53)	18 (0-61)	0.03 (0-0.1)
**Integrated Strategies**			
Family Planning	21 (0-55)	19 (0-63)	0.03 (0-0.09)
Community + Linkages	20 (0-53)	18 (0-64)	0.03 (0-0.09)
Facilities + Linkages	20 (0-56)	17 (0-62)	0.03 (0-0.1)
Facilities + Linkages + Quality	20 (0-56)	17 (0-63)	0.03 (0-0.1)
Comprehensive	20 (0-53)	17 (0-57)	0.03 (0-0.09)

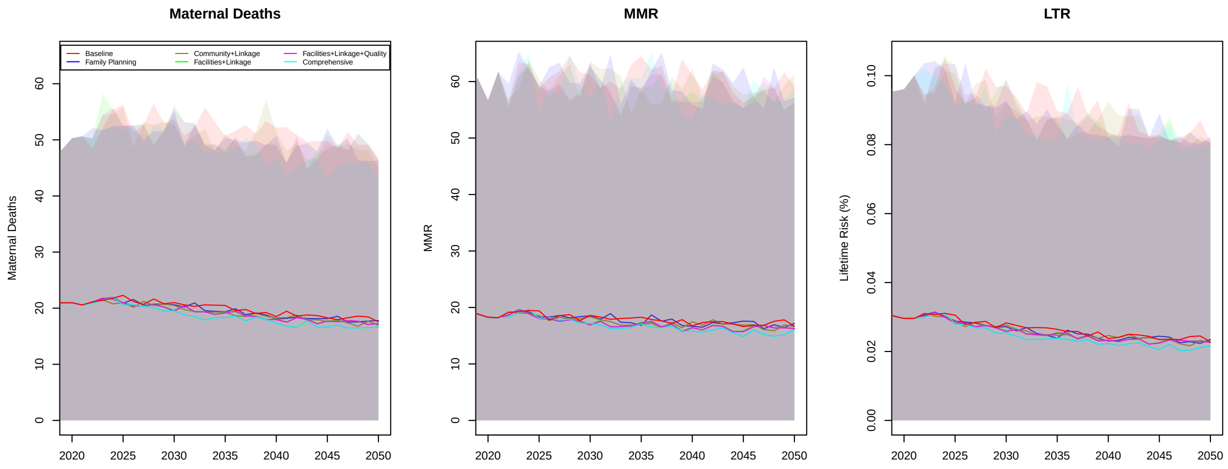

Oman

ISO Code	Region	Area	Income Group
OMN	Western Asia	Asia	High income

Projected Maternal Indicators in 2030 by Scenario

Scenario	Maternal Deaths	MMR	LTR
**Baseline**	24 (0-90)	16 (0-74)	0.04 (0-0.19)
**Family Planning Interventions**			
Contraception	20 (0-89)	15 (0-74)	0.04 (0-0.19)
Medical abortion	24 (0-89)	16 (0-70)	0.04 (0-0.18)
**Community-Based Interventions**			
ANC	23 (0-92)	16 (0-73)	0.04 (0-0.19)
SBA	24 (0-90)	17 (0-76)	0.04 (0-0.19)
**Facility-Based Interventions**			
Facility births	24 (0-93)	17 (0-79)	0.04 (0-0.2)
nonEmOC services	24 (0-90)	16 (0-77)	0.04 (0-0.2)
bEmOC services	24 (0-90)	16 (0-74)	0.04 (0-0.19)
cEmOC services	25 (0-91)	17 (0-74)	0.04 (0-0.19)
**System-Relevant Interventions**			
Quality of care	22 (0-89)	15 (0-75)	0.04 (0-0.19)
Referral	24 (0-90)	16 (0-77)	0.04 (0-0.19)
Transport	23 (0-90)	16 (0-69)	0.04 (0-0.18)
Targeted transfers	24 (0-90)	16 (0-70)	0.04 (0-0.19)
**Integrated Strategies**			
Family Planning	22 (0-86)	15 (0-72)	0.04 (0-0.18)
Community + Linkages	22 (0-90)	15 (0-72)	0.04 (0-0.18)
Facilities + Linkages	23 (0-90)	15 (0-75)	0.04 (0-0.19)
Facilities + Linkages + Quality	21 (0-82)	14 (0-64)	0.03 (0-0.18)
Comprehensive	18 (0-82)	12 (0-64)	0.03 (0-0.17)

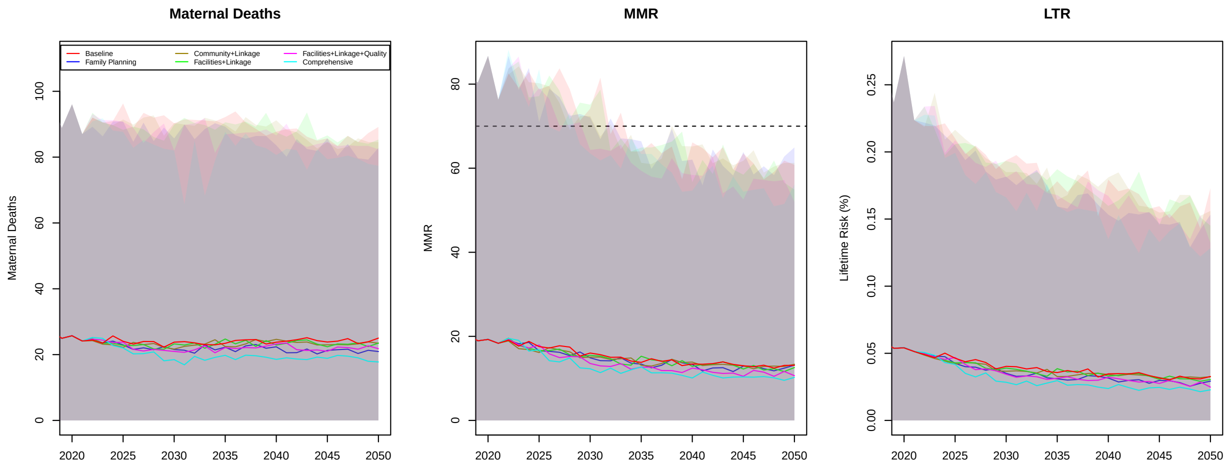

Pakistan

ISO Code	Region	Area	Income Group
PAK	Southern Asia	Asia	Lower middle income

Projected Maternal Indicators in 2030 by Scenario

Scenario	Maternal Deaths	MMR	LTR
**Baseline**	7462 (3942-14369)	82 (41-146)	0.23 (0.09-0.52)
**Family Planning Interventions**			
Contraception	6723 (3213-13345)	78 (39-145)	0.2 (0.08-0.5)
Medical abortion	7430 (3978-14008)	82 (40-147)	0.23 (0.1-0.52)
**Community-Based Interventions**			
ANC	7176 (3684-14160)	78 (39-138)	0.22 (0.09-0.5)
SBA	7328 (3975-13143)	80 (41-146)	0.23 (0.1-0.47)
**Facility-Based Interventions**			
Facility births	5574 (3194-8993)	52 (33-80)	0.15 (0.08-0.27)
nonEmOC services	7446 (3890-14704)	82 (40-148)	0.23 (0.09-0.52)
bEmOC services	7395 (3744-14516)	81 (40-146)	0.23 (0.09-0.52)
cEmOC services	7156 (3531-14137)	77 (34-145)	0.22 (0.08-0.51)
**System-Relevant Interventions**			
Quality of care	5886 (2807-11946)	58 (17-118)	0.17 (0.04-0.39)
Referral	7357 (3955-14263)	80 (40-143)	0.23 (0.1-0.52)
Transport	6762 (3677-12202)	74 (40-133)	0.21 (0.09-0.45)
Targeted transfers	7418 (3721-14406)	82 (40-146)	0.23 (0.09-0.51)
**Integrated Strategies**			
Family Planning	6750 (3226-13438)	77 (40-147)	0.2 (0.08-0.5)
Community + Linkages	6192 (3514-11005)	66 (38-115)	0.19 (0.09-0.39)
Facilities + Linkages	4856 (2816-8021)	44 (28-74)	0.13 (0.07-0.24)
Facilities + Linkages + Quality	3175 (1887-4982)	20 (10-37)	0.06 (0.03-0.12)
Comprehensive	2771 (1566-4279)	17 (9-27)	0.04 (0.02-0.08)

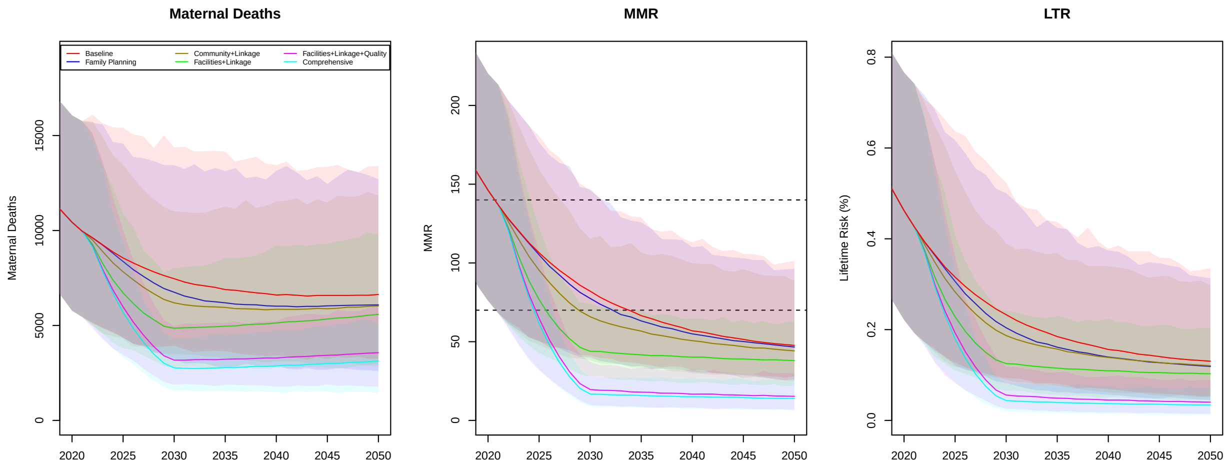

Palau

ISO Code	Region	Area	Income Group
PLW	Micronesia	Oceania	High income

Projected Maternal Indicators in 2030 by Scenario

Scenario	Maternal Deaths	MMR	LTR
**Baseline**	1 (0-4)	303 (0-924)	1.18 (0-3.66)
**Family Planning Interventions**			
Contraception	1 (0-4)	300 (0-944)	1.08 (0-3.46)
Medical abortion	1 (0-4)	301 (0-917)	1.18 (0-3.6)
**Community-Based Interventions**			
ANC	1 (0-4)	304 (0-924)	1.19 (0-3.65)
SBA	1 (0-4)	303 (0-924)	1.18 (0-3.66)
**Facility-Based Interventions**			
Facility births	1 (0-4)	303 (0-924)	1.18 (0-3.66)
nonEmOC services	1 (0-4)	303 (0-924)	1.18 (0-3.66)
bEmOC services	1 (0-4)	303 (0-924)	1.18 (0-3.66)
cEmOC services	1 (0-4)	300 (0-924)	1.17 (0-3.65)
**System-Relevant Interventions**			
Quality of care	1 (0-4)	297 (0-908)	1.16 (0-3.62)
Referral	1 (0-4)	303 (0-924)	1.18 (0-3.66)
Transport	1 (0-4)	307 (0-925)	1.2 (0-3.68)
Targeted transfers	1 (0-4)	301 (0-924)	1.17 (0-3.66)
**Integrated Strategies**			
Family Planning	1 (0-4)	298 (0-944)	1.07 (0-3.45)
Community + Linkages	1 (0-4)	306 (0-917)	1.2 (0-3.66)
Facilities + Linkages	1 (0-4)	302 (0-925)	1.18 (0-3.69)
Facilities + Linkages + Quality	1 (0-4)	289 (0-898)	1.12 (0-3.56)
Comprehensive	1 (0-3)	287 (0-874)	1.03 (0-3.07)

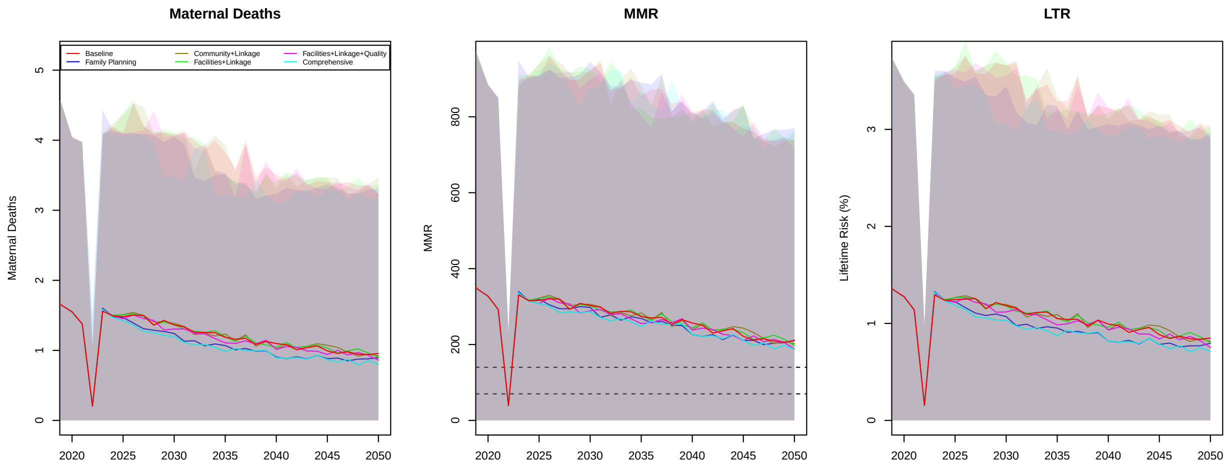

Panama

ISO Code	Region	Area	Income Group
PAN	Central America	Latin America and the Caribbean	High income

Projected Maternal Indicators in 2030 by Scenario

Scenario	Maternal Deaths	MMR	LTR
**Baseline**	69 (16-151)	64 (11-155)	0.16 (0.03-0.39)
**Family Planning Interventions**			
Contraception	63 (13-137)	60 (8-144)	0.15 (0.02-0.36)
Medical abortion	55 (10-127)	51 (4-130)	0.13 (0.01-0.33)
**Community-Based Interventions**			
ANC	67 (17-143)	63 (11-139)	0.16 (0.03-0.37)
SBA	69 (16-151)	64 (11-155)	0.16 (0.03-0.39)
**Facility-Based Interventions**			
Facility births	68 (16-151)	64 (11-155)	0.16 (0.03-0.39)
nonEmOC services	68 (16-148)	64 (11-153)	0.16 (0.03-0.39)
bEmOC services	68 (16-151)	64 (11-154)	0.16 (0.03-0.39)
cEmOC services	68 (17-143)	64 (10-149)	0.16 (0.03-0.38)
**System-Relevant Interventions**			
Quality of care	67 (17-148)	63 (11-148)	0.16 (0.03-0.39)
Referral	68 (16-149)	64 (11-155)	0.16 (0.03-0.39)
Transport	67 (17-147)	64 (11-150)	0.16 (0.03-0.39)
Targeted transfers	68 (17-148)	64 (11-155)	0.16 (0.03-0.39)
**Integrated Strategies**			
Family Planning	51 (10-123)	48 (4-120)	0.12 (0.01-0.32)
Community + Linkages	65 (17-150)	61 (10-147)	0.15 (0.03-0.39)
Facilities + Linkages	66 (17-142)	63 (13-150)	0.16 (0.03-0.37)
Facilities + Linkages + Quality	66 (15-142)	63 (11-150)	0.16 (0.02-0.38)
Comprehensive	50 (7-118)	47 (4-116)	0.12 (0.01-0.3)

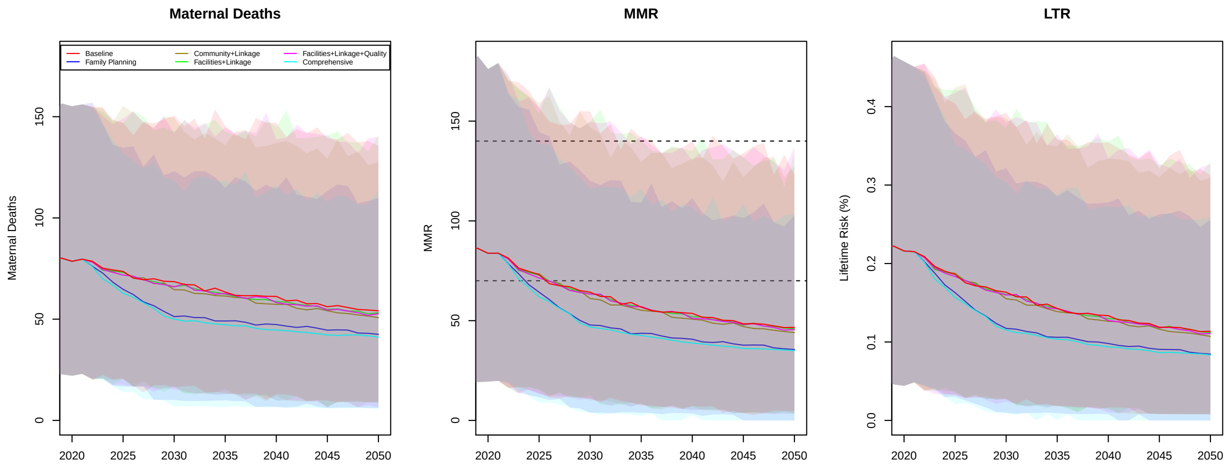

Papua New Guinea

ISO Code	Region	Area	Income Group
PNG	Melanesia	Oceania	Lower middle income

Projected Maternal Indicators in 2030 by Scenario

Scenario	Maternal Deaths	MMR	LTR
**Baseline**	787 (174-1755)	189 (40-414)	0.71 (0.13-1.66)
**Family Planning Interventions**			
Contraception	687 (167-1595)	182 (42-392)	0.62 (0.11-1.52)
Medical abortion	776 (167-1777)	186 (45-388)	0.7 (0.13-1.64)
**Community-Based Interventions**			
ANC	774 (151-1721)	189 (37-411)	0.71 (0.12-1.65)
SBA	787 (152-1765)	188 (38-403)	0.71 (0.11-1.67)
**Facility-Based Interventions**			
Facility births	763 (134-1771)	179 (39-375)	0.69 (0.1-1.67)
nonEmOC services	784 (164-1807)	189 (40-442)	0.71 (0.13-1.6)
bEmOC services	783 (174-1771)	189 (39-420)	0.7 (0.12-1.64)
cEmOC services	751 (160-1626)	181 (43-405)	0.68 (0.12-1.52)
**System-Relevant Interventions**			
Quality of care	475 (80-1146)	106 (13-297)	0.4 (0.05-0.99)
Referral	781 (186-1744)	190 (45-423)	0.71 (0.14-1.64)
Transport	761 (157-1746)	186 (33-370)	0.7 (0.1-1.62)
Targeted transfers	781 (160-1717)	187 (43-409)	0.71 (0.13-1.6)
**Integrated Strategies**			
Family Planning	676 (142-1534)	180 (38-402)	0.61 (0.1-1.44)
Community + Linkages	719 (154-1568)	175 (43-383)	0.66 (0.11-1.52)
Facilities + Linkages	676 (130-1557)	163 (38-343)	0.62 (0.11-1.56)
Facilities + Linkages + Quality	367 (43-889)	80 (0-209)	0.31 (0-0.83)
Comprehensive	299 (0-787)	70 (0-190)	0.24 (0-0.72)

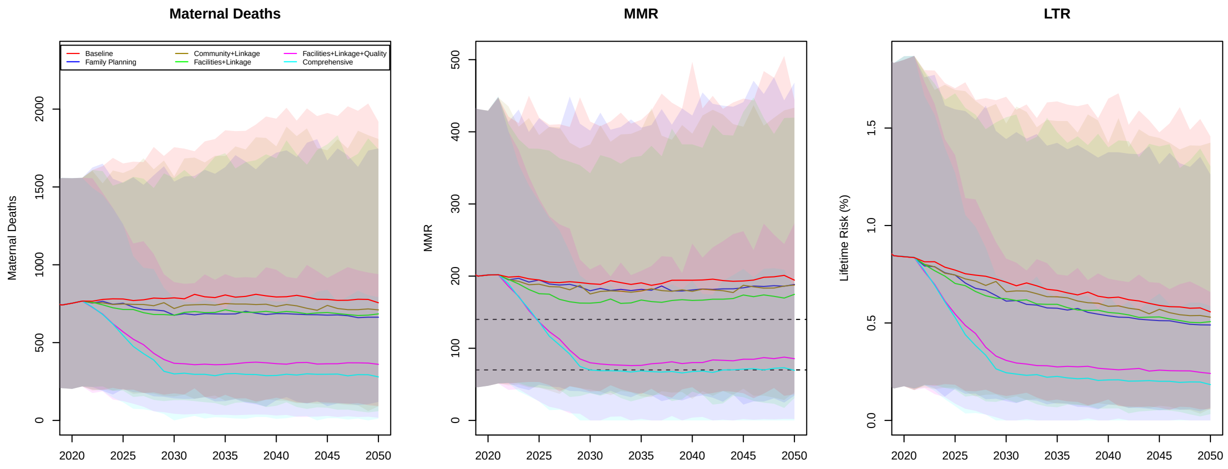

Paraguay

ISO Code	Region	Area	Income Group
PRY	South America	Latin America and the Caribbean	Upper middle income

Projected Maternal Indicators in 2030 by Scenario

Scenario	Maternal Deaths	MMR	LTR
**Baseline**	142 (17-364)	75 (0-197)	0.2 (0-0.58)
**Family Planning Interventions**			
Contraception	127 (10-342)	69 (0-186)	0.18 (0-0.52)
Medical abortion	143 (17-364)	75 (0-197)	0.2 (0-0.58)
**Community-Based Interventions**			
ANC	140 (17-347)	74 (0-185)	0.2 (0-0.55)
SBA	130 (11-335)	68 (0-180)	0.18 (0-0.54)
**Facility-Based Interventions**			
Facility births	101 (0-302)	49 (0-160)	0.13 (0-0.48)
nonEmOC services	144 (19-352)	75 (0-200)	0.2 (0-0.57)
bEmOC services	143 (17-367)	75 (0-197)	0.2 (0-0.58)
cEmOC services	141 (17-344)	75 (0-190)	0.2 (0-0.56)
**System-Relevant Interventions**			
Quality of care	145 (16-369)	76 (0-196)	0.21 (0-0.59)
Referral	147 (16-363)	78 (0-205)	0.21 (0-0.56)
Transport	129 (10-351)	69 (0-176)	0.19 (0-0.54)
Targeted transfers	142 (17-364)	75 (0-197)	0.2 (0-0.58)
**Integrated Strategies**			
Family Planning	128 (10-342)	69 (0-186)	0.18 (0-0.52)
Community + Linkages	118 (5-324)	61 (0-170)	0.17 (0-0.5)
Facilities + Linkages	96 (0-300)	47 (0-157)	0.13 (0-0.47)
Facilities + Linkages + Quality	95 (0-287)	46 (0-146)	0.13 (0-0.45)
Comprehensive	88 (0-277)	45 (0-164)	0.12 (0-0.43)

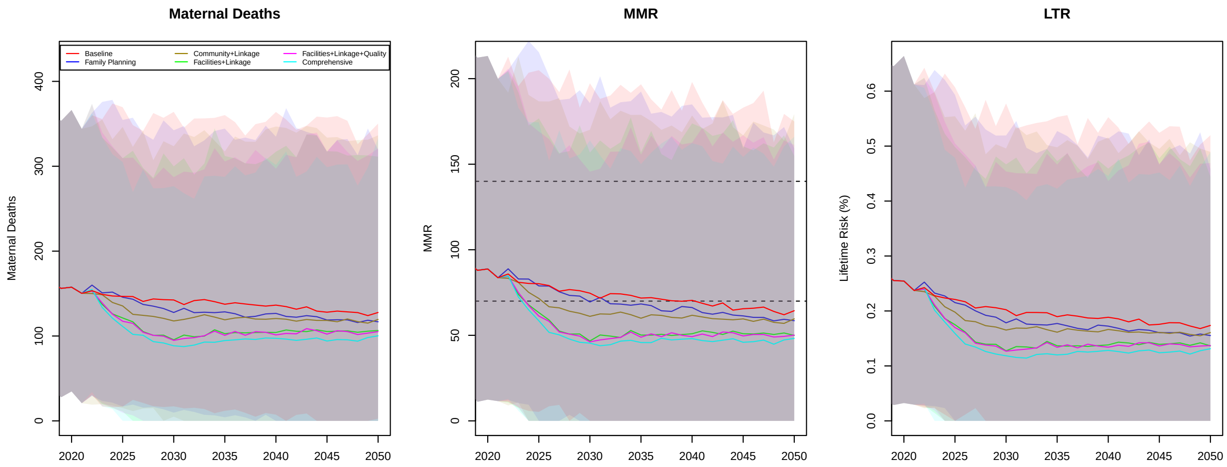

Peru

ISO Code	Region	Area	Income Group
PER	South America	Latin America and the Caribbean	Upper middle income

Projected Maternal Indicators in 2030 by Scenario

Scenario	Maternal Deaths	MMR	LTR
**Baseline**	371 (110-815)	52 (11-120)	0.11 (0.02-0.26)
**Family Planning Interventions**			
Contraception	347 (111-704)	50 (10-112)	0.1 (0.02-0.22)
Medical abortion	371 (110-815)	52 (11-120)	0.11 (0.02-0.26)
**Community-Based Interventions**			
ANC	369 (93-808)	52 (11-123)	0.1 (0.02-0.26)
SBA	345 (91-761)	48 (8-116)	0.1 (0.01-0.24)
**Facility-Based Interventions**			
Facility births	266 (55-644)	34 (4-103)	0.07 (0.01-0.2)
nonEmOC services	373 (102-791)	53 (11-122)	0.11 (0.02-0.25)
bEmOC services	369 (108-791)	52 (11-119)	0.1 (0.02-0.25)
cEmOC services	373 (106-822)	52 (10-122)	0.11 (0.02-0.25)
**System-Relevant Interventions**			
Quality of care	370 (110-798)	52 (12-125)	0.11 (0.02-0.26)
Referral	370 (96-843)	52 (12-122)	0.1 (0.02-0.27)
Transport	338 (97-752)	48 (9-115)	0.1 (0.02-0.24)
Targeted transfers	363 (104-773)	51 (11-120)	0.1 (0.02-0.25)
**Integrated Strategies**			
Family Planning	347 (109-710)	50 (10-113)	0.1 (0.02-0.22)
Community + Linkages	309 (82-703)	43 (7-105)	0.09 (0.01-0.21)
Facilities + Linkages	256 (55-664)	33 (3-105)	0.07 (0.01-0.2)
Facilities + Linkages + Quality	254 (53-675)	33 (2-111)	0.07 (0-0.22)
Comprehensive	236 (50-578)	31 (2-89)	0.06 (0-0.18)

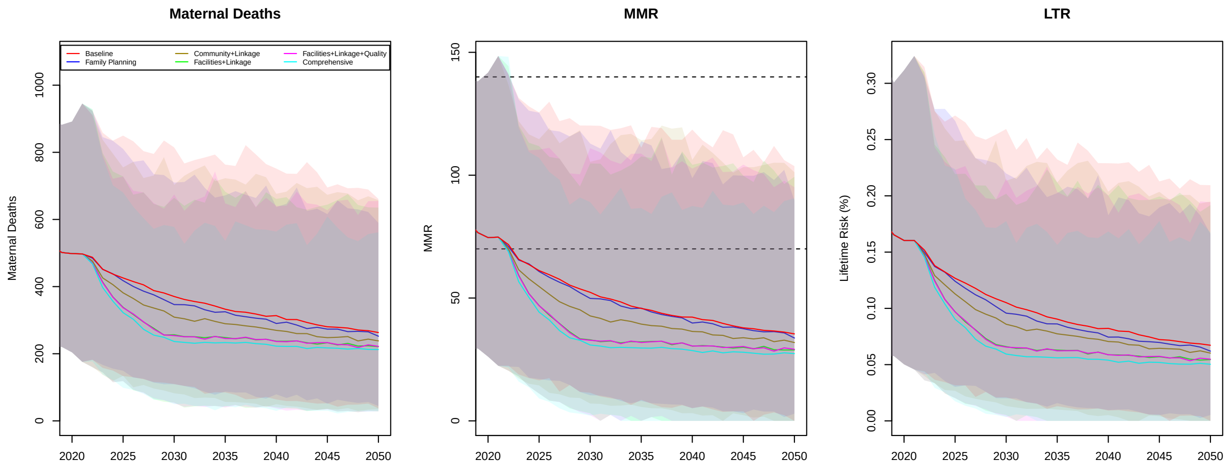

Philippines

ISO Code	Region	Area	Income Group
PHL	South-Eastern Asia	Asia	Lower middle income

Projected Maternal Indicators in 2030 by Scenario

Scenario	Maternal Deaths	MMR	LTR
**Baseline**	4171 (1368-8972)	128 (36-284)	0.34 (0.1-0.77)
**Family Planning Interventions**			
Contraception	4044 (1229-9027)	127 (38-271)	0.34 (0.09-0.78)
Medical abortion	4162 (1205-8968)	128 (37-283)	0.34 (0.1-0.79)
**Community-Based Interventions**			
ANC	4185 (1289-8986)	129 (37-284)	0.35 (0.09-0.76)
SBA	3374 (1215-6217)	101 (33-189)	0.27 (0.09-0.52)
**Facility-Based Interventions**			
Facility births	1778 (608-3321)	44 (13-82)	0.12 (0.03-0.24)
nonEmOC services	4134 (1215-9276)	127 (39-284)	0.34 (0.1-0.79)
bEmOC services	4181 (1344-9141)	129 (38-278)	0.34 (0.1-0.79)
cEmOC services	4080 (1282-9126)	125 (35-281)	0.33 (0.09-0.77)
**System-Relevant Interventions**			
Quality of care	4172 (1312-9153)	129 (39-283)	0.35 (0.1-0.79)
Referral	4157 (1248-9295)	128 (38-286)	0.34 (0.1-0.79)
Transport	3463 (1174-6560)	107 (36-200)	0.29 (0.09-0.56)
Targeted transfers	4167 (1368-8924)	129 (38-281)	0.34 (0.1-0.77)
**Integrated Strategies**			
Family Planning	4023 (1268-8690)	126 (37-268)	0.33 (0.09-0.74)
Community + Linkages	3051 (1149-5583)	92 (32-174)	0.25 (0.08-0.47)
Facilities + Linkages	1532 (483-2853)	38 (11-74)	0.11 (0.02-0.22)
Facilities + Linkages + Quality	1476 (449-2772)	37 (11-74)	0.1 (0.02-0.22)
Comprehensive	1365 (397-2575)	34 (8-69)	0.09 (0.02-0.2)

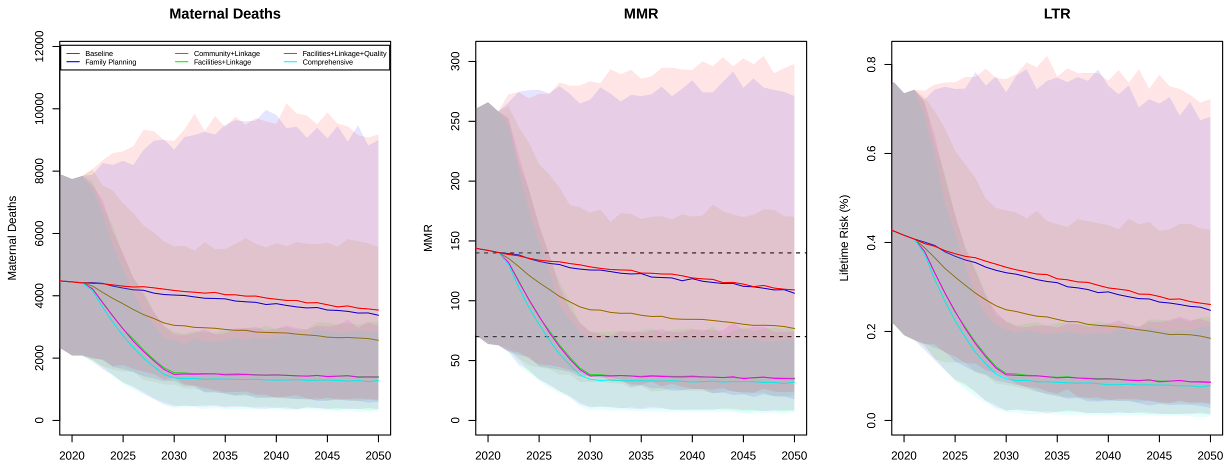

Poland

ISO Code	Region	Area	Income Group
POL	Eastern Europe	Europe	High income

Projected Maternal Indicators in 2030 by Scenario

Scenario	Maternal Deaths	MMR	LTR
**Baseline**	78 (0-180)	11 (0-37)	0.01 (0-0.05)
**Family Planning Interventions**			
Contraception	77 (0-177)	11 (0-35)	0.01 (0-0.05)
Medical abortion	77 (0-181)	11 (0-36)	0.01 (0-0.05)
**Community-Based Interventions**			
ANC	76 (0-180)	11 (0-35)	0.01 (0-0.05)
SBA	77 (0-180)	11 (0-36)	0.01 (0-0.05)
**Facility-Based Interventions**			
Facility births	78 (0-180)	11 (0-37)	0.01 (0-0.05)
nonEmOC services	78 (0-180)	11 (0-37)	0.01 (0-0.05)
bEmOC services	78 (0-181)	11 (0-37)	0.01 (0-0.05)
cEmOC services	76 (0-174)	11 (0-34)	0.01 (0-0.05)
**System-Relevant Interventions**			
Quality of care	78 (0-180)	11 (0-37)	0.01 (0-0.05)
Referral	78 (0-180)	11 (0-37)	0.01 (0-0.05)
Transport	75 (0-174)	11 (0-35)	0.01 (0-0.04)
Targeted transfers	78 (0-180)	11 (0-37)	0.01 (0-0.05)
**Integrated Strategies**			
Family Planning	78 (0-169)	12 (0-36)	0.01 (0-0.05)
Community + Linkages	73 (0-166)	11 (0-33)	0.01 (0-0.05)
Facilities + Linkages	72 (0-163)	11 (0-36)	0.01 (0-0.05)
Facilities + Linkages + Quality	72 (0-163)	11 (0-36)	0.01 (0-0.05)
Comprehensive	73 (0-176)	11 (0-34)	0.01 (0-0.04)

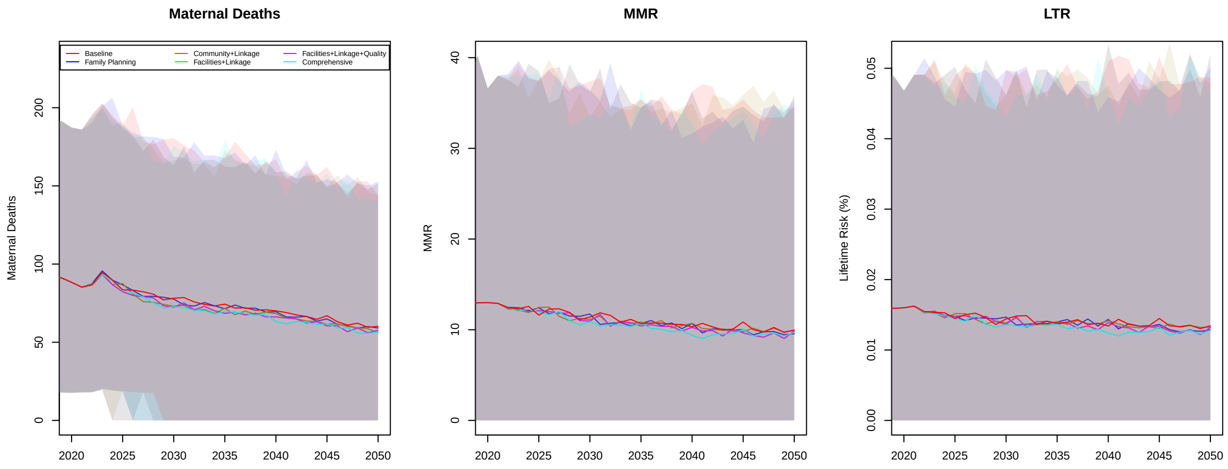

Portugal

ISO Code	Region	Area	Income Group
PRT	Southern Europe	Europe	High income

Projected Maternal Indicators in 2030 by Scenario

Scenario	Maternal Deaths	MMR	LTR
**Baseline**	42 (7-98)	36 (0-109)	0.05 (0-0.15)
**Family Planning Interventions**			
Contraception	39 (7-87)	33 (0-91)	0.04 (0-0.11)
Medical abortion	41 (6-88)	34 (0-97)	0.05 (0-0.12)
**Community-Based Interventions**			
ANC	41 (7-92)	34 (0-103)	0.04 (0-0.13)
SBA	41 (7-93)	35 (0-101)	0.05 (0-0.13)
**Facility-Based Interventions**			
Facility births	41 (6-96)	34 (0-103)	0.04 (0-0.13)
nonEmOC services	42 (7-98)	36 (0-112)	0.05 (0-0.15)
bEmOC services	42 (7-98)	36 (0-109)	0.05 (0-0.14)
cEmOC services	41 (7-94)	35 (0-109)	0.05 (0-0.13)
**System-Relevant Interventions**			
Quality of care	41 (7-98)	35 (0-109)	0.05 (0-0.14)
Referral	41 (8-97)	35 (0-107)	0.05 (0-0.14)
Transport	40 (7-89)	35 (0-102)	0.05 (0-0.13)
Targeted transfers	41 (8-96)	35 (0-110)	0.05 (0-0.14)
**Integrated Strategies**			
Family Planning	39 (7-87)	32 (0-89)	0.04 (0-0.11)
Community + Linkages	39 (6-92)	34 (0-99)	0.04 (0-0.13)
Facilities + Linkages	39 (6-92)	34 (0-100)	0.04 (0-0.13)
Facilities + Linkages + Quality	40 (7-91)	34 (0-101)	0.04 (0-0.13)
Comprehensive	35 (3-80)	29 (0-83)	0.04 (0-0.11)

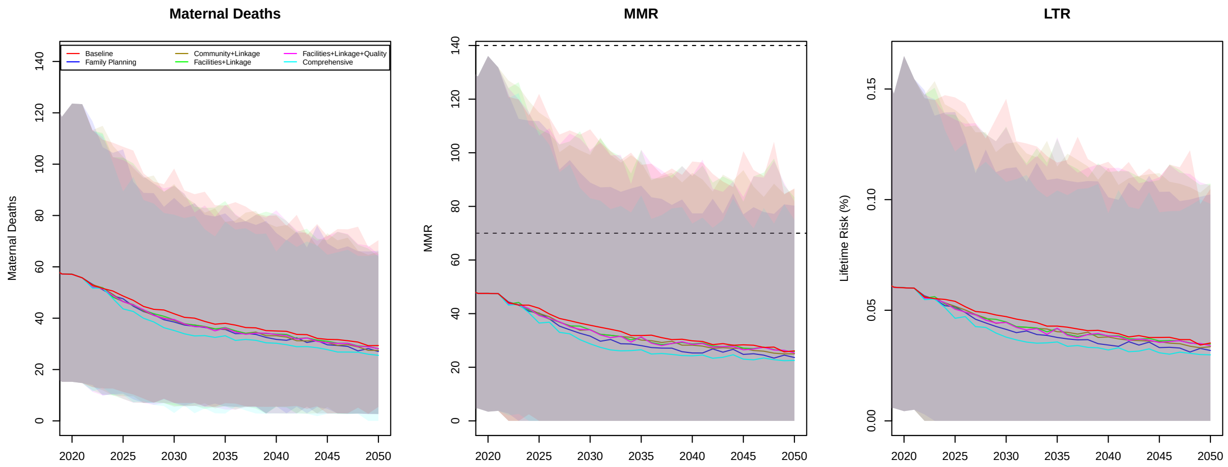

Puerto Rico

ISO Code	Region	Area	Income Group
PRI	Caribbean	Latin America and the Caribbean	High income

Projected Maternal Indicators in 2030 by Scenario

Scenario	Maternal Deaths	MMR	LTR
**Baseline**	5 (0-16)	12 (0-57)	0.01 (0-0.06)
**Family Planning Interventions**			
Contraception	5 (0-15)	11 (0-57)	0.01 (0-0.06)
Medical abortion	5 (0-15)	11 (0-54)	0.01 (0-0.06)
**Community-Based Interventions**			
ANC	5 (0-16)	12 (0-55)	0.01 (0-0.06)
SBA	5 (0-16)	12 (0-57)	0.01 (0-0.06)
**Facility-Based Interventions**			
Facility births	5 (0-16)	12 (0-58)	0.01 (0-0.06)
nonEmOC services	5 (0-16)	12 (0-57)	0.01 (0-0.06)
bEmOC services	5 (0-16)	12 (0-57)	0.01 (0-0.06)
cEmOC services	5 (0-16)	12 (0-56)	0.01 (0-0.06)
**System-Relevant Interventions**			
Quality of care	5 (0-16)	12 (0-56)	0.01 (0-0.06)
Referral	5 (0-16)	12 (0-57)	0.01 (0-0.06)
Transport	5 (0-16)	12 (0-58)	0.01 (0-0.06)
Targeted transfers	5 (0-16)	12 (0-57)	0.01 (0-0.06)
**Integrated Strategies**			
Family Planning	4 (0-15)	10 (0-53)	0.01 (0-0.05)
Community + Linkages	5 (0-15)	12 (0-57)	0.01 (0-0.06)
Facilities + Linkages	5 (0-16)	12 (0-57)	0.01 (0-0.06)
Facilities + Linkages + Quality	5 (0-16)	12 (0-57)	0.01 (0-0.06)
Comprehensive	4 (0-14)	10 (0-54)	0.01 (0-0.05)

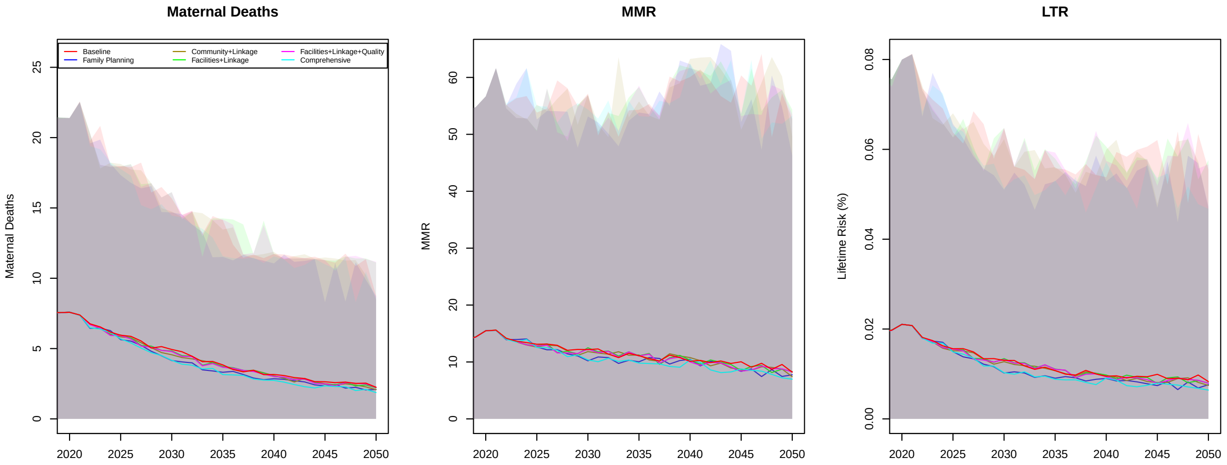

Qatar

ISO Code	Region	Area	Income Group
QAT	Western Asia	Asia	High income

Projected Maternal Indicators in 2030 by Scenario

Scenario	Maternal Deaths	MMR	LTR
**Baseline**	11 (0-40)	19 (0-96)	0.04 (0-0.22)
**Family Planning Interventions**			
Contraception	9 (0-39)	16 (0-82)	0.03 (0-0.2)
Medical abortion	10 (0-39)	19 (0-87)	0.04 (0-0.21)
**Community-Based Interventions**			
ANC	11 (0-39)	19 (0-99)	0.04 (0-0.21)
SBA	11 (0-40)	19 (0-96)	0.04 (0-0.22)
**Facility-Based Interventions**			
Facility births	11 (0-40)	19 (0-94)	0.04 (0-0.23)
nonEmOC services	11 (0-40)	19 (0-96)	0.04 (0-0.22)
bEmOC services	11 (0-40)	19 (0-96)	0.04 (0-0.22)
cEmOC services	11 (0-43)	20 (0-111)	0.04 (0-0.23)
**System-Relevant Interventions**			
Quality of care	11 (0-40)	20 (0-97)	0.04 (0-0.23)
Referral	11 (0-40)	19 (0-96)	0.04 (0-0.22)
Transport	11 (0-40)	20 (0-102)	0.04 (0-0.22)
Targeted transfers	11 (0-40)	19 (0-96)	0.04 (0-0.22)
**Integrated Strategies**			
Family Planning	9 (0-39)	15 (0-80)	0.03 (0-0.18)
Community + Linkages	11 (0-41)	20 (0-105)	0.04 (0-0.24)
Facilities + Linkages	10 (0-42)	20 (0-102)	0.04 (0-0.23)
Facilities + Linkages + Quality	10 (0-41)	20 (0-103)	0.04 (0-0.26)
Comprehensive	8 (0-39)	13 (0-74)	0.03 (0-0.16)

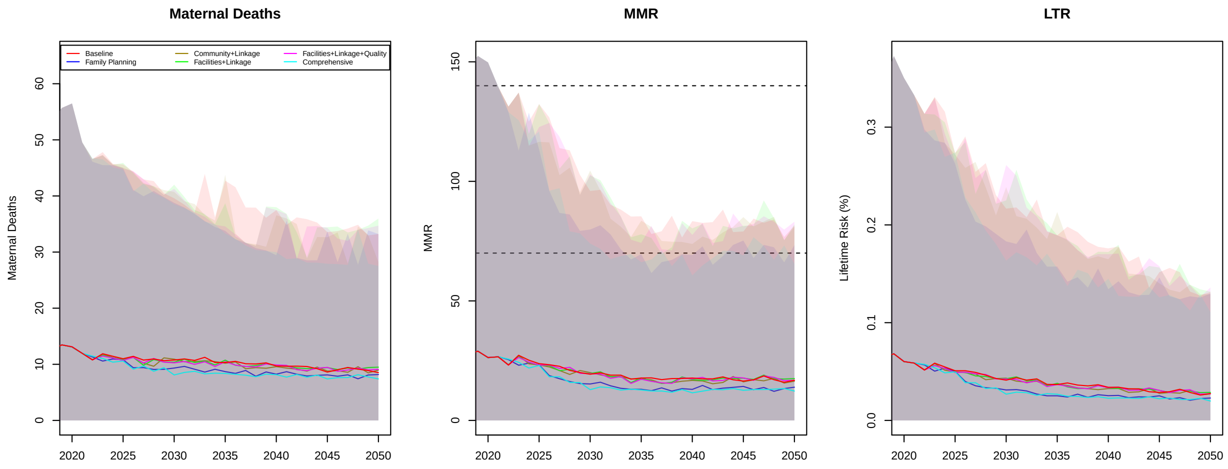

Republic of Korea

ISO Code	Region	Area	Income Group
KOR	Eastern Asia	Asia	High income

Projected Maternal Indicators in 2030 by Scenario

Scenario	Maternal Deaths	MMR	LTR
**Baseline**	67 (11-177)	10 (0-30)	0.01 (0-0.04)
**Family Planning Interventions**			
Contraception	66 (13-176)	9 (0-28)	0.01 (0-0.04)
Medical abortion	67 (12-169)	10 (0-30)	0.01 (0-0.04)
**Community-Based Interventions**			
ANC	66 (12-169)	10 (0-27)	0.01 (0-0.04)
SBA	67 (11-177)	10 (0-31)	0.01 (0-0.04)
**Facility-Based Interventions**			
Facility births	68 (8-180)	10 (0-30)	0.01 (0-0.04)
nonEmOC services	68 (11-176)	10 (0-30)	0.01 (0-0.04)
bEmOC services	68 (11-177)	10 (0-30)	0.01 (0-0.04)
cEmOC services	66 (13-177)	10 (0-30)	0.01 (0-0.04)
**System-Relevant Interventions**			
Quality of care	65 (8-157)	9 (0-27)	0.01 (0-0.04)
Referral	68 (13-177)	10 (0-30)	0.01 (0-0.04)
Transport	65 (8-166)	10 (0-30)	0.01 (0-0.04)
Targeted transfers	69 (12-174)	10 (0-30)	0.01 (0-0.04)
**Integrated Strategies**			
Family Planning	66 (12-174)	9 (0-28)	0.01 (0-0.04)
Community + Linkages	65 (8-176)	10 (0-27)	0.01 (0-0.04)
Facilities + Linkages	63 (8-171)	9 (0-29)	0.01 (0-0.04)
Facilities + Linkages + Quality	61 (10-160)	9 (0-27)	0.01 (0-0.04)
Comprehensive	61 (11-159)	8 (0-25)	0.01 (0-0.04)

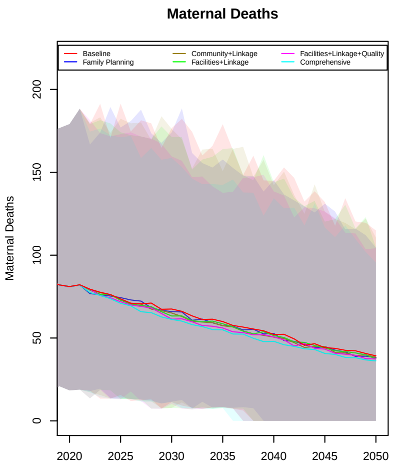

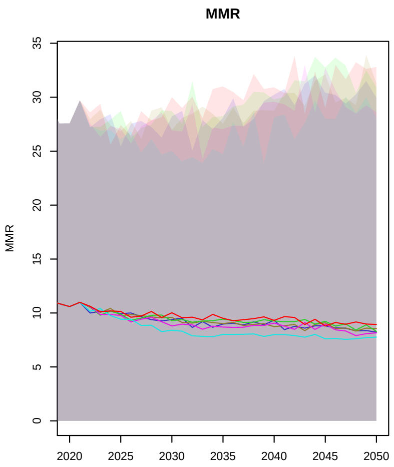

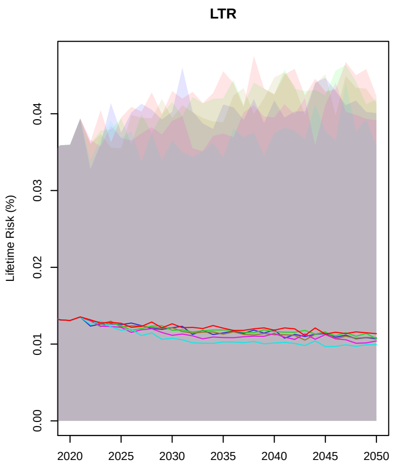

Republic of Moldova

ISO Code	Region	Area	Income Group
MDA	Eastern Europe	Europe	Lower middle income

Projected Maternal Indicators in 2030 by Scenario

Scenario	Maternal Deaths	MMR	LTR
**Baseline**	37 (0-108)	79 (0-315)	0.09 (0-0.39)
**Family Planning Interventions**			
Contraception	54 (0-147)	66 (0-239)	0.13 (0-0.46)
Medical abortion	37 (0-107)	78 (0-310)	0.09 (0-0.39)
**Community-Based Interventions**			
ANC	36 (0-109)	74 (0-283)	0.09 (0-0.37)
SBA	37 (0-107)	78 (0-319)	0.09 (0-0.38)
**Facility-Based Interventions**			
Facility births	38 (0-108)	80 (0-323)	0.1 (0-0.39)
nonEmOC services	37 (0-109)	79 (0-315)	0.1 (0-0.39)
bEmOC services	37 (0-112)	79 (0-315)	0.1 (0-0.39)
cEmOC services	37 (0-103)	75 (0-289)	0.09 (0-0.35)
**System-Relevant Interventions**			
Quality of care	32 (0-98)	60 (0-263)	0.07 (0-0.32)
Referral	37 (0-109)	78 (0-315)	0.09 (0-0.39)
Transport	35 (0-111)	81 (0-313)	0.1 (0-0.41)
Targeted transfers	37 (0-108)	79 (0-312)	0.09 (0-0.39)
**Integrated Strategies**			
Family Planning	54 (0-147)	66 (0-243)	0.13 (0-0.47)
Community + Linkages	34 (0-110)	77 (0-329)	0.09 (0-0.41)
Facilities + Linkages	33 (0-106)	70 (0-291)	0.09 (0-0.37)
Facilities + Linkages + Quality	26 (0-86)	45 (0-227)	0.05 (0-0.29)
Comprehensive	36 (0-110)	39 (0-179)	0.08 (0-0.36)

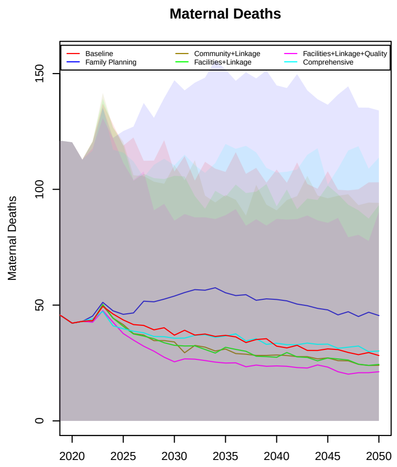

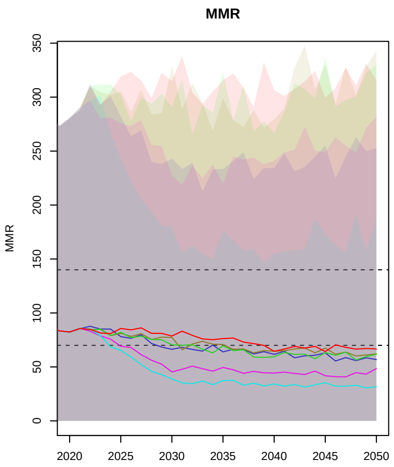

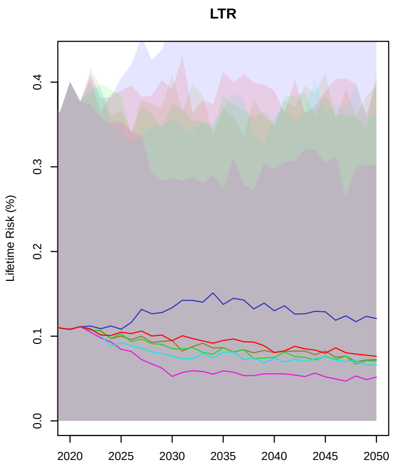

Romania

ISO Code	Region	Area	Income Group
ROU	Eastern Europe	Europe	Upper middle income

Projected Maternal Indicators in 2030 by Scenario

Scenario	Maternal Deaths	MMR	LTR
**Baseline**	104 (36-205)	41 (6-99)	0.06 (0.01-0.13)
**Family Planning Interventions**			
Contraception	96 (33-196)	41 (6-96)	0.05 (0.01-0.13)
Medical abortion	104 (36-206)	41 (6-101)	0.06 (0.01-0.13)
**Community-Based Interventions**			
ANC	104 (34-211)	41 (6-97)	0.06 (0.01-0.14)
SBA	104 (36-204)	42 (6-100)	0.06 (0.01-0.13)
**Facility-Based Interventions**			
Facility births	105 (37-205)	42 (7-100)	0.06 (0.01-0.13)
nonEmOC services	104 (37-205)	41 (6-98)	0.06 (0.01-0.13)
bEmOC services	103 (35-197)	40 (6-97)	0.05 (0.01-0.13)
cEmOC services	104 (36-201)	41 (6-99)	0.06 (0.01-0.13)
**System-Relevant Interventions**			
Quality of care	103 (35-200)	41 (6-97)	0.06 (0.01-0.13)
Referral	104 (36-201)	41 (6-98)	0.06 (0.01-0.13)
Transport	96 (31-192)	39 (6-94)	0.05 (0.01-0.13)
Targeted transfers	103 (37-198)	41 (6-97)	0.05 (0.01-0.13)
**Integrated Strategies**			
Family Planning	96 (36-191)	40 (7-95)	0.05 (0.01-0.12)
Community + Linkages	96 (32-188)	39 (6-92)	0.05 (0.01-0.13)
Facilities + Linkages	96 (34-187)	38 (6-94)	0.05 (0.01-0.12)
Facilities + Linkages + Quality	96 (34-187)	38 (6-94)	0.05 (0.01-0.12)
Comprehensive	85 (27-171)	36 (4-88)	0.05 (0.01-0.12)

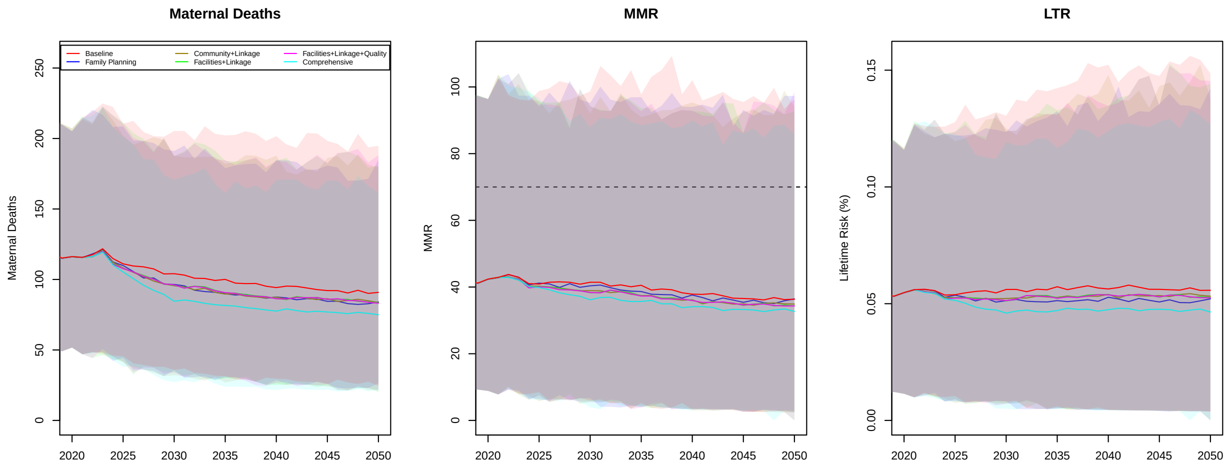

Russian Federation

ISO Code	Region	Area	Income Group
RUS	Eastern Europe	Europe	Upper middle income

Projected Maternal Indicators in 2030 by Scenario

Scenario	Maternal Deaths	MMR	LTR
**Baseline**	1168 (543-2020)	61 (22-115)	0.09 (0.03-0.18)
**Family Planning Interventions**			
Contraception	1125 (458-2094)	60 (21-119)	0.09 (0.03-0.18)
Medical abortion	1146 (540-1991)	59 (22-113)	0.09 (0.03-0.17)
**Community-Based Interventions**			
ANC	1141 (499-1979)	59 (20-111)	0.09 (0.03-0.17)
SBA	1173 (541-2040)	61 (22-117)	0.09 (0.03-0.18)
**Facility-Based Interventions**			
Facility births	1169 (543-2019)	61 (22-115)	0.09 (0.03-0.18)
nonEmOC services	1168 (543-2020)	61 (22-115)	0.09 (0.03-0.18)
bEmOC services	1160 (541-1988)	60 (22-115)	0.09 (0.03-0.17)
cEmOC services	1149 (501-2020)	59 (20-112)	0.09 (0.03-0.18)
**System-Relevant Interventions**			
Quality of care	1099 (462-2009)	56 (18-114)	0.08 (0.03-0.17)
Referral	1171 (543-2023)	61 (22-115)	0.09 (0.03-0.18)
Transport	1059 (501-1909)	58 (22-110)	0.09 (0.03-0.17)
Targeted transfers	1169 (543-2013)	61 (22-117)	0.09 (0.03-0.18)
**Integrated Strategies**			
Family Planning	1104 (517-1965)	59 (23-115)	0.09 (0.03-0.17)
Community + Linkages	1018 (452-1769)	55 (20-99)	0.08 (0.03-0.16)
Facilities + Linkages	1026 (493-1793)	55 (22-106)	0.08 (0.03-0.17)
Facilities + Linkages + Quality	956 (404-1751)	51 (17-99)	0.08 (0.02-0.16)
Comprehensive	865 (320-1610)	47 (13-94)	0.07 (0.02-0.14)

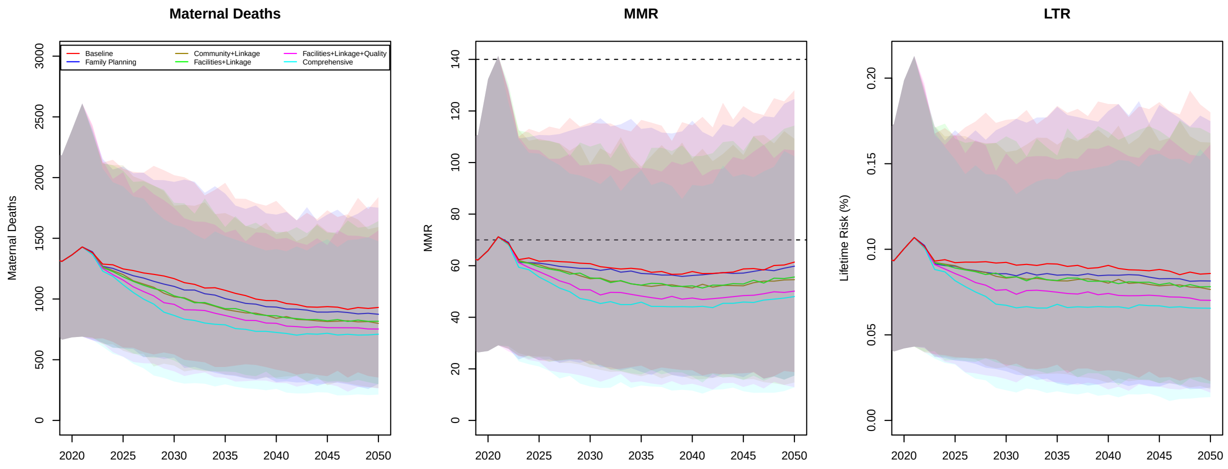

Rwanda

ISO Code	Region	Area	Income Group
RWA	Eastern Africa	Africa	Low income

Projected Maternal Indicators in 2030 by Scenario

Scenario	Maternal Deaths	MMR	LTR
**Baseline**	901 (215-2047)	136 (14-348)	0.53 (0.04-1.39)
**Family Planning Interventions**			
Contraception	823 (214-1876)	127 (17-341)	0.47 (0.06-1.21)
Medical abortion	856 (194-1910)	126 (14-328)	0.5 (0.05-1.3)
**Community-Based Interventions**			
ANC	907 (210-2135)	136 (19-360)	0.53 (0.07-1.37)
SBA	901 (230-2228)	137 (16-361)	0.53 (0.06-1.48)
**Facility-Based Interventions**			
Facility births	918 (228-2053)	139 (15-359)	0.55 (0.06-1.36)
nonEmOC services	905 (228-2007)	136 (15-353)	0.53 (0.06-1.37)
bEmOC services	908 (209-2179)	137 (16-362)	0.54 (0.06-1.46)
cEmOC services	891 (203-2111)	134 (14-357)	0.52 (0.06-1.44)
**System-Relevant Interventions**			
Quality of care	578 (93-1385)	74 (0-226)	0.3 (0-1)
Referral	894 (221-1997)	135 (20-359)	0.53 (0.07-1.39)
Transport	874 (195-2100)	137 (15-368)	0.54 (0.05-1.44)
Targeted transfers	917 (208-2143)	138 (19-352)	0.54 (0.06-1.5)
**Integrated Strategies**			
Family Planning	804 (192-1776)	122 (15-322)	0.45 (0.05-1.16)
Community + Linkages	838 (199-1954)	130 (15-347)	0.5 (0.06-1.35)
Facilities + Linkages	839 (142-2080)	130 (15-348)	0.51 (0.06-1.4)
Facilities + Linkages + Quality	513 (63-1297)	68 (0-223)	0.27 (0-0.89)
Comprehensive	398 (0-1200)	49 (0-201)	0.19 (0-0.85)

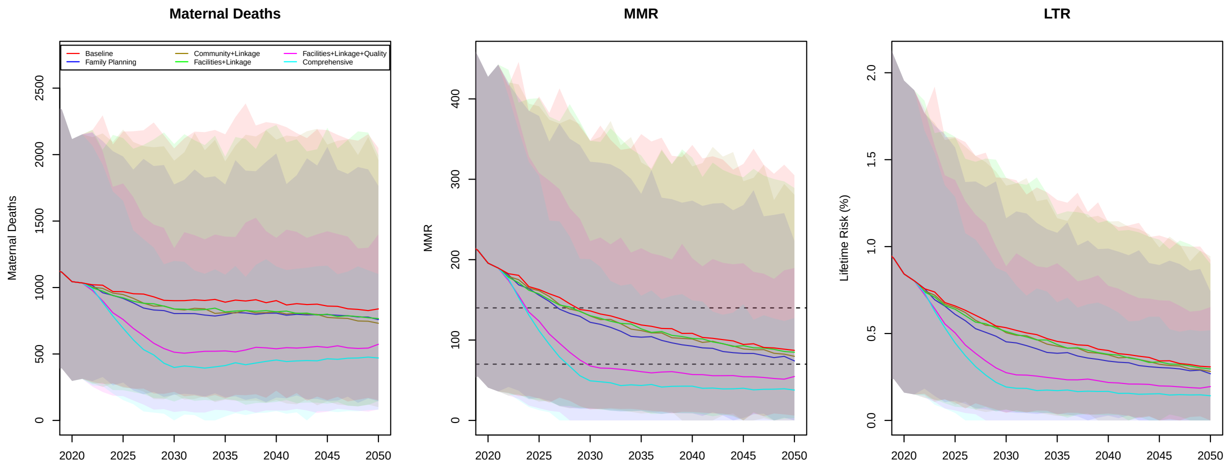

Saint Kitts and Nevis

ISO Code	Region	Area	Income Group
KNA	Caribbean	Latin America and the Caribbean	High income

Projected Maternal Indicators in 2030 by Scenario

Scenario	Maternal Deaths	MMR	LTR
**Baseline**	1 (0-3)	70 (0-319)	0.15 (0-0.71)
**Family Planning Interventions**			
Contraception	1 (0-3)	63 (0-324)	0.13 (0-0.58)
Medical abortion	1 (0-3)	64 (0-334)	0.14 (0-0.73)
**Community-Based Interventions**			
ANC	1 (0-3)	69 (0-325)	0.15 (0-0.72)
SBA	1 (0-3)	70 (0-319)	0.15 (0-0.71)
**Facility-Based Interventions**			
Facility births	1 (0-3)	70 (0-319)	0.15 (0-0.71)
nonEmOC services	1 (0-3)	69 (0-319)	0.15 (0-0.71)
bEmOC services	1 (0-3)	69 (0-319)	0.15 (0-0.71)
cEmOC services	1 (0-3)	69 (0-321)	0.15 (0-0.7)
**System-Relevant Interventions**			
Quality of care	1 (0-3)	73 (0-372)	0.15 (0-0.76)
Referral	1 (0-3)	70 (0-319)	0.15 (0-0.71)
Transport	1 (0-3)	64 (0-319)	0.14 (0-0.71)
Targeted transfers	1 (0-3)	70 (0-319)	0.15 (0-0.71)
**Integrated Strategies**			
Family Planning	1 (0-3)	59 (0-296)	0.12 (0-0.56)
Community + Linkages	1 (0-3)	66 (0-319)	0.14 (0-0.73)
Facilities + Linkages	1 (0-3)	67 (0-336)	0.14 (0-0.71)
Facilities + Linkages + Quality	1 (0-3)	64 (0-319)	0.14 (0-0.75)
Comprehensive	0 (0-3)	53 (0-284)	0.11 (0-0.57)

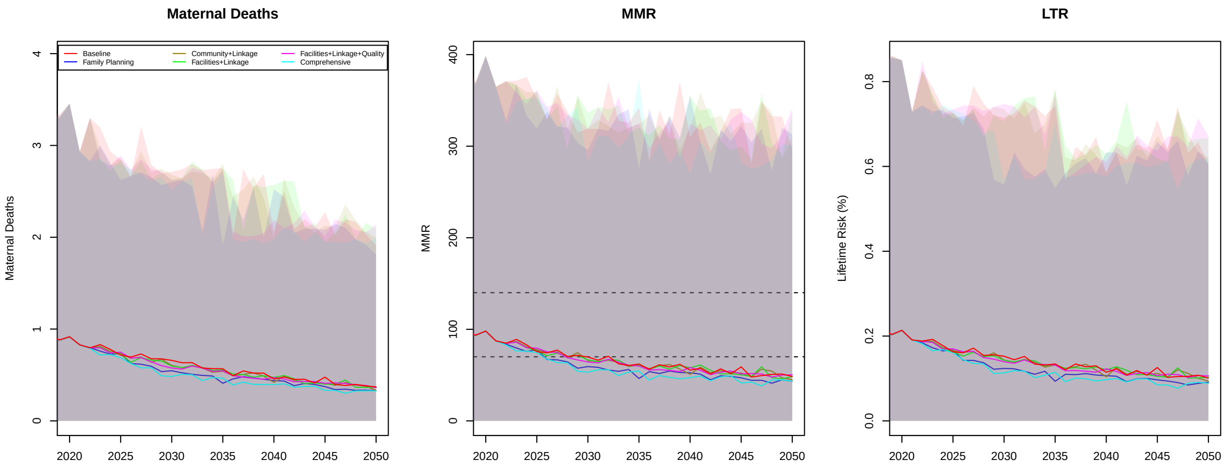

Saint Lucia

ISO Code	Region	Area	Income Group
LCA	Caribbean	Latin America and the Caribbean	Upper middle income

Projected Maternal Indicators in 2030 by Scenario

Scenario	Maternal Deaths	MMR	LTR
**Baseline**	1 (0-6)	46 (0-297)	0.06 (0-0.38)
**Family Planning Interventions**			
Contraception	1 (0-6)	42 (0-252)	0.06 (0-0.37)
Medical abortion	1 (0-6)	42 (0-279)	0.05 (0-0.36)
**Community-Based Interventions**			
ANC	1 (0-6)	47 (0-290)	0.06 (0-0.38)
SBA	1 (0-6)	45 (0-295)	0.06 (0-0.38)
**Facility-Based Interventions**			
Facility births	1 (0-6)	45 (0-297)	0.06 (0-0.38)
nonEmOC services	1 (0-6)	49 (0-308)	0.06 (0-0.4)
bEmOC services	1 (0-6)	47 (0-311)	0.06 (0-0.39)
cEmOC services	1 (0-6)	46 (0-331)	0.06 (0-0.38)
**System-Relevant Interventions**			
Quality of care	1 (0-6)	51 (0-332)	0.07 (0-0.41)
Referral	1 (0-6)	47 (0-292)	0.06 (0-0.38)
Transport	1 (0-6)	45 (0-285)	0.06 (0-0.38)
Targeted transfers	1 (0-6)	43 (0-277)	0.05 (0-0.34)
**Integrated Strategies**			
Family Planning	1 (0-6)	43 (0-258)	0.05 (0-0.34)
Community + Linkages	1 (0-6)	45 (0-279)	0.06 (0-0.4)
Facilities + Linkages	1 (0-6)	46 (0-299)	0.06 (0-0.38)
Facilities + Linkages + Quality	1 (0-6)	49 (0-303)	0.06 (0-0.38)
Comprehensive	1 (0-6)	40 (0-257)	0.05 (0-0.35)

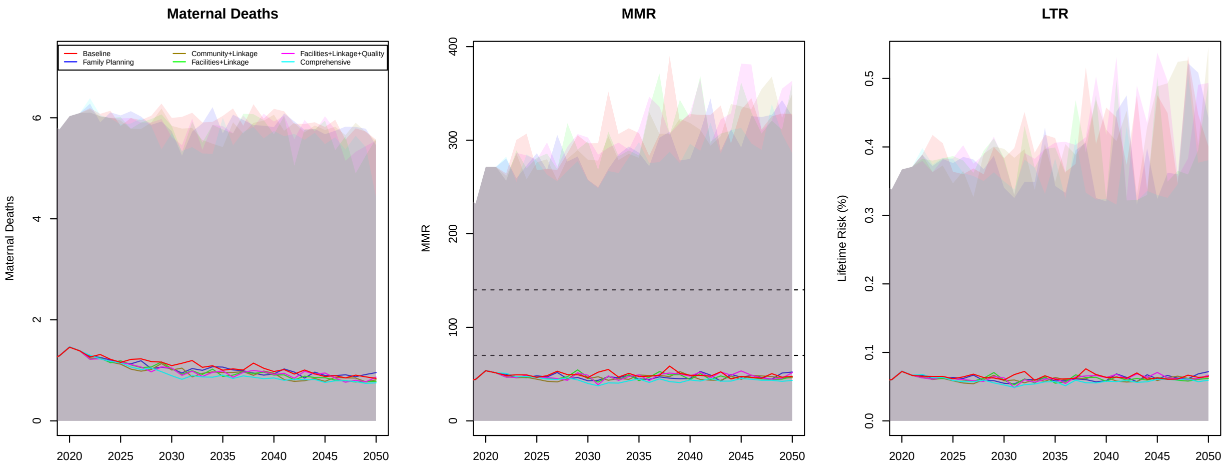

Saint Vincent and the Grenadines

ISO Code	Region	Area	Income Group
VCT	Caribbean	Latin America and the Caribbean	Upper middle income

Projected Maternal Indicators in 2030 by Scenario

Scenario	Maternal Deaths	MMR	LTR
**Baseline**	1 (0-6)	59 (0-385)	0.12 (0-0.82)
**Family Planning Interventions**			
Contraception	1 (0-4)	40 (0-232)	0.08 (0-0.49)
Medical abortion	1 (0-4)	35 (0-242)	0.07 (0-0.53)
**Community-Based Interventions**			
ANC	1 (0-7)	61 (0-432)	0.13 (0-0.88)
SBA	1 (0-6)	55 (0-392)	0.12 (0-0.82)
**Facility-Based Interventions**			
Facility births	1 (0-6)	52 (0-361)	0.11 (0-0.76)
nonEmOC services	1 (0-6)	59 (0-385)	0.12 (0-0.82)
bEmOC services	1 (0-6)	59 (0-387)	0.12 (0-0.82)
cEmOC services	1 (0-6)	59 (0-385)	0.12 (0-0.75)
**System-Relevant Interventions**			
Quality of care	1 (0-6)	60 (0-375)	0.12 (0-0.75)
Referral	1 (0-6)	59 (0-385)	0.12 (0-0.89)
Transport	1 (0-6)	60 (0-375)	0.12 (0-0.79)
Targeted transfers	1 (0-6)	55 (0-361)	0.11 (0-0.76)
**Integrated Strategies**			
Family Planning	1 (0-3)	30 (0-200)	0.06 (0-0.41)
Community + Linkages	1 (0-5)	48 (0-308)	0.1 (0-0.66)
Facilities + Linkages	1 (0-5)	46 (0-288)	0.09 (0-0.63)
Facilities + Linkages + Quality	1 (0-5)	45 (0-282)	0.09 (0-0.65)
Comprehensive	0 (0-2)	18 (0-164)	0.04 (0-0.31)

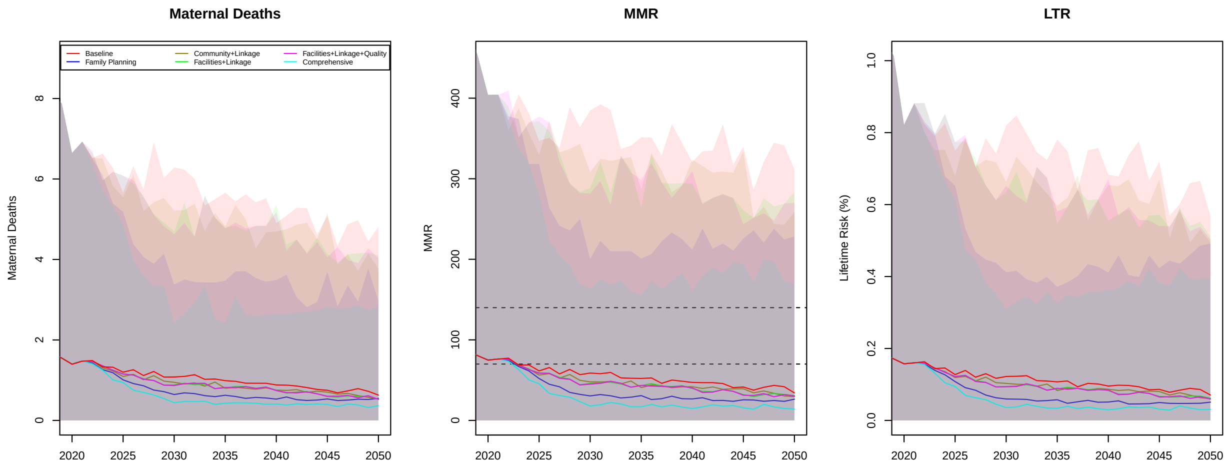

Samoa

ISO Code	Region	Area	Income Group
WSM	Polynesia	Oceania	Upper middle income

Projected Maternal Indicators in 2030 by Scenario

Scenario	Maternal Deaths	MMR	LTR
**Baseline**	8 (0-21)	98 (0-258)	0.46 (0-1.21)
**Family Planning Interventions**			
Contraception	4 (0-12)	88 (0-271)	0.22 (0-0.66)
Medical abortion	8 (0-21)	97 (0-259)	0.46 (0-1.23)
**Community-Based Interventions**			
ANC	8 (0-20)	91 (0-236)	0.43 (0-1.16)
SBA	8 (0-21)	99 (0-249)	0.46 (0-1.21)
**Facility-Based Interventions**			
Facility births	8 (0-21)	99 (0-257)	0.46 (0-1.21)
nonEmOC services	8 (0-21)	98 (0-258)	0.46 (0-1.21)
bEmOC services	9 (0-22)	100 (0-259)	0.47 (0-1.24)
cEmOC services	8 (0-20)	94 (0-245)	0.44 (0-1.11)
**System-Relevant Interventions**			
Quality of care	8 (0-22)	96 (0-258)	0.45 (0-1.25)
Referral	8 (0-21)	98 (0-259)	0.46 (0-1.23)
Transport	8 (0-20)	95 (0-231)	0.45 (0-1.1)
Targeted transfers	8 (0-20)	98 (0-248)	0.46 (0-1.17)
**Integrated Strategies**			
Family Planning	4 (0-13)	87 (0-270)	0.22 (0-0.67)
Community + Linkages	8 (0-18)	92 (0-228)	0.43 (0-1.08)
Facilities + Linkages	8 (0-20)	93 (0-249)	0.44 (0-1.2)
Facilities + Linkages + Quality	8 (0-20)	93 (0-246)	0.43 (0-1.2)
Comprehensive	4 (0-11)	79 (0-252)	0.2 (0-0.63)

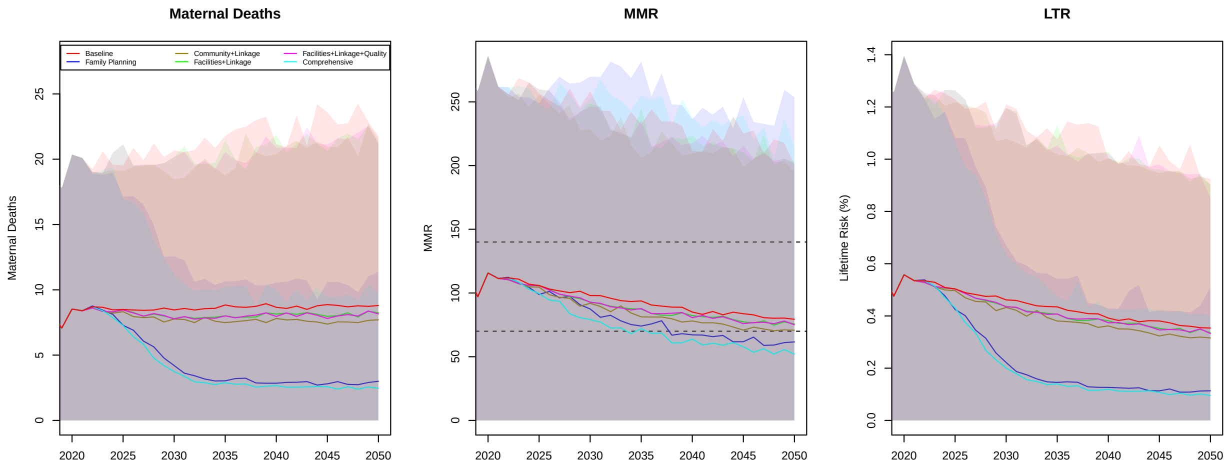

San Marino

ISO Code	Region	Area	Income Group
SMR	Southern Europe	Europe	High income

Projected Maternal Indicators in 2030 by Scenario

Scenario	Maternal Deaths	MMR	LTR
**Baseline**	0 (0-2)	18 (0-276)	0.05 (0-0.74)
**Family Planning Interventions**			
Contraception	0 (0-2)	15 (0-264)	0.04 (0-0.72)
Medical abortion	0 (0-2)	18 (0-276)	0.05 (0-0.74)
**Community-Based Interventions**			
ANC	0 (0-2)	18 (0-276)	0.05 (0-0.74)
SBA	0 (0-2)	18 (0-276)	0.05 (0-0.74)
**Facility-Based Interventions**			
Facility births	0 (0-2)	18 (0-278)	0.05 (0-0.72)
nonEmOC services	0 (0-2)	18 (0-276)	0.05 (0-0.74)
bEmOC services	0 (0-2)	18 (0-276)	0.05 (0-0.74)
cEmOC services	0 (0-2)	20 (0-296)	0.05 (0-0.77)
**System-Relevant Interventions**			
Quality of care	0 (0-2)	18 (0-276)	0.05 (0-0.74)
Referral	0 (0-2)	18 (0-276)	0.05 (0-0.74)
Transport	0 (0-2)	17 (0-276)	0.04 (0-0.72)
Targeted transfers	0 (0-2)	18 (0-276)	0.05 (0-0.74)
**Integrated Strategies**			
Family Planning	0 (0-2)	14 (0-262)	0.04 (0-0.72)
Community + Linkages	0 (0-2)	18 (0-276)	0.05 (0-0.74)
Facilities + Linkages	0 (0-2)	21 (0-314)	0.05 (0-0.76)
Facilities + Linkages + Quality	0 (0-2)	21 (0-314)	0.05 (0-0.76)
Comprehensive	0 (0-2)	13 (0-265)	0.03 (0-0.69)

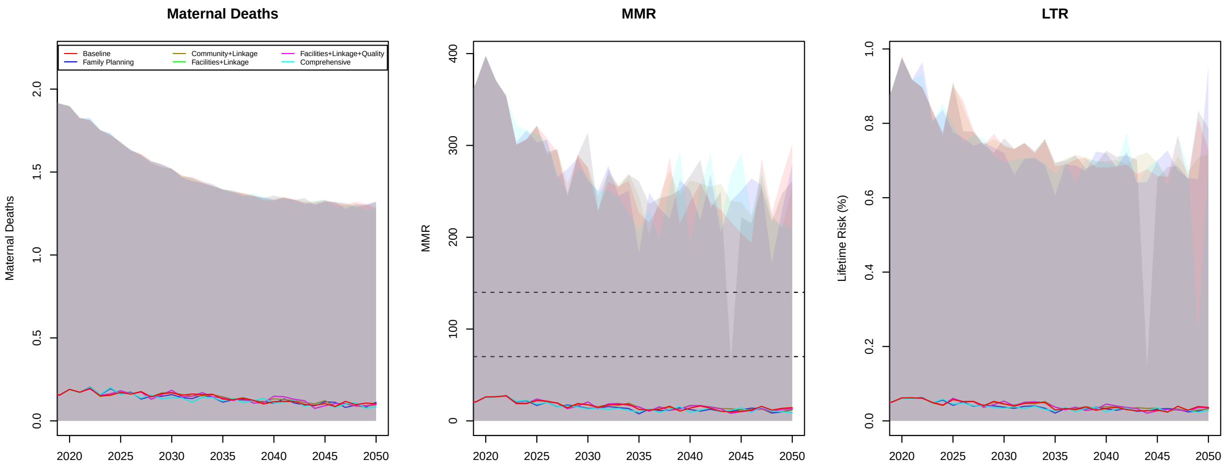

Sao Tome and Principe

ISO Code	Region	Area	Income Group
STP	Middle Africa	Africa	Lower middle income

Projected Maternal Indicators in 2030 by Scenario

Scenario	Maternal Deaths	MMR	LTR
**Baseline**	15 (4-31)	126 (23-271)	0.59 (0.09-1.33)
**Family Planning Interventions**			
Contraception	14 (3-29)	119 (21-270)	0.54 (0.08-1.2)
Medical abortion	15 (3-30)	120 (24-265)	0.56 (0.09-1.23)
**Community-Based Interventions**			
ANC	15 (4-30)	123 (28-270)	0.58 (0.09-1.29)
SBA	15 (3-31)	124 (22-268)	0.59 (0.1-1.38)
**Facility-Based Interventions**			
Facility births	14 (2-30)	119 (18-271)	0.56 (0.05-1.22)
nonEmOC services	15 (4-31)	126 (29-275)	0.59 (0.11-1.3)
bEmOC services	15 (4-30)	126 (29-276)	0.59 (0.1-1.28)
cEmOC services	13 (2-28)	105 (12-259)	0.49 (0.04-1.19)
**System-Relevant Interventions**			
Quality of care	11 (1-25)	83 (6-212)	0.39 (0.02-1.01)
Referral	15 (3-32)	125 (26-280)	0.59 (0.11-1.34)
Transport	15 (3-32)	124 (18-282)	0.58 (0.07-1.38)
Targeted transfers	15 (3-31)	126 (24-274)	0.6 (0.08-1.33)
**Integrated Strategies**			
Family Planning	14 (3-30)	119 (19-269)	0.53 (0.07-1.2)
Community + Linkages	14 (3-29)	118 (20-263)	0.56 (0.07-1.28)
Facilities + Linkages	12 (1-27)	95 (8-247)	0.44 (0.03-1.17)
Facilities + Linkages + Quality	7 (0-20)	51 (0-178)	0.24 (0-0.87)
Comprehensive	6 (0-18)	47 (0-164)	0.21 (0-0.73)

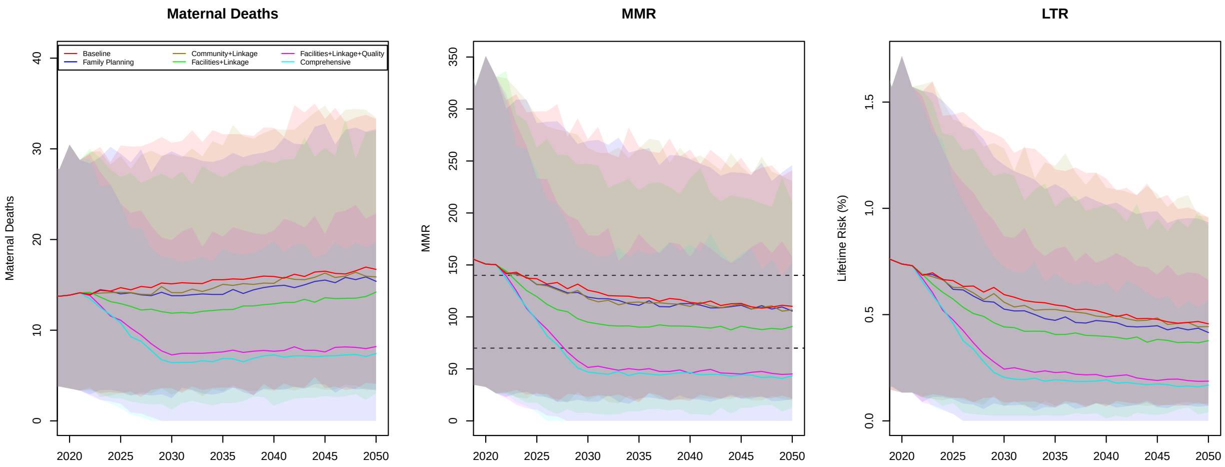

Saudi Arabia

ISO Code	Region	Area	Income Group
SAU	Western Asia	Asia	High income

Projected Maternal Indicators in 2030 by Scenario

Scenario	Maternal Deaths	MMR	LTR
**Baseline**	188 (36-434)	16 (0-47)	0.04 (0-0.11)
**Family Planning Interventions**			
Contraception	183 (34-411)	16 (0-45)	0.04 (0-0.11)
Medical abortion	190 (35-442)	16 (0-45)	0.04 (0-0.11)
**Community-Based Interventions**			
ANC	186 (34-429)	16 (0-46)	0.04 (0-0.11)
SBA	183 (29-407)	15 (0-48)	0.04 (0-0.11)
**Facility-Based Interventions**			
Facility births	182 (34-403)	15 (0-42)	0.04 (0-0.1)
nonEmOC services	187 (36-434)	16 (0-47)	0.04 (0-0.11)
bEmOC services	188 (36-434)	16 (0-47)	0.04 (0-0.11)
cEmOC services	189 (33-437)	16 (0-47)	0.04 (0-0.12)
**System-Relevant Interventions**			
Quality of care	187 (35-430)	16 (0-48)	0.04 (0-0.11)
Referral	190 (35-452)	16 (0-48)	0.04 (0-0.12)
Transport	192 (33-442)	16 (0-50)	0.04 (0-0.12)
Targeted transfers	187 (35-434)	16 (0-46)	0.04 (0-0.11)
**Integrated Strategies**			
Family Planning	184 (29-414)	15 (0-48)	0.04 (0-0.11)
Community + Linkages	186 (34-446)	15 (0-45)	0.04 (0-0.11)
Facilities + Linkages	177 (29-411)	15 (0-43)	0.03 (0-0.11)
Facilities + Linkages + Quality	172 (29-411)	14 (0-43)	0.03 (0-0.11)
Comprehensive	169 (23-394)	14 (0-37)	0.03 (0-0.1)

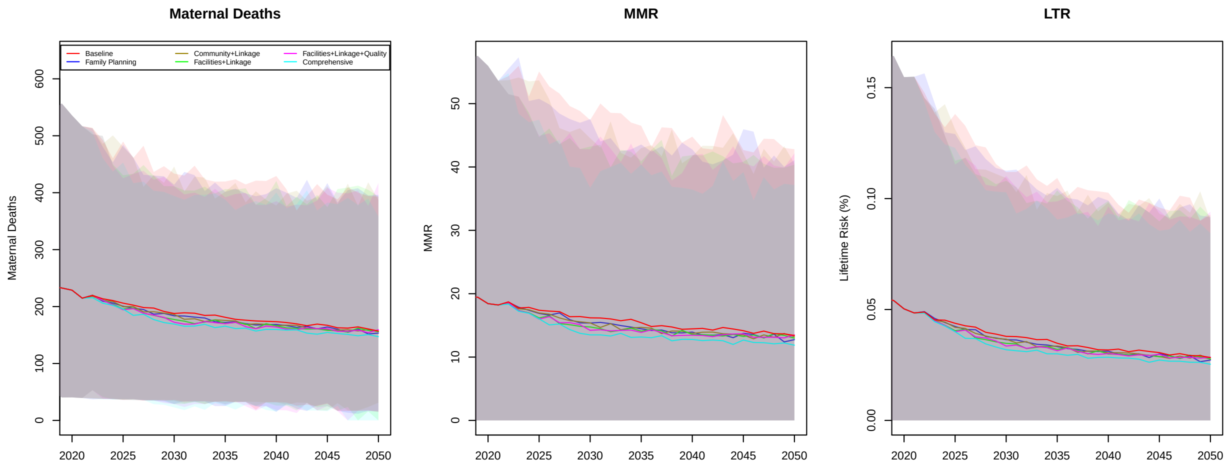

Senegal

ISO Code	Region	Area	Income Group
SEN	Western Africa	Africa	Low income

Projected Maternal Indicators in 2030 by Scenario

Scenario	Maternal Deaths	MMR	LTR
**Baseline**	2137 (1094-3340)	248 (134-380)	1.02 (0.48-1.63)
**Family Planning Interventions**			
Contraception	1994 (1048-3123)	247 (135-378)	0.93 (0.44-1.51)
Medical abortion	2160 (1069-3318)	250 (133-365)	1.02 (0.47-1.6)
**Community-Based Interventions**			
ANC	2109 (1068-3331)	244 (131-368)	1 (0.45-1.64)
SBA	2124 (1110-3322)	246 (141-361)	1.01 (0.48-1.61)
**Facility-Based Interventions**			
Facility births	1946 (936-3103)	224 (113-347)	0.91 (0.42-1.49)
nonEmOC services	2137 (1081-3376)	247 (134-376)	1.01 (0.47-1.64)
bEmOC services	2127 (1067-3342)	246 (134-374)	1.01 (0.48-1.63)
cEmOC services	2069 (1019-3288)	239 (129-361)	0.98 (0.43-1.64)
**System-Relevant Interventions**			
Quality of care	787 (257-1458)	69 (16-144)	0.29 (0.06-0.63)
Referral	2108 (1107-3214)	244 (132-368)	1 (0.47-1.61)
Transport	2070 (1084-3207)	241 (135-361)	0.99 (0.49-1.59)
Targeted transfers	2122 (1146-3370)	247 (136-381)	1 (0.49-1.63)
**Integrated Strategies**			
Family Planning	1974 (998-3174)	246 (133-372)	0.93 (0.42-1.5)
Community + Linkages	2000 (981-3204)	231 (122-348)	0.94 (0.45-1.54)
Facilities + Linkages	1874 (956-2989)	214 (117-334)	0.87 (0.41-1.45)
Facilities + Linkages + Quality	468 (103-940)	30 (0-74)	0.12 (0-0.33)
Comprehensive	410 (82-889)	27 (0-73)	0.1 (0-0.32)

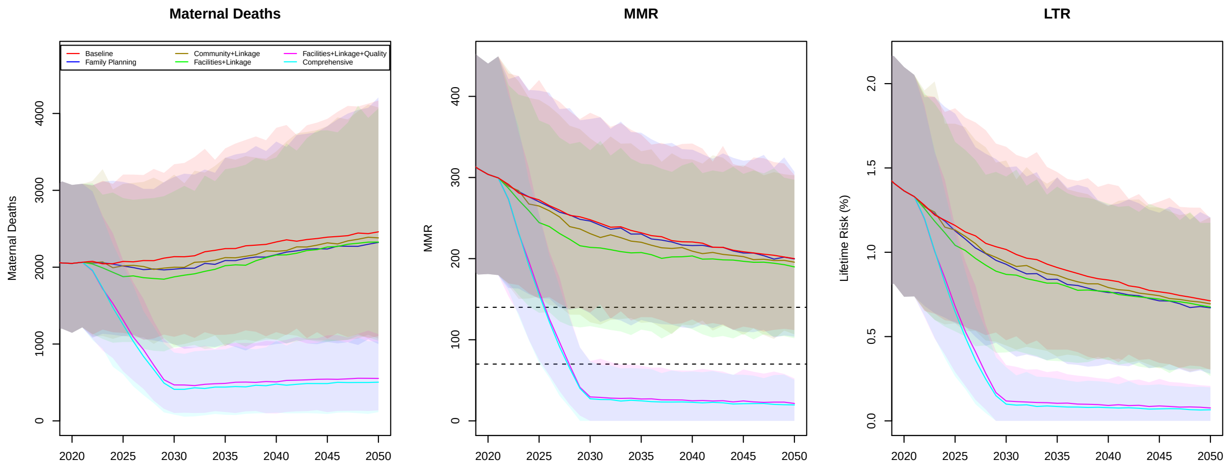

Serbia

ISO Code	Region	Area	Income Group
SRB	Southern Europe	Europe	Upper middle income

Projected Maternal Indicators in 2030 by Scenario

Scenario	Maternal Deaths	MMR	LTR
**Baseline**	24 (4-53)	18 (0-55)	0.03 (0-0.09)
**Family Planning Interventions**			
Contraception	21 (4-46)	16 (0-53)	0.02 (0-0.07)
Medical abortion	24 (4-49)	17 (0-53)	0.02 (0-0.08)
**Community-Based Interventions**			
ANC	24 (4-52)	18 (0-56)	0.03 (0-0.08)
SBA	24 (4-53)	18 (0-55)	0.03 (0-0.09)
**Facility-Based Interventions**			
Facility births	24 (4-53)	18 (0-56)	0.03 (0-0.09)
nonEmOC services	24 (4-53)	18 (0-55)	0.03 (0-0.08)
bEmOC services	24 (4-53)	18 (0-56)	0.03 (0-0.08)
cEmOC services	24 (4-51)	18 (0-54)	0.03 (0-0.08)
**System-Relevant Interventions**			
Quality of care	24 (4-53)	18 (0-55)	0.03 (0-0.09)
Referral	24 (4-53)	18 (0-55)	0.03 (0-0.08)
Transport	23 (4-52)	18 (0-53)	0.03 (0-0.08)
Targeted transfers	24 (4-53)	18 (0-55)	0.03 (0-0.08)
**Integrated Strategies**			
Family Planning	21 (4-46)	15 (0-48)	0.02 (0-0.07)
Community + Linkages	23 (4-49)	17 (0-57)	0.03 (0-0.08)
Facilities + Linkages	23 (4-47)	18 (0-54)	0.03 (0-0.08)
Facilities + Linkages + Quality	23 (4-47)	18 (0-55)	0.03 (0-0.08)
Comprehensive	19 (0-44)	15 (0-49)	0.02 (0-0.07)

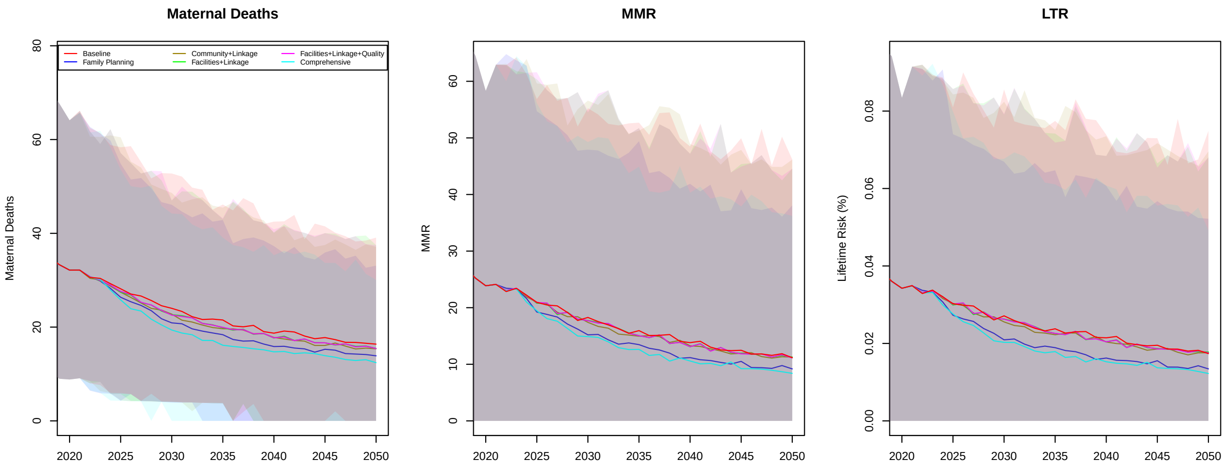

Seychelles

ISO Code	Region	Area	Income Group
SYC	Eastern Africa	Africa	High income

Projected Maternal Indicators in 2030 by Scenario

Scenario	Maternal Deaths	MMR	LTR
**Baseline**	2 (0-6)	86 (0-359)	0.2 (0-0.8)
**Family Planning Interventions**			
Contraception	1 (0-5)	77 (0-304)	0.16 (0-0.66)
Medical abortion	1 (0-5)	70 (0-280)	0.16 (0-0.62)
**Community-Based Interventions**			
ANC	2 (0-6)	86 (0-356)	0.2 (0-0.8)
SBA	1 (0-5)	84 (0-346)	0.19 (0-0.76)
**Facility-Based Interventions**			
Facility births	2 (0-6)	89 (0-380)	0.2 (0-0.81)
nonEmOC services	1 (0-6)	85 (0-349)	0.2 (0-0.78)
bEmOC services	2 (0-6)	85 (0-356)	0.2 (0-0.78)
cEmOC services	2 (0-6)	87 (0-346)	0.2 (0-0.77)
**System-Relevant Interventions**			
Quality of care	1 (0-6)	85 (0-352)	0.19 (0-0.78)
Referral	1 (0-6)	85 (0-352)	0.19 (0-0.77)
Transport	2 (0-6)	87 (0-352)	0.2 (0-0.81)
Targeted transfers	1 (0-6)	86 (0-359)	0.19 (0-0.8)
**Integrated Strategies**			
Family Planning	1 (0-5)	64 (0-288)	0.14 (0-0.6)
Community + Linkages	2 (0-6)	89 (0-357)	0.2 (0-0.82)
Facilities + Linkages	2 (0-6)	89 (0-360)	0.21 (0-0.81)
Facilities + Linkages + Quality	2 (0-6)	89 (0-359)	0.2 (0-0.81)
Comprehensive	1 (0-4)	67 (0-272)	0.14 (0-0.57)

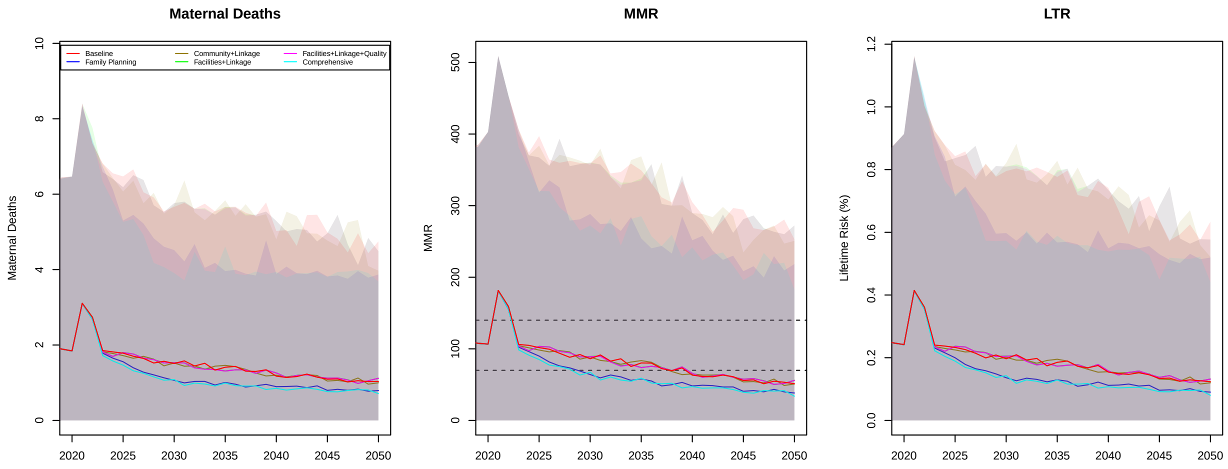

Sierra Leone

ISO Code	Region	Area	Income Group
SLE	Western Africa	Africa	Low income

Projected Maternal Indicators in 2030 by Scenario

Scenario	Maternal Deaths	MMR	LTR
**Baseline**	1990 (1053-3276)	511 (305-794)	2 (1.01-3.59)
**Family Planning Interventions**			
Contraception	1769 (966-3042)	490 (288-758)	1.75 (0.89-3.26)
Medical abortion	1999 (1107-3275)	514 (308-802)	2.01 (1.01-3.61)
**Community-Based Interventions**			
ANC	1952 (1077-3084)	502 (308-753)	1.96 (0.97-3.46)
SBA	1961 (1093-3199)	504 (310-787)	1.98 (1.01-3.52)
**Facility-Based Interventions**			
Facility births	1456 (704-2515)	363 (189-617)	1.44 (0.62-2.74)
nonEmOC services	1975 (1075-3276)	507 (296-775)	1.98 (1.01-3.59)
bEmOC services	1978 (1103-3241)	510 (309-783)	1.99 (1.04-3.55)
cEmOC services	1970 (1084-3257)	505 (299-760)	1.98 (1.03-3.44)
**System-Relevant Interventions**			
Quality of care	1278 (419-2355)	314 (84-584)	1.22 (0.28-2.69)
Referral	1971 (1075-3299)	504 (288-786)	1.97 (0.99-3.52)
Transport	1875 (1015-3031)	481 (290-738)	1.89 (0.9-3.27)
Targeted transfers	1979 (1066-3259)	511 (296-780)	2 (0.99-3.5)
**Integrated Strategies**			
Family Planning	1775 (931-2940)	493 (288-743)	1.75 (0.84-3.17)
Community + Linkages	1706 (915-2827)	435 (250-664)	1.72 (0.81-3.1)
Facilities + Linkages	1434 (708-2537)	360 (178-604)	1.43 (0.64-2.79)
Facilities + Linkages + Quality	476 (99-1344)	96 (8-313)	0.4 (0-1.53)
Comprehensive	418 (84-1151)	88 (8-289)	0.33 (0-1.27)

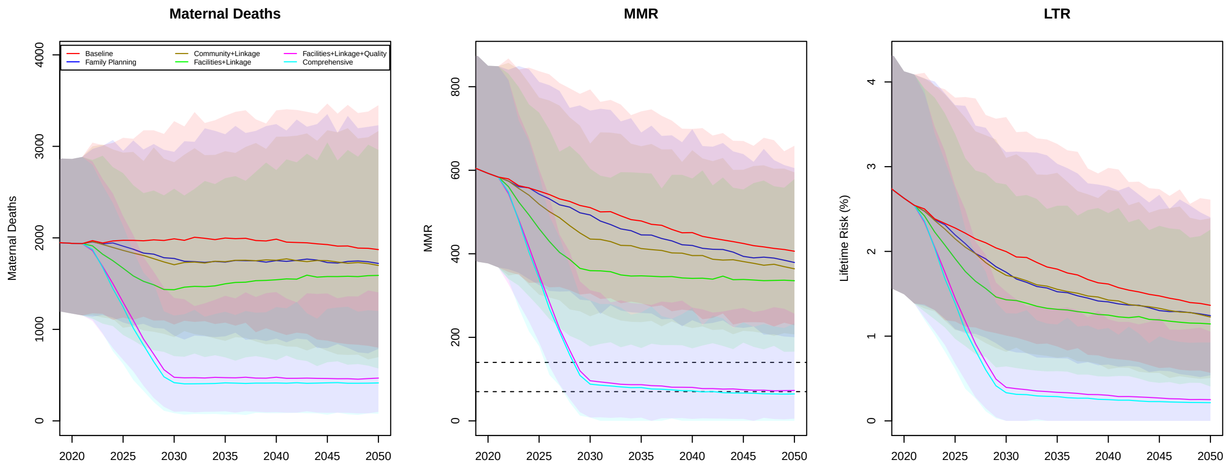

Singapore

ISO Code	Region	Area	Income Group
SGP	South-Eastern Asia	Asia	High income

Projected Maternal Indicators in 2030 by Scenario

Scenario	Maternal Deaths	MMR	LTR
**Baseline**	11 (0-39)	21 (0-84)	0.02 (0-0.08)
**Family Planning Interventions**			
Contraception	10 (0-32)	19 (0-73)	0.02 (0-0.08)
Medical abortion	11 (0-39)	21 (0-84)	0.02 (0-0.08)
**Community-Based Interventions**			
ANC	10 (0-32)	19 (0-77)	0.02 (0-0.08)
SBA	11 (0-32)	21 (0-84)	0.02 (0-0.08)
**Facility-Based Interventions**			
Facility births	10 (0-33)	20 (0-82)	0.02 (0-0.08)
nonEmOC services	11 (0-39)	21 (0-84)	0.02 (0-0.08)
bEmOC services	11 (0-39)	21 (0-84)	0.02 (0-0.08)
cEmOC services	10 (0-32)	19 (0-78)	0.02 (0-0.08)
**System-Relevant Interventions**			
Quality of care	11 (0-32)	21 (0-81)	0.02 (0-0.08)
Referral	11 (0-39)	21 (0-83)	0.02 (0-0.08)
Transport	10 (0-40)	20 (0-82)	0.02 (0-0.08)
Targeted transfers	11 (0-39)	21 (0-84)	0.02 (0-0.08)
**Integrated Strategies**			
Family Planning	10 (0-32)	19 (0-73)	0.02 (0-0.08)
Community + Linkages	10 (0-32)	18 (0-80)	0.02 (0-0.08)
Facilities + Linkages	10 (0-40)	19 (0-83)	0.02 (0-0.08)
Facilities + Linkages + Quality	10 (0-40)	19 (0-80)	0.02 (0-0.08)
Comprehensive	9 (0-32)	17 (0-71)	0.02 (0-0.08)

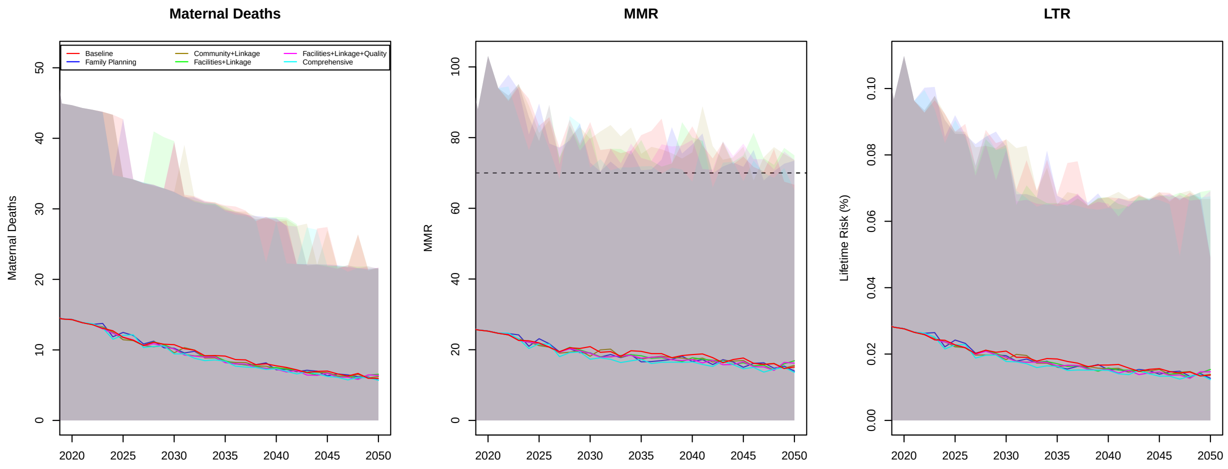

Slovakia

ISO Code	Region	Area	Income Group
SVK	Eastern Europe	Europe	High income

Projected Maternal Indicators in 2030 by Scenario

Scenario	Maternal Deaths	MMR	LTR
**Baseline**	28 (0-86)	47 (0-153)	0.07 (0-0.23)
**Family Planning Interventions**			
Contraception	26 (0-80)	43 (0-143)	0.06 (0-0.21)
Medical abortion	25 (0-73)	40 (0-129)	0.06 (0-0.19)
**Community-Based Interventions**			
ANC	27 (0-92)	44 (0-148)	0.06 (0-0.25)
SBA	28 (0-86)	47 (0-153)	0.07 (0-0.23)
**Facility-Based Interventions**			
Facility births	28 (0-84)	47 (0-153)	0.07 (0-0.23)
nonEmOC services	28 (0-86)	47 (0-153)	0.07 (0-0.23)
bEmOC services	28 (0-92)	47 (0-153)	0.07 (0-0.24)
cEmOC services	28 (0-86)	47 (0-152)	0.07 (0-0.22)
**System-Relevant Interventions**			
Quality of care	28 (0-86)	47 (0-153)	0.07 (0-0.23)
Referral	28 (0-84)	47 (0-153)	0.07 (0-0.23)
Transport	27 (0-83)	45 (0-151)	0.07 (0-0.23)
Targeted transfers	28 (0-84)	47 (0-153)	0.07 (0-0.23)
**Integrated Strategies**			
Family Planning	25 (0-74)	40 (0-130)	0.06 (0-0.2)
Community + Linkages	26 (0-83)	44 (0-150)	0.07 (0-0.24)
Facilities + Linkages	27 (0-83)	45 (0-149)	0.07 (0-0.22)
Facilities + Linkages + Quality	27 (0-83)	45 (0-149)	0.07 (0-0.22)
Comprehensive	25 (0-74)	40 (0-137)	0.06 (0-0.2)

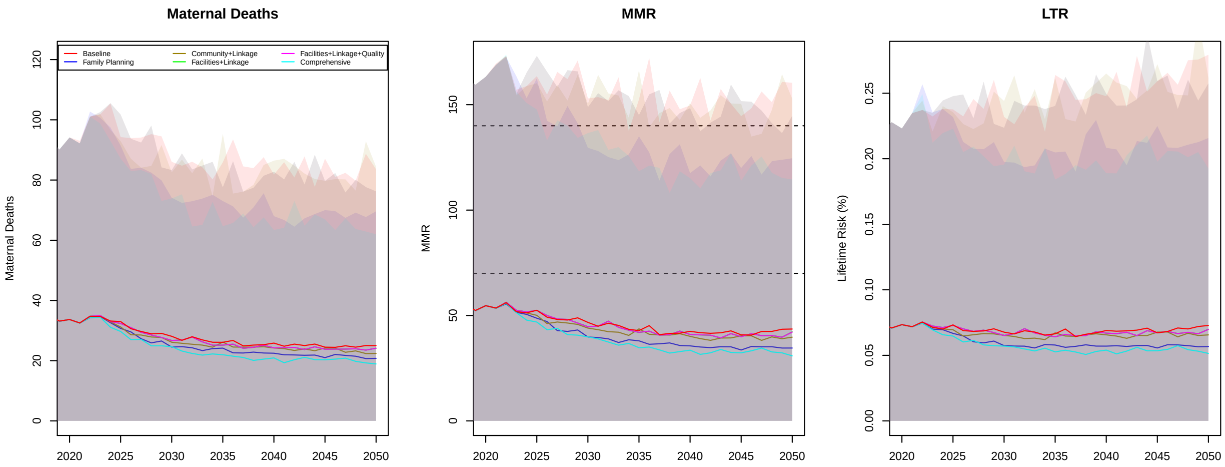

Slovenia

ISO Code	Region	Area	Income Group
SVN	Southern Europe	Europe	High income

Projected Maternal Indicators in 2030 by Scenario

Scenario	Maternal Deaths	MMR	LTR
**Baseline**	7 (0-20)	25 (0-96)	0.04 (0-0.16)
**Family Planning Interventions**			
Contraception	7 (0-23)	24 (0-101)	0.04 (0-0.16)
Medical abortion	7 (0-23)	27 (0-103)	0.04 (0-0.16)
**Community-Based Interventions**			
ANC	7 (0-21)	25 (0-90)	0.04 (0-0.15)
SBA	7 (0-21)	25 (0-98)	0.04 (0-0.16)
**Facility-Based Interventions**			
Facility births	7 (0-21)	24 (0-94)	0.04 (0-0.16)
nonEmOC services	7 (0-20)	25 (0-96)	0.04 (0-0.16)
bEmOC services	7 (0-20)	25 (0-96)	0.04 (0-0.16)
cEmOC services	7 (0-21)	25 (0-97)	0.04 (0-0.16)
**System-Relevant Interventions**			
Quality of care	7 (0-20)	25 (0-97)	0.04 (0-0.16)
Referral	7 (0-20)	25 (0-96)	0.04 (0-0.16)
Transport	6 (0-21)	26 (0-103)	0.04 (0-0.16)
Targeted transfers	7 (0-20)	25 (0-96)	0.04 (0-0.16)
**Integrated Strategies**			
Family Planning	7 (0-23)	25 (0-100)	0.04 (0-0.17)
Community + Linkages	6 (0-21)	26 (0-103)	0.04 (0-0.16)
Facilities + Linkages	6 (0-22)	24 (0-91)	0.04 (0-0.16)
Facilities + Linkages + Quality	6 (0-21)	24 (0-91)	0.04 (0-0.16)
Comprehensive	6 (0-21)	24 (0-94)	0.03 (0-0.15)

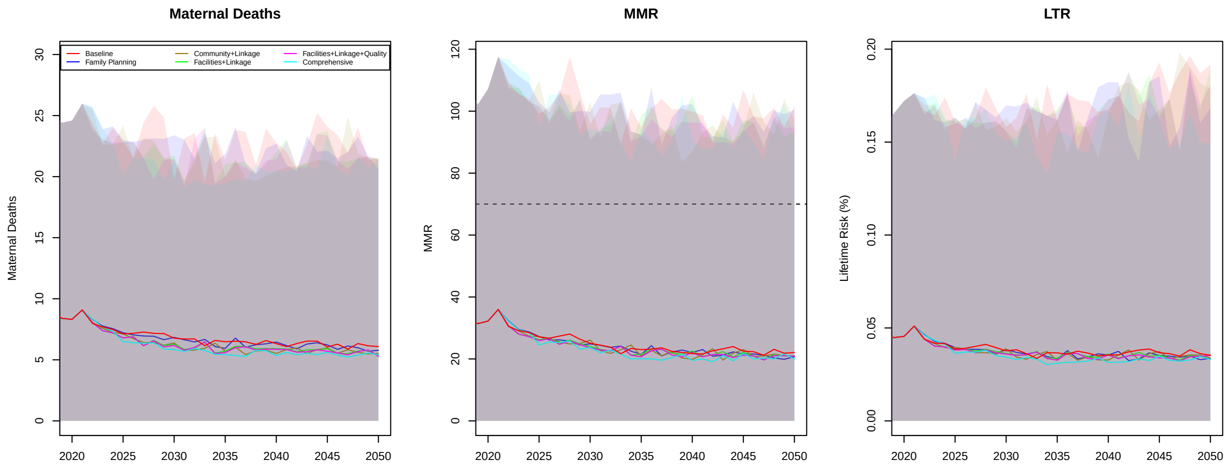

Solomon Islands

ISO Code	Region	Area	Income Group
SLB	Melanesia	Oceania	Lower middle income

Projected Maternal Indicators in 2030 by Scenario

Scenario	Maternal Deaths	MMR	LTR
**Baseline**	49 (9-101)	166 (34-355)	0.64 (0.09-1.44)
**Family Planning Interventions**			
Contraception	43 (9-94)	159 (35-323)	0.56 (0.07-1.24)
Medical abortion	48 (13-99)	161 (37-325)	0.62 (0.11-1.33)
**Community-Based Interventions**			
ANC	48 (12-96)	161 (37-313)	0.62 (0.12-1.3)
SBA	47 (12-97)	161 (36-335)	0.62 (0.11-1.36)
**Facility-Based Interventions**			
Facility births	45 (9-97)	152 (27-306)	0.58 (0.07-1.32)
nonEmOC services	49 (13-100)	167 (36-359)	0.64 (0.11-1.41)
bEmOC services	49 (12-102)	164 (36-345)	0.63 (0.08-1.44)
cEmOC services	46 (9-96)	153 (25-314)	0.59 (0.08-1.32)
**System-Relevant Interventions**			
Quality of care	26 (4-58)	78 (0-183)	0.31 (0-0.77)
Referral	49 (13-102)	166 (37-338)	0.64 (0.13-1.37)
Transport	47 (13-96)	164 (37-332)	0.63 (0.13-1.31)
Targeted transfers	49 (10-102)	164 (36-345)	0.63 (0.11-1.44)
**Integrated Strategies**			
Family Planning	44 (9-88)	162 (25-310)	0.57 (0.07-1.24)
Community + Linkages	45 (9-92)	156 (33-310)	0.6 (0.09-1.31)
Facilities + Linkages	43 (9-90)	144 (28-312)	0.56 (0.07-1.31)
Facilities + Linkages + Quality	18 (0-47)	52 (0-150)	0.2 (0-0.6)
Comprehensive	16 (0-40)	46 (0-140)	0.16 (0-0.52)

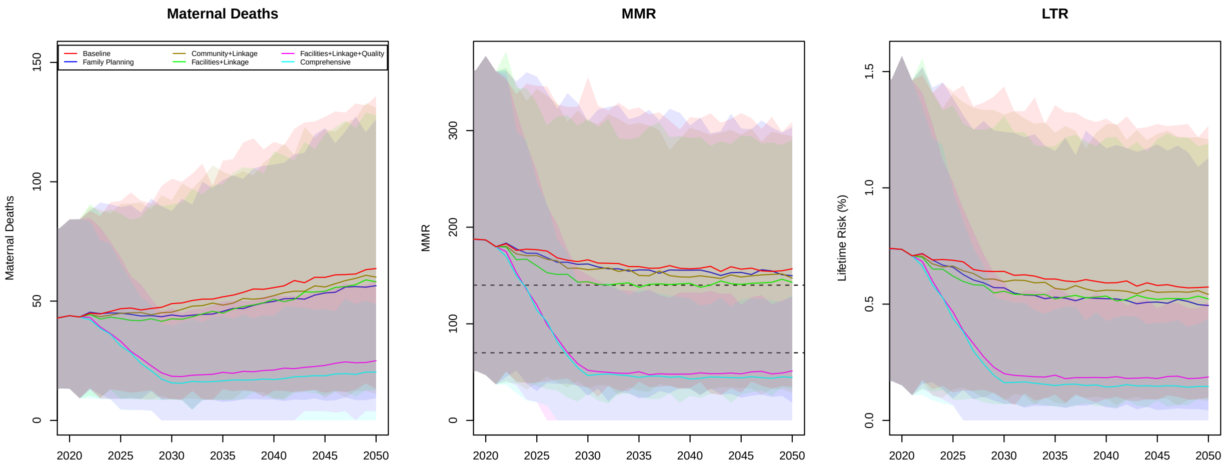

Somalia

ISO Code	Region	Area	Income Group
SOM	Eastern Africa	Africa	Low income

Projected Maternal Indicators in 2030 by Scenario

Scenario	Maternal Deaths	MMR	LTR
**Baseline**	3870 (800-7493)	452 (63-865)	2.15 (0.3-4.33)
**Family Planning Interventions**			
Contraception	3648 (758-7467)	426 (59-818)	2.01 (0.26-4.23)
Medical abortion	3776 (742-7586)	439 (58-858)	2.09 (0.26-4.34)
**Community-Based Interventions**			
ANC	3646 (751-7323)	423 (57-822)	2.01 (0.26-4.17)
SBA	3445 (735-6871)	397 (56-768)	1.91 (0.25-3.96)
**Facility-Based Interventions**			
Facility births	1598 (549-3560)	164 (42-423)	0.81 (0.17-2.12)
nonEmOC services	3879 (803-7608)	453 (64-871)	2.15 (0.3-4.47)
bEmOC services	3843 (761-7641)	448 (54-869)	2.13 (0.26-4.4)
cEmOC services	3808 (696-7569)	444 (52-872)	2.11 (0.23-4.39)
**System-Relevant Interventions**			
Quality of care	3755 (745-7455)	437 (56-857)	2.09 (0.26-4.33)
Referral	3796 (758-7570)	442 (60-864)	2.11 (0.28-4.43)
Transport	3386 (710-6877)	393 (55-759)	1.88 (0.26-3.93)
Targeted transfers	3859 (808-7507)	450 (58-865)	2.14 (0.25-4.4)
**Integrated Strategies**			
Family Planning	3603 (750-7523)	420 (59-836)	1.98 (0.26-4.27)
Community + Linkages	2552 (658-5030)	288 (49-579)	1.39 (0.23-2.99)
Facilities + Linkages	1429 (404-3447)	147 (28-397)	0.73 (0.1-2.11)
Facilities + Linkages + Quality	1288 (375-3262)	129 (25-367)	0.64 (0.08-1.95)
Comprehensive	1135 (342-3024)	110 (20-328)	0.54 (0.06-1.78)

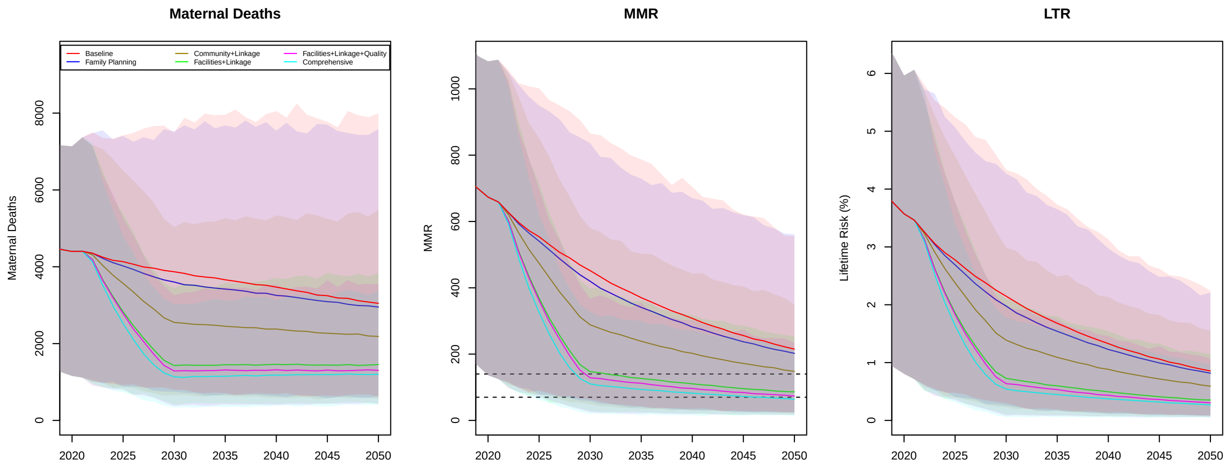

South Africa

ISO Code	Region	Area	Income Group
ZAF	Southern Africa	Africa	Upper middle income

Projected Maternal Indicators in 2030 by Scenario

Scenario	Maternal Deaths	MMR	LTR
**Baseline**	1457 (627-2624)	82 (30-180)	0.22 (0.08-0.43)
**Family Planning Interventions**			
Contraception	1356 (560-2543)	76 (22-166)	0.21 (0.06-0.42)
Medical abortion	1361 (580-2414)	75 (25-165)	0.21 (0.07-0.41)
**Community-Based Interventions**			
ANC	1434 (627-2610)	81 (27-177)	0.22 (0.08-0.44)
SBA	1455 (586-2590)	82 (27-179)	0.22 (0.07-0.44)
**Facility-Based Interventions**			
Facility births	1451 (641-2572)	81 (27-170)	0.22 (0.08-0.43)
nonEmOC services	1459 (625-2624)	82 (30-180)	0.22 (0.08-0.43)
bEmOC services	1456 (627-2596)	82 (30-178)	0.22 (0.08-0.43)
cEmOC services	1451 (629-2555)	82 (28-181)	0.23 (0.08-0.44)
**System-Relevant Interventions**			
Quality of care	1353 (595-2548)	76 (27-178)	0.21 (0.07-0.43)
Referral	1460 (649-2632)	83 (30-180)	0.23 (0.08-0.43)
Transport	1423 (640-2547)	82 (29-182)	0.22 (0.08-0.45)
Targeted transfers	1455 (627-2602)	82 (30-180)	0.22 (0.08-0.43)
**Integrated Strategies**			
Family Planning	1297 (540-2398)	72 (21-149)	0.2 (0.06-0.4)
Community + Linkages	1408 (612-2499)	80 (28-171)	0.22 (0.08-0.43)
Facilities + Linkages	1417 (589-2518)	81 (29-179)	0.22 (0.08-0.44)
Facilities + Linkages + Quality	1292 (544-2493)	73 (24-174)	0.2 (0.06-0.42)
Comprehensive	1129 (428-2206)	62 (16-136)	0.17 (0.04-0.37)

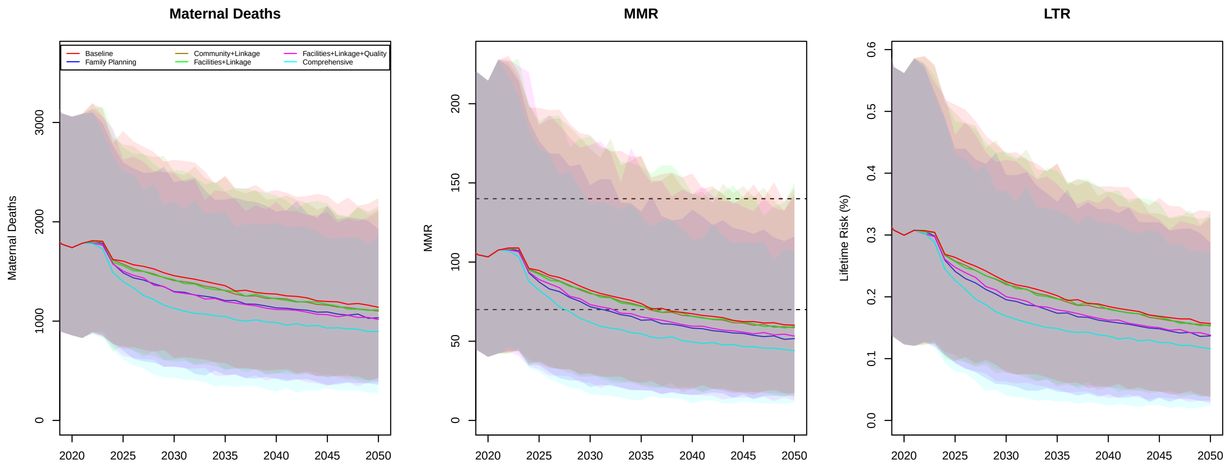

South Sudan

ISO Code	Region	Area	Income Group
SSD	Eastern Africa	Africa	Low income

Projected Maternal Indicators in 2030 by Scenario

Scenario	Maternal Deaths	MMR	LTR
**Baseline**	5018 (2405-8525)	1005 (539-1578)	4.7 (2.17-7.75)
**Family Planning Interventions**			
Contraception	4307 (2054-7593)	897 (480-1356)	3.99 (1.87-6.74)
Medical abortion	4693 (2294-8149)	927 (559-1344)	4.35 (2.1-7.28)
**Community-Based Interventions**			
ANC	4853 (2313-8263)	969 (519-1500)	4.53 (2.11-7.57)
SBA	4688 (2362-7717)	939 (500-1523)	4.43 (2.13-7.5)
**Facility-Based Interventions**			
Facility births	3164 (1269-5566)	625 (237-1218)	3 (1.04-5.44)
nonEmOC services	5040 (2501-8800)	1008 (566-1596)	4.72 (2.18-7.8)
bEmOC services	5001 (2281-8835)	1000 (544-1556)	4.68 (2.18-7.92)
cEmOC services	4982 (2498-8552)	995 (531-1535)	4.67 (2.23-7.9)
**System-Relevant Interventions**			
Quality of care	4639 (2211-8100)	923 (507-1492)	4.37 (2.12-7.63)
Referral	4857 (2399-8320)	973 (521-1547)	4.56 (2.16-7.96)
Transport	4589 (2306-7692)	919 (516-1417)	4.31 (2.06-7.06)
Targeted transfers	5007 (2368-8525)	1004 (533-1565)	4.69 (2.25-7.76)
**Integrated Strategies**			
Family Planning	4215 (2100-7691)	877 (492-1291)	3.87 (1.87-6.76)
Community + Linkages	4000 (1840-6741)	798 (400-1345)	3.73 (1.66-6.28)
Facilities + Linkages	3036 (1158-5516)	602 (208-1111)	2.89 (0.98-5.24)
Facilities + Linkages + Quality	2405 (820-4328)	474 (144-954)	2.31 (0.64-4.26)
Comprehensive	1794 (446-3212)	356 (86-611)	1.67 (0.33-3.15)

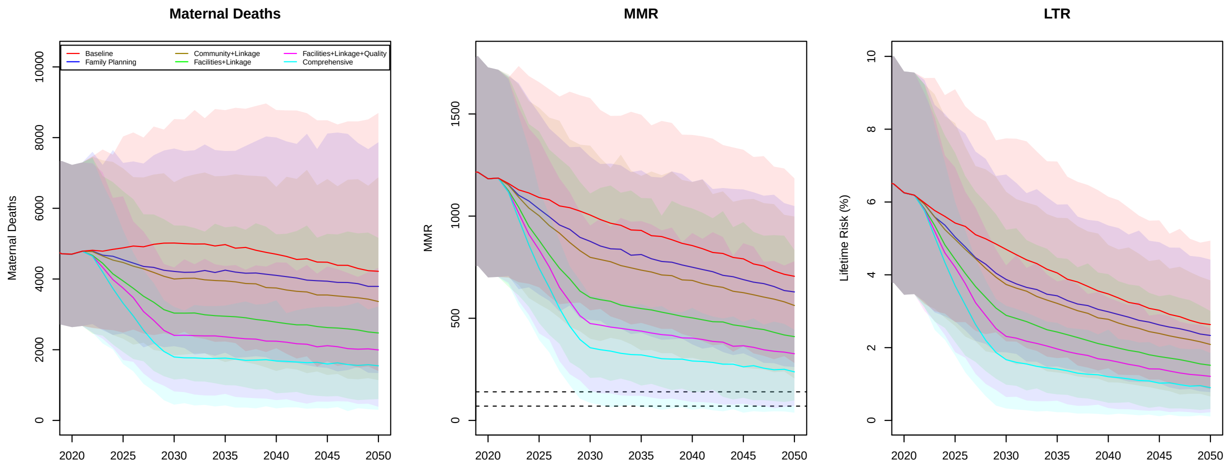

Spain

ISO Code	Region	Area	Income Group
ESP	Southern Europe	Europe	High income

Projected Maternal Indicators in 2030 by Scenario

Scenario	Maternal Deaths	MMR	LTR
**Baseline**	137 (28-271)	17 (0-43)	0.02 (0-0.06)
**Family Planning Interventions**			
Contraception	137 (27-279)	16 (0-42)	0.02 (0-0.06)
Medical abortion	138 (29-281)	17 (0-44)	0.02 (0-0.06)
**Community-Based Interventions**			
ANC	136 (27-276)	17 (0-42)	0.02 (0-0.06)
SBA	138 (28-275)	17 (0-44)	0.02 (0-0.06)
**Facility-Based Interventions**			
Facility births	139 (29-275)	17 (0-44)	0.02 (0-0.06)
nonEmOC services	137 (27-267)	17 (0-42)	0.02 (0-0.06)
bEmOC services	137 (28-271)	17 (0-43)	0.02 (0-0.06)
cEmOC services	139 (27-276)	18 (0-45)	0.02 (0-0.06)
**System-Relevant Interventions**			
Quality of care	137 (28-271)	17 (0-43)	0.02 (0-0.06)
Referral	137 (28-271)	17 (0-44)	0.02 (0-0.06)
Transport	132 (27-264)	16 (0-44)	0.02 (0-0.06)
Targeted transfers	137 (27-275)	17 (0-43)	0.02 (0-0.06)
**Integrated Strategies**			
Family Planning	139 (27-289)	16 (0-43)	0.02 (0-0.06)
Community + Linkages	130 (27-261)	16 (0-43)	0.02 (0-0.06)
Facilities + Linkages	131 (20-264)	16 (0-44)	0.02 (0-0.06)
Facilities + Linkages + Quality	131 (20-264)	16 (0-44)	0.02 (0-0.06)
Comprehensive	133 (27-278)	15 (0-40)	0.02 (0-0.06)

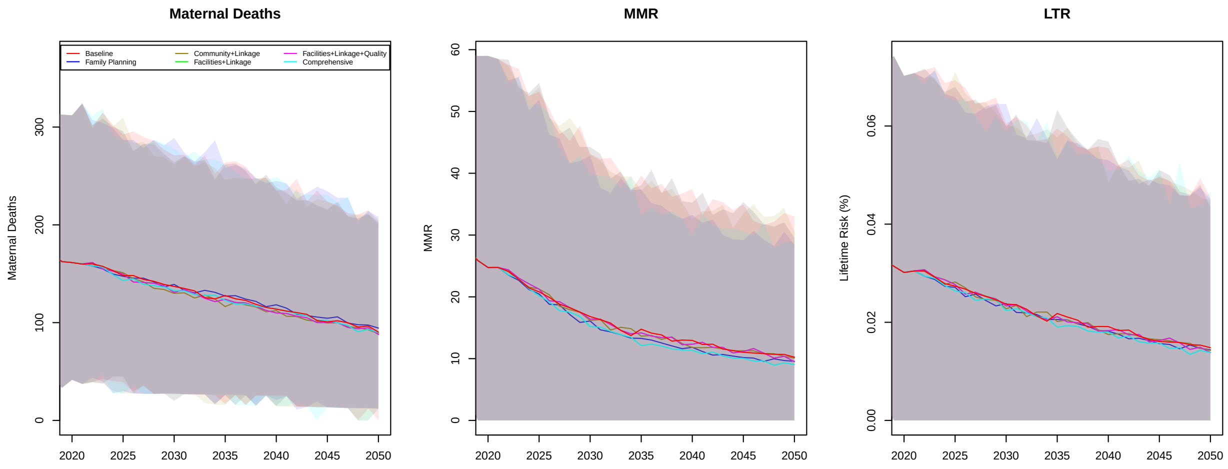

Sri Lanka

ISO Code	Region	Area	Income Group
LKA	Southern Asia	Asia	Lower middle income

Projected Maternal Indicators in 2030 by Scenario

Scenario	Maternal Deaths	MMR	LTR
**Baseline**	166 (0-408)	17 (0-63)	0.04 (0-0.13)
**Family Planning Interventions**			
Contraception	154 (0-391)	17 (0-64)	0.03 (0-0.12)
Medical abortion	169 (0-421)	18 (0-67)	0.04 (0-0.15)
**Community-Based Interventions**			
ANC	163 (0-393)	17 (0-63)	0.03 (0-0.14)
SBA	163 (0-403)	16 (0-62)	0.03 (0-0.14)
**Facility-Based Interventions**			
Facility births	168 (0-424)	17 (0-61)	0.04 (0-0.14)
nonEmOC services	166 (0-416)	16 (0-63)	0.03 (0-0.13)
bEmOC services	166 (0-403)	17 (0-63)	0.04 (0-0.14)
cEmOC services	168 (0-409)	17 (0-63)	0.04 (0-0.14)
**System-Relevant Interventions**			
Quality of care	166 (0-424)	17 (0-63)	0.04 (0-0.14)
Referral	167 (0-397)	17 (0-57)	0.04 (0-0.13)
Transport	152 (0-365)	17 (0-65)	0.04 (0-0.14)
Targeted transfers	167 (0-448)	17 (0-65)	0.04 (0-0.14)
**Integrated Strategies**			
Family Planning	154 (0-384)	16 (0-59)	0.03 (0-0.13)
Community + Linkages	137 (0-352)	17 (0-65)	0.03 (0-0.13)
Facilities + Linkages	141 (0-366)	15 (0-55)	0.03 (0-0.13)
Facilities + Linkages + Quality	136 (0-358)	14 (0-55)	0.03 (0-0.12)
Comprehensive	119 (0-321)	13 (0-57)	0.03 (0-0.12)

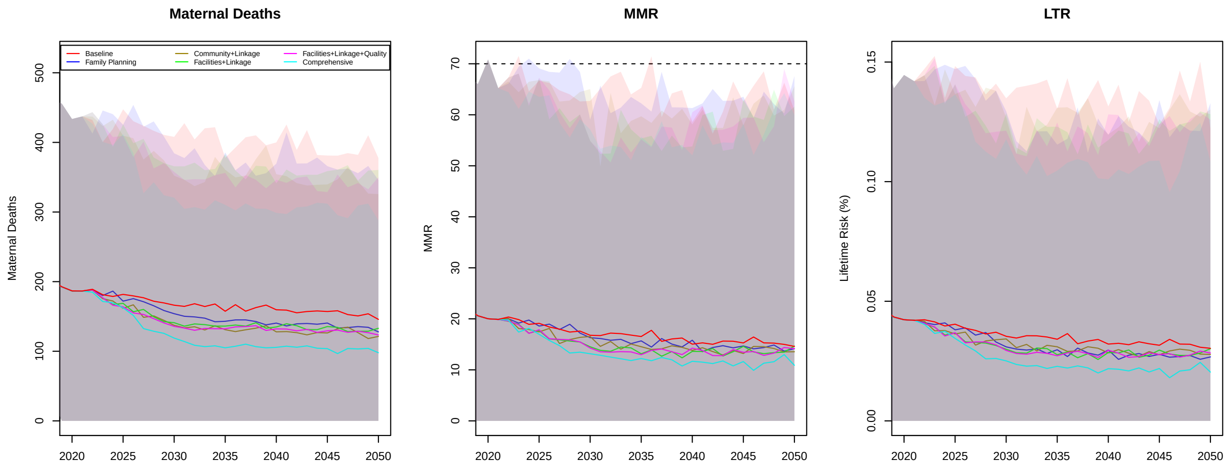

State of Palestine

ISO Code	Region	Area	Income Group
PSE	Western Asia	Asia	Lower middle income

Projected Maternal Indicators in 2030 by Scenario

Scenario	Maternal Deaths	MMR	LTR
**Baseline**	124 (32-243)	21 (0-54)	0.09 (0-0.27)
**Family Planning Interventions**			
Contraception	115 (22-236)	19 (0-58)	0.08 (0-0.24)
Medical abortion	123 (29-265)	20 (0-58)	0.09 (0-0.28)
**Community-Based Interventions**			
ANC	123 (24-250)	19 (0-57)	0.09 (0-0.28)
SBA	124 (31-242)	20 (0-54)	0.09 (0-0.27)
**Facility-Based Interventions**			
Facility births	123 (32-240)	20 (0-53)	0.09 (0-0.26)
nonEmOC services	124 (31-242)	20 (0-55)	0.09 (0-0.28)
bEmOC services	124 (29-255)	20 (0-60)	0.09 (0-0.27)
cEmOC services	115 (26-241)	17 (0-57)	0.08 (0-0.27)
**System-Relevant Interventions**			
Quality of care	116 (31-228)	19 (0-57)	0.09 (0-0.27)
Referral	124 (32-243)	20 (0-54)	0.09 (0-0.27)
Transport	121 (30-254)	19 (0-56)	0.09 (0-0.27)
Targeted transfers	122 (32-246)	20 (0-58)	0.09 (0-0.27)
**Integrated Strategies**			
Family Planning	117 (24-240)	20 (0-62)	0.08 (0-0.26)
Community + Linkages	121 (25-244)	20 (0-55)	0.09 (0-0.26)
Facilities + Linkages	112 (20-236)	16 (0-52)	0.07 (0-0.25)
Facilities + Linkages + Quality	102 (23-221)	15 (0-46)	0.07 (0-0.23)
Comprehensive	99 (19-210)	16 (0-52)	0.07 (0-0.24)

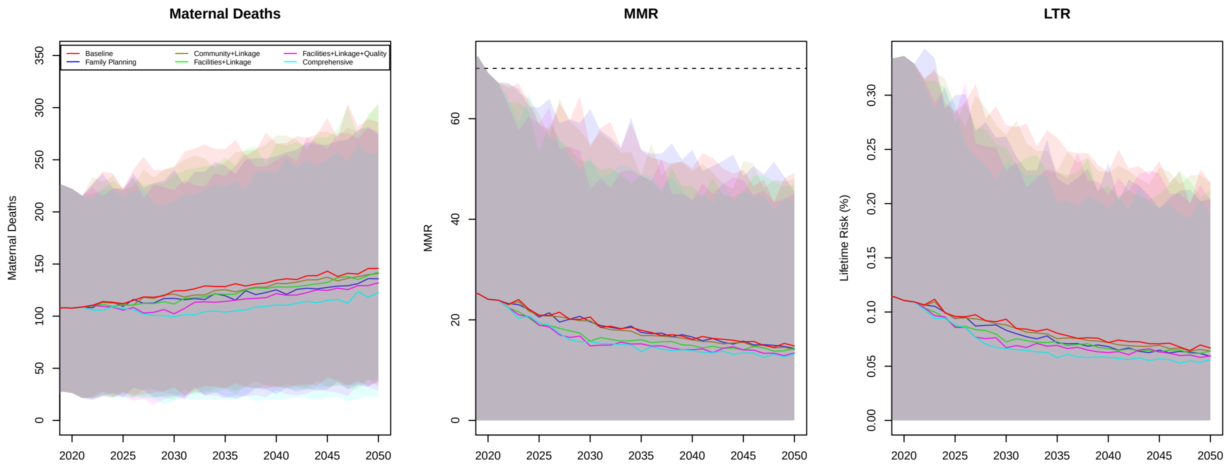

Sudan

ISO Code	Region	Area	Income Group
SDN	Northern Africa	Africa	Lower middle income

Projected Maternal Indicators in 2030 by Scenario

Scenario	Maternal Deaths	MMR	LTR
**Baseline**	2666 (895-5590)	117 (38-264)	0.52 (0.13-1.2)
**Family Planning Interventions**			
Contraception	2633 (931-5821)	116 (40-257)	0.52 (0.14-1.23)
Medical abortion	2677 (884-5665)	118 (38-259)	0.52 (0.13-1.2)
**Community-Based Interventions**			
ANC	2645 (881-5558)	115 (35-255)	0.51 (0.12-1.19)
SBA	2611 (970-5576)	114 (40-257)	0.5 (0.13-1.19)
**Facility-Based Interventions**			
Facility births	2336 (774-5100)	101 (27-231)	0.45 (0.09-1.07)
nonEmOC services	2666 (872-5695)	117 (37-255)	0.52 (0.13-1.18)
bEmOC services	2672 (878-5667)	118 (37-261)	0.52 (0.13-1.23)
cEmOC services	2440 (798-5091)	105 (34-226)	0.46 (0.11-1.04)
**System-Relevant Interventions**			
Quality of care	2595 (836-5608)	114 (32-260)	0.51 (0.11-1.18)
Referral	2679 (910-5738)	118 (36-268)	0.52 (0.13-1.22)
Transport	2549 (866-5635)	114 (36-251)	0.5 (0.13-1.2)
Targeted transfers	2660 (860-5755)	117 (38-262)	0.52 (0.12-1.27)
**Integrated Strategies**			
Family Planning	2637 (931-5551)	116 (41-252)	0.52 (0.14-1.18)
Community + Linkages	2392 (758-5160)	106 (30-240)	0.47 (0.1-1.11)
Facilities + Linkages	1998 (578-4290)	85 (22-199)	0.38 (0.07-0.92)
Facilities + Linkages + Quality	1988 (556-4526)	85 (18-204)	0.38 (0.06-0.93)
Comprehensive	1872 (485-4186)	81 (17-198)	0.36 (0.06-0.9)

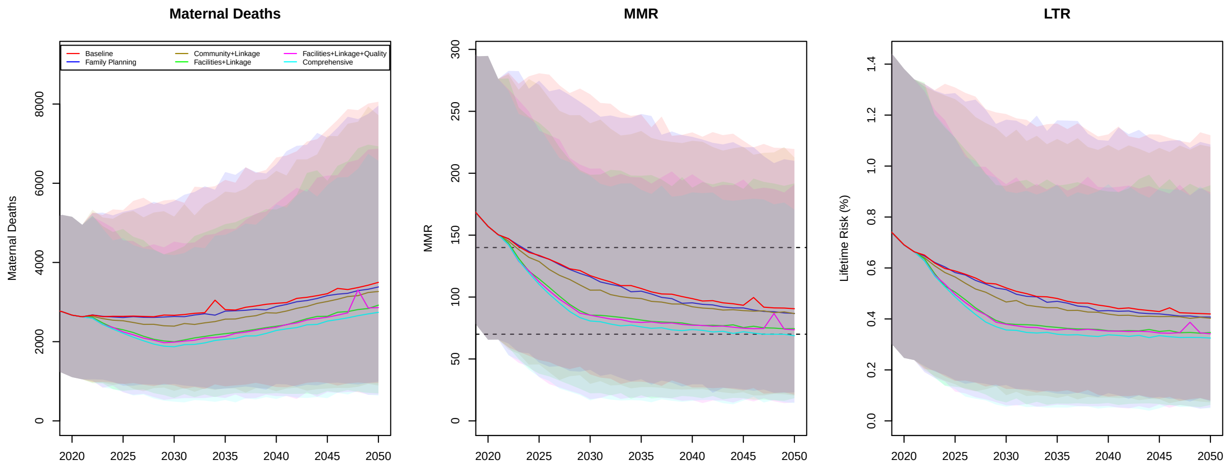

Suriname

ISO Code	Region	Area	Income Group
SUR	South America	Latin America and the Caribbean	Upper middle income

Projected Maternal Indicators in 2030 by Scenario

Scenario	Maternal Deaths	MMR	LTR
**Baseline**	14 (0-38)	109 (0-348)	0.24 (0-0.77)
**Family Planning Interventions**			
Contraception	11 (0-36)	102 (0-347)	0.19 (0-0.68)
Medical abortion	14 (0-38)	109 (0-348)	0.24 (0-0.76)
**Community-Based Interventions**			
ANC	14 (0-42)	113 (0-356)	0.25 (0-0.77)
SBA	13 (0-39)	101 (0-334)	0.22 (0-0.75)
**Facility-Based Interventions**			
Facility births	8 (0-29)	56 (0-249)	0.13 (0-0.54)
nonEmOC services	14 (0-38)	109 (0-329)	0.24 (0-0.73)
bEmOC services	14 (0-40)	110 (0-358)	0.24 (0-0.78)
cEmOC services	14 (0-39)	111 (0-350)	0.25 (0-0.78)
**System-Relevant Interventions**			
Quality of care	14 (0-40)	108 (0-329)	0.24 (0-0.76)
Referral	14 (0-44)	113 (0-343)	0.26 (0-0.76)
Transport	12 (0-37)	102 (0-318)	0.22 (0-0.69)
Targeted transfers	13 (0-37)	105 (0-320)	0.23 (0-0.72)
**Integrated Strategies**			
Family Planning	11 (0-36)	103 (0-347)	0.19 (0-0.68)
Community + Linkages	10 (0-35)	80 (0-284)	0.18 (0-0.65)
Facilities + Linkages	8 (0-32)	65 (0-276)	0.15 (0-0.61)
Facilities + Linkages + Quality	8 (0-33)	62 (0-272)	0.14 (0-0.62)
Comprehensive	6 (0-25)	54 (0-244)	0.1 (0-0.47)

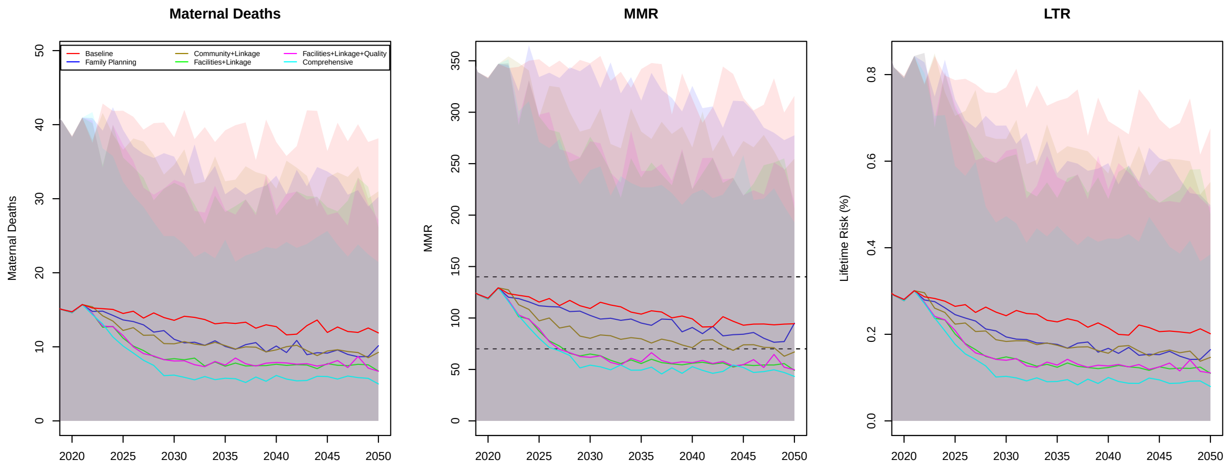

Sweden

ISO Code	Region	Area	Income Group
SWE	Northern Europe	Europe	High income

Projected Maternal Indicators in 2030 by Scenario

Scenario	Maternal Deaths	MMR	LTR
**Baseline**	38 (7-88)	13 (0-43)	0.02 (0-0.08)
**Family Planning Interventions**			
Contraception	38 (7-88)	13 (0-46)	0.02 (0-0.08)
Medical abortion	38 (7-88)	13 (0-42)	0.02 (0-0.08)
**Community-Based Interventions**			
ANC	38 (7-82)	13 (0-40)	0.02 (0-0.08)
SBA	38 (4-88)	13 (0-43)	0.02 (0-0.08)
**Facility-Based Interventions**			
Facility births	38 (7-85)	13 (0-43)	0.02 (0-0.08)
nonEmOC services	38 (7-88)	13 (0-43)	0.02 (0-0.08)
bEmOC services	38 (7-88)	13 (0-43)	0.02 (0-0.08)
cEmOC services	38 (7-85)	13 (0-46)	0.02 (0-0.08)
**System-Relevant Interventions**			
Quality of care	38 (7-88)	12 (0-40)	0.02 (0-0.08)
Referral	38 (4-88)	13 (0-43)	0.02 (0-0.08)
Transport	36 (7-80)	13 (0-43)	0.02 (0-0.08)
Targeted transfers	38 (7-87)	13 (0-43)	0.02 (0-0.08)
**Integrated Strategies**			
Family Planning	38 (7-88)	13 (0-47)	0.02 (0-0.08)
Community + Linkages	36 (4-82)	13 (0-43)	0.02 (0-0.08)
Facilities + Linkages	36 (7-80)	13 (0-43)	0.02 (0-0.08)
Facilities + Linkages + Quality	36 (7-81)	13 (0-44)	0.02 (0-0.08)
Comprehensive	36 (4-87)	12 (0-39)	0.02 (0-0.08)

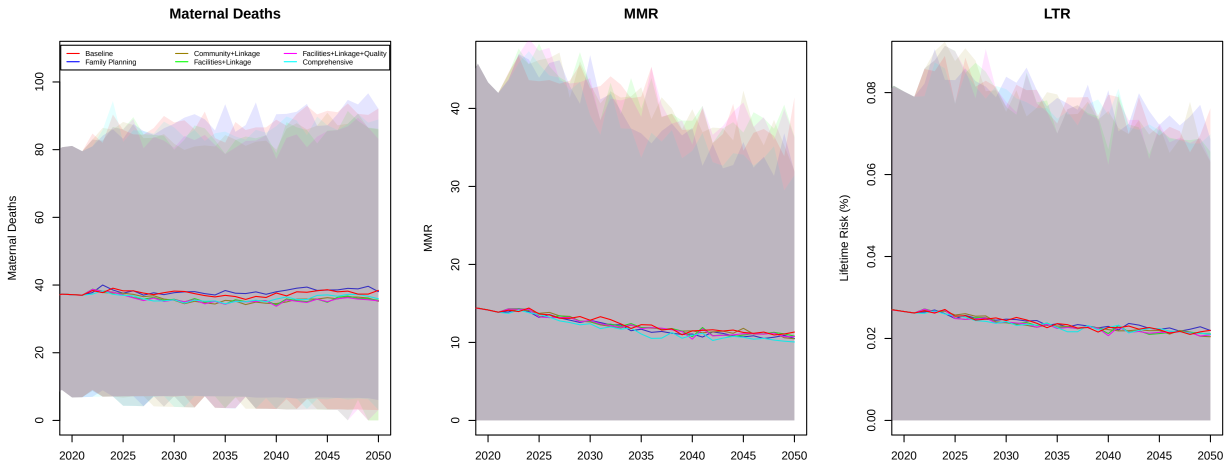

Switzerland

ISO Code	Region	Area	Income Group
CHE	Western Europe	Europe	High income

Projected Maternal Indicators in 2030 by Scenario

Scenario	Maternal Deaths	MMR	LTR
**Baseline**	32 (7-67)	24 (0-55)	0.04 (0-0.08)
**Family Planning Interventions**			
Contraception	32 (7-66)	24 (0-57)	0.03 (0-0.08)
Medical abortion	32 (7-68)	24 (0-57)	0.04 (0-0.09)
**Community-Based Interventions**			
ANC	32 (7-67)	24 (0-57)	0.04 (0-0.09)
SBA	32 (7-66)	24 (0-57)	0.04 (0-0.09)
**Facility-Based Interventions**			
Facility births	32 (7-67)	24 (0-55)	0.04 (0-0.09)
nonEmOC services	32 (7-67)	24 (0-55)	0.04 (0-0.09)
bEmOC services	32 (7-67)	24 (0-55)	0.04 (0-0.08)
cEmOC services	32 (7-66)	23 (0-56)	0.03 (0-0.08)
**System-Relevant Interventions**			
Quality of care	32 (7-67)	24 (0-55)	0.04 (0-0.08)
Referral	32 (7-67)	24 (0-57)	0.04 (0-0.09)
Transport	32 (7-71)	24 (0-61)	0.04 (0-0.09)
Targeted transfers	32 (7-66)	24 (0-55)	0.04 (0-0.08)
**Integrated Strategies**			
Family Planning	32 (7-67)	24 (0-58)	0.03 (0-0.08)
Community + Linkages	32 (7-67)	23 (0-59)	0.04 (0-0.09)
Facilities + Linkages	32 (7-71)	24 (0-61)	0.04 (0-0.09)
Facilities + Linkages + Quality	32 (7-71)	24 (0-61)	0.04 (0-0.09)
Comprehensive	30 (7-65)	23 (0-57)	0.03 (0-0.08)

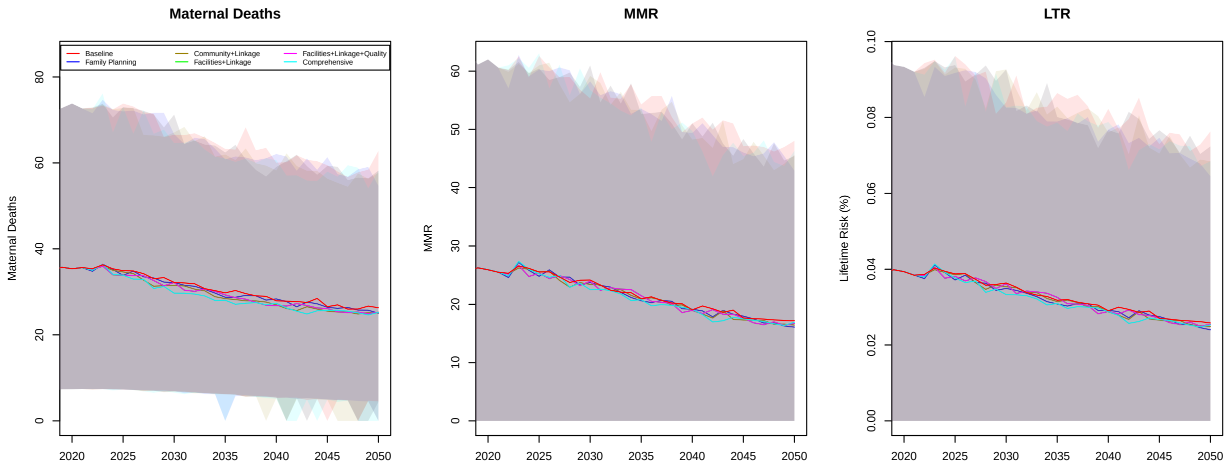

Syrian Arab Republic

ISO Code	Region	Area	Income Group
SYR	Western Asia	Asia	Low income

Projected Maternal Indicators in 2030 by Scenario

Scenario	Maternal Deaths	MMR	LTR
**Baseline**	492 (93-1075)	37 (0-97)	0.08 (0-0.23)
**Family Planning Interventions**			
Contraception	485 (99-1083)	36 (0-103)	0.08 (0-0.24)
Medical abortion	496 (100-1114)	37 (0-100)	0.08 (0-0.23)
**Community-Based Interventions**			
ANC	485 (96-1122)	36 (0-100)	0.08 (0-0.24)
SBA	491 (93-1075)	37 (0-97)	0.08 (0-0.23)
**Facility-Based Interventions**			
Facility births	492 (93-1075)	37 (0-97)	0.08 (0-0.23)
nonEmOC services	492 (100-1065)	37 (0-95)	0.08 (0-0.23)
bEmOC services	493 (102-1063)	38 (0-101)	0.08 (0-0.24)
cEmOC services	471 (87-1032)	34 (0-99)	0.07 (0-0.23)
**System-Relevant Interventions**			
Quality of care	485 (92-1066)	36 (0-101)	0.08 (0-0.24)
Referral	492 (91-1066)	37 (0-96)	0.08 (0-0.23)
Transport	486 (92-1115)	37 (0-102)	0.08 (0-0.23)
Targeted transfers	499 (100-1069)	37 (0-97)	0.08 (0-0.22)
**Integrated Strategies**			
Family Planning	480 (106-1031)	36 (0-102)	0.08 (0-0.24)
Community + Linkages	459 (91-1012)	34 (0-96)	0.07 (0-0.21)
Facilities + Linkages	454 (77-980)	32 (0-94)	0.07 (0-0.2)
Facilities + Linkages + Quality	440 (71-966)	31 (0-90)	0.07 (0-0.2)
Comprehensive	413 (93-925)	28 (0-82)	0.06 (0-0.18)

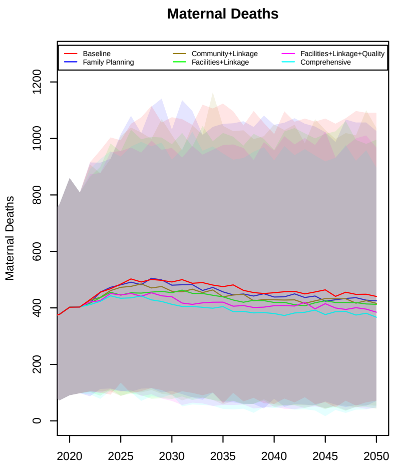

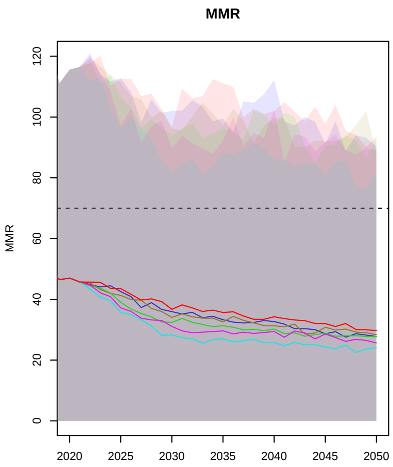

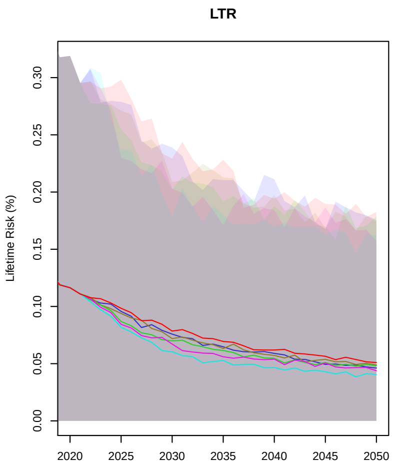

Taiwan

ISO Code	Region	Area	Income Group
TWN	Eastern Asia	Asia	High income

Projected Maternal Indicators in 2030 by Scenario

Scenario	Maternal Deaths	MMR	LTR
**Baseline**	25 (3-56)	12 (0-32)	0.01 (0-0.03)
**Family Planning Interventions**			
Contraception	25 (3-58)	11 (0-34)	0.01 (0-0.03)
Medical abortion	25 (4-62)	12 (0-33)	0.01 (0-0.03)
**Community-Based Interventions**			
ANC	25 (3-58)	12 (0-31)	0.01 (0-0.03)
SBA	25 (3-55)	12 (0-31)	0.01 (0-0.03)
**Facility-Based Interventions**			
Facility births	25 (5-53)	12 (0-29)	0.01 (0-0.03)
nonEmOC services	25 (5-56)	12 (1-32)	0.01 (0-0.03)
bEmOC services	25 (3-56)	12 (0-32)	0.01 (0-0.03)
cEmOC services	24 (3-56)	11 (0-31)	0.01 (0-0.03)
**System-Relevant Interventions**			
Quality of care	23 (3-59)	11 (0-31)	0.01 (0-0.03)
Referral	25 (3-58)	12 (0-32)	0.01 (0-0.03)
Transport	25 (3-59)	11 (0-31)	0.01 (0-0.03)
Targeted transfers	25 (3-57)	12 (0-32)	0.01 (0-0.03)
**Integrated Strategies**			
Family Planning	25 (3-63)	11 (0-36)	0.01 (0-0.04)
Community + Linkages	25 (3-59)	11 (0-30)	0.01 (0-0.03)
Facilities + Linkages	24 (2-56)	11 (0-28)	0.01 (0-0.03)
Facilities + Linkages + Quality	22 (2-54)	10 (0-26)	0.01 (0-0.03)
Comprehensive	22 (3-52)	10 (0-27)	0.01 (0-0.03)

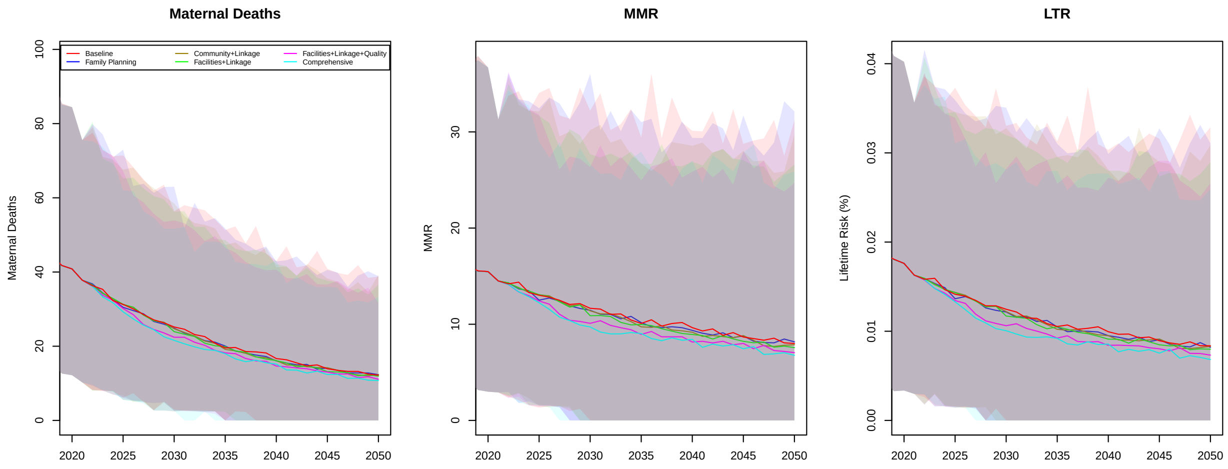

Tajikistan

ISO Code	Region	Area	Income Group
TJK	Central Asia	Asia	Low income

Projected Maternal Indicators in 2030 by Scenario

Scenario	Maternal Deaths	MMR	LTR
**Baseline**	670 (157-1615)	213 (28-573)	0.62 (0.07-1.67)
**Family Planning Interventions**			
Contraception	559 (138-1210)	178 (26-434)	0.5 (0.06-1.19)
Medical abortion	616 (133-1332)	187 (29-441)	0.55 (0.07-1.35)
**Community-Based Interventions**			
ANC	651 (157-1432)	204 (26-496)	0.6 (0.07-1.49)
SBA	640 (157-1579)	202 (30-570)	0.59 (0.09-1.61)
**Facility-Based Interventions**			
Facility births	422 (32-1198)	116 (0-410)	0.32 (0-1.21)
nonEmOC services	670 (142-1680)	213 (28-573)	0.62 (0.08-1.76)
bEmOC services	667 (140-1594)	210 (30-579)	0.61 (0.08-1.66)
cEmOC services	695 (158-1665)	218 (34-598)	0.64 (0.1-1.73)
**System-Relevant Interventions**			
Quality of care	660 (159-1596)	211 (30-583)	0.62 (0.09-1.69)
Referral	673 (154-1610)	213 (31-582)	0.63 (0.09-1.64)
Transport	617 (144-1520)	199 (26-548)	0.58 (0.06-1.63)
Targeted transfers	678 (155-1605)	214 (28-559)	0.63 (0.08-1.67)
**Integrated Strategies**			
Family Planning	542 (118-1246)	169 (0-434)	0.48 (0-1.22)
Community + Linkages	538 (109-1251)	167 (21-461)	0.49 (0.06-1.25)
Facilities + Linkages	386 (0-1196)	110 (0-411)	0.31 (0-1.28)
Facilities + Linkages + Quality	370 (0-1250)	106 (0-412)	0.3 (0-1.27)
Comprehensive	225 (0-555)	52 (0-175)	0.14 (0-0.48)

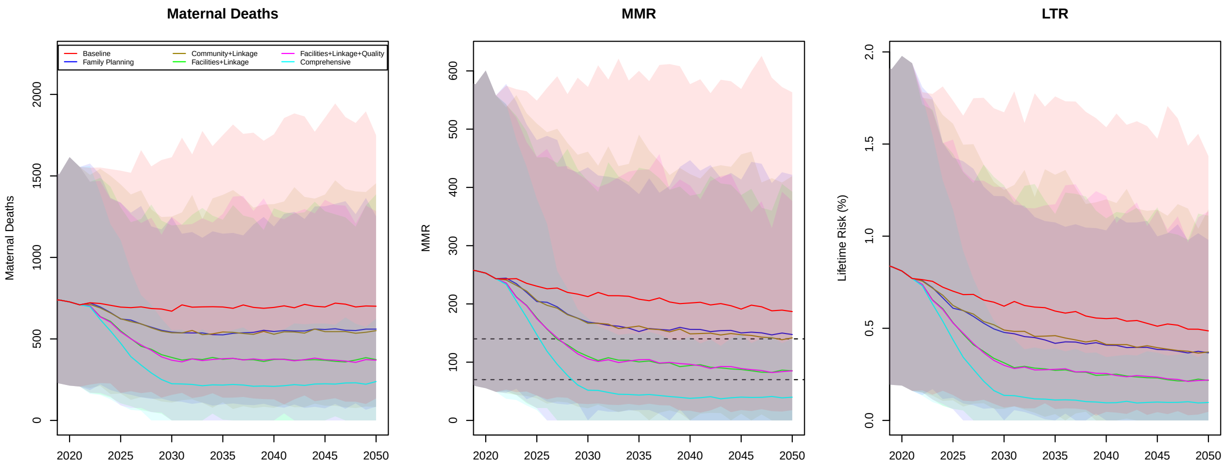

Thailand

ISO Code	Region	Area	Income Group
THA	South-Eastern Asia	Asia	Upper middle income

Projected Maternal Indicators in 2030 by Scenario

Scenario	Maternal Deaths	MMR	LTR
**Baseline**	206 (0-556)	16 (0-58)	0.03 (0-0.09)
**Family Planning Interventions**			
Contraception	207 (0-541)	15 (0-52)	0.02 (0-0.08)
Medical abortion	212 (0-568)	16 (0-58)	0.02 (0-0.09)
**Community-Based Interventions**			
ANC	201 (0-534)	15 (0-52)	0.02 (0-0.08)
SBA	205 (0-549)	16 (0-55)	0.03 (0-0.09)
**Facility-Based Interventions**			
Facility births	208 (0-568)	16 (0-58)	0.03 (0-0.09)
nonEmOC services	206 (0-538)	16 (0-55)	0.02 (0-0.09)
bEmOC services	203 (0-538)	15 (0-55)	0.02 (0-0.09)
cEmOC services	207 (0-566)	16 (0-56)	0.03 (0-0.09)
**System-Relevant Interventions**			
Quality of care	208 (0-566)	16 (0-56)	0.03 (0-0.09)
Referral	207 (0-562)	16 (0-58)	0.03 (0-0.09)
Transport	197 (0-519)	15 (0-51)	0.02 (0-0.08)
Targeted transfers	208 (0-562)	16 (0-59)	0.03 (0-0.09)
**Integrated Strategies**			
Family Planning	201 (0-530)	15 (0-49)	0.02 (0-0.08)
Community + Linkages	192 (0-538)	14 (0-48)	0.02 (0-0.08)
Facilities + Linkages	194 (0-518)	14 (0-52)	0.02 (0-0.08)
Facilities + Linkages + Quality	187 (0-513)	14 (0-46)	0.02 (0-0.08)
Comprehensive	181 (0-503)	13 (0-49)	0.02 (0-0.08)

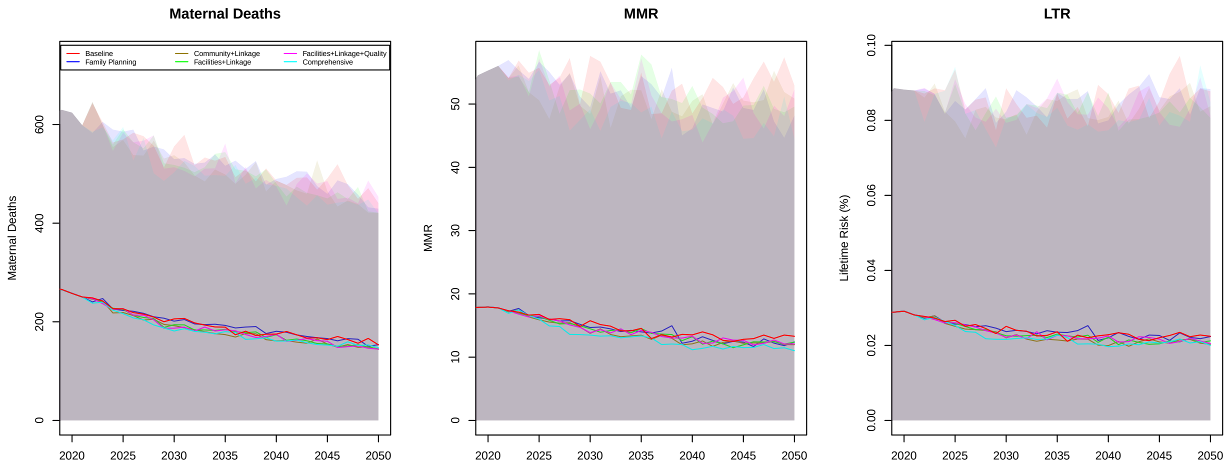

Timor-Leste

ISO Code	Region	Area	Income Group
TLS	South-Eastern Asia	Asia	Lower middle income

Projected Maternal Indicators in 2030 by Scenario

Scenario	Maternal Deaths	MMR	LTR
**Baseline**	128 (48-223)	209 (71-384)	0.88 (0.3-1.57)
**Family Planning Interventions**			
Contraception	108 (37-197)	176 (51-326)	0.72 (0.18-1.37)
Medical abortion	114 (41-210)	185 (56-366)	0.77 (0.24-1.45)
**Community-Based Interventions**			
ANC	123 (48-212)	202 (66-368)	0.84 (0.28-1.53)
SBA	106 (38-189)	170 (46-319)	0.72 (0.21-1.37)
**Facility-Based Interventions**			
Facility births	50 (9-114)	67 (4-169)	0.31 (0.02-0.81)
nonEmOC services	126 (46-219)	207 (68-389)	0.87 (0.3-1.59)
bEmOC services	127 (45-221)	210 (72-381)	0.88 (0.29-1.59)
cEmOC services	124 (45-212)	205 (68-374)	0.86 (0.29-1.52)
**System-Relevant Interventions**			
Quality of care	121 (46-213)	199 (65-369)	0.84 (0.28-1.54)
Referral	127 (47-225)	209 (66-378)	0.88 (0.28-1.61)
Transport	111 (41-196)	180 (55-336)	0.76 (0.24-1.41)
Targeted transfers	127 (47-221)	208 (72-384)	0.88 (0.3-1.56)
**Integrated Strategies**			
Family Planning	105 (32-197)	170 (45-330)	0.68 (0.18-1.3)
Community + Linkages	91 (34-167)	145 (46-280)	0.62 (0.19-1.21)
Facilities + Linkages	45 (8-104)	60 (6-160)	0.28 (0.01-0.73)
Facilities + Linkages + Quality	42 (6-96)	55 (0-162)	0.25 (0-0.7)
Comprehensive	24 (0-64)	23 (0-76)	0.1 (0-0.34)

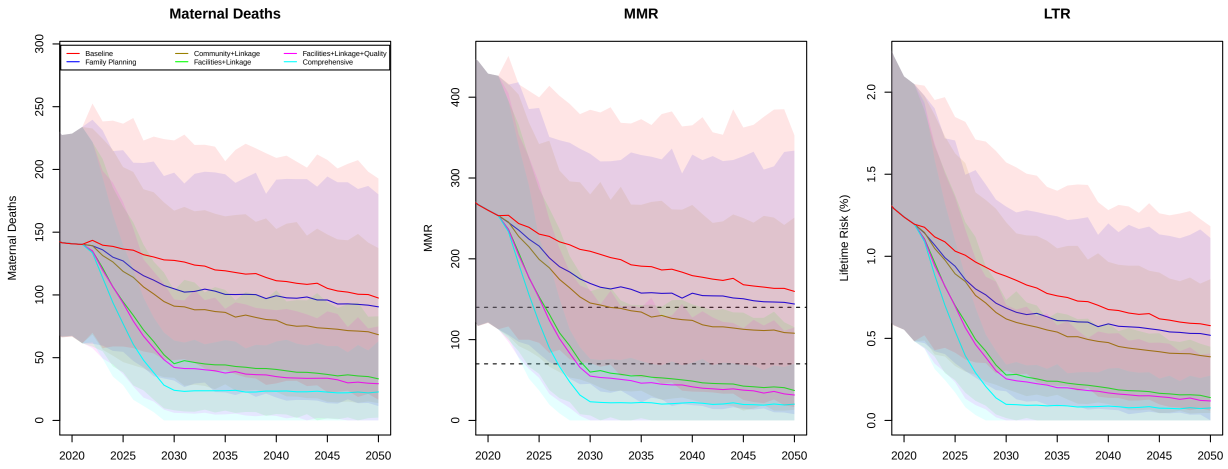

Togo

ISO Code	Region	Area	Income Group
TGO	Western Africa	Africa	Low income

Projected Maternal Indicators in 2030 by Scenario

Scenario	Maternal Deaths	MMR	LTR
**Baseline**	1516 (627-2459)	329 (153-537)	1.54 (0.62-2.55)
**Family Planning Interventions**			
Contraception	1468 (538-2375)	322 (137-511)	1.49 (0.52-2.47)
Medical abortion	1491 (641-2429)	323 (159-506)	1.51 (0.59-2.54)
**Community-Based Interventions**			
ANC	1476 (613-2449)	319 (135-511)	1.49 (0.58-2.48)
SBA	1377 (576-2196)	296 (135-479)	1.4 (0.53-2.29)
**Facility-Based Interventions**			
Facility births	1027 (365-1774)	213 (72-382)	1.01 (0.28-1.87)
nonEmOC services	1484 (557-2345)	320 (132-514)	1.5 (0.49-2.49)
bEmOC services	1475 (631-2357)	320 (142-510)	1.49 (0.58-2.47)
cEmOC services	1474 (544-2363)	318 (133-513)	1.49 (0.5-2.45)
**System-Relevant Interventions**			
Quality of care	1001 (292-1728)	204 (62-350)	0.96 (0.22-1.73)
Referral	1501 (566-2421)	325 (137-520)	1.53 (0.57-2.55)
Transport	1392 (546-2259)	301 (136-495)	1.41 (0.52-2.42)
Targeted transfers	1479 (543-2420)	320 (125-516)	1.51 (0.49-2.51)
**Integrated Strategies**			
Family Planning	1462 (540-2320)	320 (140-503)	1.48 (0.52-2.45)
Community + Linkages	1223 (464-2064)	260 (106-438)	1.21 (0.43-2.1)
Facilities + Linkages	957 (388-1746)	198 (74-381)	0.93 (0.29-1.79)
Facilities + Linkages + Quality	320 (47-707)	46 (0-122)	0.2 (0-0.6)
Comprehensive	300 (46-711)	41 (0-119)	0.18 (0-0.56)

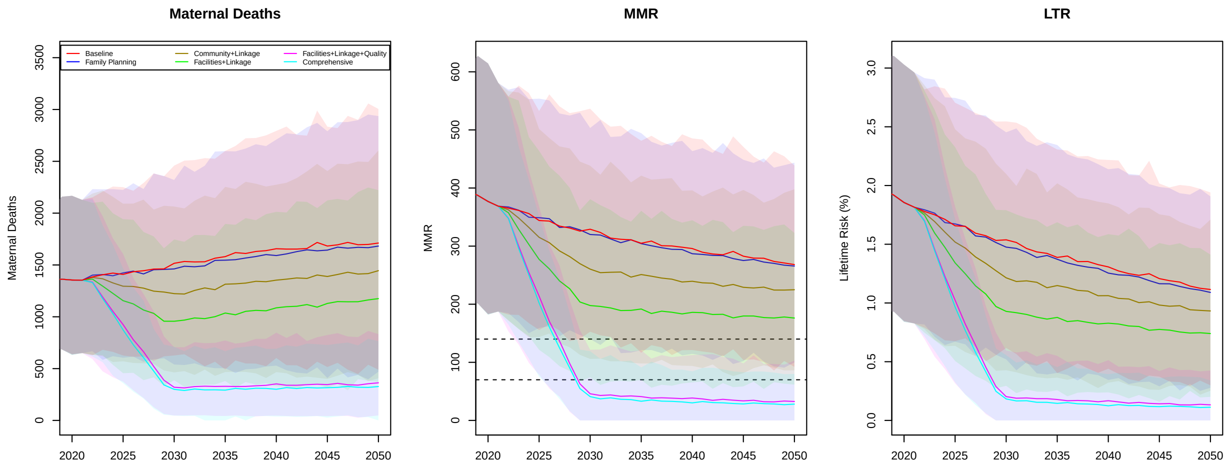

Tonga

ISO Code	Region	Area	Income Group
TON	Polynesia	Oceania	Upper middle income

Projected Maternal Indicators in 2030 by Scenario

Scenario	Maternal Deaths	MMR	LTR
**Baseline**	5 (0-11)	164 (0-389)	0.57 (0-1.32)
**Family Planning Interventions**			
Contraception	4 (0-10)	154 (0-359)	0.49 (0-1.2)
Medical abortion	5 (0-11)	164 (0-391)	0.57 (0-1.3)
**Community-Based Interventions**			
ANC	5 (0-12)	163 (0-396)	0.57 (0-1.37)
SBA	5 (0-11)	165 (0-389)	0.57 (0-1.34)
**Facility-Based Interventions**			
Facility births	5 (0-11)	167 (0-396)	0.58 (0-1.35)
nonEmOC services	5 (0-11)	164 (0-389)	0.57 (0-1.3)
bEmOC services	5 (0-11)	163 (0-381)	0.56 (0-1.25)
cEmOC services	5 (0-11)	165 (0-389)	0.58 (0-1.35)
**System-Relevant Interventions**			
Quality of care	5 (0-11)	165 (0-407)	0.57 (0-1.37)
Referral	5 (0-11)	164 (0-389)	0.57 (0-1.32)
Transport	5 (0-11)	166 (0-390)	0.58 (0-1.33)
Targeted transfers	5 (0-11)	165 (0-395)	0.57 (0-1.36)
**Integrated Strategies**			
Family Planning	4 (0-10)	154 (0-378)	0.49 (0-1.22)
Community + Linkages	5 (0-11)	166 (0-396)	0.57 (0-1.35)
Facilities + Linkages	5 (0-11)	163 (0-390)	0.56 (0-1.33)
Facilities + Linkages + Quality	5 (0-11)	168 (0-392)	0.58 (0-1.36)
Comprehensive	4 (0-10)	151 (0-372)	0.49 (0-1.19)

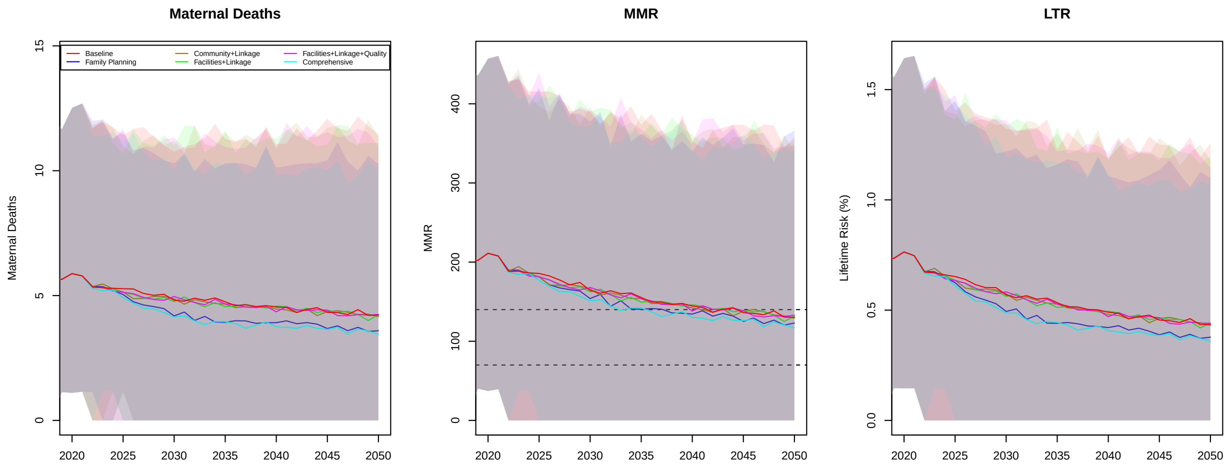

Trinidad and Tobago

ISO Code	Region	Area	Income Group
TTO	Caribbean	Latin America and the Caribbean	High income

Projected Maternal Indicators in 2030 by Scenario

Scenario	Maternal Deaths	MMR	LTR
**Baseline**	23 (0-76)	133 (0-527)	0.2 (0-0.69)
**Family Planning Interventions**			
Contraception	19 (0-55)	113 (0-379)	0.16 (0-0.49)
Medical abortion	19 (0-48)	109 (0-296)	0.17 (0-0.43)
**Community-Based Interventions**			
ANC	23 (0-74)	134 (0-524)	0.2 (0-0.68)
SBA	23 (0-76)	134 (0-530)	0.2 (0-0.69)
**Facility-Based Interventions**			
Facility births	23 (0-75)	133 (0-527)	0.2 (0-0.69)
nonEmOC services	23 (0-76)	133 (0-527)	0.2 (0-0.69)
bEmOC services	23 (0-76)	133 (0-527)	0.2 (0-0.69)
cEmOC services	23 (0-73)	134 (0-521)	0.2 (0-0.68)
**System-Relevant Interventions**			
Quality of care	23 (0-75)	133 (0-527)	0.2 (0-0.68)
Referral	23 (0-75)	133 (0-515)	0.2 (0-0.69)
Transport	23 (0-74)	133 (0-513)	0.2 (0-0.68)
Targeted transfers	23 (0-76)	133 (0-527)	0.2 (0-0.69)
**Integrated Strategies**			
Family Planning	16 (0-39)	93 (0-235)	0.13 (0-0.35)
Community + Linkages	22 (0-75)	130 (0-509)	0.2 (0-0.69)
Facilities + Linkages	22 (0-70)	132 (0-508)	0.2 (0-0.63)
Facilities + Linkages + Quality	22 (0-70)	132 (0-508)	0.2 (0-0.63)
Comprehensive	15 (0-39)	92 (0-246)	0.13 (0-0.36)

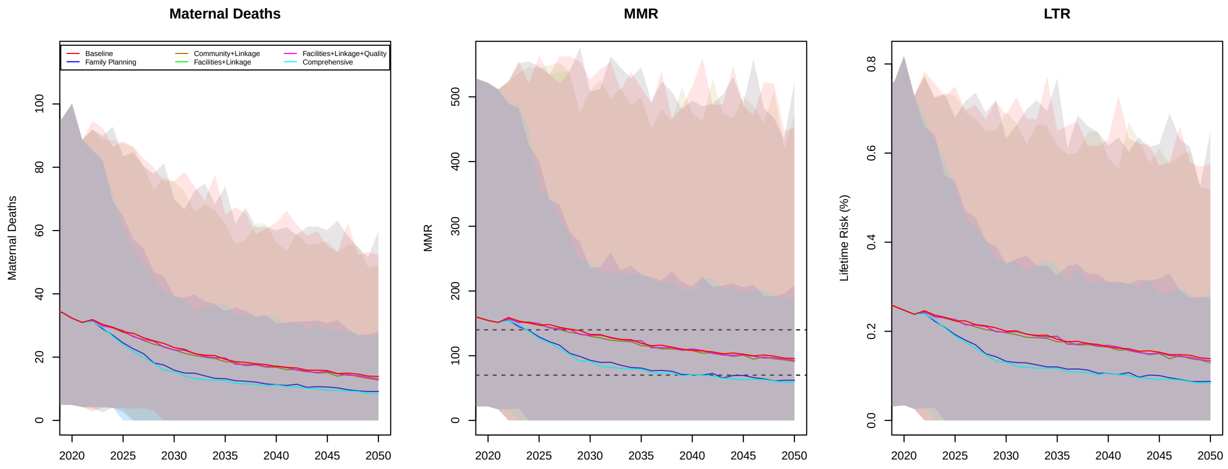

Tunisia

ISO Code	Region	Area	Income Group
TUN	Northern Africa	Africa	Lower middle income

Projected Maternal Indicators in 2030 by Scenario

Scenario	Maternal Deaths	MMR	LTR
**Baseline**	113 (31-264)	35 (5-107)	0.08 (0.01-0.25)
**Family Planning Interventions**			
Contraception	122 (36-277)	33 (4-95)	0.08 (0.01-0.24)
Medical abortion	114 (32-263)	35 (5-102)	0.08 (0.01-0.23)
**Community-Based Interventions**			
ANC	111 (28-248)	36 (5-105)	0.08 (0.01-0.23)
SBA	108 (28-232)	33 (5-97)	0.08 (0.01-0.19)
**Facility-Based Interventions**			
Facility births	88 (20-198)	23 (1-64)	0.05 (0-0.14)
nonEmOC services	113 (28-264)	35 (5-110)	0.08 (0.01-0.23)
bEmOC services	113 (32-254)	35 (6-97)	0.08 (0.01-0.23)
cEmOC services	110 (29-250)	34 (5-100)	0.08 (0.01-0.21)
**System-Relevant Interventions**			
Quality of care	112 (32-259)	35 (6-102)	0.08 (0.01-0.23)
Referral	114 (31-266)	35 (5-101)	0.08 (0.01-0.23)
Transport	106 (25-238)	33 (5-92)	0.08 (0.01-0.19)
Targeted transfers	113 (30-260)	36 (5-106)	0.08 (0.01-0.25)
**Integrated Strategies**			
Family Planning	123 (36-272)	34 (4-91)	0.08 (0.01-0.24)
Community + Linkages	94 (24-210)	29 (5-81)	0.06 (0.01-0.18)
Facilities + Linkages	79 (13-173)	21 (0-57)	0.05 (0-0.13)
Facilities + Linkages + Quality	78 (14-181)	21 (0-56)	0.05 (0-0.13)
Comprehensive	83 (13-180)	19 (0-51)	0.05 (0-0.13)

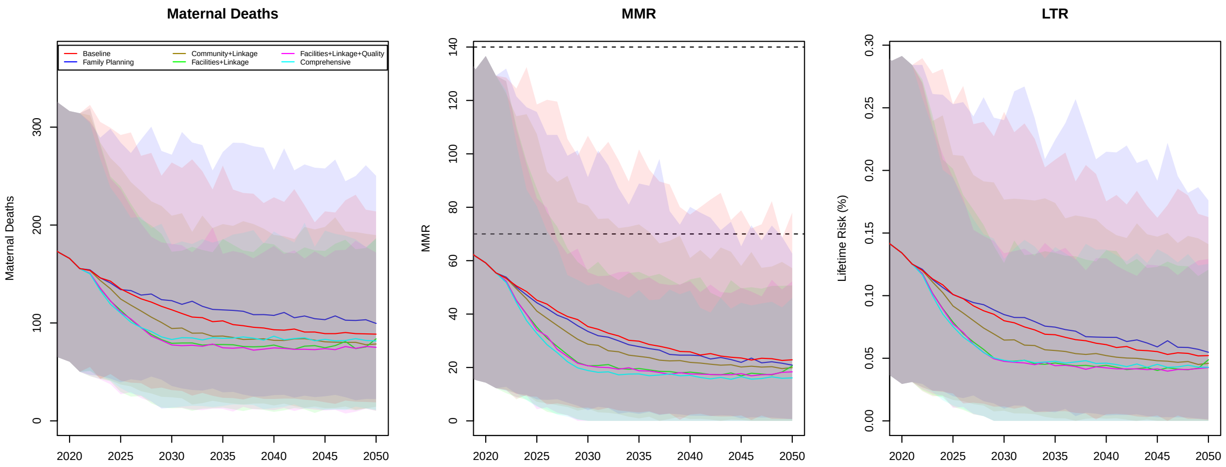

Türkiye

ISO Code	Region	Area	Income Group
TUR	Western Asia	Asia	Upper middle income

Projected Maternal Indicators in 2030 by Scenario

Scenario	Maternal Deaths	MMR	LTR
**Baseline**	414 (74-939)	15 (0-43)	0.03 (0-0.1)
**Family Planning Interventions**			
Contraception	410 (68-956)	15 (0-42)	0.03 (0-0.11)
Medical abortion	398 (33-900)	15 (0-42)	0.03 (0-0.1)
**Community-Based Interventions**			
ANC	417 (74-905)	15 (0-42)	0.03 (0-0.1)
SBA	406 (70-984)	15 (0-41)	0.03 (0-0.1)
**Facility-Based Interventions**			
Facility births	395 (56-945)	14 (0-41)	0.03 (0-0.1)
nonEmOC services	415 (75-925)	15 (0-43)	0.03 (0-0.1)
bEmOC services	412 (73-949)	15 (0-43)	0.03 (0-0.1)
cEmOC services	414 (68-963)	15 (0-44)	0.03 (0-0.1)
**System-Relevant Interventions**			
Quality of care	367 (34-865)	13 (0-40)	0.03 (0-0.09)
Referral	411 (75-968)	15 (0-41)	0.03 (0-0.1)
Transport	412 (35-948)	15 (0-44)	0.03 (0-0.1)
Targeted transfers	416 (77-949)	15 (0-45)	0.03 (0-0.1)
**Integrated Strategies**			
Family Planning	401 (62-860)	15 (0-41)	0.03 (0-0.09)
Community + Linkages	400 (57-927)	15 (0-42)	0.03 (0-0.1)
Facilities + Linkages	394 (64-897)	15 (0-42)	0.03 (0-0.1)
Facilities + Linkages + Quality	346 (35-821)	12 (0-38)	0.03 (0-0.09)
Comprehensive	344 (29-825)	11 (0-38)	0.03 (0-0.09)

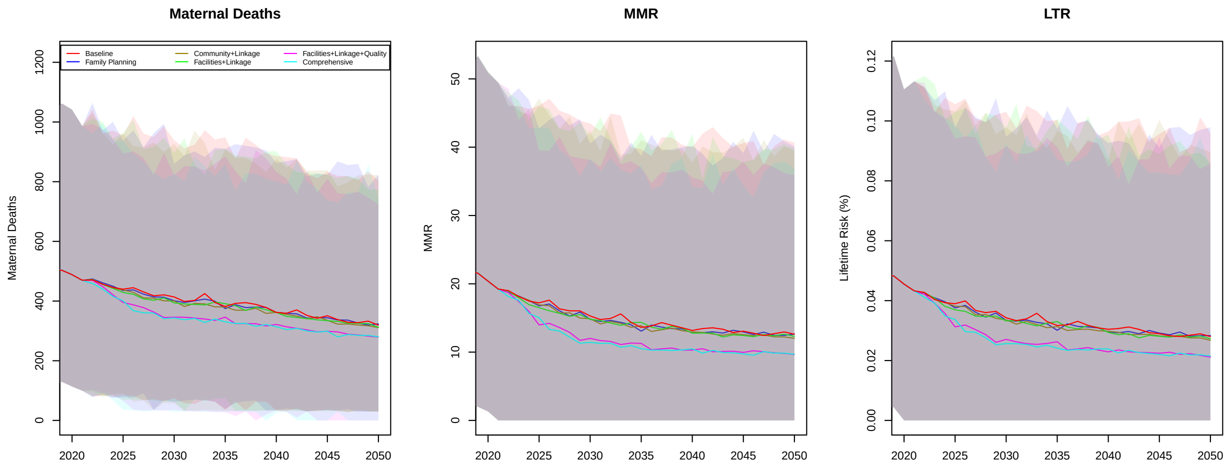

Turkmenistan

ISO Code	Region	Area	Income Group
TKM	Central Asia	Asia	Upper middle income

Projected Maternal Indicators in 2030 by Scenario

Scenario	Maternal Deaths	MMR	LTR
**Baseline**	68 (0-240)	38 (0-154)	0.1 (0-0.42)
**Family Planning Interventions**			
Contraception	60 (0-209)	37 (0-142)	0.09 (0-0.35)
Medical abortion	71 (0-225)	40 (0-145)	0.1 (0-0.37)
**Community-Based Interventions**			
ANC	65 (0-226)	37 (0-144)	0.1 (0-0.39)
SBA	68 (0-240)	38 (0-154)	0.1 (0-0.41)
**Facility-Based Interventions**			
Facility births	66 (0-239)	37 (0-146)	0.09 (0-0.38)
nonEmOC services	68 (0-240)	38 (0-154)	0.1 (0-0.42)
bEmOC services	68 (0-240)	38 (0-153)	0.1 (0-0.41)
cEmOC services	66 (0-230)	37 (0-143)	0.09 (0-0.37)
**System-Relevant Interventions**			
Quality of care	67 (0-231)	37 (0-146)	0.09 (0-0.38)
Referral	69 (0-239)	38 (0-153)	0.1 (0-0.41)
Transport	64 (0-227)	37 (0-144)	0.1 (0-0.39)
Targeted transfers	68 (0-242)	38 (0-154)	0.1 (0-0.42)
**Integrated Strategies**			
Family Planning	58 (0-196)	35 (0-123)	0.08 (0-0.32)
Community + Linkages	63 (0-239)	36 (0-153)	0.09 (0-0.4)
Facilities + Linkages	61 (0-233)	36 (0-147)	0.09 (0-0.44)
Facilities + Linkages + Quality	60 (0-231)	36 (0-144)	0.09 (0-0.43)
Comprehensive	47 (0-189)	28 (0-125)	0.07 (0-0.3)

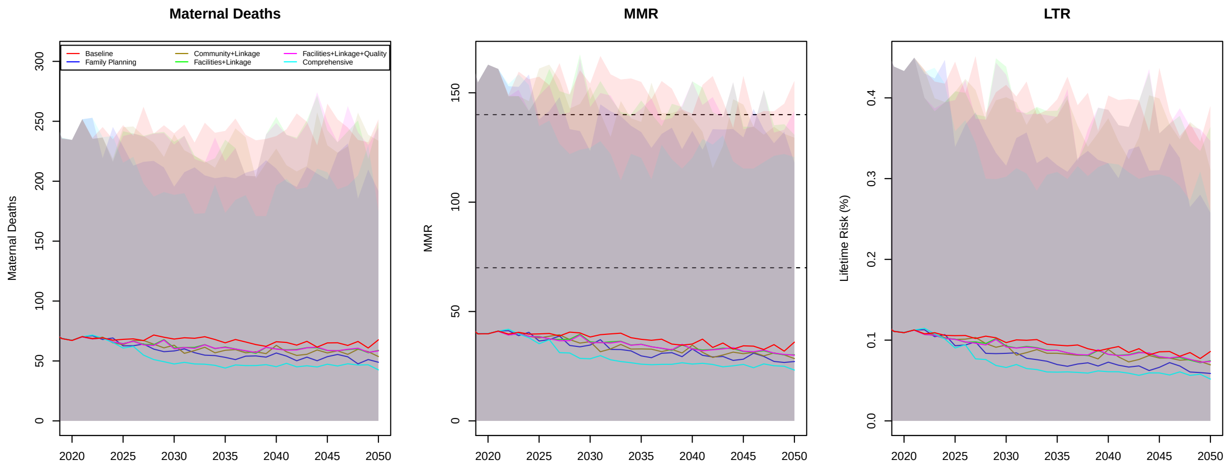

Tuvalu

ISO Code	Region	Area	Income Group
TUV	Polynesia	Oceania	Upper middle income

Projected Maternal Indicators in 2030 by Scenario

Scenario	Maternal Deaths	MMR	LTR
**Baseline**	0 (0-2)	187 (0-1179)	0.41 (0-2.12)
**Family Planning Interventions**			
Contraception	0 (0-2)	173 (0-1248)	0.31 (0-2.02)
Medical abortion	0 (0-2)	184 (0-1178)	0.4 (0-2.12)
**Community-Based Interventions**			
ANC	0 (0-2)	179 (0-1102)	0.39 (0-2.03)
SBA	0 (0-2)	187 (0-1179)	0.4 (0-2.12)
**Facility-Based Interventions**			
Facility births	0 (0-2)	187 (0-1179)	0.41 (0-2.12)
nonEmOC services	0 (0-2)	184 (0-1169)	0.4 (0-2.12)
bEmOC services	0 (0-2)	187 (0-1179)	0.4 (0-2.12)
cEmOC services	0 (0-2)	187 (0-1116)	0.39 (0-2.04)
**System-Relevant Interventions**			
Quality of care	0 (0-2)	186 (0-1178)	0.39 (0-2.05)
Referral	0 (0-2)	185 (0-1179)	0.4 (0-2.07)
Transport	0 (0-2)	199 (0-1184)	0.43 (0-2.23)
Targeted transfers	0 (0-2)	182 (0-1179)	0.39 (0-2.05)
**Integrated Strategies**			
Family Planning	0 (0-2)	175 (0-1305)	0.32 (0-2.05)
Community + Linkages	0 (0-2)	195 (0-1220)	0.42 (0-2.18)
Facilities + Linkages	0 (0-2)	177 (0-1169)	0.38 (0-2.15)
Facilities + Linkages + Quality	0 (0-2)	191 (0-1182)	0.4 (0-2.14)
Comprehensive	0 (0-2)	180 (0-1221)	0.32 (0-1.97)

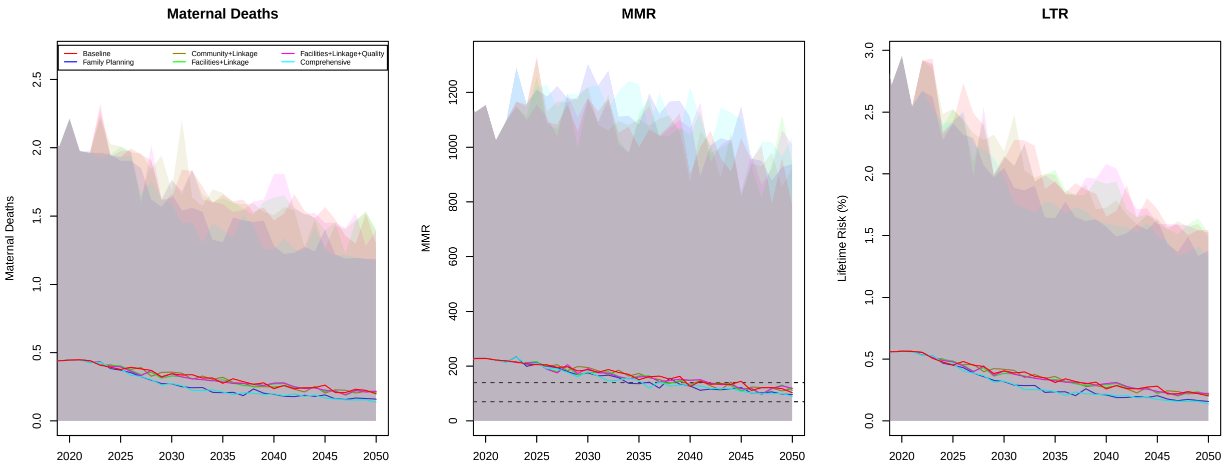

Uganda

ISO Code	Region	Area	Income Group
UGA	Eastern Africa	Africa	Low income

Projected Maternal Indicators in 2030 by Scenario

Scenario	Maternal Deaths	MMR	LTR
**Baseline**	5693 (3306-9267)	206 (116-372)	0.95 (0.54-1.61)
**Family Planning Interventions**			
Contraception	5358 (3062-8512)	194 (110-331)	0.88 (0.49-1.4)
Medical abortion	5226 (2787-8422)	184 (101-317)	0.85 (0.44-1.42)
**Community-Based Interventions**			
ANC	5604 (3324-9508)	203 (115-370)	0.93 (0.52-1.6)
SBA	5507 (3145-8941)	198 (109-361)	0.92 (0.49-1.61)
**Facility-Based Interventions**			
Facility births	3452 (1633-6714)	112 (35-261)	0.49 (0.15-1.08)
nonEmOC services	5679 (3417-9421)	205 (117-376)	0.94 (0.53-1.62)
bEmOC services	5658 (3401-9146)	204 (115-373)	0.94 (0.53-1.6)
cEmOC services	5562 (3329-9247)	200 (112-357)	0.93 (0.52-1.62)
**System-Relevant Interventions**			
Quality of care	4772 (2738-8222)	166 (91-311)	0.78 (0.43-1.35)
Referral	5496 (3185-8885)	198 (108-358)	0.91 (0.5-1.63)
Transport	4968 (2790-8474)	179 (94-342)	0.81 (0.42-1.47)
Targeted transfers	5645 (3312-9333)	204 (114-368)	0.94 (0.53-1.61)
**Integrated Strategies**			
Family Planning	5101 (2643-8401)	183 (102-309)	0.83 (0.42-1.39)
Community + Linkages	4287 (2455-7469)	151 (77-301)	0.68 (0.36-1.24)
Facilities + Linkages	3075 (1349-5992)	100 (30-245)	0.44 (0.12-0.99)
Facilities + Linkages + Quality	2108 (1050-4399)	60 (19-180)	0.26 (0.07-0.73)
Comprehensive	1527 (789-2507)	35 (14-62)	0.15 (0.05-0.29)

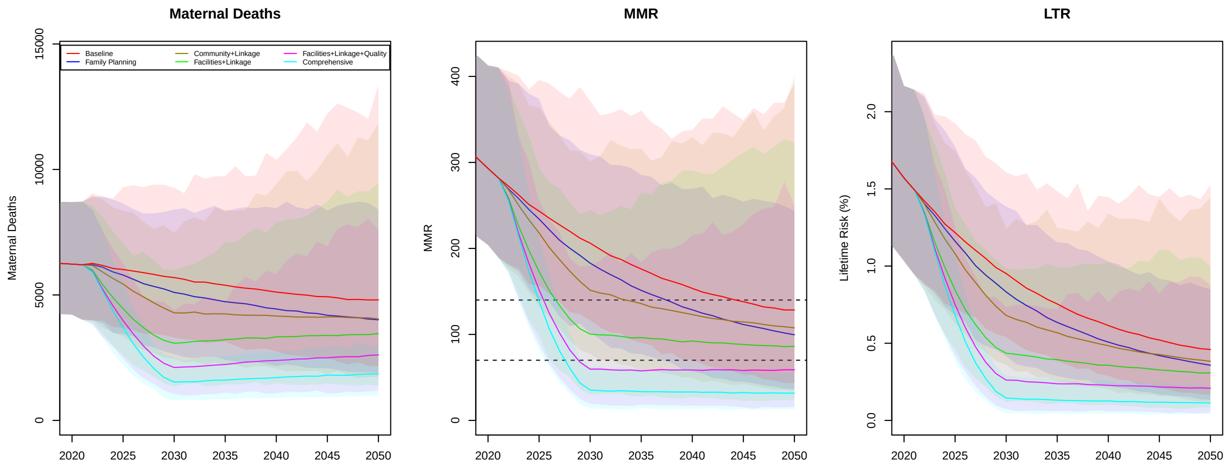

Ukraine

ISO Code	Region	Area	Income Group
UKR	Eastern Europe	Europe	Lower middle income

Projected Maternal Indicators in 2030 by Scenario

Scenario	Maternal Deaths	MMR	LTR
**Baseline**	251 (79-524)	42 (5-112)	0.05 (0-0.14)
**Family Planning Interventions**			
Contraception	223 (69-462)	36 (0-104)	0.04 (0-0.12)
Medical abortion	255 (84-521)	42 (4-111)	0.05 (0-0.13)
**Community-Based Interventions**			
ANC	249 (79-522)	41 (5-112)	0.05 (0-0.13)
SBA	251 (79-524)	42 (5-112)	0.05 (0-0.14)
**Facility-Based Interventions**			
Facility births	252 (82-524)	43 (5-113)	0.05 (0-0.14)
nonEmOC services	252 (79-524)	42 (5-112)	0.05 (0-0.14)
bEmOC services	251 (78-518)	41 (5-103)	0.05 (0-0.13)
cEmOC services	245 (74-506)	39 (0-109)	0.05 (0-0.13)
**System-Relevant Interventions**			
Quality of care	254 (75-525)	42 (5-117)	0.05 (0-0.14)
Referral	252 (79-524)	42 (5-113)	0.05 (0-0.14)
Transport	214 (54-448)	38 (4-98)	0.04 (0-0.12)
Targeted transfers	252 (79-524)	42 (5-113)	0.05 (0-0.14)
**Integrated Strategies**			
Family Planning	226 (68-484)	36 (0-107)	0.04 (0-0.13)
Community + Linkages	202 (59-438)	37 (4-97)	0.04 (0-0.12)
Facilities + Linkages	201 (53-413)	36 (0-90)	0.04 (0-0.11)
Facilities + Linkages + Quality	202 (53-413)	36 (0-92)	0.04 (0-0.1)
Comprehensive	180 (50-388)	30 (0-83)	0.03 (0-0.1)

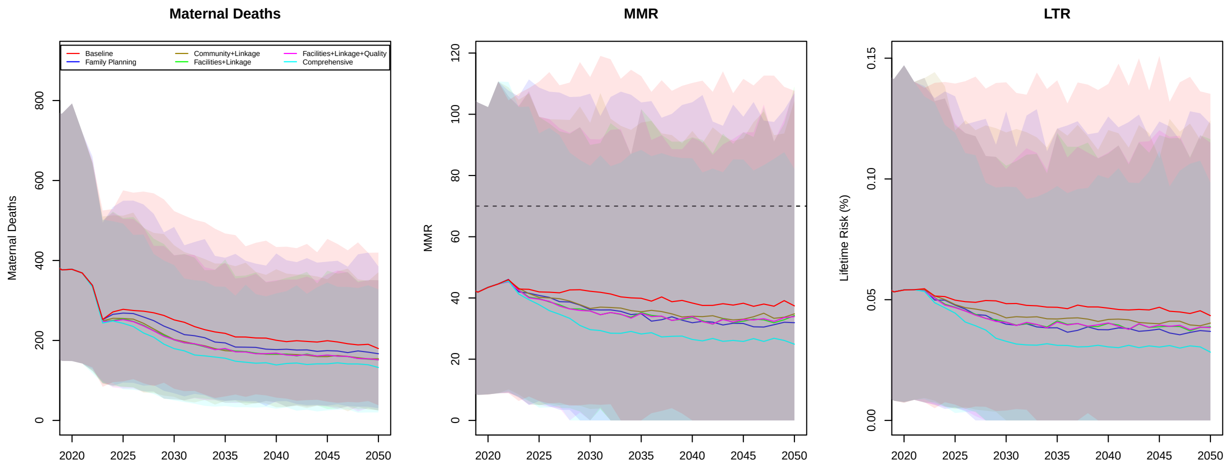

United Arab Emirates

ISO Code	Region	Area	Income Group
ARE	Western Asia	Asia	High income

Projected Maternal Indicators in 2030 by Scenario

Scenario	Maternal Deaths	MMR	LTR
**Baseline**	41 (0-157)	29 (0-175)	0.04 (0-0.23)
**Family Planning Interventions**			
Contraception	33 (0-110)	17 (0-71)	0.03 (0-0.14)
Medical abortion	39 (0-138)	28 (0-179)	0.05 (0-0.22)
**Community-Based Interventions**			
ANC	40 (0-157)	29 (0-175)	0.04 (0-0.23)
SBA	40 (0-150)	29 (0-186)	0.04 (0-0.21)
**Facility-Based Interventions**			
Facility births	40 (0-159)	29 (0-174)	0.04 (0-0.25)
nonEmOC services	40 (0-150)	29 (0-175)	0.04 (0-0.23)
bEmOC services	41 (0-157)	29 (0-175)	0.04 (0-0.23)
cEmOC services	40 (0-160)	29 (0-190)	0.04 (0-0.21)
**System-Relevant Interventions**			
Quality of care	40 (0-159)	29 (0-190)	0.04 (0-0.23)
Referral	40 (0-157)	29 (0-175)	0.04 (0-0.23)
Transport	38 (0-137)	28 (0-165)	0.04 (0-0.21)
Targeted transfers	40 (0-157)	29 (0-175)	0.04 (0-0.23)
**Integrated Strategies**			
Family Planning	33 (0-109)	17 (0-67)	0.03 (0-0.13)
Community + Linkages	39 (0-138)	28 (0-164)	0.04 (0-0.21)
Facilities + Linkages	38 (0-159)	28 (0-191)	0.04 (0-0.23)
Facilities + Linkages + Quality	38 (0-148)	28 (0-187)	0.04 (0-0.23)
Comprehensive	32 (0-98)	17 (0-65)	0.03 (0-0.14)

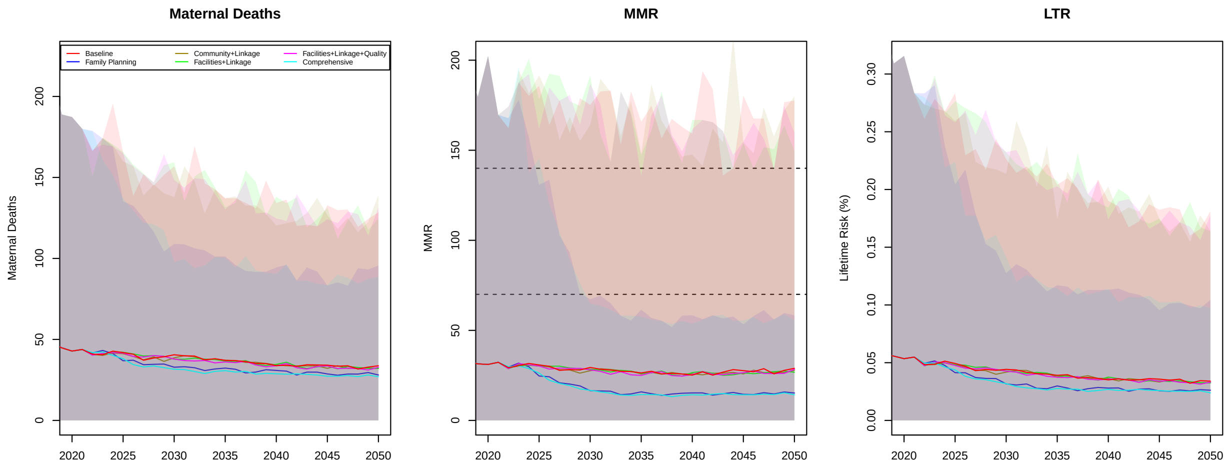

United Kingdom

ISO Code	Region	Area	Income Group
GBR	Northern Europe	Europe	High income

Projected Maternal Indicators in 2030 by Scenario

Scenario	Maternal Deaths	MMR	LTR
**Baseline**	189 (39-382)	7 (0-21)	0.01 (0-0.04)
**Family Planning Interventions**			
Contraception	180 (38-371)	7 (0-23)	0.01 (0-0.04)
Medical abortion	189 (39-384)	7 (0-21)	0.01 (0-0.04)
**Community-Based Interventions**			
ANC	190 (39-388)	7 (0-22)	0.01 (0-0.04)
SBA	188 (39-386)	7 (0-21)	0.01 (0-0.04)
**Facility-Based Interventions**			
Facility births	189 (39-380)	7 (0-21)	0.01 (0-0.04)
nonEmOC services	189 (39-382)	7 (0-21)	0.01 (0-0.04)
bEmOC services	189 (39-382)	7 (0-21)	0.01 (0-0.04)
cEmOC services	189 (40-386)	7 (0-22)	0.01 (0-0.04)
**System-Relevant Interventions**			
Quality of care	189 (39-382)	7 (0-21)	0.01 (0-0.04)
Referral	187 (39-381)	7 (0-21)	0.01 (0-0.04)
Transport	183 (39-359)	7 (0-22)	0.01 (0-0.04)
Targeted transfers	189 (39-382)	7 (0-21)	0.01 (0-0.04)
**Integrated Strategies**			
Family Planning	182 (39-376)	7 (0-23)	0.01 (0-0.04)
Community + Linkages	185 (39-364)	7 (0-22)	0.01 (0-0.04)
Facilities + Linkages	182 (39-380)	7 (0-21)	0.01 (0-0.04)
Facilities + Linkages + Quality	182 (39-380)	7 (0-21)	0.01 (0-0.04)
Comprehensive	173 (39-363)	7 (0-22)	0.01 (0-0.04)

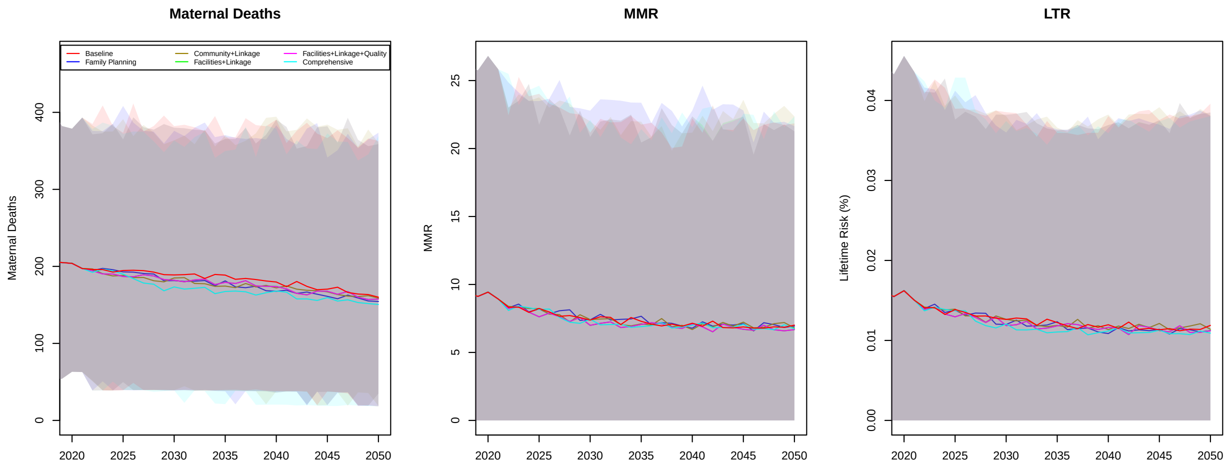

United Republic of Tanzania

ISO Code	Region	Area	Income Group
TZA	Eastern Africa	Africa	Low income

Projected Maternal Indicators in 2030 by Scenario

Scenario	Maternal Deaths	MMR	LTR
**Baseline**	8223 (4181-13424)	244 (145-370)	1.05 (0.49-1.85)
**Family Planning Interventions**			
Contraception	7404 (3872-13003)	228 (128-340)	0.91 (0.41-1.7)
Medical abortion	8037 (4120-12809)	237 (141-344)	1.02 (0.51-1.72)
**Community-Based Interventions**			
ANC	7863 (4052-12821)	232 (134-343)	0.99 (0.45-1.73)
SBA	6730 (3693-10582)	194 (114-291)	0.85 (0.4-1.47)
**Facility-Based Interventions**			
Facility births	3967 (2175-6495)	101 (48-169)	0.45 (0.18-0.86)
nonEmOC services	8201 (4135-13155)	244 (140-369)	1.05 (0.5-1.85)
bEmOC services	8218 (4095-13273)	244 (142-370)	1.04 (0.5-1.84)
cEmOC services	8105 (4168-13267)	240 (135-363)	1.03 (0.47-1.82)
**System-Relevant Interventions**			
Quality of care	8127 (4318-13155)	241 (134-366)	1.04 (0.49-1.85)
Referral	8195 (4271-13477)	243 (144-372)	1.04 (0.51-1.83)
Transport	6968 (3774-11237)	205 (117-315)	0.88 (0.43-1.53)
Targeted transfers	8227 (4257-13362)	244 (143-370)	1.05 (0.49-1.84)
**Integrated Strategies**			
Family Planning	7289 (3895-12193)	223 (130-332)	0.9 (0.4-1.56)
Community + Linkages	5599 (3053-9051)	158 (96-247)	0.69 (0.32-1.23)
Facilities + Linkages	3674 (1850-6330)	94 (41-164)	0.42 (0.16-0.85)
Facilities + Linkages + Quality	3512 (1674-6022)	89 (36-168)	0.4 (0.13-0.81)
Comprehensive	2952 (1463-4792)	74 (29-129)	0.32 (0.1-0.58)

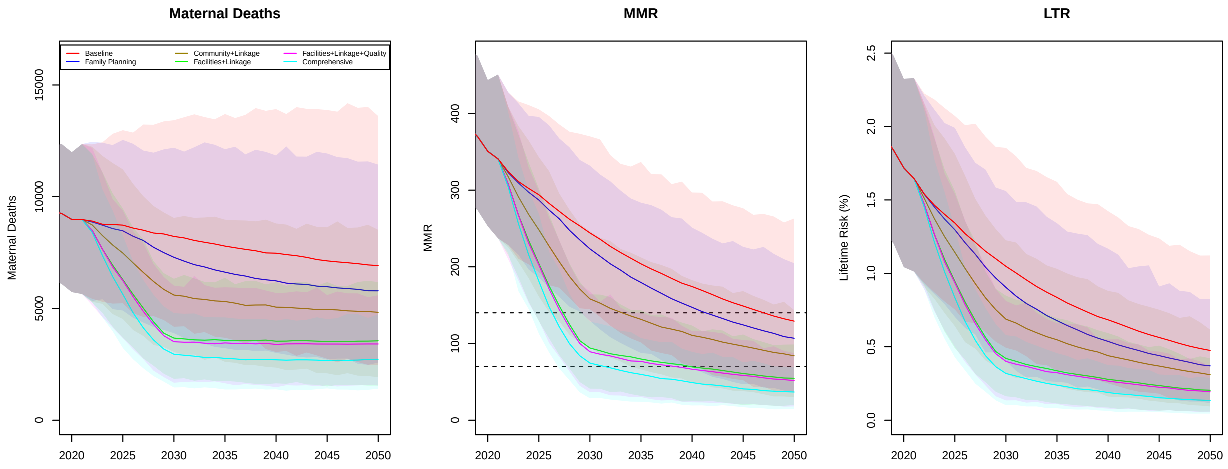

United States of America

ISO Code	Region	Area	Income Group
USA	Northern America	Northern America	High income

Projected Maternal Indicators in 2030 by Scenario

Scenario	Maternal Deaths	MMR	LTR
**Baseline**	1262 (553-2243)	11 (4-20)	0.03 (0.01-0.06)
**Family Planning Interventions**			
Contraception	1229 (522-2313)	11 (3-20)	0.03 (0.01-0.05)
Medical abortion	1261 (543-2303)	11 (3-22)	0.03 (0.01-0.06)
**Community-Based Interventions**			
ANC	1247 (516-2277)	11 (4-22)	0.03 (0.01-0.06)
SBA	1263 (551-2243)	11 (3-20)	0.03 (0.01-0.06)
**Facility-Based Interventions**			
Facility births	1262 (553-2243)	11 (4-20)	0.03 (0.01-0.06)
nonEmOC services	1263 (546-2243)	11 (4-21)	0.03 (0.01-0.06)
bEmOC services	1263 (553-2243)	11 (4-20)	0.03 (0.01-0.06)
cEmOC services	1247 (504-2265)	11 (3-20)	0.03 (0.01-0.06)
**System-Relevant Interventions**			
Quality of care	1174 (472-2168)	11 (3-19)	0.03 (0.01-0.06)
Referral	1260 (553-2226)	11 (4-20)	0.03 (0.01-0.06)
Transport	1238 (511-2317)	11 (3-20)	0.03 (0.01-0.06)
Targeted transfers	1254 (538-2243)	11 (4-21)	0.03 (0.01-0.06)
**Integrated Strategies**			
Family Planning	1222 (512-2270)	11 (3-21)	0.03 (0.01-0.06)
Community + Linkages	1249 (551-2178)	11 (3-21)	0.03 (0.01-0.05)
Facilities + Linkages	1239 (508-2253)	11 (3-21)	0.03 (0.01-0.05)
Facilities + Linkages + Quality	1172 (488-2239)	11 (4-21)	0.03 (0.01-0.06)
Comprehensive	1148 (405-2228)	11 (2-21)	0.03 (0-0.06)

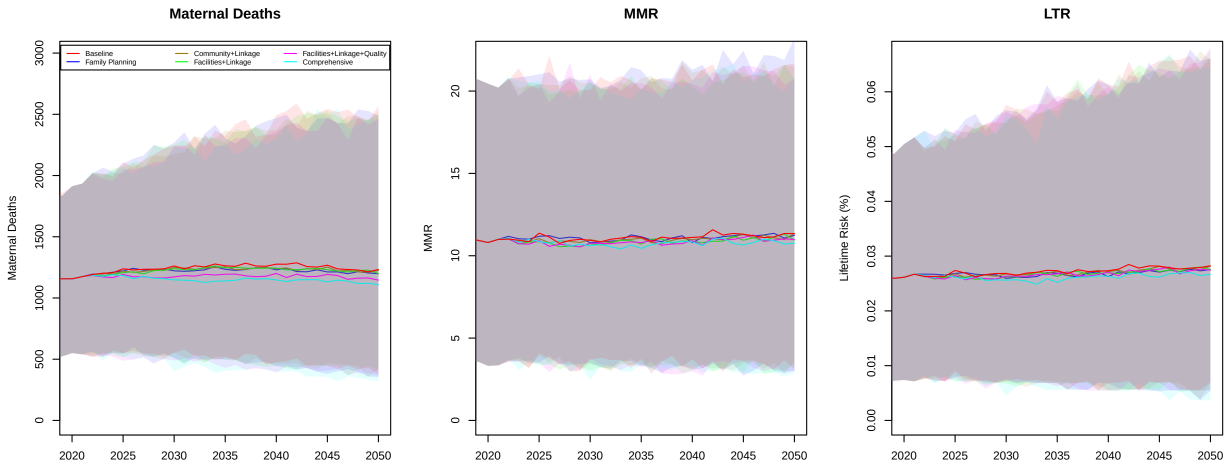

Uruguay

ISO Code	Region	Area	Income Group
URY	South America	Latin America and the Caribbean	High income

Projected Maternal Indicators in 2030 by Scenario

Scenario	Maternal Deaths	MMR	LTR
**Baseline**	46 (16-86)	93 (33-184)	0.18 (0.06-0.35)
**Family Planning Interventions**			
Contraception	41 (14-75)	84 (28-151)	0.15 (0.05-0.29)
Medical abortion	46 (16-86)	93 (33-184)	0.18 (0.06-0.35)
**Community-Based Interventions**			
ANC	47 (15-89)	93 (28-187)	0.18 (0.05-0.35)
SBA	46 (16-85)	92 (31-178)	0.18 (0.06-0.34)
**Facility-Based Interventions**			
Facility births	47 (17-86)	94 (35-183)	0.18 (0.06-0.34)
nonEmOC services	46 (16-86)	93 (33-178)	0.18 (0.06-0.34)
bEmOC services	46 (16-86)	93 (33-184)	0.18 (0.06-0.35)
cEmOC services	47 (15-87)	93 (29-180)	0.18 (0.05-0.35)
**System-Relevant Interventions**			
Quality of care	46 (15-86)	93 (32-182)	0.18 (0.05-0.33)
Referral	46 (15-82)	92 (31-178)	0.18 (0.06-0.32)
Transport	46 (15-86)	93 (30-181)	0.18 (0.06-0.34)
Targeted transfers	46 (16-85)	93 (33-182)	0.18 (0.06-0.34)
**Integrated Strategies**			
Family Planning	41 (14-75)	84 (28-151)	0.15 (0.05-0.29)
Community + Linkages	46 (15-86)	92 (27-178)	0.18 (0.06-0.34)
Facilities + Linkages	45 (14-87)	91 (29-180)	0.17 (0.05-0.34)
Facilities + Linkages + Quality	46 (14-84)	92 (29-179)	0.17 (0.05-0.33)
Comprehensive	40 (12-75)	83 (24-151)	0.15 (0.04-0.3)

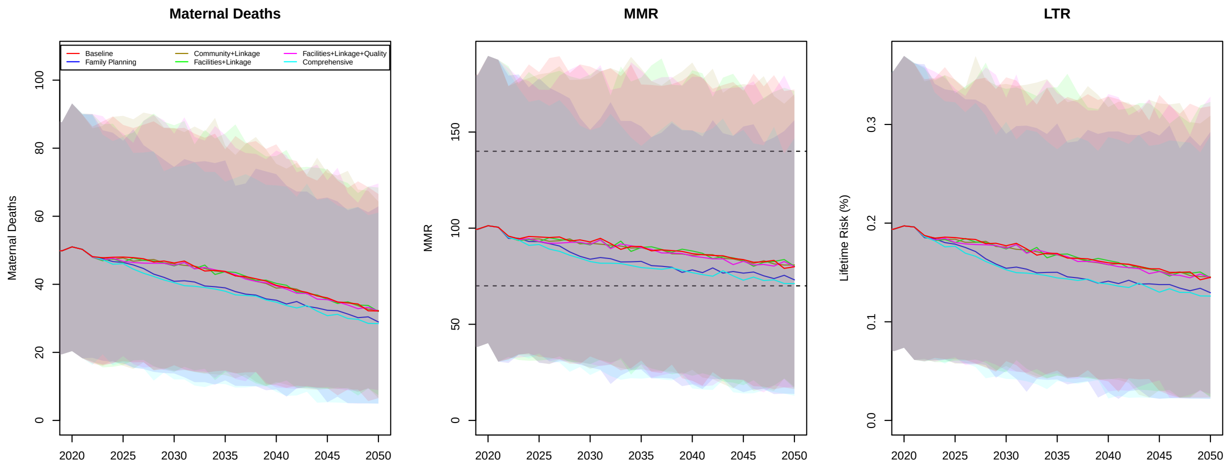

Uzbekistan

ISO Code	Region	Area	Income Group
UZB	Central Asia	Asia	Lower middle income

Projected Maternal Indicators in 2030 by Scenario

Scenario	Maternal Deaths	MMR	LTR
**Baseline**	334 (57-683)	31 (0-86)	0.06 (0-0.17)
**Family Planning Interventions**			
Contraception	324 (51-717)	30 (0-86)	0.06 (0-0.18)
Medical abortion	322 (50-697)	28 (0-82)	0.06 (0-0.17)
**Community-Based Interventions**			
ANC	321 (49-693)	30 (0-86)	0.06 (0-0.18)
SBA	336 (55-691)	31 (0-86)	0.06 (0-0.17)
**Facility-Based Interventions**			
Facility births	332 (70-678)	31 (0-85)	0.06 (0-0.17)
nonEmOC services	333 (53-683)	31 (0-86)	0.06 (0-0.17)
bEmOC services	333 (56-669)	31 (0-89)	0.06 (0-0.17)
cEmOC services	317 (50-659)	28 (0-86)	0.06 (0-0.18)
**System-Relevant Interventions**			
Quality of care	317 (52-672)	29 (0-87)	0.06 (0-0.18)
Referral	335 (57-678)	31 (0-85)	0.06 (0-0.17)
Transport	298 (48-685)	30 (0-86)	0.06 (0-0.19)
Targeted transfers	335 (53-700)	31 (0-88)	0.06 (0-0.17)
**Integrated Strategies**			
Family Planning	309 (52-655)	28 (0-77)	0.06 (0-0.16)
Community + Linkages	300 (49-662)	30 (0-83)	0.06 (0-0.18)
Facilities + Linkages	287 (49-637)	27 (0-83)	0.06 (0-0.17)
Facilities + Linkages + Quality	267 (0-624)	25 (0-80)	0.05 (0-0.16)
Comprehensive	224 (0-531)	20 (0-62)	0.04 (0-0.13)

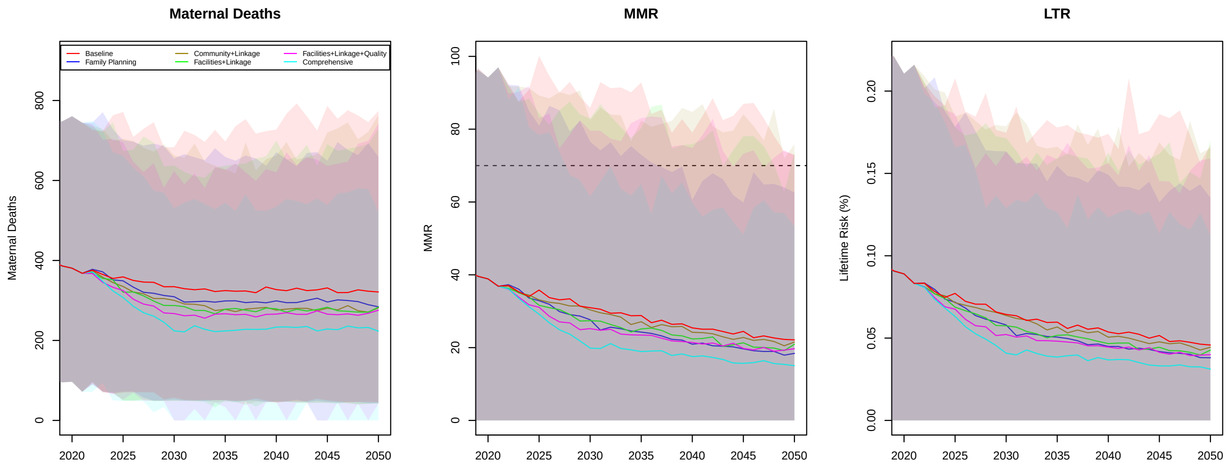

Vanuatu

ISO Code	Region	Area	Income Group
VUT	Melanesia	Oceania	Lower middle income

Projected Maternal Indicators in 2030 by Scenario

Scenario	Maternal Deaths	MMR	LTR
**Baseline**	27 (5-62)	251 (31-566)	0.79 (0.1-1.85)
**Family Planning Interventions**			
Contraception	23 (4-51)	218 (31-484)	0.66 (0.09-1.56)
Medical abortion	23 (3-50)	212 (24-493)	0.68 (0.07-1.59)
**Community-Based Interventions**			
ANC	26 (5-59)	245 (30-573)	0.78 (0.09-1.85)
SBA	27 (5-61)	252 (36-572)	0.8 (0.1-1.88)
**Facility-Based Interventions**			
Facility births	27 (5-62)	251 (30-571)	0.79 (0.09-1.9)
nonEmOC services	26 (5-61)	247 (31-564)	0.78 (0.1-1.87)
bEmOC services	27 (5-62)	249 (31-570)	0.79 (0.09-1.84)
cEmOC services	26 (5-60)	243 (30-579)	0.77 (0.09-1.87)
**System-Relevant Interventions**			
Quality of care	15 (0-41)	125 (0-425)	0.4 (0-1.16)
Referral	27 (5-61)	248 (29-564)	0.79 (0.09-1.89)
Transport	27 (5-59)	248 (37-580)	0.79 (0.09-1.82)
Targeted transfers	27 (4-63)	251 (30-598)	0.8 (0.09-1.9)
**Integrated Strategies**			
Family Planning	22 (3-50)	208 (27-493)	0.64 (0.08-1.57)
Community + Linkages	26 (4-59)	246 (30-573)	0.78 (0.09-1.82)
Facilities + Linkages	26 (5-60)	243 (44-575)	0.77 (0.12-1.83)
Facilities + Linkages + Quality	14 (0-40)	118 (0-403)	0.38 (0-1.21)
Comprehensive	9 (0-25)	75 (0-214)	0.24 (0-0.72)

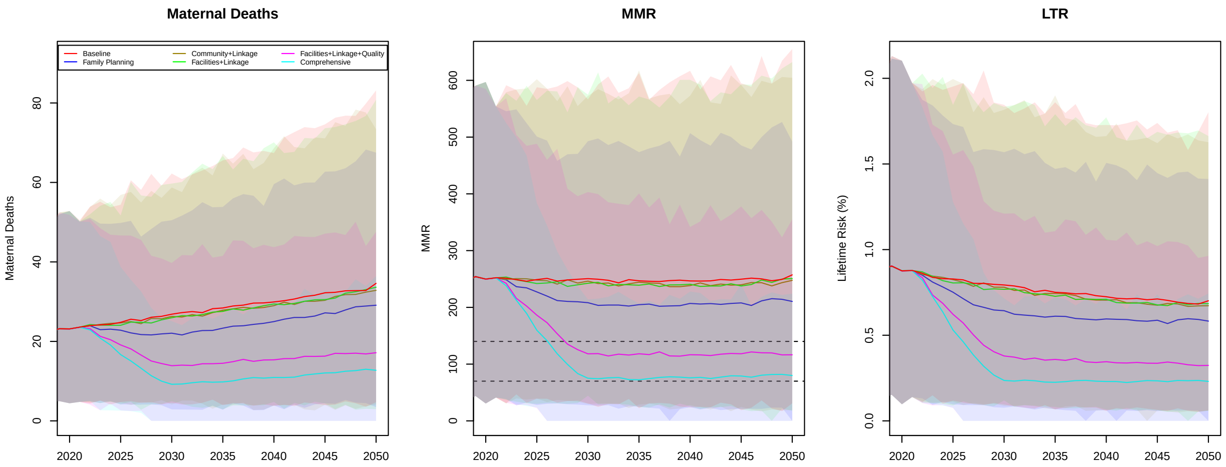

Venezuela (Bolivarian Republic of)

ISO Code	Region	Area	Income Group
VEN	South America	Latin America and the Caribbean	Upper middle income

Projected Maternal Indicators in 2030 by Scenario

Scenario	Maternal Deaths	MMR	LTR
**Baseline**	340 (75-677)	45 (7-91)	0.11 (0.01-0.23)
**Family Planning Interventions**			
Contraception	260 (38-582)	39 (6-84)	0.08 (0.01-0.19)
Medical abortion	340 (75-677)	45 (7-91)	0.11 (0.01-0.23)
**Community-Based Interventions**			
ANC	339 (76-658)	45 (7-92)	0.11 (0.01-0.23)
SBA	340 (75-677)	45 (7-91)	0.11 (0.01-0.23)
**Facility-Based Interventions**			
Facility births	340 (75-677)	45 (7-91)	0.11 (0.01-0.23)
nonEmOC services	342 (75-683)	45 (7-94)	0.11 (0.01-0.24)
bEmOC services	339 (75-674)	45 (7-93)	0.11 (0.01-0.24)
cEmOC services	337 (73-677)	44 (7-92)	0.11 (0.01-0.23)
**System-Relevant Interventions**			
Quality of care	299 (75-568)	38 (6-79)	0.09 (0.01-0.2)
Referral	340 (76-678)	44 (7-91)	0.11 (0.02-0.23)
Transport	330 (73-654)	44 (8-95)	0.11 (0.02-0.24)
Targeted transfers	340 (75-674)	45 (7-91)	0.11 (0.01-0.24)
**Integrated Strategies**			
Family Planning	260 (38-582)	39 (6-84)	0.08 (0.01-0.19)
Community + Linkages	326 (73-648)	44 (7-91)	0.1 (0.01-0.24)
Facilities + Linkages	326 (72-641)	43 (7-90)	0.1 (0.01-0.22)
Facilities + Linkages + Quality	283 (64-562)	37 (6-81)	0.09 (0.01-0.19)
Comprehensive	209 (36-444)	31 (0-71)	0.06 (0-0.15)

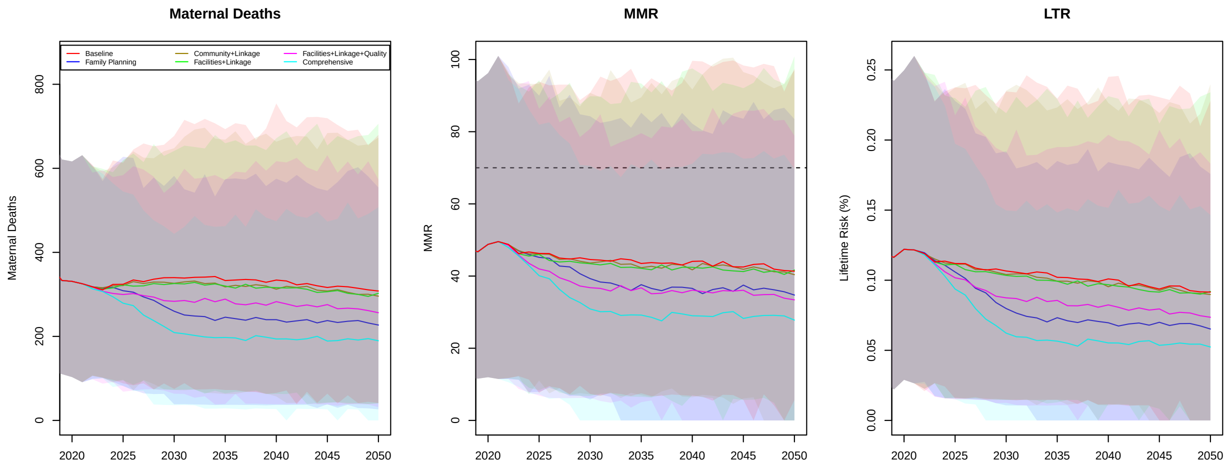

Viet Nam

ISO Code	Region	Area	Income Group
VNM	South-Eastern Asia	Asia	Lower middle income

Projected Maternal Indicators in 2030 by Scenario

Scenario	Maternal Deaths	MMR	LTR
**Baseline**	2469 (1020-4716)	98 (29-211)	0.24 (0.06-0.53)
**Family Planning Interventions**			
Contraception	2389 (940-4654)	97 (29-203)	0.23 (0.06-0.51)
Medical abortion	2460 (971-4798)	97 (29-201)	0.24 (0.06-0.53)
**Community-Based Interventions**			
ANC	2395 (996-4490)	94 (30-195)	0.23 (0.06-0.5)
SBA	2439 (957-4638)	96 (30-202)	0.23 (0.06-0.52)
**Facility-Based Interventions**			
Facility births	2349 (964-4361)	90 (30-180)	0.22 (0.06-0.48)
nonEmOC services	2453 (996-4753)	97 (30-207)	0.24 (0.06-0.53)
bEmOC services	2460 (996-4766)	97 (30-209)	0.24 (0.06-0.54)
cEmOC services	2373 (924-4631)	92 (27-199)	0.22 (0.05-0.52)
**System-Relevant Interventions**			
Quality of care	1378 (751-2232)	42 (18-79)	0.1 (0.04-0.21)
Referral	2450 (974-4701)	96 (29-207)	0.23 (0.06-0.52)
Transport	2395 (928-4640)	95 (29-203)	0.23 (0.06-0.53)
Targeted transfers	2446 (996-4708)	96 (31-205)	0.24 (0.06-0.52)
**Integrated Strategies**			
Family Planning	2389 (952-4712)	97 (30-203)	0.23 (0.06-0.52)
Community + Linkages	2318 (888-4340)	92 (28-189)	0.22 (0.05-0.48)
Facilities + Linkages	2196 (878-4337)	85 (25-179)	0.21 (0.05-0.48)
Facilities + Linkages + Quality	1108 (515-1865)	31 (12-60)	0.07 (0.02-0.16)
Comprehensive	1058 (484-1851)	30 (12-57)	0.07 (0.02-0.16)

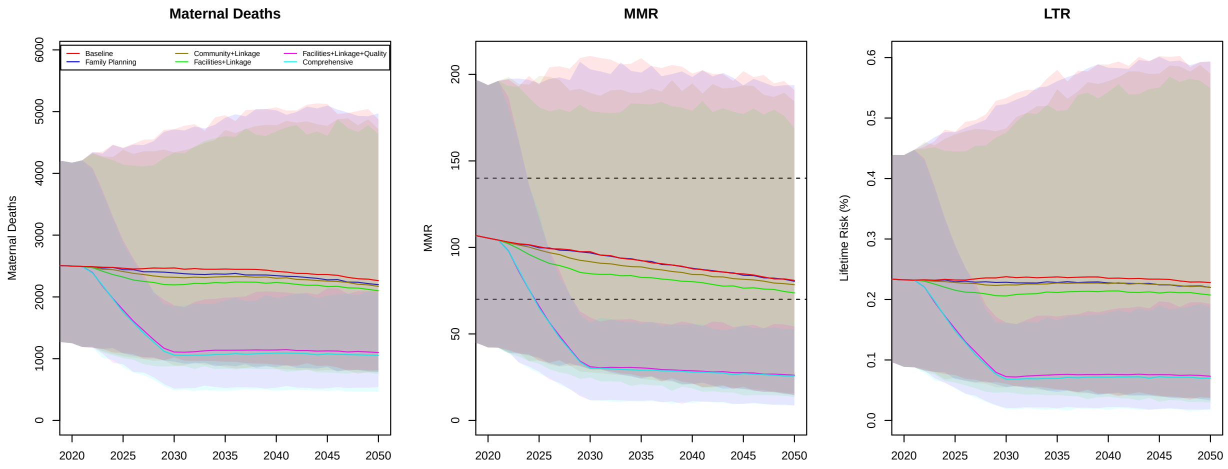

Yemen

ISO Code	Region	Area	Income Group
YEM	Western Asia	Asia	Low income

Projected Maternal Indicators in 2030 by Scenario

Scenario	Maternal Deaths	MMR	LTR
**Baseline**	4424 (1748-7754)	315 (111-539)	1.05 (0.31-1.96)
**Family Planning Interventions**			
Contraception	4377 (1661-7727)	312 (114-529)	1.04 (0.34-1.94)
Medical abortion	4407 (1736-7768)	314 (112-523)	1.05 (0.36-1.95)
**Community-Based Interventions**			
ANC	4305 (1583-7265)	305 (101-508)	1.02 (0.32-1.84)
SBA	3963 (1556-6951)	278 (96-493)	0.93 (0.32-1.75)
**Facility-Based Interventions**			
Facility births	1681 (690-3071)	89 (28-178)	0.31 (0.08-0.66)
nonEmOC services	4433 (1706-7727)	316 (110-537)	1.06 (0.33-1.97)
bEmOC services	4421 (1719-7753)	315 (102-540)	1.06 (0.34-1.98)
cEmOC services	4352 (1542-7348)	309 (100-513)	1.03 (0.32-1.9)
**System-Relevant Interventions**			
Quality of care	4353 (1443-7624)	309 (84-533)	1.03 (0.27-1.95)
Referral	4394 (1678-7501)	313 (104-534)	1.05 (0.35-1.9)
Transport	3802 (1528-6640)	265 (98-449)	0.89 (0.29-1.66)
Targeted transfers	4410 (1559-7665)	313 (91-532)	1.05 (0.29-1.93)
**Integrated Strategies**			
Family Planning	4362 (1687-7725)	311 (109-525)	1.04 (0.35-1.95)
Community + Linkages	2871 (1174-5086)	188 (69-330)	0.63 (0.22-1.21)
Facilities + Linkages	1328 (564-2319)	64 (20-130)	0.22 (0.06-0.49)
Facilities + Linkages + Quality	1117 (440-1989)	46 (12-99)	0.16 (0.03-0.36)
Comprehensive	1050 (386-1787)	41 (10-85)	0.14 (0.03-0.33)

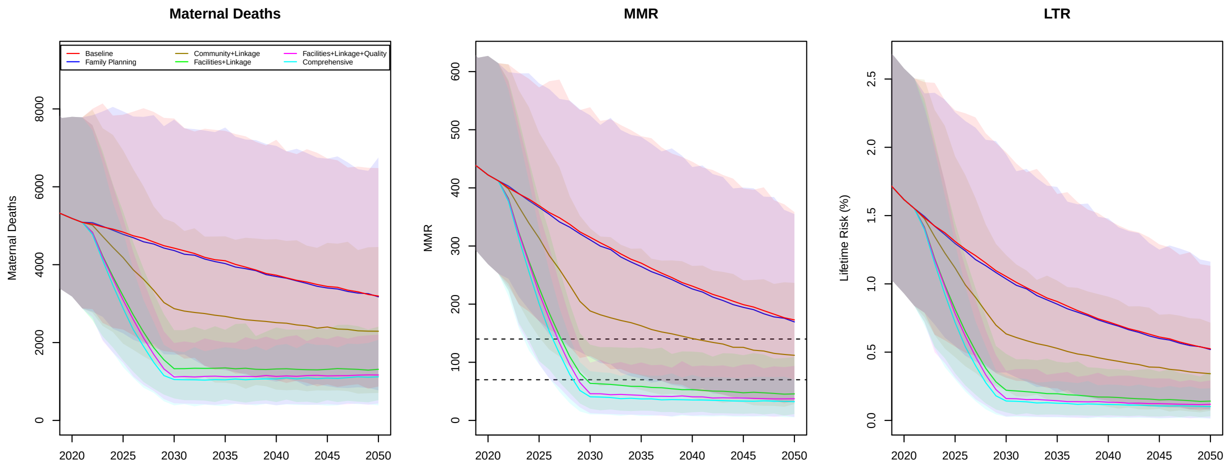

Zambia

ISO Code	Region	Area	Income Group
ZMB	Eastern Africa	Africa	Lower middle income

Projected Maternal Indicators in 2030 by Scenario

Scenario	Maternal Deaths	MMR	LTR
**Baseline**	1227 (567-2185)	126 (55-227)	0.52 (0.22-0.97)
**Family Planning Interventions**			
Contraception	1133 (485-1997)	118 (49-202)	0.47 (0.17-0.83)
Medical abortion	1161 (594-2117)	118 (58-200)	0.48 (0.21-0.88)
**Community-Based Interventions**			
ANC	1202 (549-2200)	123 (52-211)	0.5 (0.2-0.93)
SBA	1182 (556-2164)	121 (52-214)	0.49 (0.21-0.94)
**Facility-Based Interventions**			
Facility births	914 (354-1751)	88 (29-180)	0.36 (0.1-0.77)
nonEmOC services	1232 (580-2211)	127 (57-224)	0.52 (0.23-0.97)
bEmOC services	1236 (567-2246)	127 (55-224)	0.52 (0.21-0.96)
cEmOC services	1207 (536-2225)	124 (54-213)	0.5 (0.2-0.93)
**System-Relevant Interventions**			
Quality of care	962 (424-1824)	95 (38-179)	0.39 (0.13-0.76)
Referral	1222 (547-2284)	126 (54-230)	0.52 (0.21-1.03)
Transport	1108 (501-2006)	116 (49-206)	0.47 (0.2-0.9)
Targeted transfers	1235 (568-2217)	127 (55-231)	0.52 (0.22-1.02)
**Integrated Strategies**			
Family Planning	1099 (481-2000)	114 (48-199)	0.45 (0.16-0.84)
Community + Linkages	1008 (428-1874)	105 (42-186)	0.42 (0.16-0.83)
Facilities + Linkages	847 (302-1682)	86 (24-176)	0.35 (0.09-0.76)
Facilities + Linkages + Quality	557 (171-1184)	51 (12-131)	0.21 (0.03-0.56)
Comprehensive	447 (152-914)	40 (8-95)	0.16 (0.03-0.4)

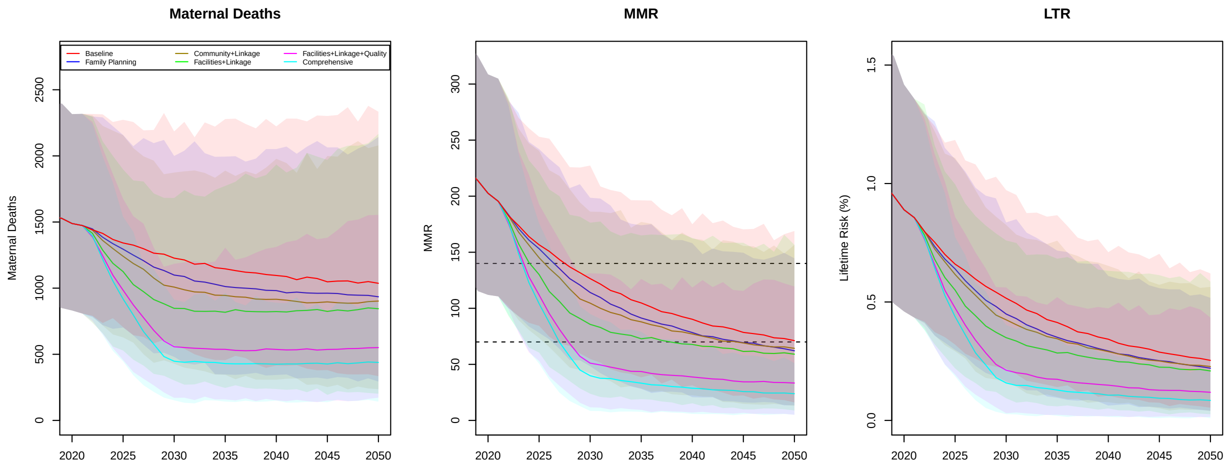

Zimbabwe

ISO Code	Region	Area	Income Group
ZWE	Eastern Africa	Africa	Low income

Projected Maternal Indicators in 2030 by Scenario

Scenario	Maternal Deaths	MMR	LTR
**Baseline**	1156 (360-2608)	190 (59-373)	0.57 (0.15-1.41)
**Family Planning Interventions**			
Contraception	1152 (396-2647)	186 (61-372)	0.57 (0.16-1.41)
Medical abortion	1150 (393-2705)	189 (63-389)	0.57 (0.16-1.46)
**Community-Based Interventions**			
ANC	1142 (373-2674)	187 (59-375)	0.56 (0.14-1.4)
SBA	1137 (360-2793)	187 (55-382)	0.57 (0.13-1.47)
**Facility-Based Interventions**			
Facility births	813 (262-1799)	126 (37-258)	0.39 (0.08-0.97)
nonEmOC services	1149 (360-2705)	189 (61-383)	0.57 (0.14-1.47)
bEmOC services	1143 (358-2651)	188 (59-381)	0.57 (0.14-1.46)
cEmOC services	1134 (376-2644)	186 (55-387)	0.56 (0.15-1.45)
**System-Relevant Interventions**			
Quality of care	1084 (345-2609)	178 (52-365)	0.54 (0.14-1.38)
Referral	1090 (356-2513)	177 (56-349)	0.54 (0.15-1.34)
Transport	1099 (333-2601)	183 (57-360)	0.55 (0.14-1.4)
Targeted transfers	1151 (339-2657)	190 (60-386)	0.57 (0.14-1.46)
**Integrated Strategies**			
Family Planning	1160 (390-2698)	186 (57-379)	0.57 (0.14-1.46)
Community + Linkages	991 (305-2402)	163 (52-343)	0.49 (0.12-1.28)
Facilities + Linkages	758 (226-1805)	119 (29-262)	0.37 (0.07-0.98)
Facilities + Linkages + Quality	726 (204-1680)	115 (26-254)	0.35 (0.06-0.93)
Comprehensive	708 (199-1679)	107 (23-231)	0.34 (0.06-0.91)

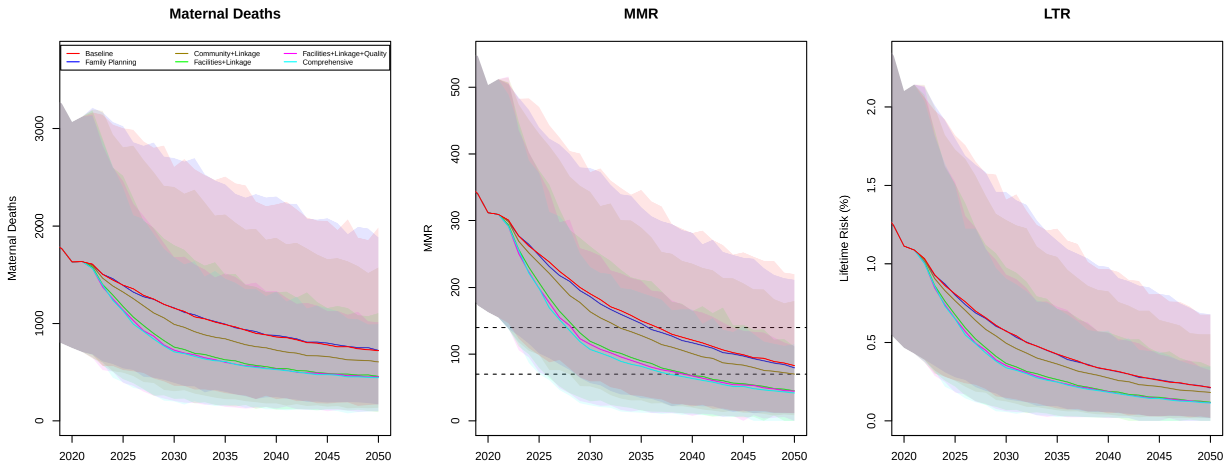

Supplement: Supplementary file 1 — Supplementary Appendix 1 (country results). [file 41591_2023_2311_MOESM1_ESM.pdf]
